# Inferring the effective reproductive number from deterministic and semi-deterministic compartmental models using incidence and mobility data

**DOI:** 10.1371/journal.pcbi.1010206

**Published:** 2022-06-27

**Authors:** Jair Andrade, Jim Duggan

**Affiliations:** 1 Data Science Institute and School of Computer Science, National University of Ireland Galway, Ireland; 2 School of Computer Science, Ryan Institute and Data Science Institute, National University of Ireland Galway, Ireland; University of Washington, UNITED STATES

## Abstract

The effective reproduction number (ℜ_*t*_) is a theoretical indicator of the course of an infectious disease that allows policymakers to evaluate whether current or previous control efforts have been successful or whether additional interventions are necessary. This metric, however, cannot be directly observed and must be inferred from available data. One approach to obtaining such estimates is fitting compartmental models to incidence data. We can envision these dynamic models as the ensemble of structures that describe the disease’s natural history and individuals’ behavioural patterns. In the context of the response to the COVID-19 pandemic, the assumption of a constant transmission rate is rendered unrealistic, and it is critical to identify a mathematical formulation that accounts for changes in contact patterns. In this work, we leverage existing approaches to propose three complementary formulations that yield similar estimates for ℜ_*t*_ based on data from Ireland’s first COVID-19 wave. We describe these Data Generating Processes (DGP) in terms of State-Space models. Two (DGP1 and DGP2) correspond to stochastic process models whose transmission rate is modelled as Brownian motion processes (Geometric and Cox-Ingersoll-Ross). These DGPs share a measurement model that accounts for incidence and transmission rates, where mobility data is assumed as a proxy of the transmission rate. We perform inference on these structures using Iterated Filtering and the Particle Filter. The final DGP (DGP3) is built from a pool of deterministic models that describe the transmission rate as information delays. We calibrate this pool of models to incidence reports using Hamiltonian Monte Carlo. By following this complementary approach, we assess the tradeoffs associated with each formulation and reflect on the benefits/risks of incorporating proxy data into the inference process. We anticipate this work will help evaluate the implications of choosing a particular formulation for the dynamics and observation of the time-varying transmission rate.

## Author summary

Policymakers use the effective reproduction number (ℜ_*t*_) to determine whether an epidemic is growing (ℜ_*t*_ > 1) or shrinking (ℜ_*t*_ < 1). One can estimate this quantity by simulating compartmental models fitted to data. These models can be seen as the ensemble of two structures: one that describes the course of a disease in an individual and another one that accounts for behavioural patterns. Nevertheless, these estimates are sensitive to the assumptions embedded in the model, such as the formulation of the time-varying transmission rate. In this paper, we couple an SEIR-type structure with three complementary formulations: 1) non-negative random-walks (Geometric Brownian Motion) 2) non-negative random-walks pulled toward a long-term goal (Cox-Ingersoll-Ross) 3) Gradual approximations towards a long-term goal (exponential smoothing). We refer to each coupling as a Data Generating Process (DGP). In essence, we simulate trajectories from these DGPs to identify plausible sets of transmission rates (based on incidence and mobility data) that explain Ireland’s first COVID-19 wave. Here, we assume that mobility data is a proxy measurement for the transmission rate. These DGPs yield similar average estimates for ℜ_*t*_, albeit with dissimilar degrees of uncertainty. Finally, we reflect on the tradeoffs of choosing each particular formulation.

## Introduction

Since early 2020, SARS coronavirus 2 (SARS-CoV-2) has spread throughout the seven continents, causing a COVID-19 pandemic of catastrophic consequences, including the loss of millions of lives and jobs. In the early days of the pandemic, given the absence of vaccines and the lack of effective therapeutics, governments primarily relied on non-pharmaceutical interventions (NPIs) to reduce the transmission of SARS-CoV-2, thereby lowering the death toll. Although effective in preventing deaths [1], NPIs such as mobility restrictions and stay-at-home orders impose a burden on society with economic and psychological costs [2]. In addition to this, the effectiveness of these interventions wanes over time as compliance progressively diminishes. Following these considerations, policymakers strive to find an adequate balance between the interventions’ severity and acceptable transmission levels. In this decision-making process, the effective reproduction number plays a crucial role. Briefly, the effective reproduction number, ℜ_*t*_, is the time-varying average number of secondary cases caused by a primary case at a calendar time *t* [3, 4], and it is a theoretical indicator of the course of an infectious process [5]. Above the *epidemic threshold* (ℜ_*t*_ > 1), each infectious person leads to more than one secondary infectious person, and the disease is (re)emerging [6]; below that threshold, there is limited secondary transmission. In practice, policymakers can use ℜ_*t*_ in two ways. First, as a guide to assess in near real-time whether the interventions are succeeding (ℜ_*t*_ < 1) or whether it is required to increment the response’s strength [4]. Second, in retrospective analyses to assess how policy decisions, population immunity, and other factors have impacted transmission at specific points in time [7].

Generally speaking, ℜ_*t*_ is the result of a combination of intrinsic (decline in susceptible individuals) and extrinsic (change in contact patterns due to the implementation of control measures) factors [4], for which there are no readily available measurements. One, therefore, must resort to statistical methods to obtain an approximation of this epidemic indicator. On one end of the spectrum, we find widely applicable and context-independent empirical methods such as the two-step Bayesian procedure proposed by Cori and colleagues [8, 9] and the likelihood-based estimation procedure proposed by Wallinga and Teunis [10]. At the other end of the spectrum, we can infer ℜ_*t*_ from compartmental models calibrated to incidence data [11], which is the focus of this paper. In addition to serving as vehicles to obtain estimates, these mechanistic models are based on a scientific understanding of infectious disease dynamics [12], which one can interpret as a dynamic hypothesis of the underlying process that produces the observable behaviour patterns. This feature implies that fitting a compartmental model to data also tests a hypothesis that links structure to behaviour [13]. It thus follows that parameter estimates derived from this procedure have an interpretation in the real world. Notwithstanding these advantages, ℜ_*t*_ estimates from compartmental models are sensitive to data availability and assumptions in the model structure [7].

One such assumption is the transmission rate’s dynamics. In the context of the COVID-19 pandemic, the assumption of a constant transmission rate is rendered unrealistic, apart from a few days in the initial phase of the outbreak [14, 15]. The rationale is that under the imminent surge of cases, governments implemented NPIs at early stages to reduce the number of contacts among the population. Modellers thus are required to describe formally the changes in the transmission rate over time. For instance, in measles studies [16–18], it is not unusual to assume *term-time forcing* structures [19], where the contact rate experiences sudden changes in time (e.g., because of school holidays). Other approaches have adopted *smoothly-varying functions* [19] to model the transmission rate in tuberculosis outbreaks [20]. In COVID-19 analyses, the transmission rate has been described as episodes of constant contact rates separated by change points where a transition occurs [14, 21]. These are likely once-off models, more appropriate for retrospective analyses, whose formulations are not designed to incorporate new data that account for policy changes (unless the structure is modified).

Nevertheless, ascertaining which deterministic formulation is the most adequate is far from straightforward. Its search involves several *trial-and-error* iterations and model comparisons until a satisfactory structure is found. If one aims for near real-time estimates, random-walk formulations offer a flexible device to *uncover* the underlying transmission rate dynamics [22]. This type of structure does not impose stringent constraints on the transmission’s rate shape, facilitating the incorporation of new data without structural modifications. This approach has been applied to studying an influenza pandemic [22, 23] and Ebola outbreaks [24, 25]. Although random-walk models yield fits to incidence data, the match between observed and simulated data may be achieved at the expense of large uncertainty bounds. Moreover, under this framework, the inference of time-independent parameters requires burdensome computational efforts. More recently, the extensive research provoked by the COVID-19 pandemic prompted researchers to use non-traditional sources of data to infer the transmission rate. In particular, mobility data has been assumed as a proxy for the changes in the transmission rate [26]. In doing so, the dynamics exhibited by the transmission rate have an inherently plausible explanation (changes in human behaviour measured by mobile devices) so that models can more easily incorporate new incidence measurements. However, it should be mentioned that this approach entails a stringent assumption wherein one tacitly assumes a perfect correlation between changes in mobility data and effective contact patterns. Thus, discrepancies between actual and assumed transmission rates may result in unnecessary corrections to the estimates of other unknown parameters.

**Fig 1:**
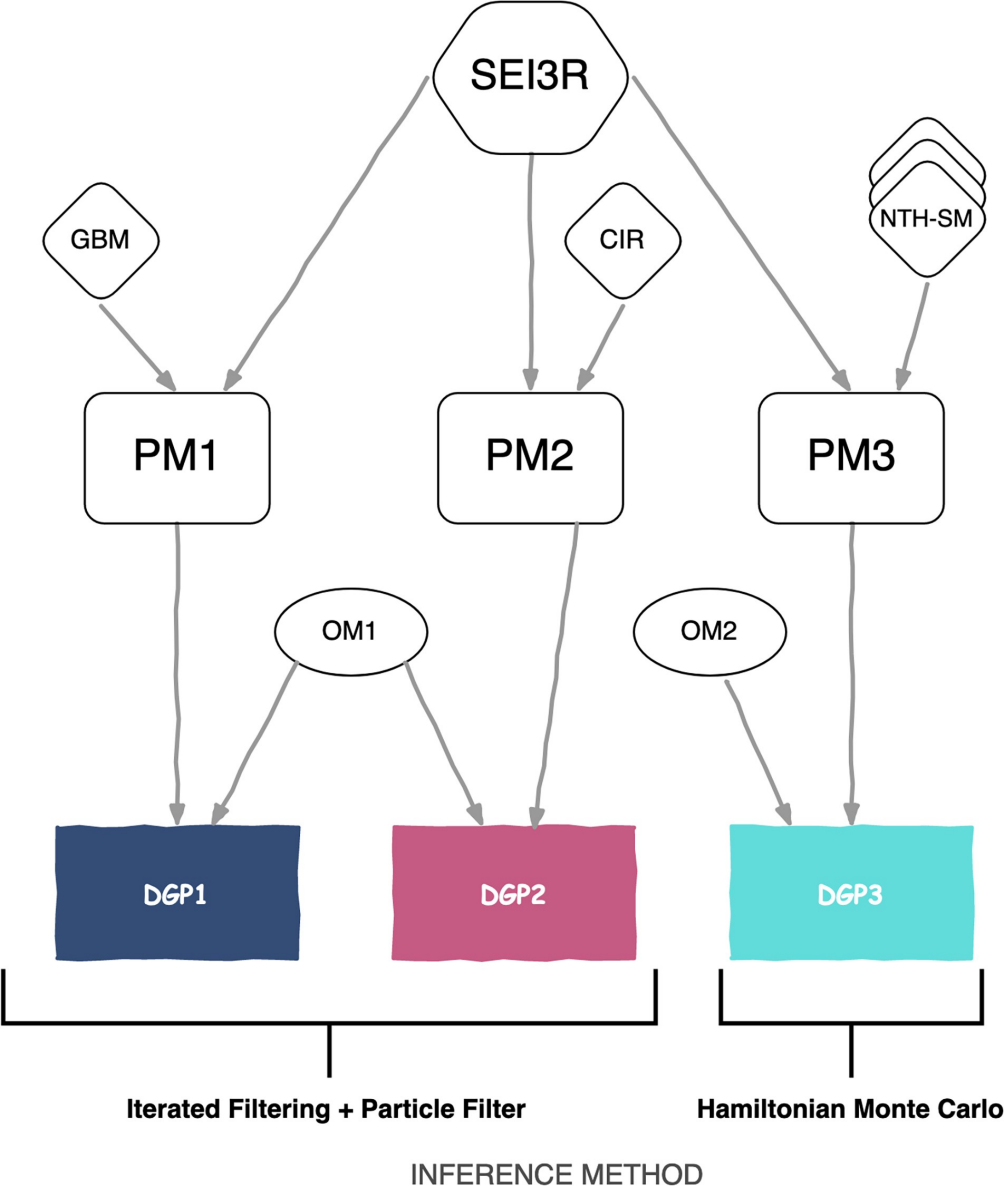
Schematic diagram of the data generating processes (DGPs) explored in this paper. This diagram aims to portray the DGPs as the ensemble of two components: a measurement or observational model (ellipse) and a process model (rounded rectangle). For instance, DGP1 is the amalgamation of the measurement model OM1 and the process model PM1. The process model is in turn the ensamble of two structures: a within-host profile (hexagon) and a time-dependent transmission rate (rhombus). Whereas all process models share a common within-host profile (SEI3R), they differ in the formulation of the transmission rate: Geometric Brownian Motion (GBM), Cox-Ingersoll-Ross (CIR), and nth-order exponential smoothing (NTH-SM). The inference method employed on each DGP depends upon the nature of the process model (Iterated Filtering + Particle Filter for stochastic structures and Hamiltonian Monte Carlo for deterministic ones).

Consequently, this paper aims to draw upon the strengths of the approaches described above to formulate a complementary process for estimating ℜ_*t*_ from compartmental models. Specifically, we build three structures or Data Generating Processes (DGP) that accounts for Ireland’s first COVID-19 wave. Two DGPs incorporate stochastic features in the transmission rate, whereas the other formulation is exclusively deterministic. These structures are complementary in the sense that the results obtained from one DGP inform the subsequent one. Below, we describe each DGP in detail, the inference process to obtain estimates for ℜ_*t*_ and other unknown quantities (Fig 1), and finally, discuss the results. All the analysis is performed in R, mainly supported by the statistical packages *pomp* [27] and *Stan* [28]. The code is freely available at https://github.com/jandraor/time_varying_beta.

## Results

### Context

**Fig 2:**
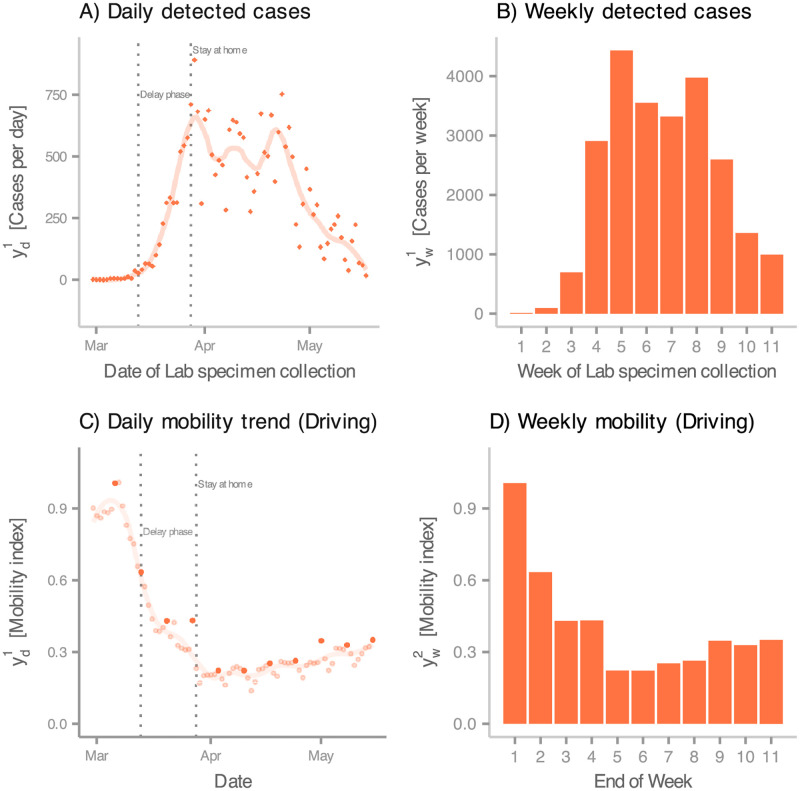
Incidence and mobility data. (A) Daily number (rhombus-shaped points) of COVID-19 cases detected during Ireland’s first wave, from the 29th of February 2020 to the 17th of May 2020. The x-axis indicates the date in which the infected individuals were swabbed. The line represents the smoothed trend (via LOESS method) from the data (B) Weekly number of COVID-19 cases detected in during Ireland’s first wave. The x-axis indicates the number of weeks since the first case was detected. (C) Apple data for Ireland from the 29th of February 2020 to the 17th of May 2020. Points represent the normalised amount of daily requests for driving directions. These indexes are normalised to the value on the 28th of February 2020. We highlight points every 7 days. These highlighted points are used to calibrate DGP1 and DGP2. The line represents the smoothed trend (via LOESS method) from the data. (D) Normalised amount of daily requests for driving directions at the end of each week starting from the 29th of February 2020. These bars correspond to the highlighted points in C.

By the end of February 2020, more than sixty countries had detected at least one case of COVID-19 [29], including Ireland, where the first confirmed case was announced on *the 29th of February*. Twelve days after this event, the Irish Government ordered the closure of all schools, colleges, and childcare facilities, followed by a stricter *stay-at-home* mandate implemented on *the 27th of March*. These interventions resulted in low incidence and mortality rates, which allowed easing the restrictions from mid-May. In Fig 2A and 2B, respectively, we present the number of daily ($y_{d}^{1}$) and weekly cases ($y_{w}^{1}$) detected from the first report up to the point where the restrictions began to be lifted, a period that we refer to as the *first wave*.

In a nutshell, stay-at-home orders and similar measures aim to restrict the movements of a population so that the risk of exposure to a transmissible pathogen is reduced. Impractical several years ago, the advent of smartphones has permitted us to gauge patterns in population mobility in real-time. For instance, since *the 13th of January, 2020*, Apple has provided an index that quantifies the level of mobility by transportation type (driving, transit, and walking). Apple generates this data by counting the number of requests made to *Apple Maps* for directions. Fig 2C shows Ireland’s daily driving mobility levels during the first wave ($y_{d}^{2}$), and Fig 2D, the value at the end of each week ($y_{w}^{2}$) from *the 29th of February 2020*. This dataset, along with the incidence reports (S1 Data), will serve as the basis to calibrate the proposed compartmental models below.

### State-Space models


$$\begin{array}{c} X_{t}∼p_{X,t}^{\theta }\left(x_{t}|x_{t-1}\right) \end{array}$$ (1)

$$\begin{array}{c} Y_{t}∼p_{Y,t}^{\theta }\left(y_{t}|x_{t}\right) \end{array}$$ (2)

One can frame the inference process for compartmental structures following the terminology provided by state-space models (SSM) [30], also known as Partially observed Markov process models [31]. Through an *SSM*, one conceives a DGP as a generative probabilistic model that consists of two discrete-time Markovian mechanisms. The first mechanism (Eq 1) describes the evolution over time of the system’s latent states (*X*), where *X*_*t*_ is drawn conditionally on the previous state of the latent process (*X*_*t*−1_) according to the density $p_{X,t}^{\theta }\left(x_{t}|x_{t-1}\right)$. Therefore, the DGP is a Markov chain [32], as the state of the latent variable at time *t* depends only on its previous state and the distribution from which it comes. In the literature, Eq 1 is often referred to as the *latent process model* [16] or the *system model* [33]. Intuitively, this formulation corresponds to the set of causal assumptions (*dynamic hypothesis*) that explains a phenomenon of interest in terms of states and transitions (rates). The process model may be defined in continuous or discrete time [31], but only its distribution at discrete times is considered (*X*_*t*_, *X*_*t*+1_, *X*_*t*+2_, …, *X*_*t*+*h*_), where *t* ≥ 1 and *h* is an integer. For simplicity, we assume that *X*_0_ is known.

In epidemiology, it is commonplace to represent the process model via compartmental structures in which individuals are categorised according to their infection status [34]. We refer to this categorisation as the *within-host profile*. Formally, one can employ a system of differential equations to build such compartmental models. The reader should recall that any system of ordinary differential equations $\frac{dx}{dt}=f\left(x\right)$ is Markovian. Here, we adopt the *SEI3R* profile [15, 35], an extension of the SEIR framework. Under this profile, we stratify individuals as susceptible (*S*_*t*_), exposed (*E*_*t*_), infectious, and recovered (*R*_*t*_). We further disaggregate the infectious class by medical status, resulting in three compartments: *preclinical* (*P*_*t*_), *clinical* (*I*_*t*_), and *subclinical* (*A*_*t*_) (see Materials and methods section for the complete description). The three DGPs presented in this paper share the SEI3R profile (Fig 1).

On the other hand, the exact state of the population at any given time is generally not observable and must be inferred from available data via statistical inference [36]. It is thus necessary to formally relate (Eq 2), at each discrete time (*t* ≥ 0), latent states to *noisy* measurements via a *measurement* or *observational* model [33], where each *Y*_*t*_ is drawn conditionally on the most recent state of the latent variable, according to the density $p_{Y,t}^{\theta }\left(y_{t}|x_{t}\right)$. This work draws on incidence and mobility data to formulate such measurement models.

### DGP1—Geometric Brownian Motion


$$\begin{array}{c} \beta_{t}=\zeta Z_{t} \end{array}$$ (3)

$$\begin{array}{c} \frac{dZ}{dt}=\alpha Z_{t}dW \end{array}$$ (4)

$$\begin{array}{c} dW∼Normal(0,\sqrt{dt}) \end{array}$$ (5)

**Fig 3:**
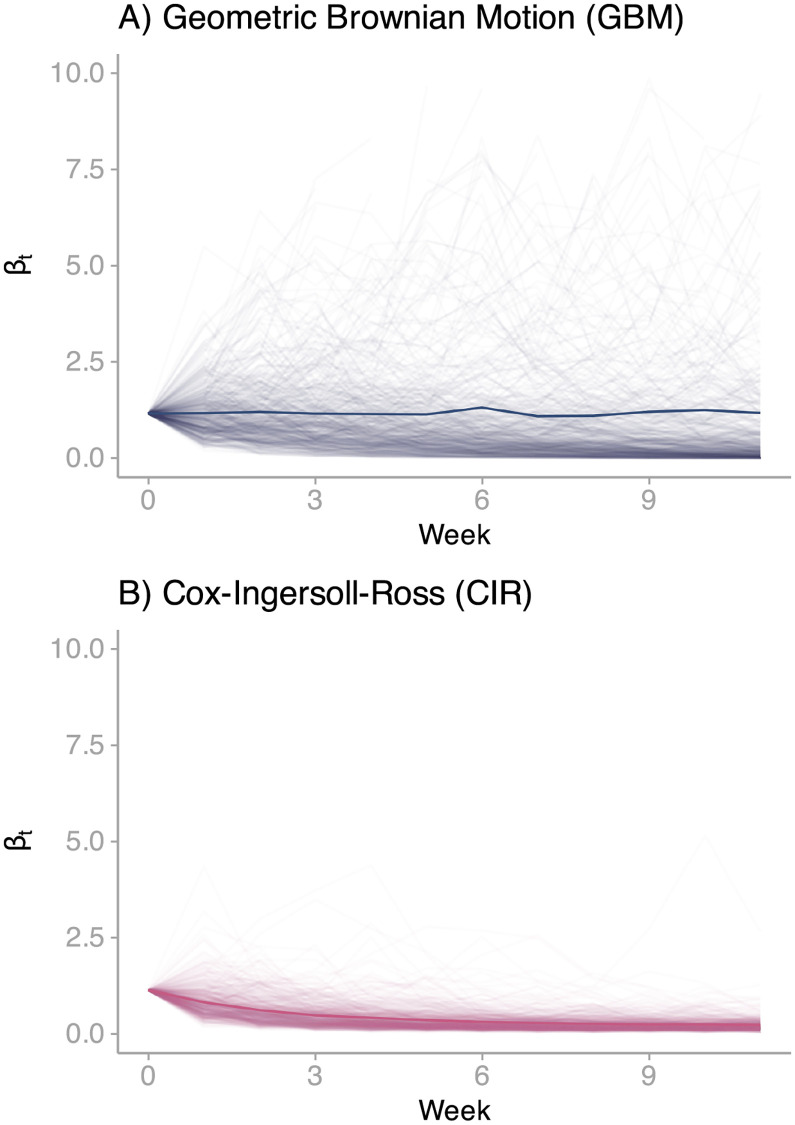
Brownian motion trajectories. (A) 200 simulations from a transmission rate described in terms of Geometric Brownian Motion. We generate these simulations from DGP1’s Maximum Likelihood Estimate (MLE) using the Euler-Maruyama algorithm. The highlighted trend corresponds to the mean trajectory path. (B) 200 simulations from a transmission rate described in terms of the Cox-Ingersoll-Ross model. We generate these simulations from DGP2’s Maximum Likelihood Estimate (MLE) using the Euler-Maruyama algorithm. The highlighted trend corresponds to the mean trajectory path.

Thus far, we have not yet defined the time-varying effective contact rate or transmission rate (*β*_*t*_). When defined, this component is integrated with the SEI3R profile to form a process model (Fig 1). For this and the other two DGPs, we formulate *β*_*t*_ as the product of two components (Eq 3). Here, *ζ* denotes the transmission rate’s initial value. Namely, *β*_0_ = *ζ*. From this definition, it follows that *Z*_*t*_ represents the transmissions rate’s change over time relative to its initial value, where *Z*_0_ = 1. In relation to *Z*_*t*_ dynamics, we initially opt for a flexible approach to build this first process model (PM1). Specifically, we define $\frac{dZ}{dt}$ in terms of Geometric Brownian Motion (GBM) with no *drift* (Eqs 4 and 5), an approach adopted in previous studies of influenza and Ebola [22–25]. This stochastic structure is a model for the change in a random process, *dZ*_*t*_, in relation to the current value, *Z*_*t*_, where the proportional change $\frac{dZ}{Z_{t}}$ follows Brownian motion [37]. That is, normal distributed random *jumps* (*dW*) moderated by a volatility parameter (*α*). We do not imply that the actual transmission rate follows a random walk. In fact, the expected value of *Z*_*t*_ is constant over time (*Z*_0_); strictly speaking, a *martingale* [37]. In practice, however, we use this structure as a *scaffold* to obtain some idea of the non-linear structure of the process without committing to a particular form of non-linear model [38]. This procedure resembles the use of smoothing splines to estimate coefficients that are allowed to vary as smooth functions of other variables [39]. Although not a requirement for this work, smoothing splines also have a Bayesian interpretation under certain conditions [40]. In particular, we use the GBM structure to generate non-negative random walks from an initial value (Fig 3A displays a set of possible trajectories). The main benefit from random walks is that at each time *t*, we propose several possible paths that the transmission rate may take and then use the available data to determine their plausibility [22]. In doing so, we *unravel* the dynamics of the effective contact rate. Formally put, we approximate *p*(*x*_*t*_|*y*_0:*t*_), the *filtering distribution* [30] (see Material and methods).

As noted above, the measurement model is the link between the process model and the data whereby one quantifies (through likelihood densities) the relative consistency of each set of parameter values, or model configuration, with observations. This quantification allows us to perform inference on time-varying and time-independent parameters. Thus, any misspecification in the measurement formulation can lead to overly confident conclusions [12] or biased estimations. In light of its importance, we prevent the consequences of model misspecification by proposing and testing six candidates that account for the incidence data (*y*^1^). Moreover, a subset of these candidates incorporate mobility data (*y*^2^), assuming that this dataset is a proxy observation of the relative contact rate (*Z*_*t*_).

Before defining each candidate, we clarify certain assumptions regarding the available datasets. On the one hand, for the incidence data (*y*^1^), we posit that *actual* periodic (daily or weekly) symptomatic COVID-19 cases (*C*_*t*_) stem from the transition *P*_*t*_ → *I*_*t*_. Our assumption implies that individuals seek the healthcare system for a diagnostic test as soon as they develop symptoms. Furthermore, under this formulation, it is implicit that *underreporting* is due to the non-identification of asymptomatic cases. On the other hand, for mobility data (*y*^2^), we emphasise its proxy nature. Contrary to the incidence time series, it is not anticipated that models yield faithful replications of Apple’s mobility indexes. Should that be the case, we would have included this data (directly or parametrically) in the process model. However, we refrain from doing so as we deem there may be instances where the two elements are not strongly correlated. To illustrate this point, we consider the case in which the government relaxes social distancing mandates and individuals adopt a mask-wearing behaviour. Under these circumstances, the resulting increase in mobility and social contacts due to relaxed rules do not necessarily entail an equivalent effect on the effective contact rate given that individuals properly wear face coverings during their interactions. Hence, rigid structures in the process model may lead to unrealistic corrections in other parameters. As opposed to such inflexibility, we expect that the mobility data acts as a *nudge* on the transmission rate, guiding the latter towards the former only when plausible. In light of these considerations, for candidates *1* and *2*, we formulate the observation of daily symptomatic COVID-19 cases ($y_{d}^{1}$) as independent Poisson and Negative Binomial counts, respectively. Then, we add an observational mechanism that relates Apple’s daily driving data ($y_{d}^{2}$) to the transmission rate’s relative level (*Z*_*t*_), yielding candidates *3* and *4*. Finally, even though King and colleagues [41] recommend that “*models should be fit to raw, disaggregated data whenever possible and never to temporally accumulated data*”, on candidates *1* and *3*, we modify their periodicity from daily to weekly measurements, resulting in candidates *5* and *6*. It should be noted that the use of weekly measurements has been performed previously in similar studies [22–25]. We refer the reader to S1 Text for the complete set of equations.

Having defined process and measurement structures, we proceed to the inference stage (Table 1 summarises the results). Since non-linear SSM do not allow closed-form solutions [30] to calculate likelihood values, we must resort to simulation-based approaches such as *Sequential Monte Carlo*, also known as the *Particle Filter*. Naturally, these estimates must be robust so as to guide the inference process. By robustness, we refer to the quality that the Particle Filter returns similar likelihood values for various runs from a single model configuration. Furthermore, as with any Monte Carlo approach, it is expected that as the number of samples tends to infinity, the likelihood error (among various runs) converges to zero. To test this feature, we run the Particle Filter using the R package *pomp*, which implements the Sequence Importance Sampling algorithm [42]. In particular, through these runs, we evaluate likelihood estimates for each model candidate by varying the number of particles (samples), the integration step size, and the model configuration (see the complete analysis in S1 Text). The results indicate that measurement models that account for daily incidence observations as Poisson counts lead to unstable estimates. This finding suggests model misspecification in candidates *1* and *3*, which are discarded from the pool.

**Table 1:** Measurement model candidates.

Id	Frequency	Incidence	Mobility	Converges	Fits incidence
1	Daily	Pois	No	No	N/A
2	Daily	Nbin	No	Yes	Yes
3	Daily	Pois	Yes	No	N/A
4	Daily	Nbin	Yes	Yes	No
5	Weekly	Pois	No	Yes	Yes
**6**	**Weekly**	**Pois**	**Yes**	**Yes**	**Yes**

To the remaining candidates, we estimate their latent states. Given its strength to infer time-varying random variables in the state space, the Particle Filter is also appropriate to numerically approximate (via samples) filtering distributions [33]. Nevertheless, drawing relevant samples requires plausible fixed-parameter values. Here, we assume that three parameters in PM1 are unknown: the effective contact rate at time 0 (*ζ*), the initial value of preclinical individuals (*P*_0_), and the volatility parameter (*α*). Moreover, additional parameters may be required depending upon the specific measurement model. To infer such parameters, we employ the *Iterated Filtering* algorithm [31, 43]. This Maximum Likelihood estimation method has been designed to perform statistical inference on SSM and has been widely used to study infectious disease models [16, 17, 31, 41, 44]. Briefly, Maximum likelihood via Iterated Filtering (MIF) is a modified version of the Particle Filter, in which a sequence of filtering operations converges to the Maximum Likelihood Estimate (MLE). The key feature in this procedure is the set of stochastic perturbations applied to the unknown parameters in between the sequence of filtering operations, resulting in the selection of plausible parameter values in the light of the available data. Furthermore, the synergy between MIF and the Particle Filter permits us to calculate uncertainty bounds around the MLE. In particular, we use the *Profile Likelihood* method [45] and its refined version, the *Monte Carlo-adjusted profile* [46]. Ultimately, all of this information facilitates the construction of the parameters’ likelihood surface.

For each model, we leverage its likelihood surface to draw sets of point estimates from the neighbourhood surrounding the MLE [41]. These draws are subsequently plugged into the Particle Filter. In addition to likelihood estimates, *pomp* returns, for every run, a set of samples representing the filtering distribution at each time *t*. Then, we assign a weight to each run based on its relative likelihood. In doing so, we account for parameter uncertainty in the results. Finally, we summarise the results by computing weighted averages on the samples. This procedure allows us to calculate the uncertainty in the predicted latent states by the filtering distribution. The reader can find the complete set of results in S2–S5 Text.

The inference process on Candidate *2* (see S2 Text) reveals that this model yields a filtering distribution that fits the observed daily incidence. Interestingly, although Candidate *2*’s measurement model does not incorporate mobility data in its structure, the predicted relative contact rate captures the observed mobility indexes, albeit with a large degree of uncertainty. This finding supports the argument that such a dataset could be an adequate proxy for the relative contact rate. Then, one would logically expect that incorporating Apple’s data into the measurement model (as we did for Candidate *4*) would diminish the resulting uncertainty in the filtering distribution. However, the results (see S3 Text) show that the enhanced fit on the effective contact rate stems from unrealistic corrections to the predicted incidence, rendering Candidate *4* unreliable. On the other hand, we notice that Candidate *5*’s filtering distribution and parameter estimates convey similar insights to those of Candidate *2* (see S8 Text Section 1). Therefore, the change in periodicity does not result in severe loss of information. Yet more important, the crucial feature of the weekly formulation is that it allows integrating mobility data seamlessly into the measurement model (Candidate *6*). This integration is accomplished without compromising the prediction on incidence counts and simultaneously reducing the uncertainty in the relative contact rate’s fit. This behaviour differs from the unrealistic fit achieved by Candidate *4*. We ascribe the resulting harmony between the two datasets to the stringency imposed by the Poisson distribution, which implicitly prioritises incidence counts over mobility indexes. In consequence, we select Candidate *6*’s measurement model (Eqs 6–8) as the structure (OM1) that completes DGP1’s formulation (Fig 1).

$$\begin{array}{c} \frac{dC}{dt}=\eta P_{t}-C_{t}\delta \left(t\;mod\;7\right) \end{array}$$ (6)

$$\begin{array}{c} y_{w}^{1}∼Pois\left(C_{t}\right) \end{array}$$ (7)

$$\begin{array}{c} y_{w}^{2}∼Normal\left(Z_{t},\tau \right) \end{array}$$ (8)

**Fig 4:**
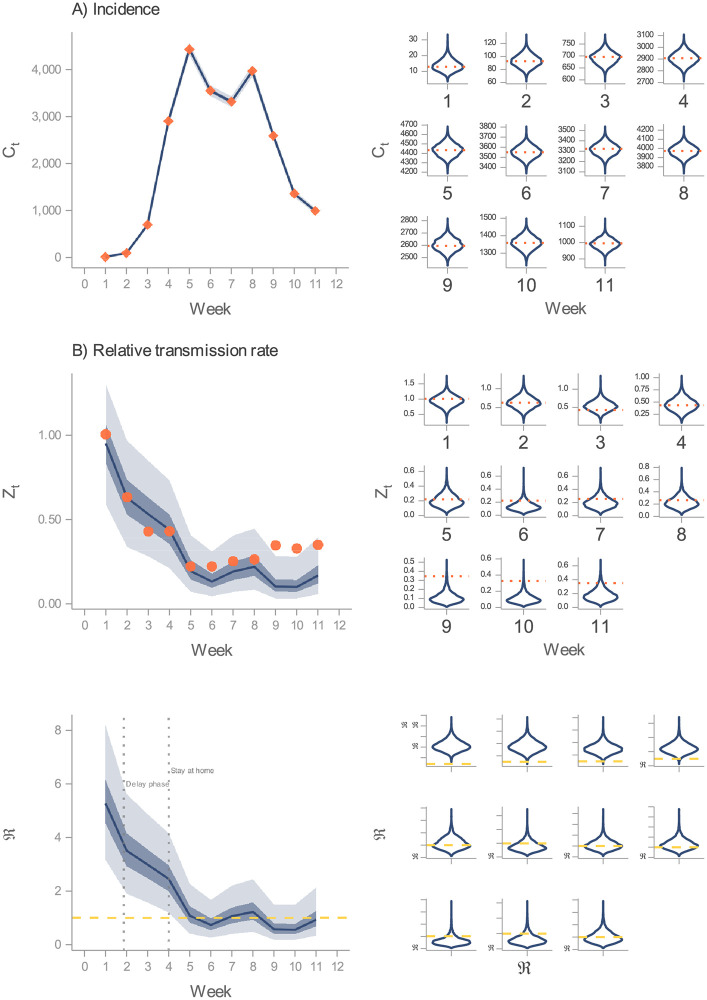
Inference on DGP1. In these three figures, the predicted values stem from DGP1’s filtering distribution. Further, in the LHS, the solid line indicates the median, and the darker and lighter ribbons represent the 50% and 95% CI, respectively. (A) Comparison between the predicted incidence (solid line and ribbons in the LHS; violin plots in the RHS) and weekly detected cases from Ireland’s first COVID-19 wave (rhombi in the LHS; horizontal dotted lines in the RHS). (B) Comparison between the predicted relative transmission rate (solid line and ribbons in the LHS; violin plots in the RHS) compared to Apple’s mobility indexes in Ireland (points in the LHS; horizontal dotted lines in the RHS). (C) Predicted effective reproduction number (solid line and ribbons in the LHS; violin plots in the RHS). Horizontal dashed lines denote the epidemics threshold.

Fig 4A presents a comparison between the predicted number of weekly symptomatic cases from DGP1 and observed incidence. Notice that this is a contrast between measurements ($y_{w}^{1}$) and a latent state (*C*_*t*_). Although this approach is not generally applicable (comparing measurements to predicted latent states), in this case it is valid given that *C*_*t*_ corresponds to the mean of the measurement model (Eq 7). In Fig 4A, it can be seen that this model’s filtering distribution captures the actual values in regions of high plausibility, thus yielding an accurate fit. This result helps us gain confidence in the model’s structure as an adequate dynamic hypothesis to the studied phenomenon, considering that it can reproduce the observed behaviour [13]. Similarly, the estimated relative effective contact rate replicates to a large extent its assumed measurement values (Fig 4B). As expected, the filtering distribution does not capture all of the measurements (Weeks 9–11), given the proxy nature of the data. However, these results allow us to elucidate the trajectory of the effective contact rate, and in turn, the effective reproductive number (see Material and methods for the estimation of this quantity). It must be remarked that in the early stages of this outbreak, the dynamics of the transmission rate determined the level of ℜ_*t*_. This characteristic occurs when the susceptible fraction is close to one, as was the case during the first wave [47]. In Fig 4C, we present the estimated ℜ_*t*_, where it can be observed that the behaviour change (presumably caused by mobility restrictions and people’s awareness) led to an ℜ_*t*_ close to or below the epidemics threshold, bringing about a lowering of the incidence rate.

### DPG2—Cox-Ingersoll-Ross


$$\begin{array}{c} \frac{dZ}{dt}=\nu \left(\upsilon -Z_{t}\right)+\sqrt{\alpha }Z_{t}dW \end{array}$$ (9)

The dynamics of the transmission rate (Fig 4B) uncovered by DGP1 exhibit a compelling pattern. The transmission rate gradually decays for several weeks from its initial value until it levels off around a determined value. In other words, a pattern that resembles *goal-seeking behaviour* [48]. Based on this recognition, we formulate the relative transmission rate in terms of the Cox-Ingersoll-Ross (CIR) model [49]. This formulation (Eq 9) is a compromise between the rigidity of a deterministic structure and the flexibility offered by random walks. Under this structure, the randomly-moving quantity of interest (*Z*_*t*_) is elastically pulled toward a central location or long-term *goal*, *υ*. The strictly positive parameter *ν* determines the speed of adjustment. In practice, we can interpret the long-term goal as the minimum level of mobility that the restrictions can achieve and the adjustment parameter as the rate at which individuals adopt such mandates. Hence, inferring these parameters permit the characterisation of the implemented interventions, a piece of information that cannot be estimated from DGP1. The randomness in this process stems from the diffusion process (second term). That is, stochastic variations from the deterministic trend. More importantly, unlike those in the *Vasicek* and *Ornstein-Uhlenbeck* structures, this particular diffusion process precludes negative values [37], a *sine qua non* to describe transmission rates. Logically, we ensemble this structure with the SEI3R profile to build the process model (*PM2*). As with DGP1, we assess the convergence of likelihood estimates obtained from the amalgamation of PM2 and the previously defined six measurement model candidates (see S1 Text Section 3). The results reveal an identical pattern to that observed in DGP1. Therefore, it is warranted to integrate PM2 and OM1 (Fig 1) to form a DGP that we refer to as *DGP2*. In Fig 3B, we present simulated trajectories from this DGP, obtained from a single set of parameters (MLE).

The main objective for building DGP2 is to estimate its latent states conditional on the available data. To do so, we repeat the process applied to DGP1. Specifically, we first perform parameter inference and construct DGP2’s likelihood surface using MIF and the Particle Filter. The next step consists of drawing samples from the MLE’s neighbourhood to plug them into the Particle Filter. There is a slight alteration in this process, however. Previously, we selected parameter combinations that yielded likelihood values near the MLE to construct DGP1’s neighbourhood. We then identified the bounds of these parameters to construct a four-dimensional hypercube. From this object, we obtained independent and uniformly distributed samples for each parameter. In light of DGP2’s complex parameter space, we opt for a copula [50] instead of a hypercube. The copula is a multivariate cumulative distribution for which the marginal probability distribution of each variable is uniform, but there is dependence (correlation) among the random variables (unknown parameters). In doing so, we mitigate biases caused by point estimates that yield abnormal likelihood values. The reader can find the complete set of results in S6 Text.

**Fig 5:**
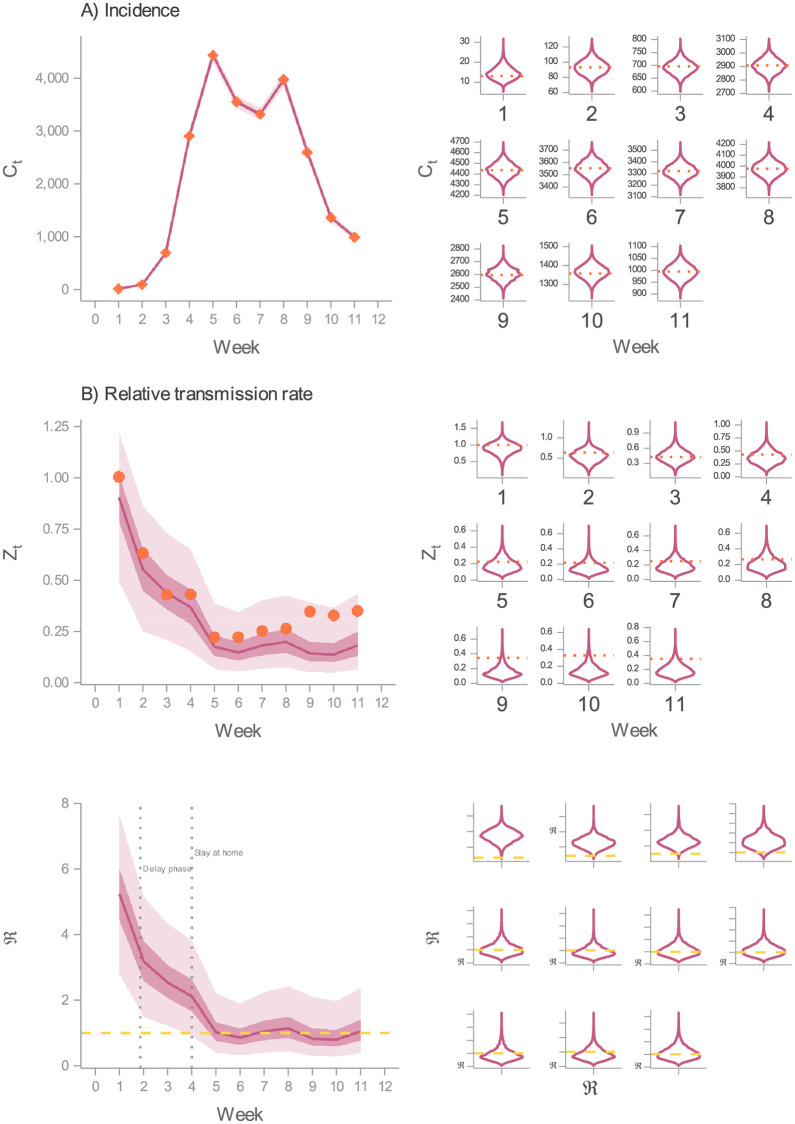
Inference on DGP2. In these three figures, the predicted values stem from DGP2’s filtering distribution. Further, in the LHS, the solid line indicates the median, and the darker and lighter ribbons represent the 50% and 95% CI, respectively. (A) Comparison between the predicted incidence (solid line and ribbons in the LHS; violin plots in the RHS) and weekly detected cases from Ireland’s first COVID-19 wave (rhombi in the LHS; horizontal dotted lines in the RHS). (B) Comparison between the predicted relative transmission rate (solid line and ribbons in the LHS; violin plots in the RHS) compared to Apple’s mobility indexes in Ireland (points in the LHS; horizontal dotted lines in the RHS). (C) Predicted effective reproduction number (solid line and ribbons in the LHS; violin plots in the RHS). Horizontal dashed lines denote the epidemics threshold.

Fig 5 displays the results obtained from the inference process carried out on DGP2. Qualitatively, the uncovered values match those obtained from DGP1. Namely, DGP2 produces an accurate fit of the incidence data (Fig 5A), and the inferred relative contact rate captures most of the mobility data (Fig 5B), resulting in a similar prediction of the effective reproduction number (Fig 5C). This outcome provides reassurance on the estimated transmission rate as an adequate account of the observed time series. That is, from two DGP that differ in the transmission rate’s formulation, we estimate equivalent trajectories. DGP2, though, does not reduce significantly the uncertainty (see S6 Text Section 2.3.7) in the parameters (*ν* and *υ*) that characterise the implemented NPIs.

### DGP3—Adaptive expectations


$$\begin{array}{c} \beta_{t}=\zeta Z_{t}^{1} \end{array}$$ (10)

$$\begin{array}{c} \frac{dZ^{i}}{dt}=\{\begin{array}{c} \frac{\left(\upsilon -Z_{t}^{i}\right)}{\left(\nu^{-1}/n\right)}\;\text{for}\;i=n\\ \frac{\left(Z_{t}^{i+1}-Z_{t}^{i}\right)}{\left(\nu^{-1}/n\right)}\;\text{for}\;i<n \end{array} \end{array}$$ (11)

The trajectories derived from the two previous DGPs (DGP1 and DGP2) suggest that it is reasonable to assume that the transmission rate’s dynamics indeed follow a goal-seeking pattern (Eq 10). This conjecture is in agreement with the economic theory of *adaptive expectations*. First applied by Irving Fisher [51], this hypothesis posits that individuals gradually adjust their beliefs, and hence behaviour, in order to eliminate the discrepancy between the current state and a *desired* one [52]. In this case, such a discrepancy is the gap between individuals’ behaviour at a given time *t* and the level of mobility that the restrictions (implicitly) aim to achieve. Mathematically, the nth-order information delay or *exponential smoothing* (Eq 11) provides a formal description of such an adjustment. This deterministic formulation describes the changes in current behaviour ($Z_{t}^{1}$) as the result of a series of intermediate exponential adjustments ($\frac{dZ^{i}}{dt}$), which one can interpret as the multiple stages intervening since the Government decrees mobility restrictions to the point where individuals alter their behaviour in accordance with the new rules. The delay order (*n*) represents the number of stages, where the most simple case (*n* = 1), the 1st-order information delay, is equivalent to the deterministic term in Eq 9. On the other hand, when *n* → ∞, the dynamics follow a *term-time forcing* pattern.

To establish the exact number of stages, we evaluate the performance of nine candidate structures (*n* = 1, …, 9) in explaining the available data (incidence and mobility). From this evaluation, we ensemble the selected candidate with the SEI3R profile to generate the process model (PM3) of the third DPG (DPG3) presented in this paper (Fig 1). To complete DGP3’s description, we formulate a measurement model (OM2) for the observed daily reported cases ($y_{d}^{1}$). As with DGP1 and DGP2, we assume these counts result from a Poisson distribution (Eqs 12 and 13). Moreover, OM2 does not include a structure relating mobility data to the relative transmission rate. We base this decision on the results shown in the previous sections. Since the mobility data is an imperfect predictor of the transmission rate, its inclusion in the inference process of a rigid deterministic structure may lead to *forced* model fits, resulting in undesired biases in parameter estimations. In relation to the inference process, since PM3 is deterministic, the inference of the filtering distribution becomes the estimation of DGP3’s expected value. We approximate such expected value from a Bayesian perspective [53, 54] using Hamiltonian Monte Carlo [55] via Stan. The complete set of results can be found in S7 Text.

$$\begin{array}{c} \frac{dC}{dt}=\eta P_{t}-C_{t}\delta \left(t\;mod\;1\right) \end{array}$$ (12)

$$\begin{array}{c} y_{1}^{d}∼Pois\left(C_{t}\right) \end{array}$$ (13)

**Fig 6:**
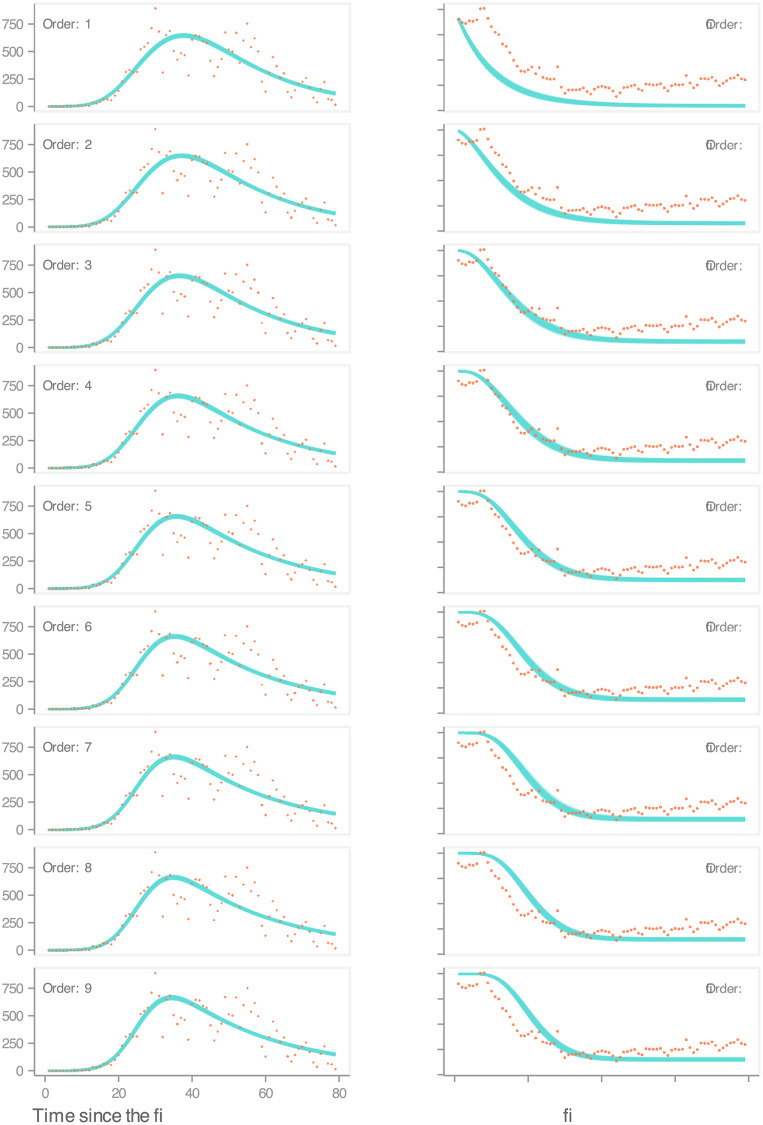
Inference on DGP3. Comparison between expected values and data. On the LHS, for each model, we show 100 overlapped simulations of the predicted incidence against daily case counts. On the RHS, for each model, we show 100 overlapped simulations of the predicted relative transmission rate against Apple’s mobility data. In this plot, we estimate the predicted values from the posterior distribution of each of the DGP3’s nine candidate process models.

To illustrate the selection of DGP3’s process model, we present the estimated expected values (fits) for each of the nine candidate structures (Fig 6). We depict expected values through simulated trajectories generated from one hundred draws from each model’s posterior distribution. The results indicate that all of these structures yield similar fits to the incidence data. Using the mean absolute scaled error (MASE), a metric designed to measure the accuracy of time-series predictions [56], we notice diminishing marginal gains in accuracy as the order (of the number of stages) increases. These gains, though, are so tenuous that they do not provide clear guidance about which model to choose. To further complicate matters, the lower the delay order, the higher the likelihood value. Nevertheless, when we compare the expected relative transmission rate to mobility data, it can be seen that some structures approximate better the latter than others. If we accept the premise that mobility data is a proxy (supported by the results from DGP1 and DGP2), yet imperfect, measurement of the relative transmission rate, we can then lean towards the delay order that yields the lowest MASE (*n* = 4). From this structure’s posterior distribution, we estimate, among others, the adjustment rate (*ν*; mean = 0.05, sd = 0.001), the minimum level of mobility (*υ*; mean = 0.11, sd = 0.005), and the effective reproduction number (discussed below). Notice that the particular form of the non-linear contact rate restricts the marginal distributions of *ν* and *υ* to such an extent that most of the probability mass concentrates on extremely narrow neighbourhoods. Despite this, those estimates resemble DPG2’s MLE (*ν* = 0.05, *υ* = 0.19), which help us gain confidence in the overall process.

Acknowledging that the performance metrics above (MASE and likelihood values) do not lead to an unambiguous choice, we explore the implications of selecting an alternative measurement model. As it is widely known, the Poisson distribution is a discrete probability distribution in which the observation mean equals the variance [32]. Hence, using this distribution as a measurement model imposes a stringent assumption on the observational process of incidence counts. By contrast, the Negative Binomial distribution offers a more flexible framework to account for overdispersion in daily incidence. Moreover, the Negative Binomial converges to the Poisson distribution under a specific configuration. For this reason, we test the implications of this alternative formulation. See the complete set of results in S7 Text Section 4. Indeed, the posterior distribution suggests the presence of a small amount of overdispersion in the incidence data. However, such gain in realism is achieved at the expense of a *degenerate* posterior distribution. Succinctly, any of the model candidates coupled with the Negative binomial distribution yields a posterior distribution of two distinct modes, even from a single unknown parameter. This kind of behaviour is not unusual in Ordinary Differential Equation models. For instance, Gelman and colleagues [57] report a similar experience in the calibration of a simple mechanistic model of planetary motion.

In the set of bimodal distributions returned by Stan, we recognise two types of modes. One that corresponds to a region of unrealistic parameter values for which the HMC algorithm reveals pathological behaviour (divergences and low E-BFMI) [55] in the sampling procedure, rendering the inference from these samples unreliable. Conversely, the Markov chains that land in the other type of mode do not trigger any warnings from Stan. Furthermore, these *well-behaved* modes are located in regions similar to those found using the Poisson distribution. Following an exploratory analysis, we find that *well-behaved* modes and the set of posterior distributions obtained from the Poisson model provide similar (although not identical) information. Overall, the choice of the Poisson distribution and the delay order (4th) is the outcome of considering as a whole the information provided by the previous DGPs, and the two explored measurement models. This assessment, therefore, implies that we envision the Poisson measurement model as an approximation that does not compromise the insights from the inference process. However, one cannot generalise this result to other applications. That is, taking the Poisson distribution as a default. On the contrary, it is imperative to test the assumptions embedded in any proposed measurement model and evaluate the trade-offs entailed by each alternative.

## Discussion

Novel datasets that may assist modellers in gaining deeper insight into the dynamics of an infectious disease deserve a thorough examination. This task entails establishing adequate links between the data and a dynamical hypothesis. Far from trivial, one may derail the entire inference process by adopting a misspecified structure. For this reason, a robust approach involves the assessment of various levels of model complexity that account for the available data, which inevitably involves trade-offs [58]. This work highlighted the implications of committing to a particular model formulation. As seen above, DGP1 and DGP2 (DGPs with a stochastic process model) can only incorporate the mobility data if they are formulated in terms of weekly observations. Notwithstanding that this requirement reduces the number of data points available for the inference process, the loss of information is negligible. In contrast, a rigid structure such as DGP3 (whose process model is deterministic) restricts the use of mobility data only as a discriminant criterion.

With regards to the inference of fixed parameters, DGP1’s *well-behaved* parameter space yields smooth quadratic profiles from which parameter uncertainty can be seamlessly calculated. Interestingly, when we amalgamate all the likelihood estimates, we obtain surfaces that resemble likelihood profiles. As a result, from three approaches (MCAP, profile, surface), we estimate similar confidence intervals. DGP2’s parameter space is, on the other hand, of challenging exploration. In fact, the volume of high plausibility is so tightly concentrated that some regions in the MLE’s neighbourhood yield vast negative log-likelihood values. To address this issue, we iterated over several hypercube sizes and densities until obtaining quadratic profiles, although not as smooth as those obtained from DGP1. Despite this hurdle, we obtain similar confidence intervals from the three quantification approaches. Regarding DGP3, given the Bayesian approach used to estimate its parameters, we refer to such uncertainty bounds as credible intervals. We obtain well-behaved quadratic posterior distributions for the nine candidate process models whose inference is backed by successful diagnostics unique to HMC. However, parameter estimates (posteriors) vary by the delay order, requiring a subjective assessment to determine which structure is more appropriate. Lastly, we consider the differences in computational burden between the inference methods (MIF + Particle Filter and HMC). Whereas performing parameter inference on DGP1 and DGP2 took roughly 14 and 20 hours, respectively; fitting DGP3’s nine candidate models required 6 hours of computational time.

**Fig 7:**
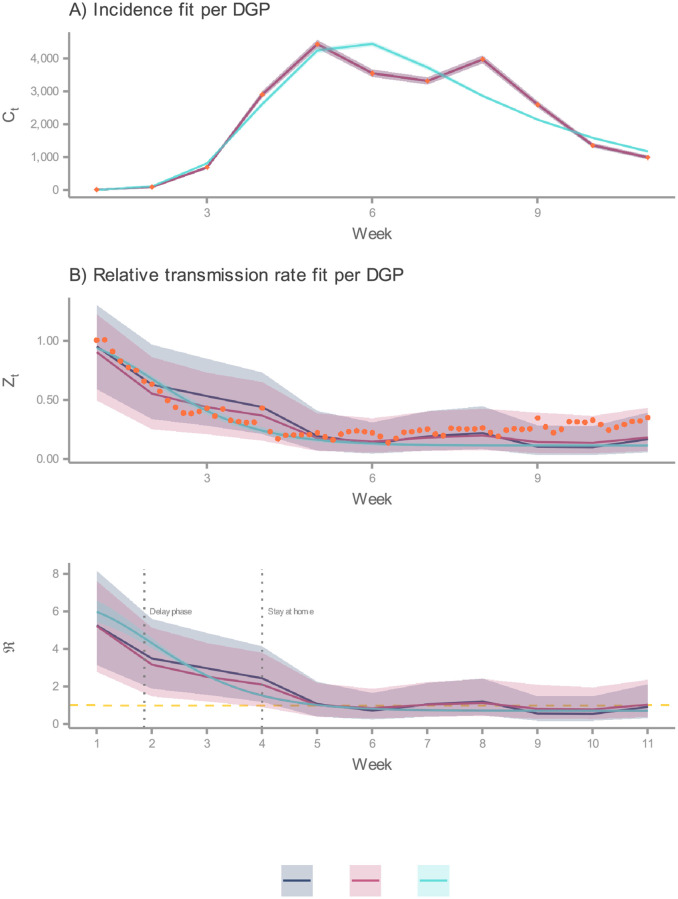
Comparison of predicted latent states. In this plot, predicted values stem either from the filtering distribution (DGP1 and DGP2) or the posterior predictive distribution (DGP3). Here, DGP3’s process model corresponds to the structure that describes the transmission rate in terms of a 4th-order information delay. Further, solid lines indicates the median, and the ribbons represent the 95% CI. (A) Comparison between predicted incidences by DGP (solid lines and ribbons) and weekly detected COVID-19 cases (rhombi) in Ireland during the first wave. (B) Comparison between predicted relative transmission rates by DGP (solid lines and ribbons) and Apple’s driving mobility indexes (points) in Ireland during the first wave. (C) Predicted effective reproductive numbers by DGP (solid lines and ribbons) during Ireland’s first COVID-19 wave. The dashed horizontal line denotes the epidemics threshold.

Likewise, the inference of the time-varying quantities deserves close inspection. DGP1 and DGP2 are more flexible than DGP3 in quantifying uncertainty. We illustrate this point with Fig 7B and 7C. Here, we notice that DGP3 generates an estimate of the relative transmission rate and the effective reproduction number with narrow uncertainty intervals in comparison with those generated by the other DGPs. This apparent precision is the result of committing to a particular form of non-linear model, which imposes a stringent constraint in the shape of the transmission rate. By choosing the 4th-order information delay structure, we implicitly discard the possibility for the other formulations to be true, reducing the uncertainty in the estimations. However, we demonstrated that the nine delay orders account similarly for the incidence data, and to various degrees of accuracy, for the mobility data. Thus, we interpret the wide intervals generated by DGP1 and DGP2 as the uncertainty in the delay order plus the measurement error. This interpretation suggests that DGP3’s plausible model candidates are subsumed under DGP1 and DGP2.

To conclude with this comparative analysis, we reflect on the role of DGPs presented in this paper. Owing to its flexible formulation, we can employ DGP1 for both retrospective and near real-time analysis (at least for the period where demographic processes do not significantly impact the dynamics of the pandemic). In contrast, DGP2 and DGP3 formulations are context-dependent, restricted to retrospective analyses. Under this last role, we note that common patterns emerge from the three DGPs. Notwithstanding structural differences, all of them produce accurate fits to the incidence data (Fig 7A). Naturally, the stochastic process models replicate every feature in the data, whereas the deterministic one captures the underlying trend. Furthermore, the estimated medians for the relative transmission and the effective reproduction number (Fig 7B and 7C) tell similar stories. That is, individuals gradually decreased their movements following public health advice, which led to a decline in the transmission rate. This reduction pulled ℜ_*t*_ below the epidemics threshold, causing the incidence rate to subside. It should be noted that this mobility reduction levels off later in Ireland’s first wave, suggesting a limit on the effectiveness of the implemented policies. We interpret this limit as the minimum mobility required for running essential services.

Finally, even though the primary interest of this work has been on estimating the effective reproduction number (ℜ_*t*_), a by-product from this inference process is the approximation of the basic reproductive number (ℜ_0_). This widely accepted metric [59] is defined as the average number of secondary infections produced when one infected individual is introduced into a totally susceptible population [3]. In the context of Ireland’s COVID-19 epidemic, we derive similar ℜ_0_ estimates from the three DGPs (DGP1: 95% CI[4.5—6.9], DGP2: 95% CI[4.4—6.8], DGP3: 95% CI[5.8—7.0]). These estimates are in close agreement with a previous modelling study on the COVID-19 pandemic in Ireland [35], albeit well above the initially reported ℜ_0_ = 2.2 value from Wuhan [60]; a value that has been adopted as the reference point by the World Health Organization and other research groups [15, 61]. Other streams of research, however, argue that the initial estimate was low [62], and instead, advocate for higher values (4.5 [62]; 4.7—6.6 [63]). Moreover, the reader should recall that ℜ_0_ is a context-dependent metric, and variations are expected due to population heterogeneity (e.g., age, spatial location, host genetics). In any case, we acknowledge the limitations that stem from the calibration of homogeneous population models, which require high ℜ_0_ values to achieve accurate fits [17]. To address such limitations, future research should test the impact of disaggregating (by age or location) the structures presented in this paper. Another research avenue could explore the effect of replacing the deterministic rates in the within-host profile of these DGPs with stochastic ones that account for demographic and environmental effects.

## Materials and methods

### SEI3R profile

This within-host profile (Eqs 14–20) is formulated based on the work from Davies and colleagues [15]. Here, we assume that individuals are initially susceptible (*S*) and become exposed (*E*), at a rate λ, after effective contact with an infectious person (*I*, *P*, *A*). After a latent period (*σ*^−1^), exposed individuals follow one of two paths. With probability *ω*, following a period (*η*^−1^) of preclinical infectiousness (*P*), individuals develop full symptoms while transmitting the pathogen. This stage is known as the clinical infection state and lasts for *γ*^−1^ days. On the second path, with probability 1−*ω*, individuals enter a subclinical state (*A*) with none (asymptomatic) or mild symptoms (paucisymptomatic), who are not captured by the healthcare system. Individuals on this path recover after *κ*^−1^ days and are relatively (*μ*) less infectious than their counterparts on the clinical path. Finally, individuals from both paths eventually converge to the recovered state (*R*), in which they are no longer infectious and are immune to re-infection. In S2–S6 Text, we provide the values for fixed parameters and initial states and their respective sources.

$$\begin{array}{c} \frac{dS}{dt}=-S_{t}\lambda_{t} \end{array}$$ (14)

$$\begin{array}{c} \frac{dE}{dt}=S_{t}\lambda_{t}-\sigma E_{t} \end{array}$$ (15)

$$\begin{array}{c} \frac{dP}{dt}=\omega \sigma E_{t}-\eta P_{t} \end{array}$$ (16)

$$\begin{array}{c} \frac{dI}{dt}=\eta P_{t}-\gamma I_{t} \end{array}$$ (17)

$$\begin{array}{c} \frac{dA}{dt}=\left(1-\omega \right)\sigma E_{t}-\kappa A_{t} \end{array}$$ (18)

$$\begin{array}{c} \frac{dR}{dt}=\kappa A_{t}+\gamma I_{t} \end{array}$$ (19)

$$\begin{array}{c} \lambda_{t}=\frac{\beta_{t}\left(P_{t}+I_{t}+\mu A_{t}\right)}{N_{t}} \end{array}$$ (20)

### Basic and effective reproductive number

To derive an analytical expression for the basic reproduction number (ℜ_0_) from the SEI3R profile, we employ the next generation matrix method [64]. That is, we rewrite the infected states’ transitions (rates) in the form of two matrices. The first matrix F corresponds to the rate of appearance of new infections in each compartment of infected individuals, and the second matrix V corresponds to the rate of other transitions between compartments of infected individuals. From these matrices, we define the next generation matrix as $FV^{-1}$, whose largest eigenvalue (spectral radius) corresponds to ℜ_0_ [65]. We obtain the spectral radius’s analytical solution (Eq 21) using the software system *Mathematica* (see Github repository). Following this expression, we can define (Eq 22) the effective reproductive number (ℜ_*t*_) as the product between ℜ_0_ and the susceptible fraction ($\frac{S_{t}}{N_{t}}$).

$$\begin{array}{c} {ℜ}_{0}=\zeta Z_{0}\left[\omega \gamma +\eta +\left(1-\omega \right)\mu \kappa \right] \end{array}$$ (21)

$$\begin{array}{c} {ℜ}_{t}={ℜ}_{0}\frac{S_{t}}{N_{t}} \end{array}$$ (22)

### Filtering distribution


$$\begin{array}{c} p\left(x_{t}|y_{1:t-1}\right)=\int p\left(x_{t}|x_{t-1}\right)p\left(x_{t-1}|y_{1:t-1}\right)dx_{t-1} \end{array}$$ (23)

$$\begin{array}{c} p\left(x_{t}|y_{1:t}\right)=\frac{p\left(y_{t}|x_{t}\right)p\left(x_{t}|y_{1:t-1}\right)}{p(y_{t}|y_{1:t-1})} \end{array}$$ (24)

The essence of the state-space approach is to estimate the state of a dynamical system using a sequence of noisy measurements made on the system. We formulate this problem in terms of a recursive filter whose purpose is to construct the state’s posterior probability density function (pdf) based on all available information, including the set of received measurements [33]. Formally, *p*(*x*_*t*_|*y*_1:*t*_). We refer to this pdf as the *filtering distribution*, whose inference process consists of two stages: *prediction* and *update*.

The prediction stage (Eq 23) draws on the *plug-and-play* property [17] to generate, from simulations of the process model *p*(*x*_*t*_|*x*_*t*−1_), a vector of predictions that describe the state at time t (*x*_*t*_), which are conditional on the previously estimated state (*x*_*t*−1_|*y*_*t*−1_). Then, the update operation (Eq 24) uses the latest measurement to modify the prediction pdf (*p*(*x*_*t*_|*y*_1:*t*−1_)). In practice, we assign weights to the prediction vector based on its plausibility, which is estimated from the measurement model *p*(*y*_*t*_|*x*_*t*_). With these weights, we use the Sequence Importance Sampling algorithm [42] to produce samples that describe the filtering distribution. It is important to remark that this is a sequential process (hence the name Sequential Monte Carlo), executed every time a measurement is received. Moreover, in this simplified formulation, it is assumed that *X*_0_ and *θ* (Eqs 1 and 2) are known. We refer the interested reader to [30, 33] for a complete treatment of this approach.

## Supporting information

S1 Text Likelihood convergence. (PDF)

S2 Text Inference on DGP1’s Candidate 2. (PDF)

S3 Text Inference on DGP1’s Candidate 4. (PDF)

S4 Text Inference on DGP1’s Candidate 5. (PDF)

S5 Text Inference on DGP1’s Candidate 6. (PDF)

S6 Text Inference on DGP2. (PDF)

S7 Text Inference on DGP3. (PDF)

S8 Text Output comparison. (PDF)

S1 Data Incidence data. (XLSX)
