# Supplementary material for: Inferring the effective reproductive number from deterministic and semi-deterministic compartmental models using incidence and mobility data
Source: PLoS Comput Biol. 2022 Jun 27;18(6):e1010206. doi: 10.1371/journal.pcbi.1010206 (PMC9269962; doi:10.1371/journal.pcbi.1010206)
Supplement: S1 Text — (PDF) [file pcbi.1010206.s001.pdf]

S1 Appendix

A critical requirement for parameter inference on State-Space Models is the robustness of likelihood estimates. Namely, different runs ought to yield similar log-likelihood values from a single set of parameters (model configuration). Consequently, in this appendix, we formulate and assess the convergence of six measurement model candidates that account for the observed incidence. Some models account for the relative contact rate, assuming mobility data as a proxy observation. We couple those candidate measurements models with two process models. The first process model (PM1) describes the relative contact rate of the SEI3R within-host profile in terms of Geometric Brownian Motion. The other process model (PM2) employs the Cox-Ingersoll-Ross formulation to describe the relative contact rate. Specifically, we perform a sensitivity analysis on the accuracy of the likelihood estimates under various particle and integration step sizes for each chosen model configuration. Overall, the results allow us to identify possible model misspecifications and to select an appropriate integration step and sample size in a context of finite computational resources.

Contents

1 Data . . . **2**

2 Geometric Brownian Motion (DGP1) . . . **3**
- 2.1 Process model (PM1) . . . 3
- 2.2 Testing measurement model candidates . . . 3
  - 2.2.1 Testing points . . . 3
  - 2.2.2 Summary . . . 4
  - 2.2.3 Candidate 1 . . . 4
  - 2.2.4 Candidate 2 . . . 6
  - 2.2.5 Candidate 3 . . . 8
  - 2.2.6 Candidate 4 . . . 10
  - 2.2.7 Candidate 5 . . . 12
  - 2.2.8 Candidate 6 . . . 14

3 Cox-Ingersoll-Ross (DGP2) . . . **16**
- 3.1 Process model (PM2) . . . 16
- 3.2 Testing measurement model candidates . . . 16
  - 3.2.1 Testing points . . . 16
  - 3.2.2 Summary . . . 17
  - 3.2.3 Candidate 7 . . . 17
  - 3.2.4 Candidate 8 . . . 19
  - 3.2.5 Candidate 9 . . . 21
  - 3.2.6 Candidate 10 . . . 23
  - 3.2.7 Candidate 11 . . . 25
  - 3.2.8 Candidate 12 . . . 27

4 Original Computing Environment . . . **29**

1 Data

In this work, we base the inference process on two datasets. The first dataset corresponds to the number of COVID-19 cases detected during Ireland's first wave, from 29 February 2020 to 17 May 2020. We refer to this dataset as y^1 and distinguish between two aggregation levels: daily (y^1_d) and weekly (y^1_w). The second dataset (y^2) is Apple's driving mobility index which quantifies the number of requests made to Apple Maps for directions. These indexes are normalised to the value on 28 February 2020. Furthermore, given that data for May 11-12 is missing, we approximate such values using linear interpolation. In a similar manner to incidence data, we employ two subsets of datasets. Accordingly, y^2_d denotes daily measurements, and y^2_w denotes the measurement of the end of the week.

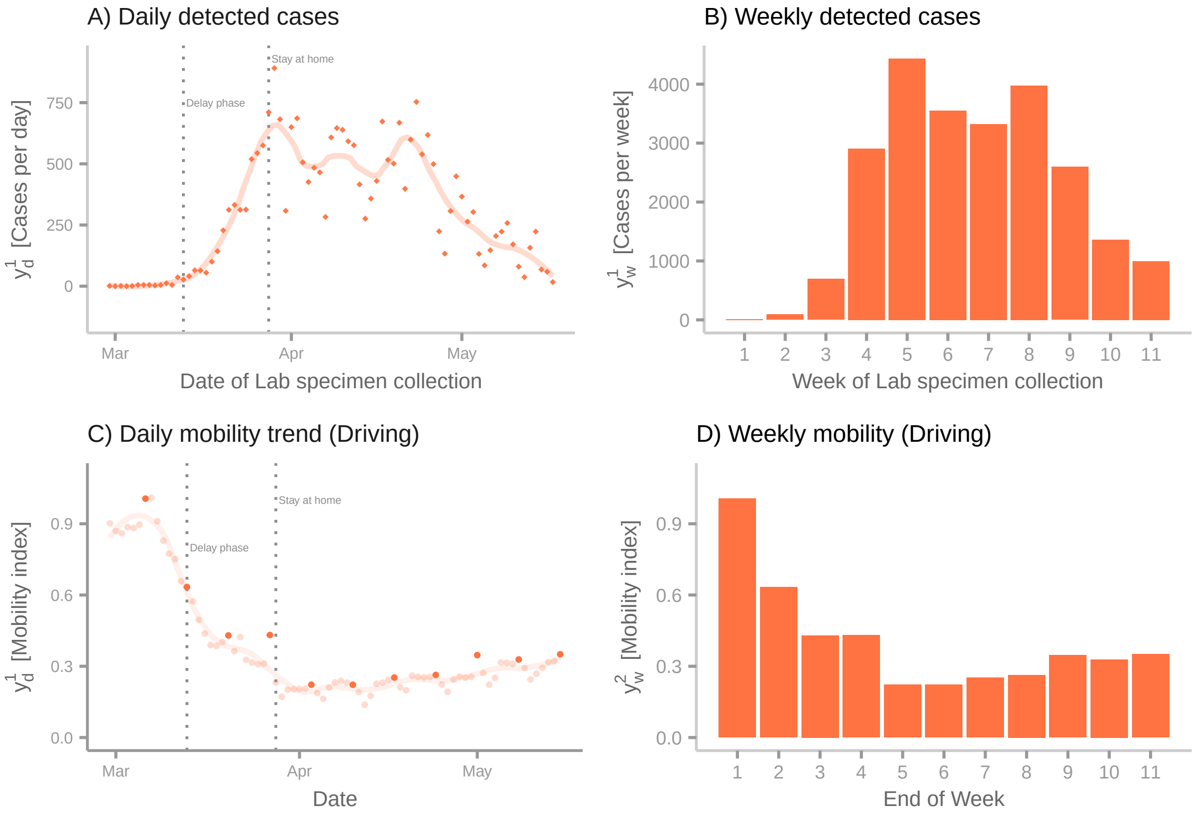

2 Geometric Brownian Motion (DGP1)

2.1 Process model (PM1)

$$\frac{dS}{dt} = -S_t\lambda_t \tag{1}$$

$$\frac{dE}{dt} = S_t\lambda_t - \sigma E_t \tag{2}$$

$$\frac{dP}{dt} = \omega\sigma E_t - \eta P_t \tag{3}$$

$$\frac{dI}{dt} = \eta P_t - \gamma I_t \tag{4}$$

$$\frac{dA}{dt} = (1-\omega)\sigma E_t - \kappa A_t \tag{5}$$

$$\frac{dR}{dt} = \kappa A_t + \gamma I_t \tag{6}$$

$$\lambda_t = \frac{\beta_t(I_t + P_t + \mu A_t)}{N_t} \tag{7}$$

$$\beta_t = \zeta Z_t \tag{8}$$

$$\frac{dZ}{dt} = \alpha Z_t dW \tag{9}$$

$$dW \sim Normal(0, \sqrt{dt}) \tag{10}$$

2.2 Testing measurement model candidates

2.2.1 Testing points

We use five point estimates (table below) as probes to assess the reliability of likelihood estimates. These point estimates correspond to *plausible* values obtained in a earlier version of this inference analysis. For the final version, we assume such values as **given**, and conduct the analysis from the start. Considering that these tests are not intended to be exhaustive but rather of exploratory nature, any arbitrary point is valid as a probe.

id	ζ	P_0	τ	α	ϕ
1	1.179	2.392	0.165	0.181	0.168
2	1.109	2.774	0.156	0.183	0.069
3	1.179	2.521	0.154	0.201	0.468
4	1.128	2.392	0.154	0.198	0.021
5	1.203	2.392	0.163	0.200	0.039

2.2.2 Summary

Specifically, we formulate six measurement model candidates. We describe these models using three features:

- Whether we fit the model to **daily** or **weekly** data.
- Whether we model incidence measurements using the **Poisson** or the **Negative binomial** distribution.
- Whether the model incorporates the mobility data as a proxy measurement for the relative effective contact rate. We assume these measurements are normally distributed.

For each probe (point estimate) and model candidate, we run the particle filter 16 times. We repeat the process for various particle and integration step sizes. For each batch of 16 runs, we estimate the log-likelihood mean, the log-likelihood standard error, and the computational time.

In a nutshell, the results indicate that when one models daily incidence measurements with the Poisson distribution, the particle filter does not converge as the number of particles increases. This finding suggests model misspecification under such an assumption.

Furthermore, the accuracy of log-likelihood estimates is not compromised by relative large integration steps (e.g., $\frac{1}{8}$), which diminishes the burden on computational resources. On the contrary, and as expected in Monte Carlo simulation, the accuracy relies on the number of samples (particles).

Id	Frequency	Incidence	Mobility	Converges
1	Daily	Pois	FALSE	No
2	Daily	Nbin	FALSE	Yes
3	Daily	Pois	TRUE	No
4	Daily	Nbin	TRUE	Yes
5	Weekly	Pois	FALSE	Yes
6	Weekly	Pois	TRUE	Yes

2.2.3 Candidate 1

This formulation assumes that daily incidence measurements are distributed according to the Poisson distribution. Also, this structure does not incorporate mobility data.

2.2.3.1 Equations

$$\frac{dC}{dt} = \eta P_t - C_t \delta(t \, mod \, 1) \tag{11}$$

$$y_d^1 \sim Pois(C_t) \tag{12}$$

2.2.3.2 Convergence test

This test shows that the likelihood standard error **does NOT** tend to zero as the number of particles increases. As a consequence, we cannot proceed with the next step in the inference process. That is, using the Iterated Filtering algorithm for parameter inference inasmuch as this plug-and-play method relies on robust likelihood estimates. Moreover, this result is also a signal of model misspecification.

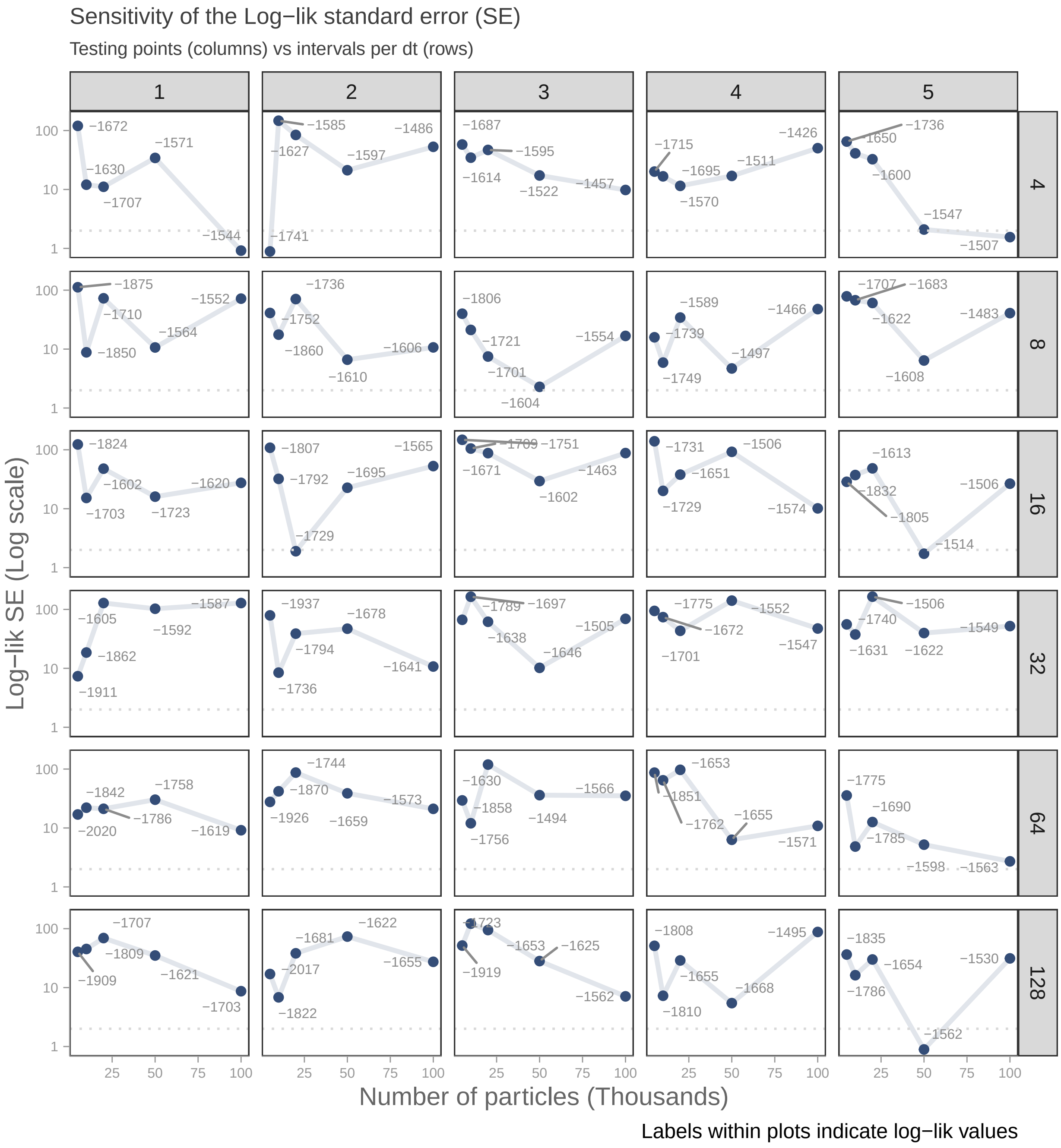

2.2.4 Candidate 2

This formulation assumes that daily incidence measurements are distributed according to the Negative binomial distribution. Also, this structure does not incorporate mobility data.

2.2.4.1 Equations

$$\frac{dC}{dt} = \eta P_t - C_t \delta(t \, mod \, 1) \tag{13}$$

$$y_d^1 \sim Nbin(C_t, \phi^{-1}) \tag{14}$$

2.2.4.2 Time comparison

As we will see below, these likelihood estimates converge. For this and other models that exhibit convergence, we present a plot comparing computational time elapsed in order to obtain likelihood estimates for testing point # 1. We do so for several scenarios. Here, scenarios refer to the number of particles and the integration step size.

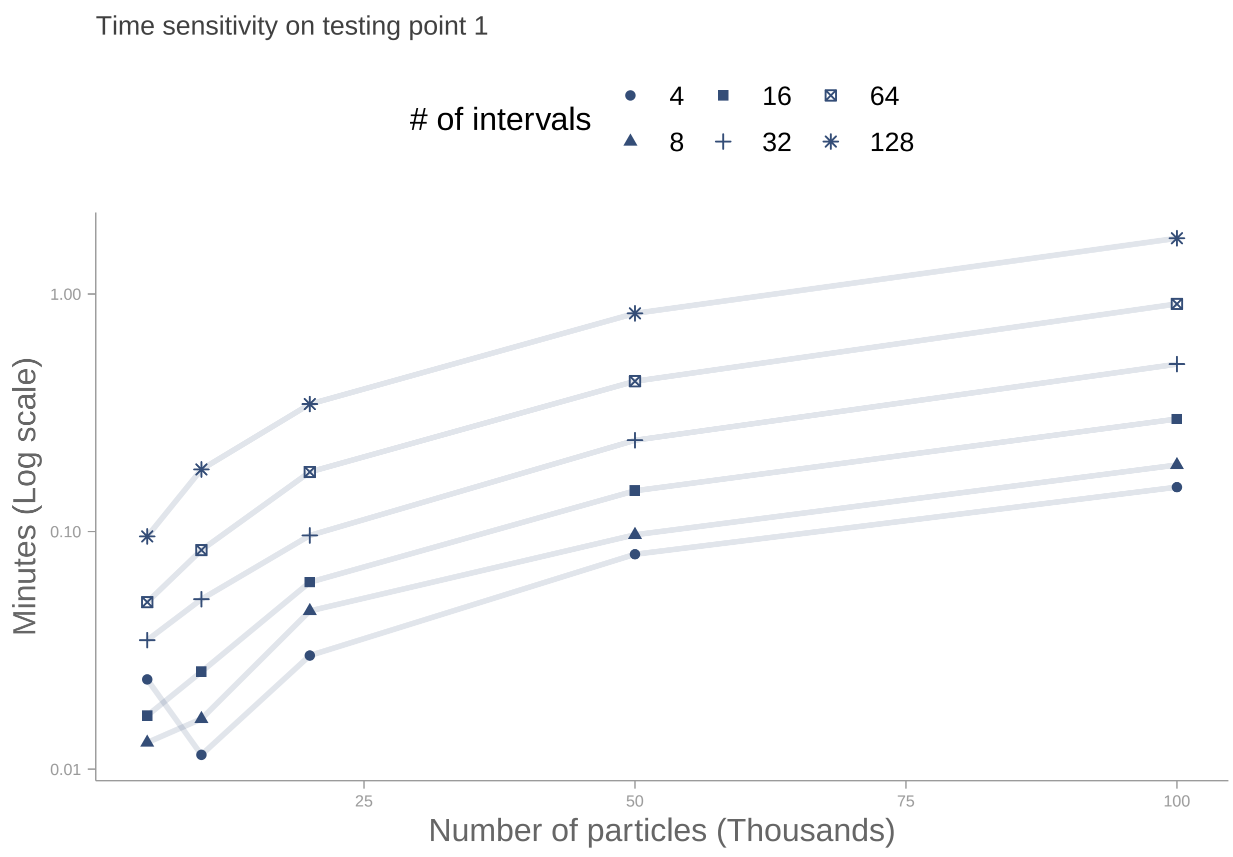

2.2.4.3 Convergence test

In this plot, we observe that the likelihood standard error approximates zero as the number of particles increases.

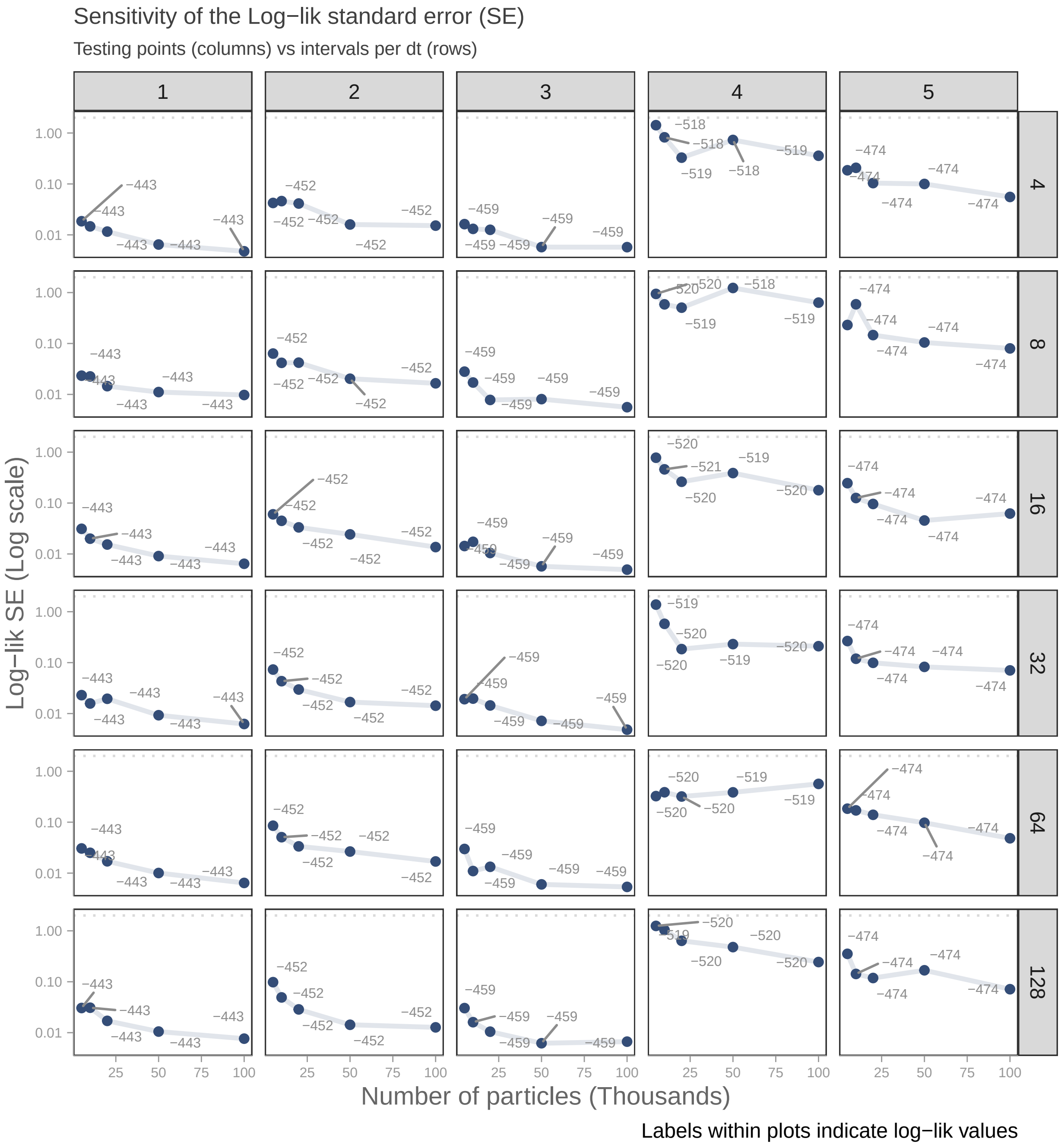

2.2.5 Candidate 3

This formulation assumes that daily incidence measurements are distributed according to the Poisson distribution. Also, this structure **incorporates** mobility data.

2.2.5.1 Equations

$$\frac{dC}{dt} = \eta P_t - C_t \delta(t \, mod \, 1) \tag{15}$$

$$y_d^1 \sim Pois(C_t) \tag{16}$$

$$y_d^2 \sim Normal(Z_t, \tau) \tag{17}$$

2.2.5.2 Convergence test

Mobility data does not redress convergence issues in the assumption that daily incidence measurements are distributed according to the Poisson distribution.

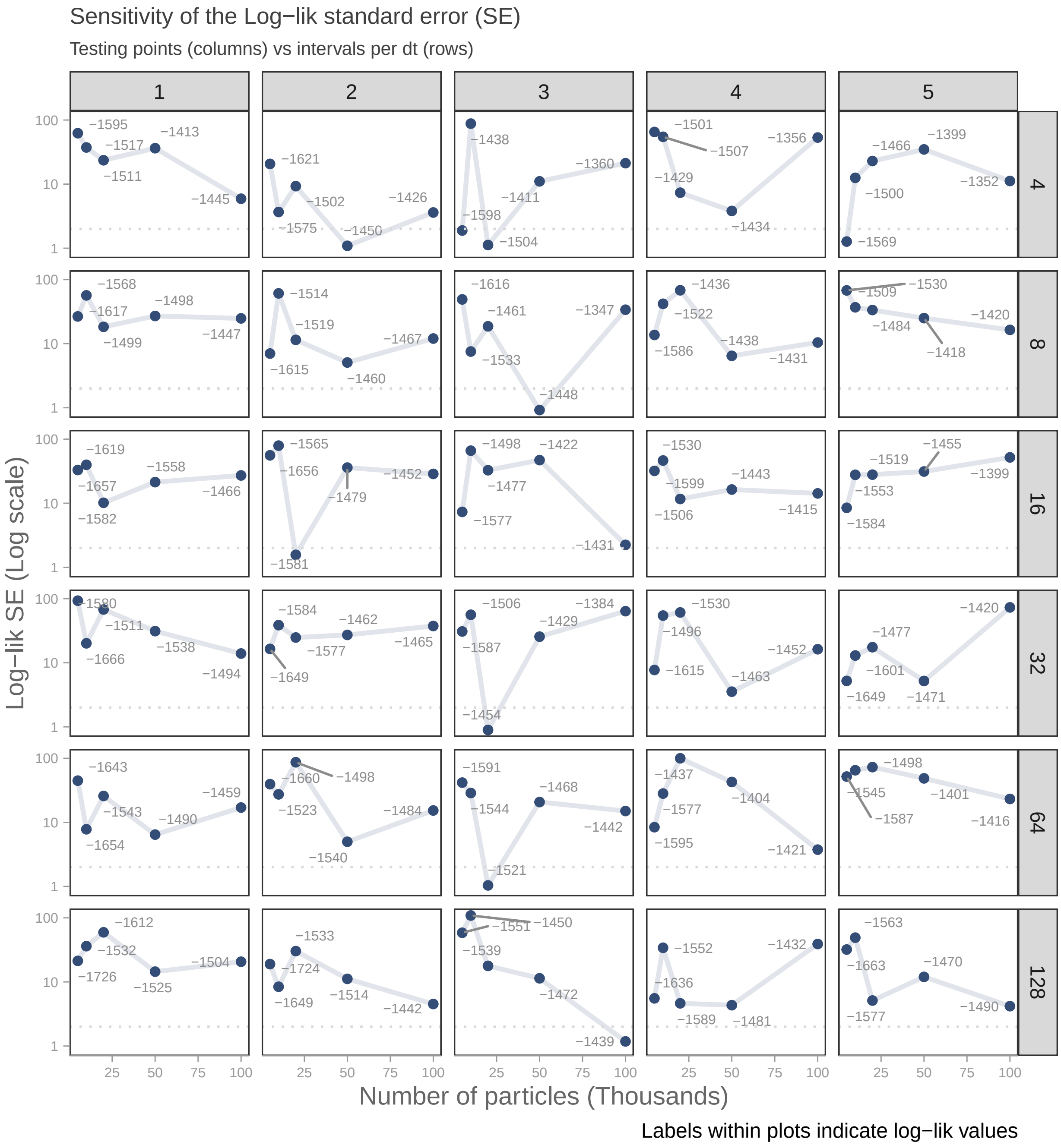

2.2.6 Candidate 4

This formulation assumes that daily incidence measurements are distributed according to the Negative Binomial distribution. Also, this structure **incorporates** mobility data.

2.2.6.1 Equations

$$\frac{dC}{dt} = \eta P_t - C_t \delta(t \, mod \, 1) \tag{18}$$

$$y_d^1 \sim Nbin(C_t, \phi^{-1}) \tag{19}$$

$$y_d^2 \sim Normal(Z_t, \tau) \tag{20}$$

2.2.6.2 Time comparison

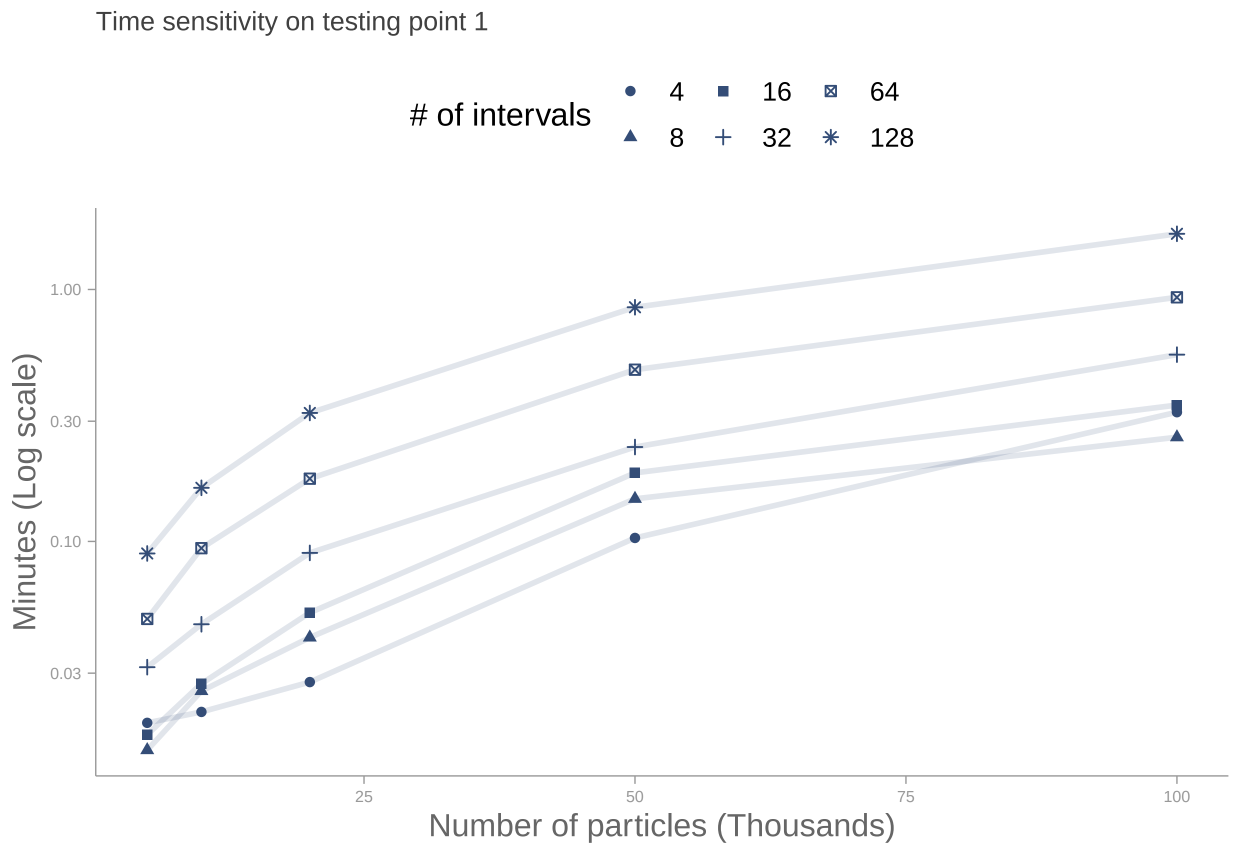

2.2.6.3 Convergence test

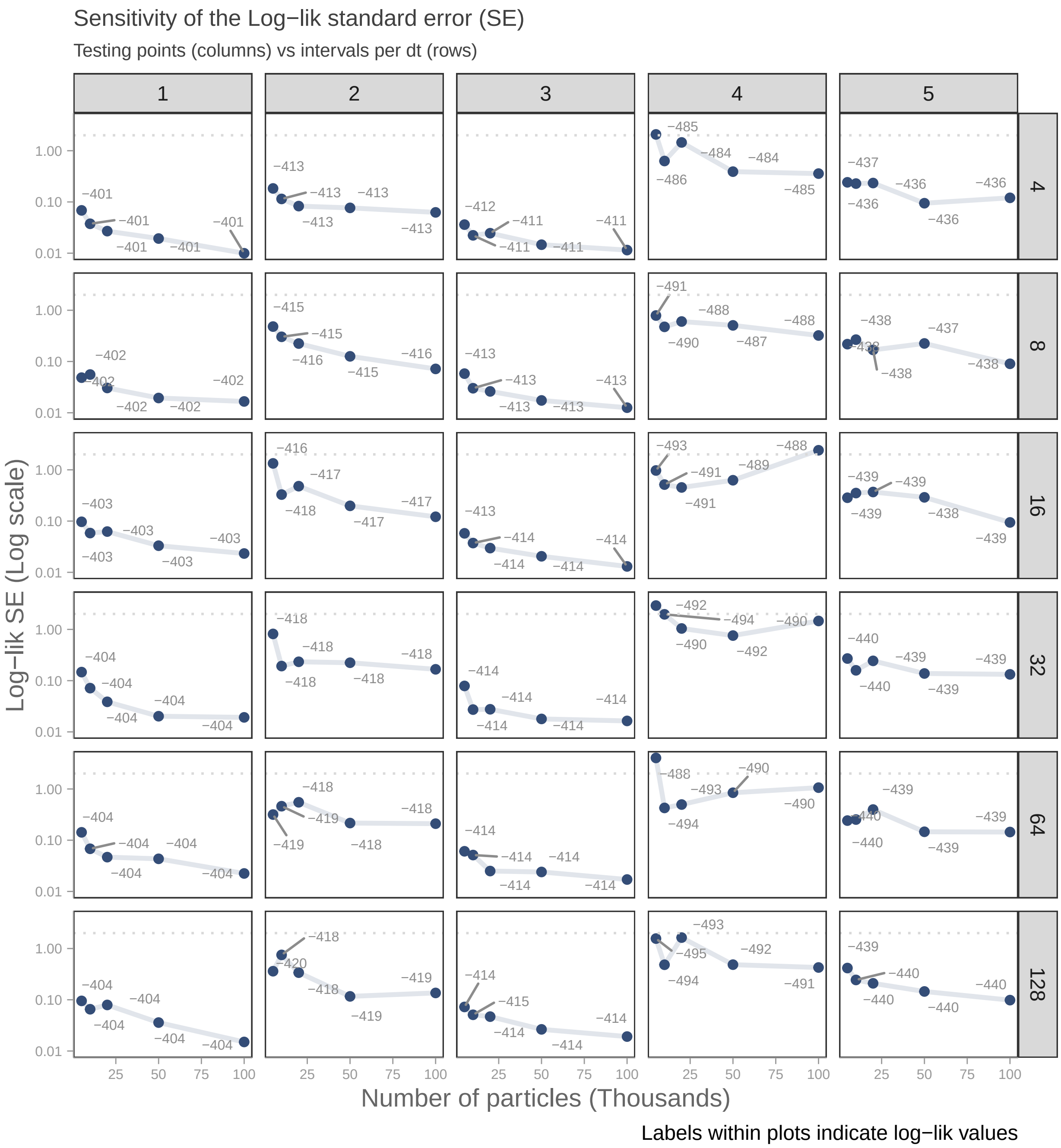

2.2.7 Candidate 5

This formulation assumes that **weekly** incidence measurements are distributed according to the Poisson distribution. Also, this structure **does not** incorporate mobility data.

2.2.7.1 Equations

$$\frac{dC}{dt} = \eta P_t - C_t \delta(t \, mod \, 7) \tag{21}$$

$$y_w^1 \sim Pois(C_t) \tag{22}$$

2.2.7.2 Time comparison

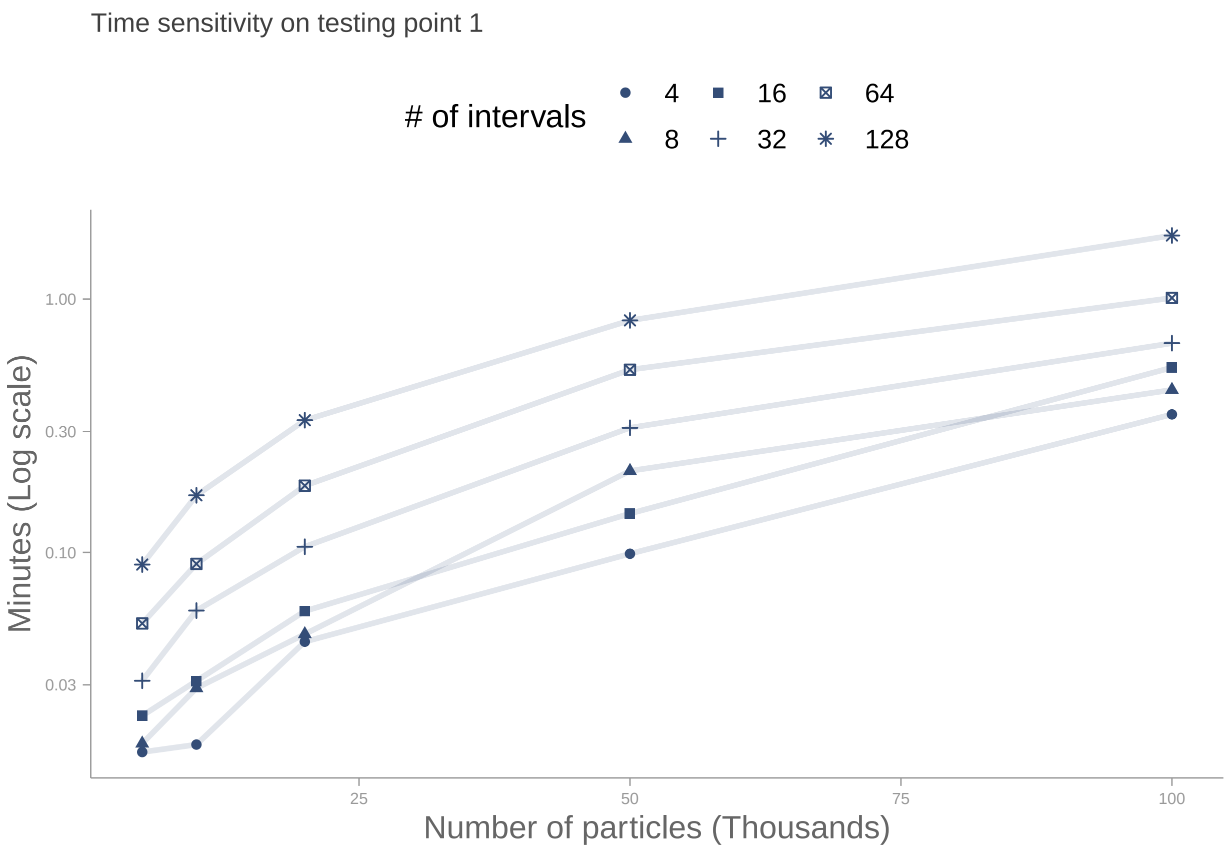

2.2.7.3 Convergence test

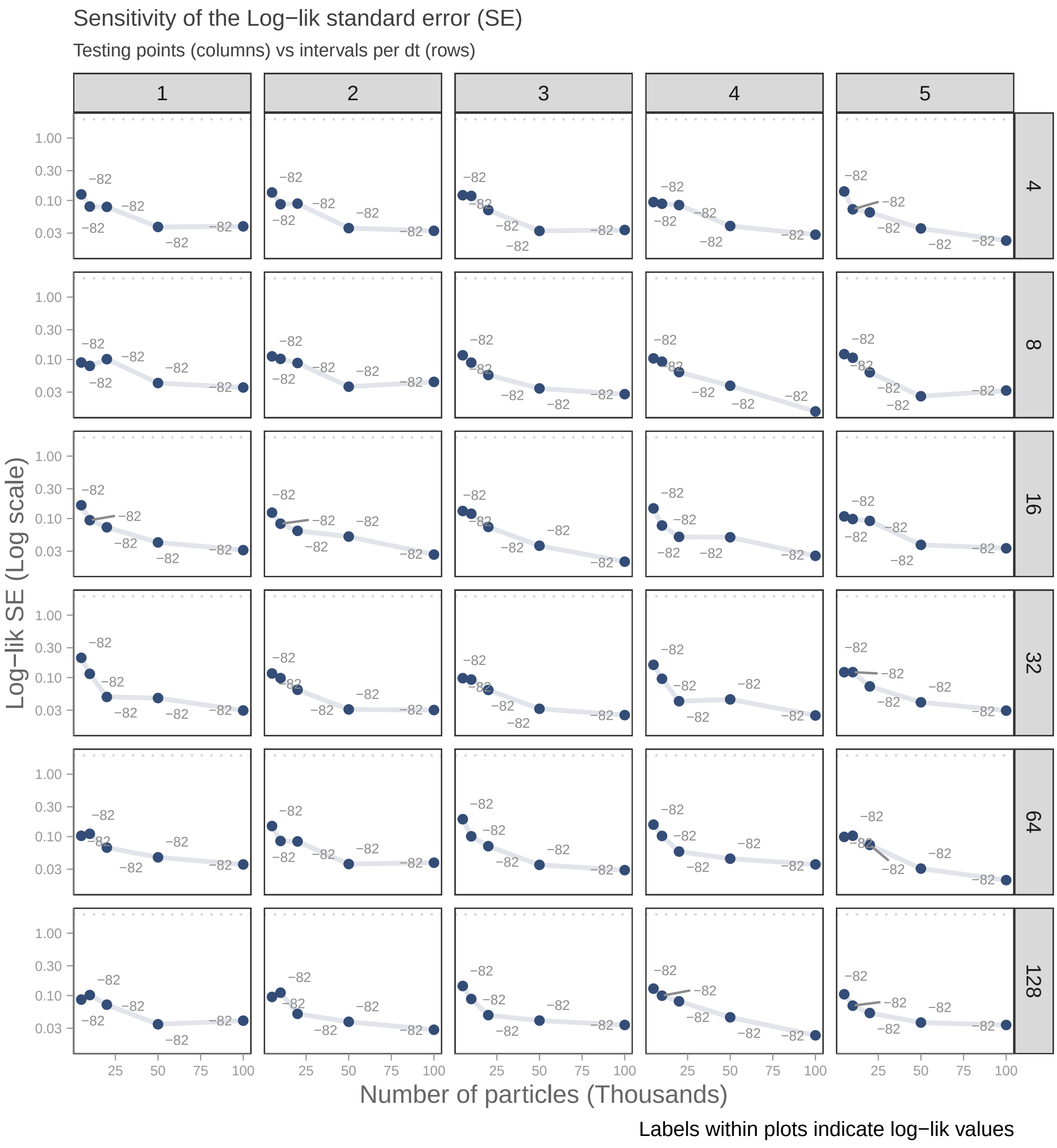

2.2.8 Candidate 6

This formulation assumes that **weekly** incidence measurements are distributed according to the Poisson distribution. Also, this structure **incorporates** mobility data.

2.2.8.1 Equations

$$\frac{dC}{dt} = \eta P_t - C_t \delta(t \, mod \, 7) \tag{23}$$

$$y_w^1 \sim Pois(C_t) \tag{24}$$

$$y_w^2 \sim Normal(Z_t, \tau) \tag{25}$$

2.2.8.2 Time comparison

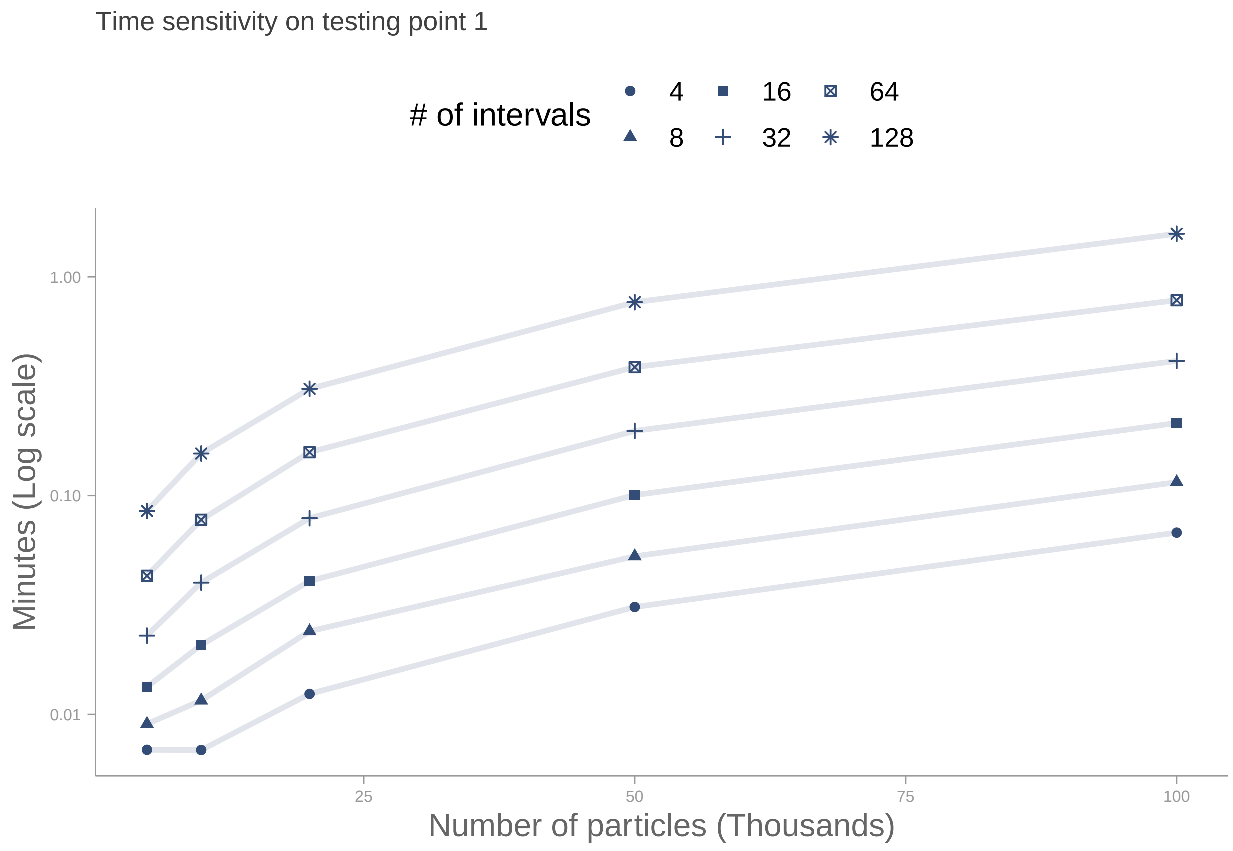

2.2.8.3 Convergence test

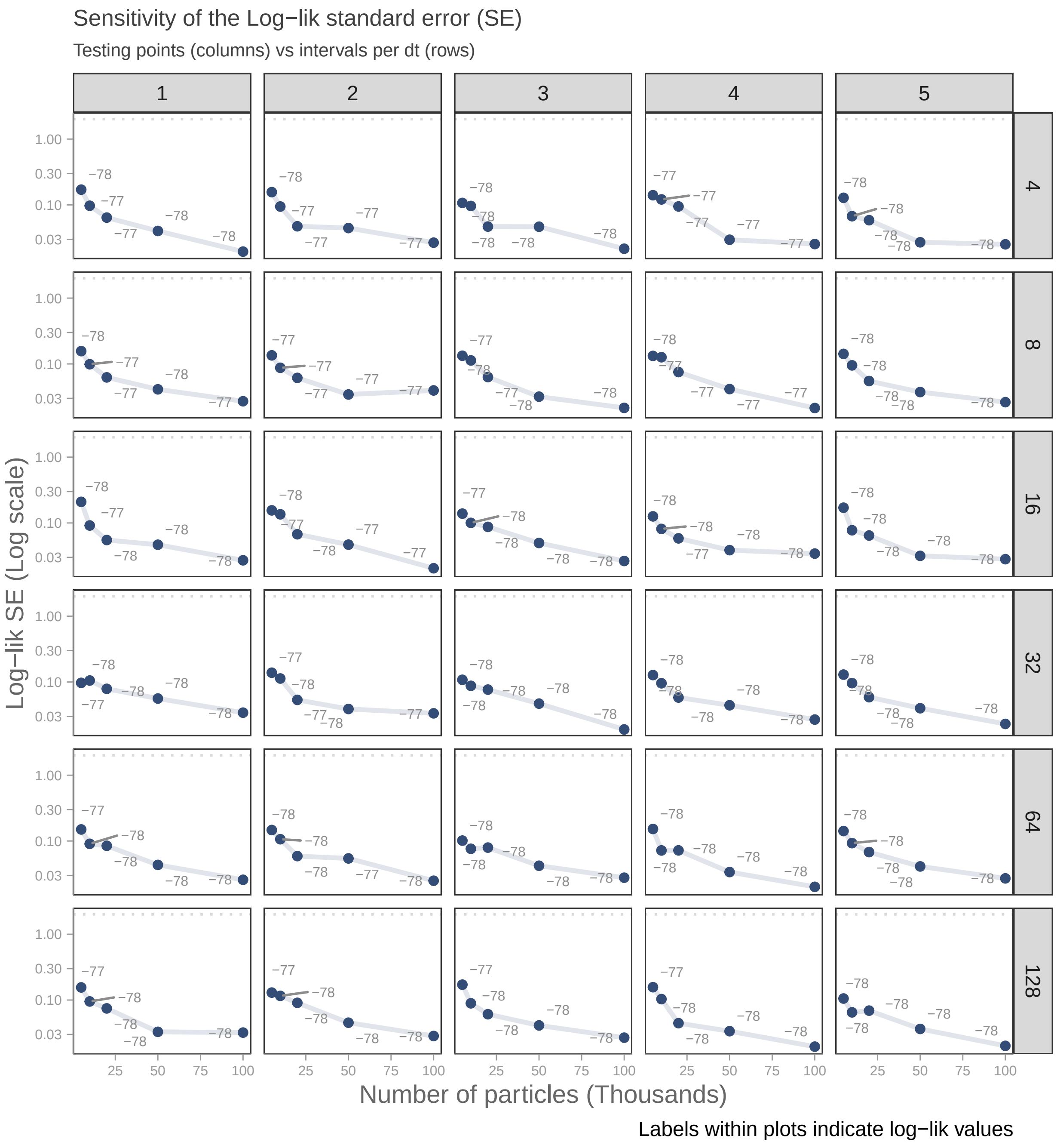

3 Cox-Ingersoll-Ross (DGP2)

3.1 Process model (PM2)

$$\frac{dS}{dt} = -S_t\lambda_t \tag{26}$$

$$\frac{dE}{dt} = S_t\lambda_t - \sigma E_t \tag{27}$$

$$\frac{dP}{dt} = \omega\sigma E_t - \eta P_t \tag{28}$$

$$\frac{dI}{dt} = \eta P_t - \gamma I_t \tag{29}$$

$$\frac{dA}{dt} = (1-\omega)\sigma E_t - \kappa A_t \tag{30}$$

$$\frac{dR}{dt} = \kappa A_t + \gamma I_t \tag{31}$$

$$\lambda_t = \frac{\beta_t(I_t + P_t + \mu A_t)}{N_t} \tag{32}$$

$$\beta_t = \zeta Z_t \tag{33}$$

$$\frac{dZ}{dt} = \nu(\upsilon - Z_t) + \sqrt{\alpha} Z_t dW \tag{34}$$

$$dW \sim Normal(0, \sqrt{dt}) \tag{35}$$

3.2 Testing measurement model candidates

3.2.1 Testing points

We use five point estimates (table below) as probes to assess the reliability of likelihood estimates. These point estimates correspond to *plausible* values obtained in a earlier version of this inference analysis. For the final version, we assume such values as **given**, and conduct the analysis from the start. Considering that these tests are not intended to be exhaustive but rather of exploratory nature, any arbitrary point is valid as a probe.

id	ζ	P_0	τ	ν	υ	α	ϕ
1	1.158	2.410	0.156	0.061	0.245	0.054	0.511
2	1.146	2.182	0.145	0.084	0.235	0.057	0.214
3	1.079	3.396	0.166	0.052	0.287	0.059	0.083
4	1.151	3.876	0.136	0.076	0.184	0.047	0.036
5	1.278	2.587	0.164	0.041	0.146	0.043	0.037

3.2.2 Summary

Similarly to the procedure followed in the previous section, we couple the CIR process model (PM2) with those six measurement model candidates formulated for the GBM process model (PM1) and repeat the analysis. In doing so, we obtain identical insights.

Id	Frequency	Incidence	Mobility	Converges
7	Daily	Pois	FALSE	No
8	Daily	Nbin	FALSE	Yes
9	Daily	Pois	TRUE	No
10	Daily	Nbin	TRUE	Yes
11	Weekly	Pois	FALSE	Yes
12	Weekly	Pois	TRUE	Yes

3.2.3 Candidate 7

This formulation assumes that daily incidence measurements are distributed according to the Poisson distribution. Also, this structure does not incorporate mobility data.

3.2.3.1 Equations

$$\frac{dC}{dt} = \eta P_t - C_t \delta(t \, mod \, 1) \tag{36}$$

$$y_d^1 \sim Pois(C_t) \tag{37}$$

3.2.3.2 Convergence test

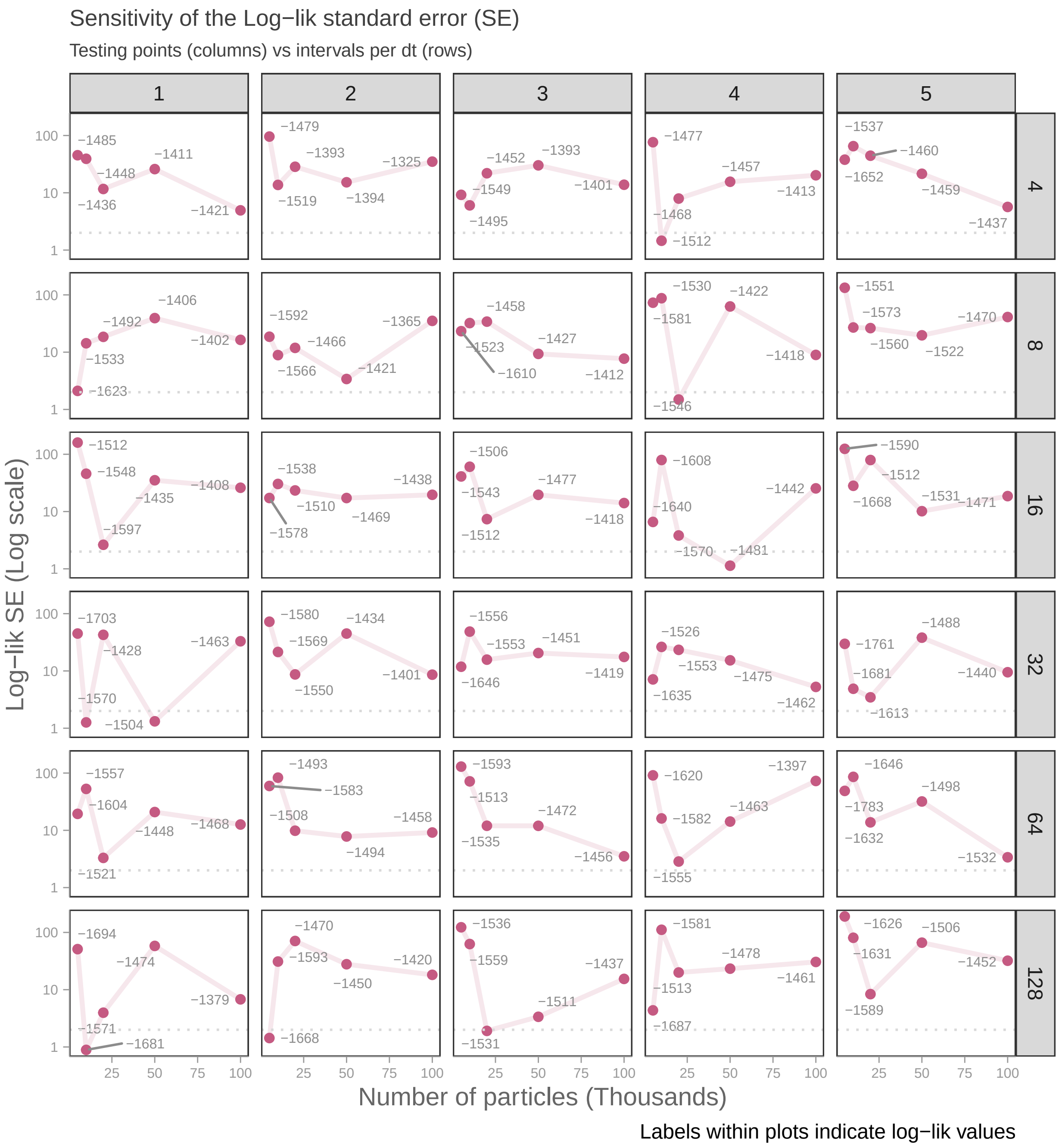

3.2.4 Candidate 8

This formulation assumes that daily incidence measurements are distributed according to the Negative binomial distribution. Also, this structure does not incorporate mobility data.

3.2.4.1 Equations

$$\frac{dC}{dt} = \eta P_t - C_t \delta(t \, mod \, 1) \tag{38}$$

$$y_d^1 \sim Nbin(C_t, \phi^{-1}) \tag{39}$$

3.2.4.2 Time comparison

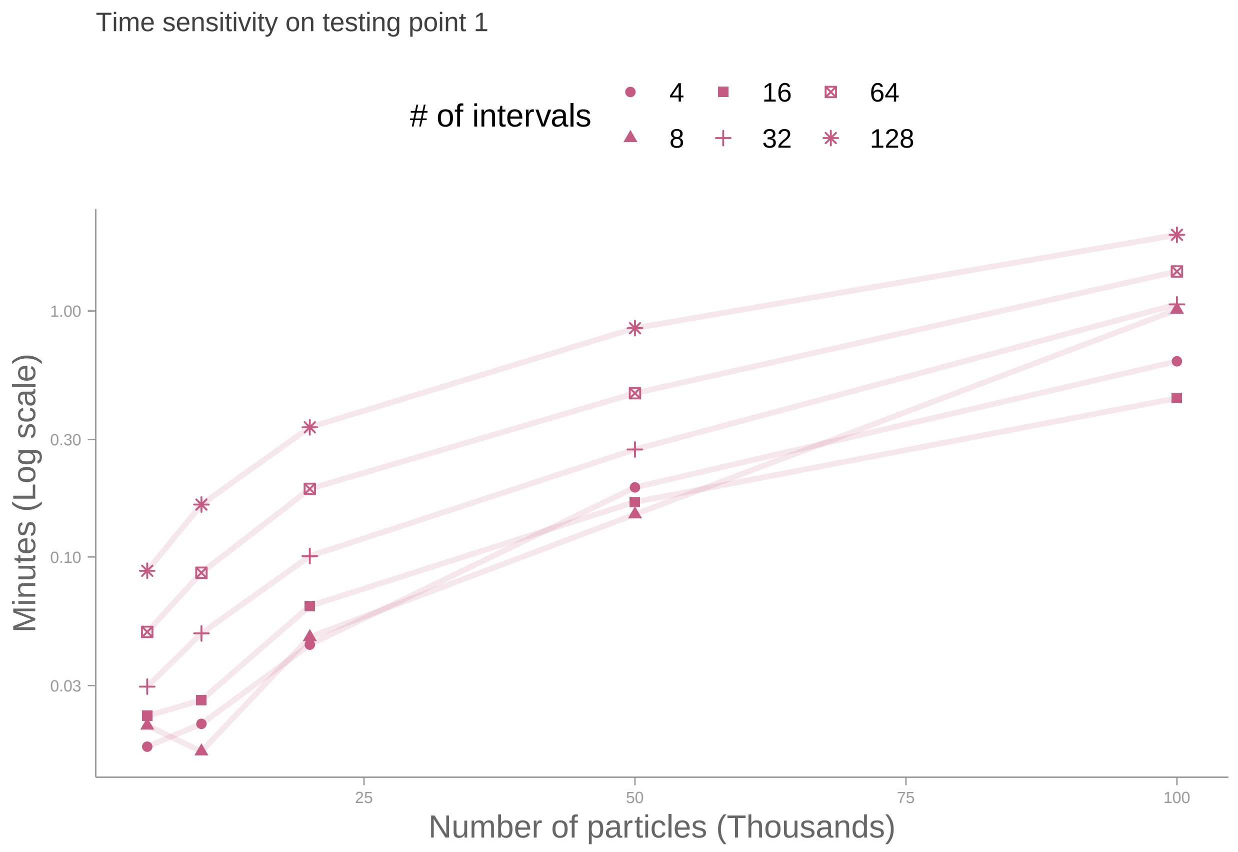

3.2.4.3 Convergence test

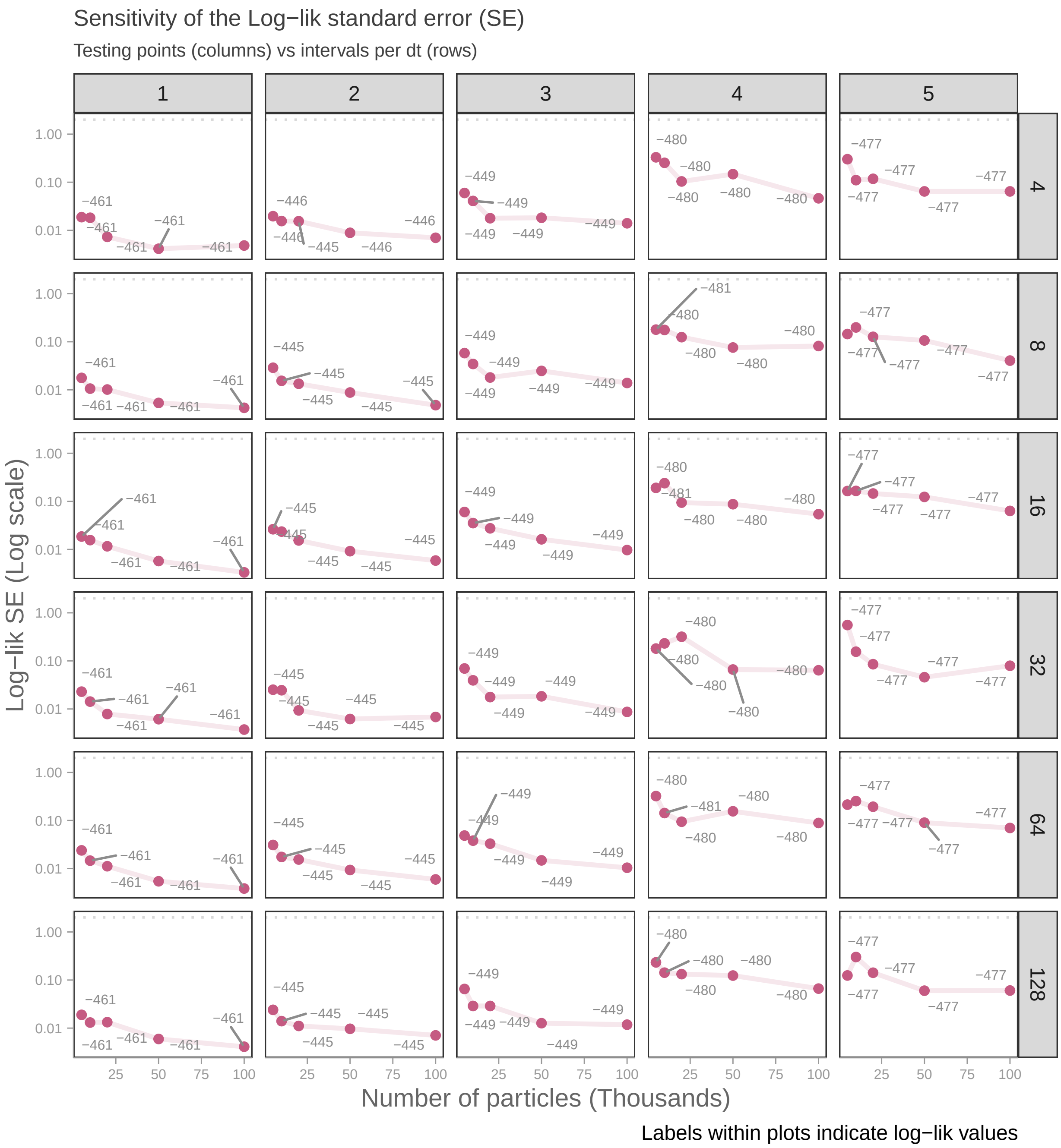

3.2.5 Candidate 9

This formulation assumes that daily incidence measurements are distributed according to the Poisson distribution. Also, this structure **incorporates** mobility data.

3.2.5.1 Equations

$$\frac{dC}{dt} = \eta P_t - C_t \delta(t \, mod \, 1) \tag{40}$$

$$y_d^1 \sim Pois(C_t) \tag{41}$$

$$y_d^2 \sim Normal(Z_t, \tau) \tag{42}$$

3.2.5.2 Convergence test

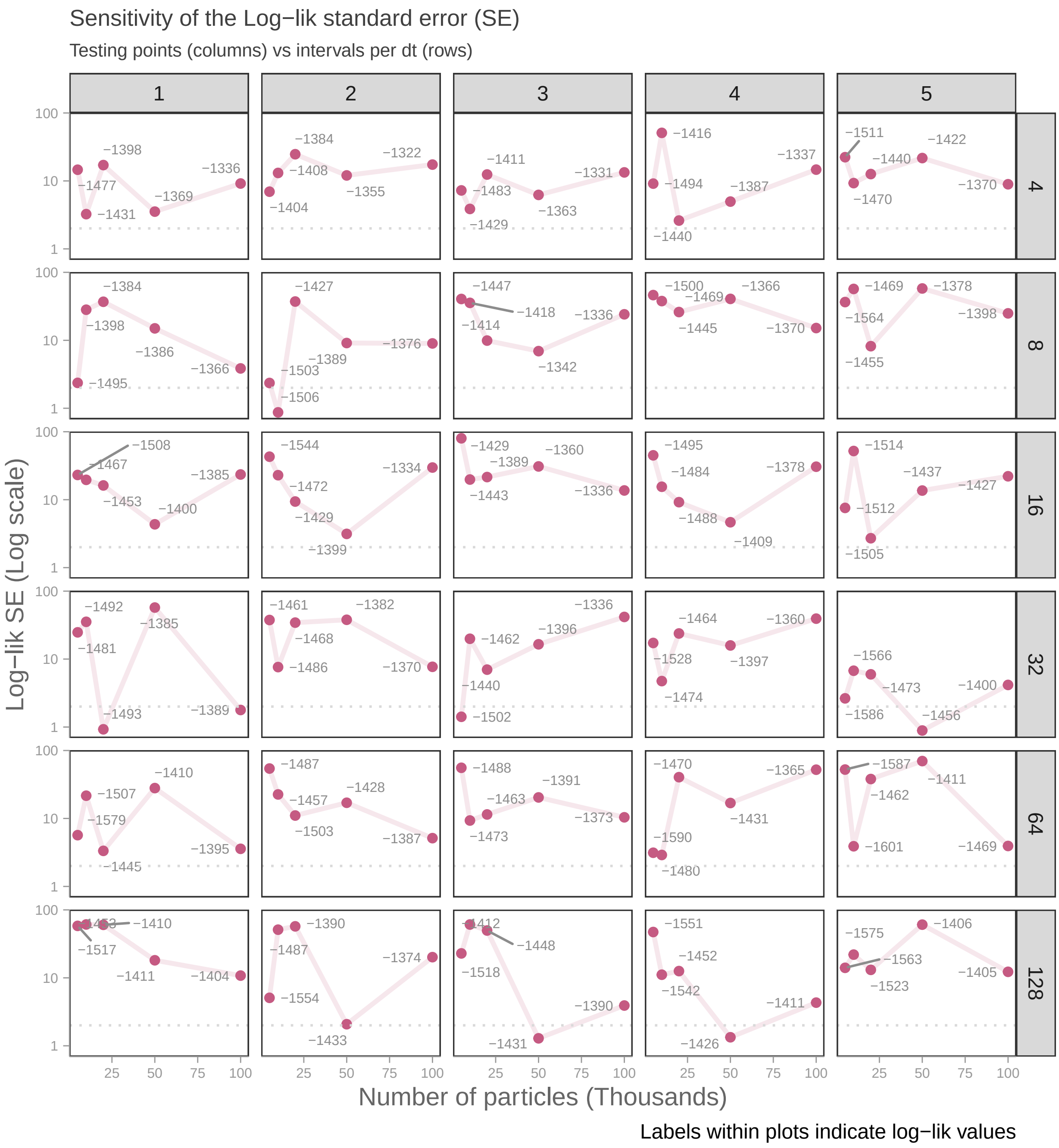

3.2.6 Candidate 10

This formulation assumes that daily incidence measurements are distributed according to the Negative Binomial distribution. Also, this structure **incorporates** mobility data.

3.2.6.1 Equations

$$\frac{dC}{dt} = \eta P_t - C_t \delta(t \, mod \, 1) \tag{43}$$

$$y_d^1 \sim Nbin(C_t, \phi^{-1}) \tag{44}$$

$$y_d^2 \sim Normal(Z_t, \tau) \tag{45}$$

3.2.6.2 Time comparison

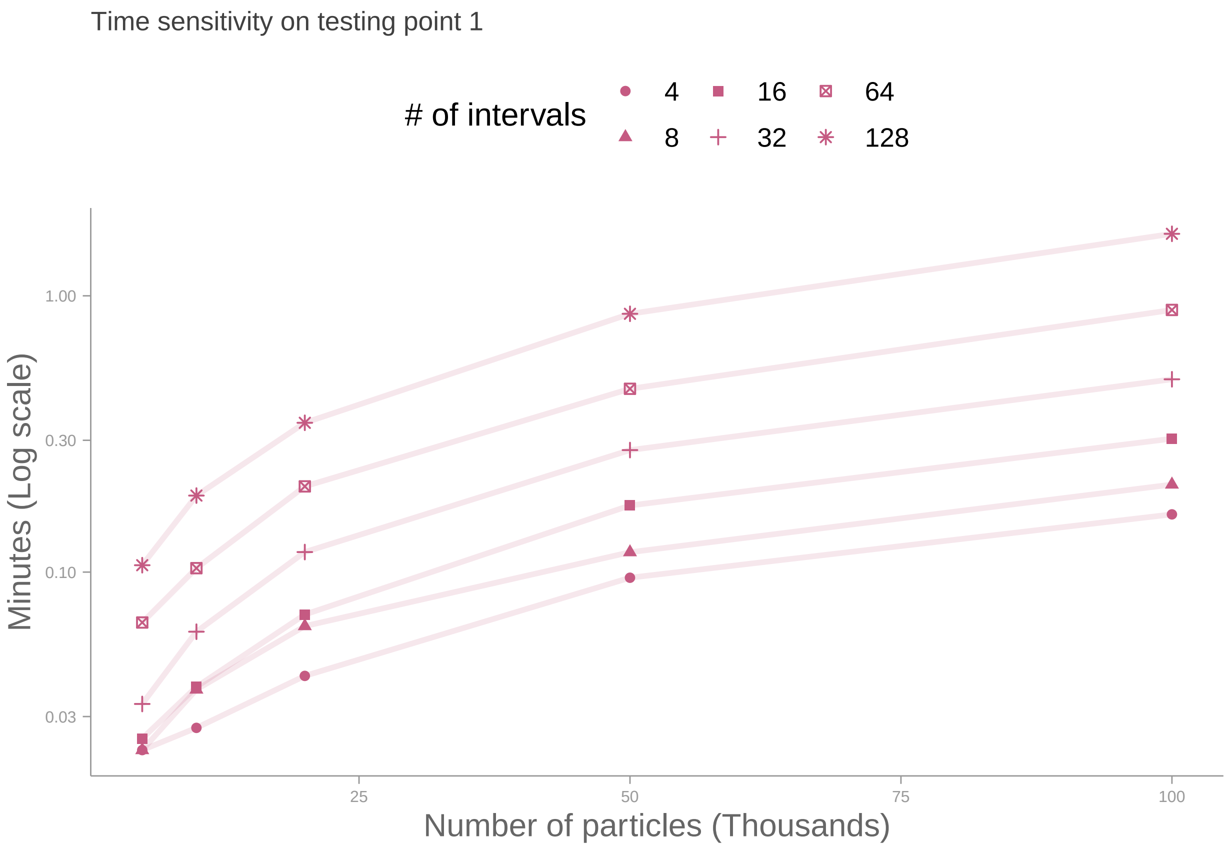

3.2.6.3 Convergence test

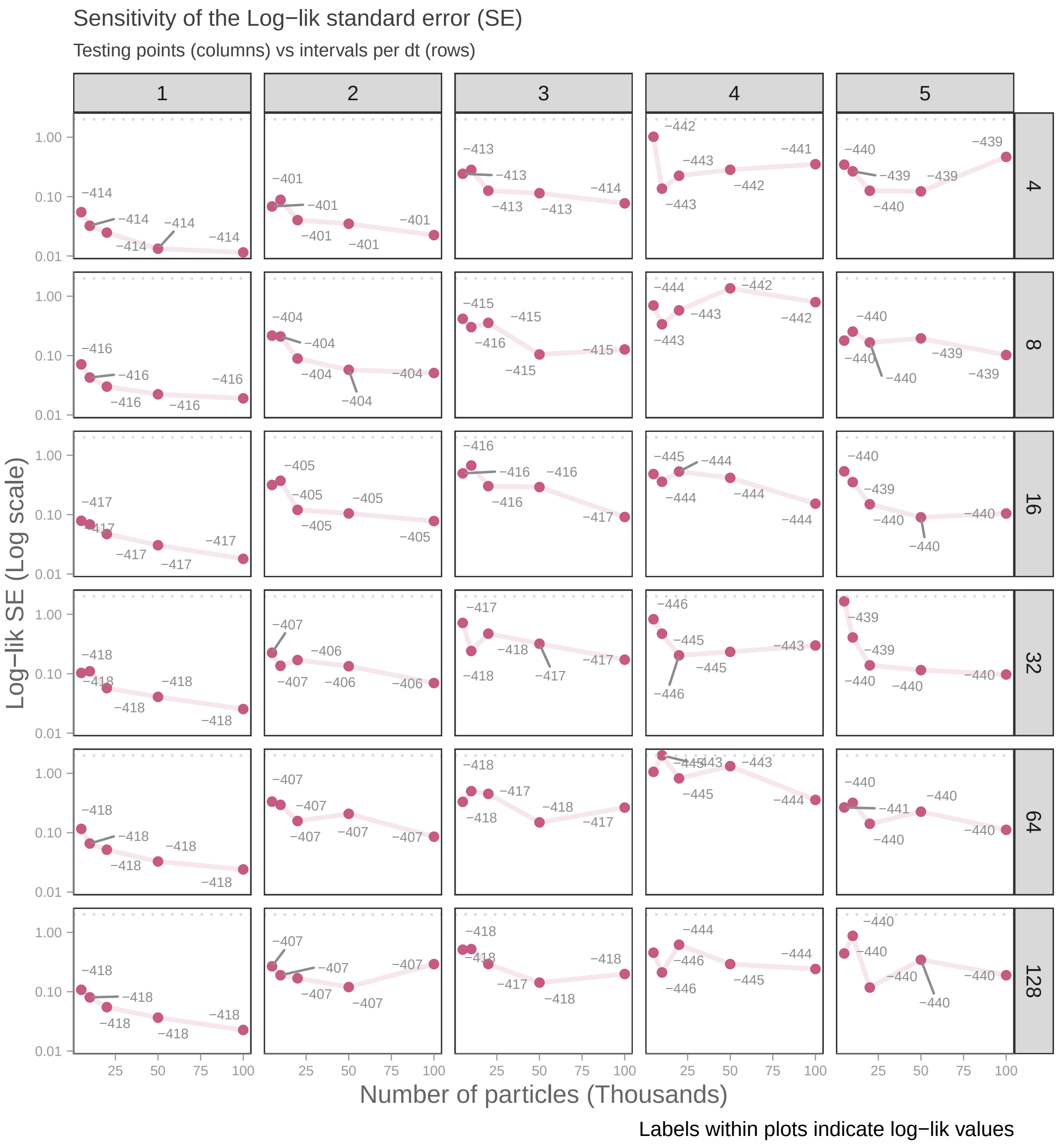

3.2.7 Candidate 11

This formulation assumes that **weekly** incidence measurements are distributed according to the Poisson distribution. Also, this structure **does not** incorporate mobility data.

3.2.7.1 Equations

$$\frac{dC}{dt} = \eta P_t - C_t \delta(t \, mod \, 7) \tag{46}$$

$$y_w^1 \sim Pois(C_t) \tag{47}$$

3.2.7.2 Time comparison

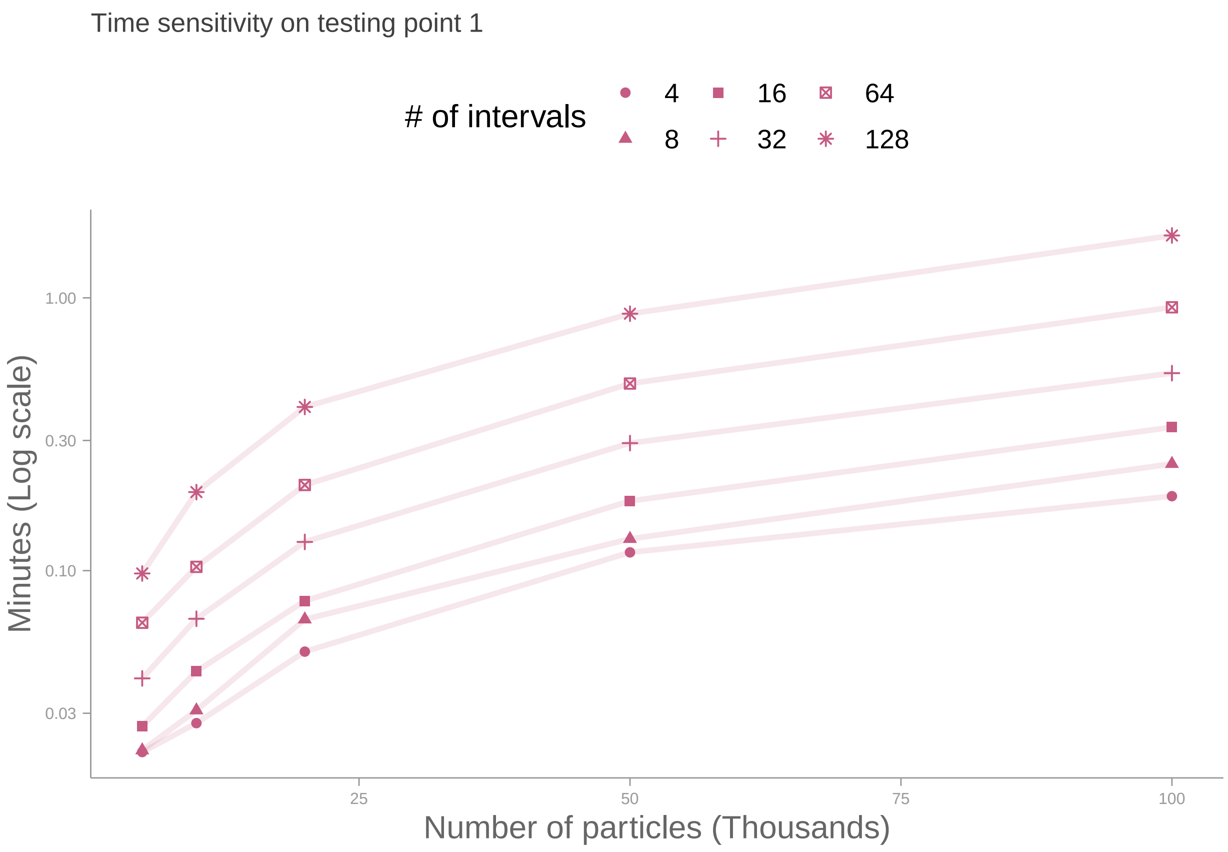

3.2.7.3 Convergence test

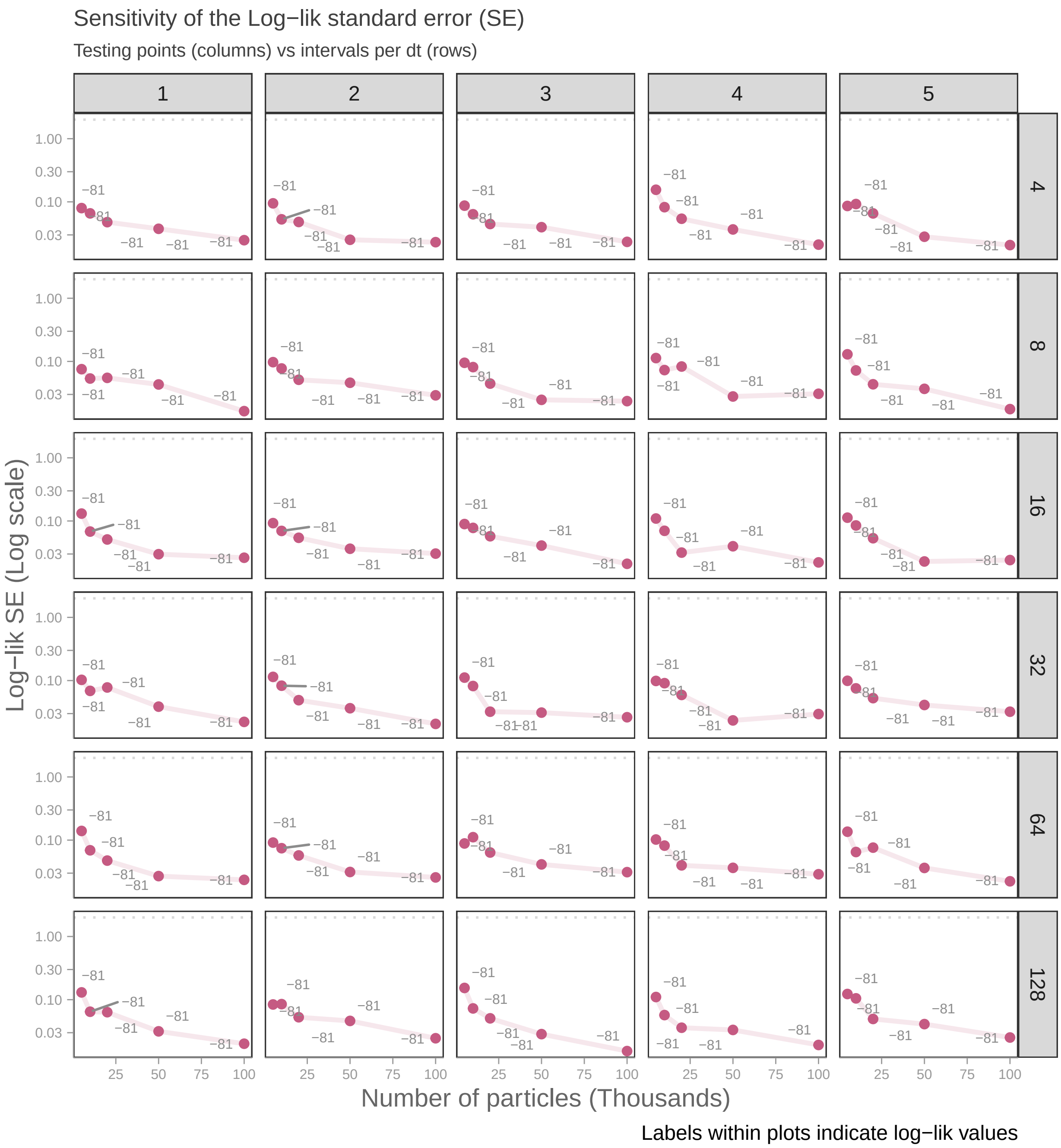

3.2.8 Candidate 12

This formulation assumes that **weekly** incidence measurements are distributed according to the Poisson distribution. Also, this structure **incorporates** mobility data.

3.2.8.1 Equations

$$\frac{dC}{dt} = \eta P_t - C_t \delta(t \, mod \, 7) \tag{48}$$

$$y_w^1 \sim Pois(C_t) \tag{49}$$

$$y_w^2 \sim Normal(Z_t, \tau) \tag{50}$$

3.2.8.2 Time comparison

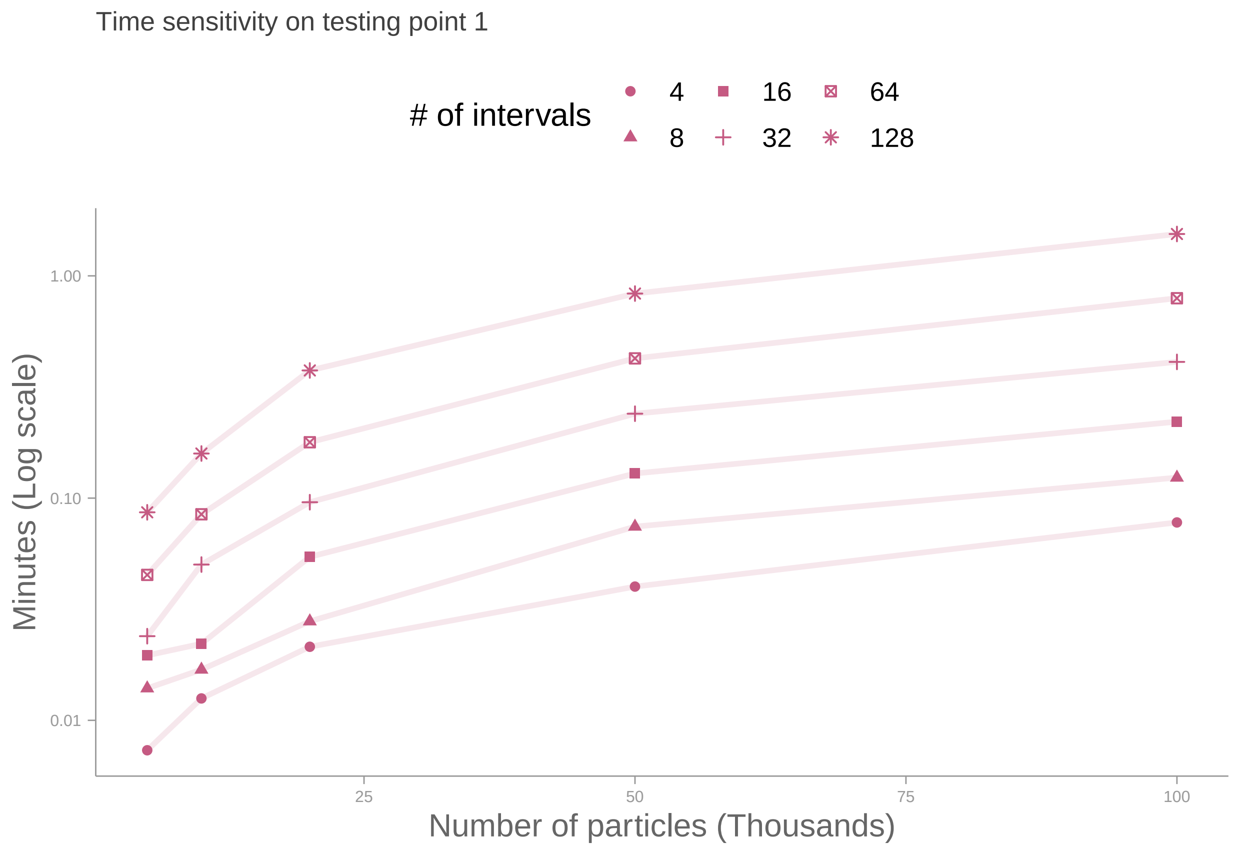

3.2.8.3 Convergence test

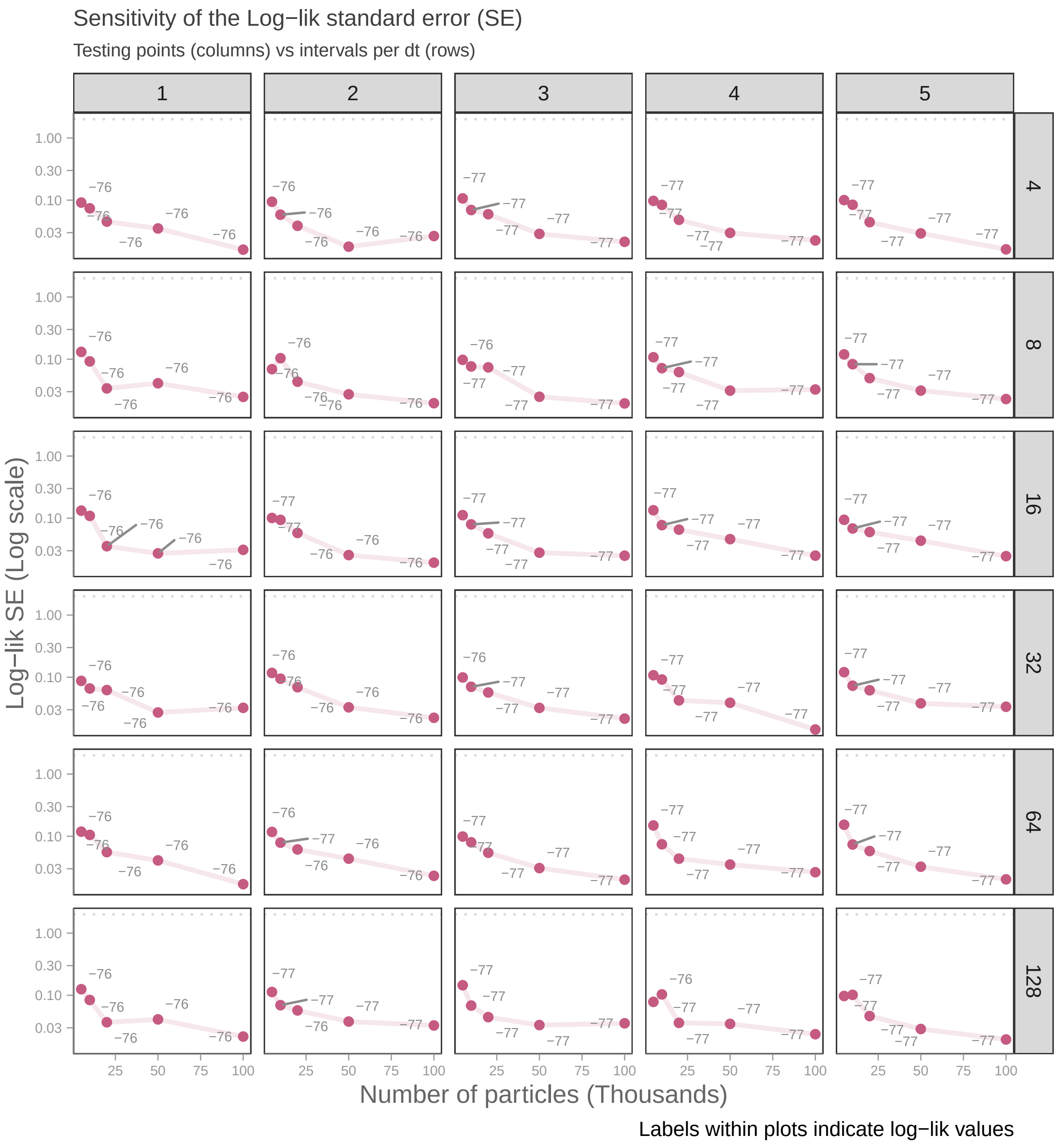

4 Original Computing Environment

```
## R version 4.1.2 (2021-11-01)
## Platform: aarch64-apple-darwin20 (64-bit)
## Running under: macOS Monterey 12.3.1
##
## Matrix products: default
## BLAS:   /Library/Frameworks/R.framework/Versions/4.1-arm64/Resources/lib/libRblas.0.dylib
## LAPACK: /Library/Frameworks/R.framework/Versions/4.1-arm64/Resources/lib/libRlapack.dylib
##
## locale:
## [1] en_US.UTF-8/en_US.UTF-8/en_US.UTF-8/C/en_US.UTF-8/en_US.UTF-8
##
## attached base packages:
## [1] parallel  stats     graphics  grDevices utils     datasets  methods
## [8] base
##
## other attached packages:
##  [1] scales_1.1.1      patchwork_1.1.1   ggrepel_0.9.1     ggpubr_0.4.0
##  [5] ggalt_0.4.0       GGally_2.1.2      ggplot2_3.3.5     tictoc_1.0.1
##  [9] stringr_1.4.0     tidyr_1.1.4       readxl_1.3.1      readr_2.1.1
## [13] purrr_0.3.4       pomp_3.6          lubridate_1.8.0   imputeTS_3.2
## [17] kableExtra_1.3.4  extraDistr_1.9.1  dplyr_1.0.8       doRNG_1.8.2
## [21] rngtools_1.5.2    doParallel_1.0.16 iterators_1.0.13  foreach_1.5.1
##
## loaded via a namespace (and not attached):
##  [1] colorspace_2.0-2   ggsignif_0.6.3     ellipsis_0.3.2     gridtext_0.1.4
##  [5] ggtext_0.1.1       rstudioapi_0.13    farver_2.1.0       bit64_4.0.5
##  [9] fansi_0.5.0        mvtnorm_1.1-3      xml2_1.3.3         codetools_0.2-18
## [13] splines_4.1.2      extrafont_0.17     knitr_1.37         broom_0.7.10
## [17] Rttf2pt1_1.3.9     compiler_4.1.2     httr_1.4.2         backports_1.4.1
## [21] Matrix_1.3-4       fastmap_1.1.0      cli_3.3.0          htmltools_0.5.2
## [25] tools_4.1.2        coda_0.19-4        gtable_0.3.0       glue_1.6.2
## [29] reshape2_1.4.4     maps_3.4.0         Rcpp_1.0.7         carData_3.0-4
## [33] cellranger_1.1.0   fracdiff_1.5-1     vctrs_0.4.1        urca_1.3-0
## [37] svglite_2.0.0      nlme_3.1-153       extrafontdb_1.0    lmtest_0.9-39
## [41] timeDate_3043.102  xfun_0.29          rvest_1.0.2        lifecycle_1.0.1
## [45] rstatix_0.7.0      MASS_7.3-54        zoo_1.8-9          vroom_1.5.7
## [49] hms_1.1.1          proj4_1.0-10.1     RColorBrewer_1.1-2 yaml_2.2.1
## [53] quantmod_0.4.18    curl_4.3.2         reshape_0.8.8      stringi_1.7.6
## [57] highr_0.9          tseries_0.10-49    TTR_0.24.3         rlang_1.0.2
## [61] pkgconfig_2.0.3    systemfonts_1.0.3  evaluate_0.14      lattice_0.20-45
## [65] labeling_0.4.2     stinepack_1.4      bit_4.0.4          tidyselect_1.1.1
## [69] deSolve_1.30       plyr_1.8.6         magrittr_2.0.3     R6_2.5.1
## [73] generics_0.1.1     pillar_1.6.4       withr_2.4.3        mgcv_1.8-38
## [77] xts_0.12.1         abind_1.4-5        nnet_7.3-16        ash_1.0-15
## [81] tibble_3.1.6       crayon_1.4.2       car_3.0-12         KernSmooth_2.23-20
## [85] utf8_1.2.2         tzdb_0.2.0         rmarkdown_2.11     grid_4.1.2
## [89] forecast_8.15      digest_0.6.29      webshot_0.5.2      munsell_0.5.0
## [93] viridisLite_0.4.0  quadprog_1.5-8
```
