# Supplementary material for: Inferring the effective reproductive number from deterministic and semi-deterministic compartmental models using incidence and mobility data
Source: PLoS Comput Biol. 2022 Jun 27;18(6):e1010206. doi: 10.1371/journal.pcbi.1010206 (PMC9269962; doi:10.1371/journal.pcbi.1010206)
Supplement: S4 Text — (PDF) [file pcbi.1010206.s004.pdf]

S4 Appendix

This appendix illustrates the inference process carried out on one of DGP1's structure candidates. This candidate's process model consists of an SEIR-type formulation whose relative effective contact rate is described by Geometric Brownian Motion. Moreover, this candidate's measurement model assumes that weekly incidence counts are distributed according to the **Poisson distribution**. We refer to this model in S1 as **Candidate 5**. In particular, we apply *Iterated Filtering* and the *Particle Filter* to obtain from, Candidate 5, estimates (via samples) for the effective reproductive number and other parameters that explain Ireland's first wave of COVID-19 in 2020.

Contents

1 Structure **2**
1.1 Process model . . . 2
1.2 Measurement model . . . 2
1.3 Unmodelled predictors . . . 3
1.4 Unknown parameters . . . 3

2 Parameter inference **4**
2.1 Local search . . . 4
2.1.1 Likelihood maximisation . . . 4
2.1.2 Likelihood estimates . . . 5
2.2 Global search . . . 6
2.2.1 Likelihood maximisation . . . 6
2.2.2 Likelihood estimates . . . 7
2.3 Profile likelihood . . . 8
2.3.1 ζ - Initial effective contact rate . . . 8
2.3.2 P_0 - Initial number of preclinical individuals . . . 10
2.3.3 α - Volatility of effective contact rate . . . 12
2.3.4 Estimates . . . 14

3 Prediction **16**
3.1 Sampling space . . . 16
3.2 Draws . . . 17
3.3 Hidden states . . . 18

4 Original Computing Environment **19**

1 Structure

1.1 Process model

$$\frac{dS}{dt} = -S_t \lambda_t \tag{1}$$

$$\frac{dE}{dt} = S_t \lambda_t - \sigma E_t \tag{2}$$

$$\frac{dP}{dt} = \omega \sigma E_t - \eta P_t \tag{3}$$

$$\frac{dI}{dt} = \eta P_t - \gamma I_t \tag{4}$$

$$\frac{dA}{dt} = (1 - \omega)\sigma E_t - \kappa A_t \tag{5}$$

$$\frac{dR}{dt} = \kappa A_t + \gamma I_t \tag{6}$$

$$\lambda_t = \frac{\beta_t (I_t + P_t + \mu A_t)}{N_t} \tag{7}$$

$$\beta_t = \zeta Z_t \tag{8}$$

$$\frac{dZ}{dt} = \alpha Z_t dW \tag{9}$$

$$dW \sim Normal(0, \sqrt{dt}) \tag{10}$$

1.2 Measurement model

$$\frac{dC}{dt} = \eta P_t - C_t \delta(t \, mod \, 7) \tag{11}$$

$$y_w^1 \sim Pois(C_t) \tag{12}$$

1.3 Unmodelled predictors

Name	Symbol	Value	Units	Source
Incubation period	σ^{-1}	3	Days	Davies (2020)
Duration of preclinical infectiousness	η^{-1}	2.1	Days	Davies (2020)
Duration of clinical infectiousness	γ^{-1}	2.9	Days	Davies (2020)
Clinical fraction	ω	0.7	Unitless	HPSC (2020)
Asymptomatic infectious period	κ^{-1}	5	Days	Davies (2020)
Population	N_0	4937796	People	United Nations (2019)
Relative infectiousness	μ	0.5	Unitless	Davies (2020)
Initial susceptible	S_0	$N_0 - P_0$	People	Assumption
Initial exposed	E_0	0	People	Assumption
Initial clinical infectious	I_0	0	People	Assumption
Initial recovered	R_0	0	People	Assumption
Initial subclinical infectious	A_0	0	People	Assumption
Initial reported cases	C_0	0	People	By definition
Initial mobility effect	Z_0	1	Unitless	By definition

1.4 Unknown parameters

Name	Symbol	Units
Initial effective contact rate	ζ	People / day
Volatility of effective contact rate	α	Unitless
Initial preclinical infectious	P_0	People

2 Parameter inference

2.1 Local search

We start the inference process with a preliminary test. Specifically, we verify that Iterated Filtering algorithm, applied to this DGP (DGP1) and data, converges to regions of high likelihood.

2.1.1 Likelihood maximisation

Accordingly, from a single point in the parameter space, we search for the Maximum Likelihood Estimate (MLE) via Iterated Filtering. We repeat this process **twenty** times.

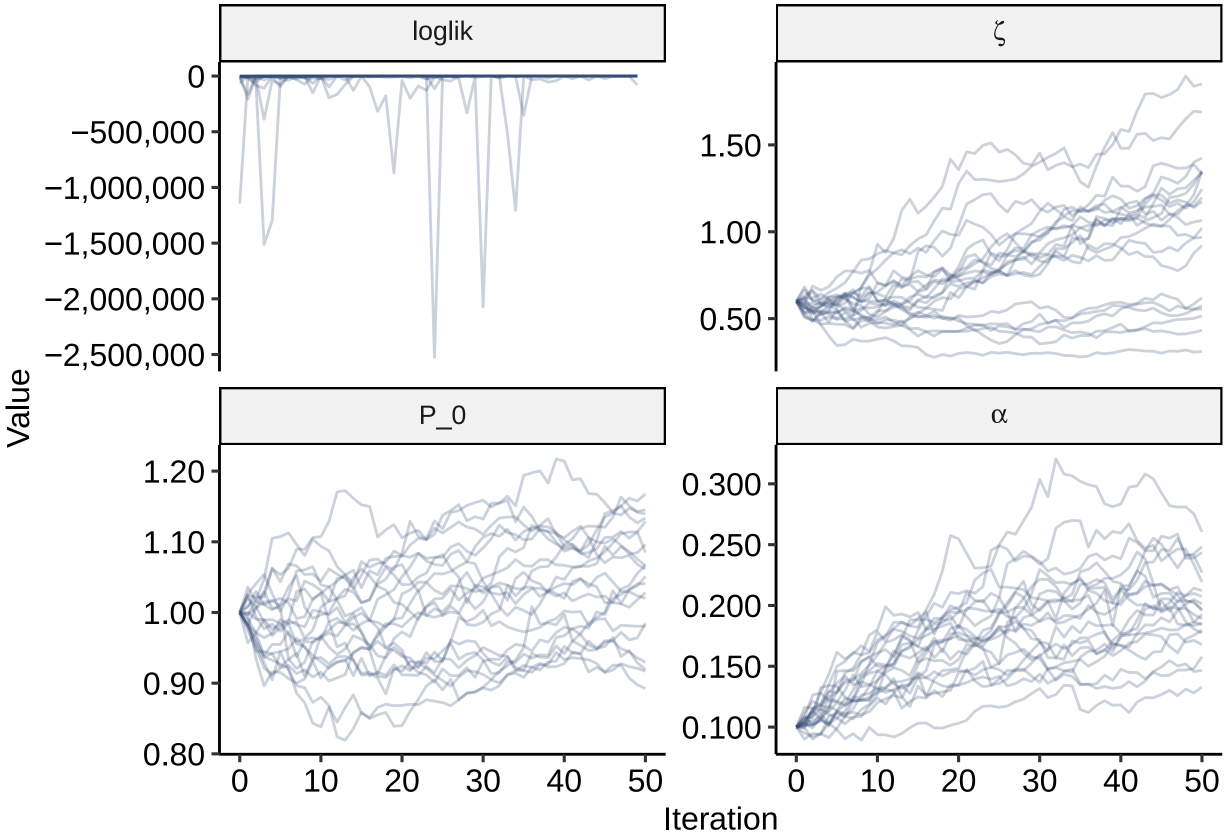

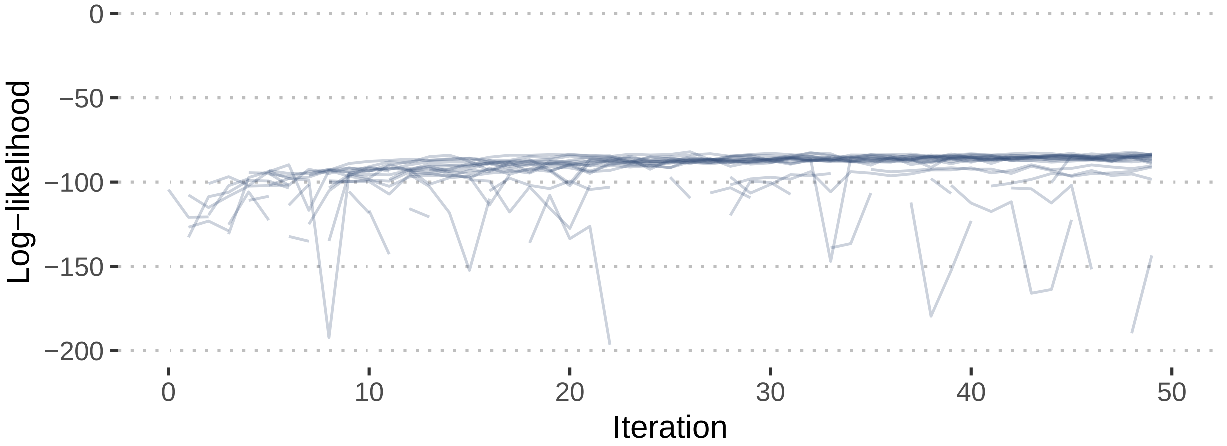

2.1.2 Likelihood estimates

The likelihood estimates obtained from the Iterated Filtering algorithm are merely an approximation to the actual values at those points. This difference occurs for two reasons: the Iterated Filtering algorithm is run with fewer particles than are needed for a good likelihood evaluation; 2) the stochastic perturbations applied to the inferred parameters at each iteration. Consequently, it is necessary to run the Particle Filter to obtain reliable likelihood estimates. Specifically, we use the values from each run's final filtering iteration as inputs to the Particle Filter.

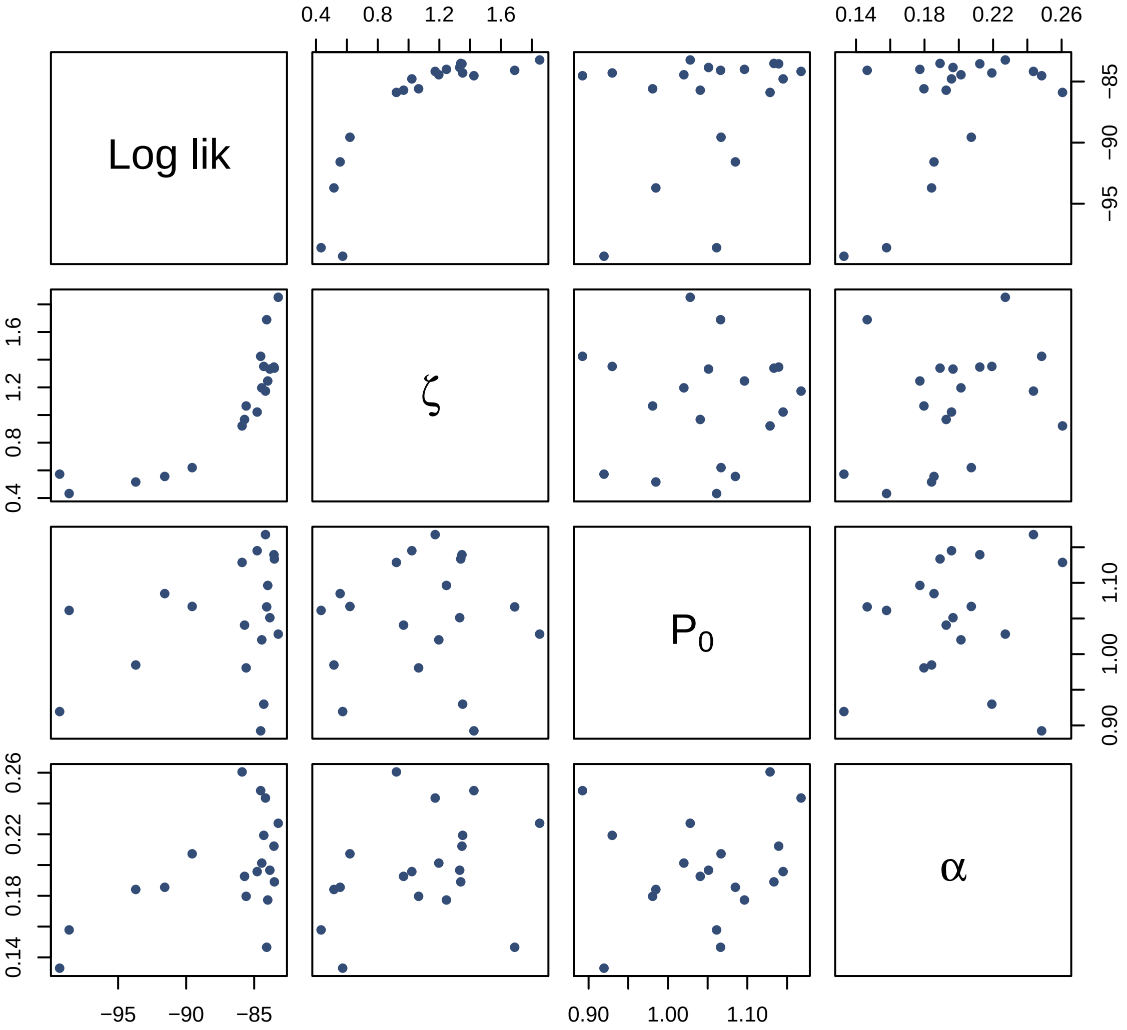

2.2 Global search

In this step, we follow a similar process described in Section 2.1, but this time increasing the number of starting points (300) and filtering iterations. Also, there is only one run for each starting point (in contrast with the 20 runs in Section 2.1). We refer to this step as *global search*, whose purpose is to construct a likelihood surface that allows us to identify regions of high plausibility.

2.2.1 Likelihood maximisation

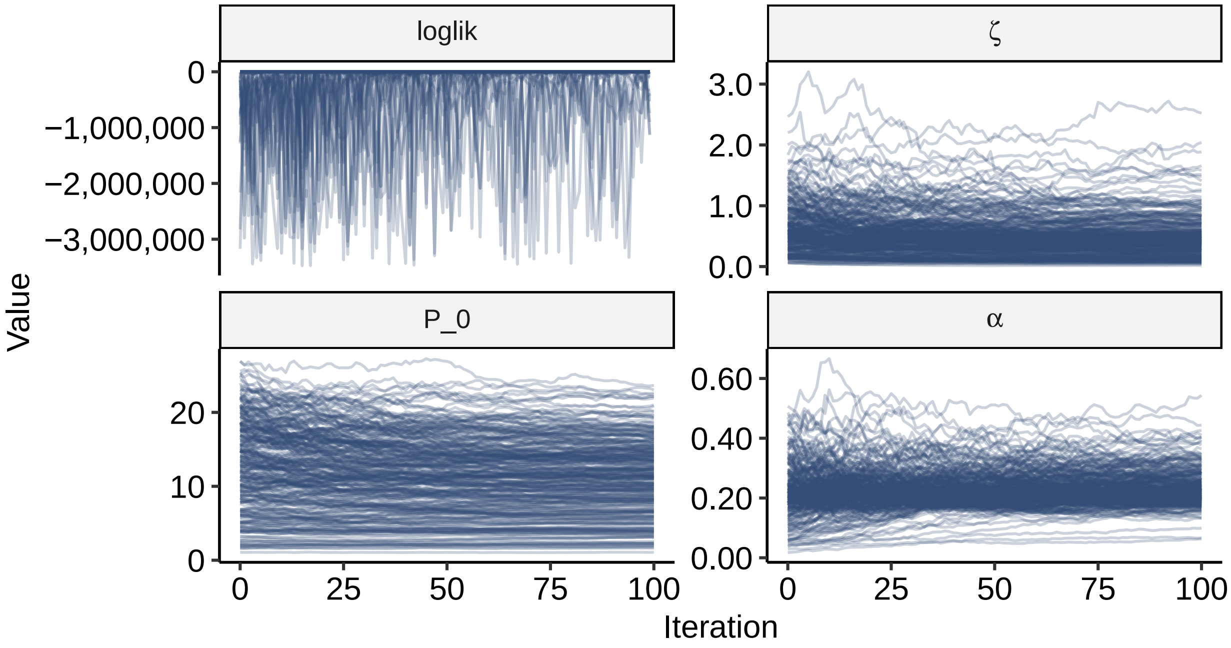

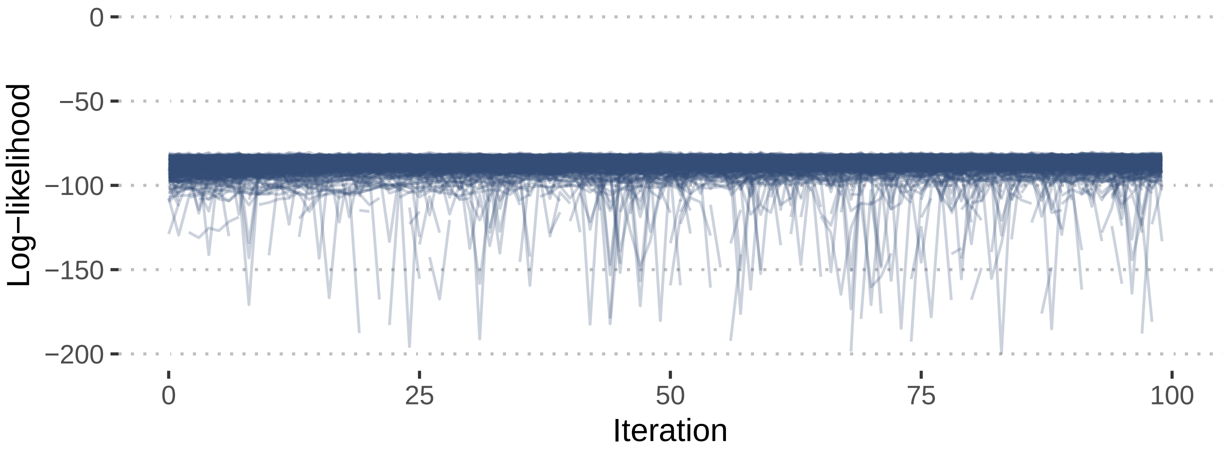

2.2.2 Likelihood estimates

In the graph below, grey dots denote starting points, whereas the other dots are the point estimates obtained from the Iterated Filtering algorithm. We notice that the estimates tend to converge to certain regions of the parameter space.

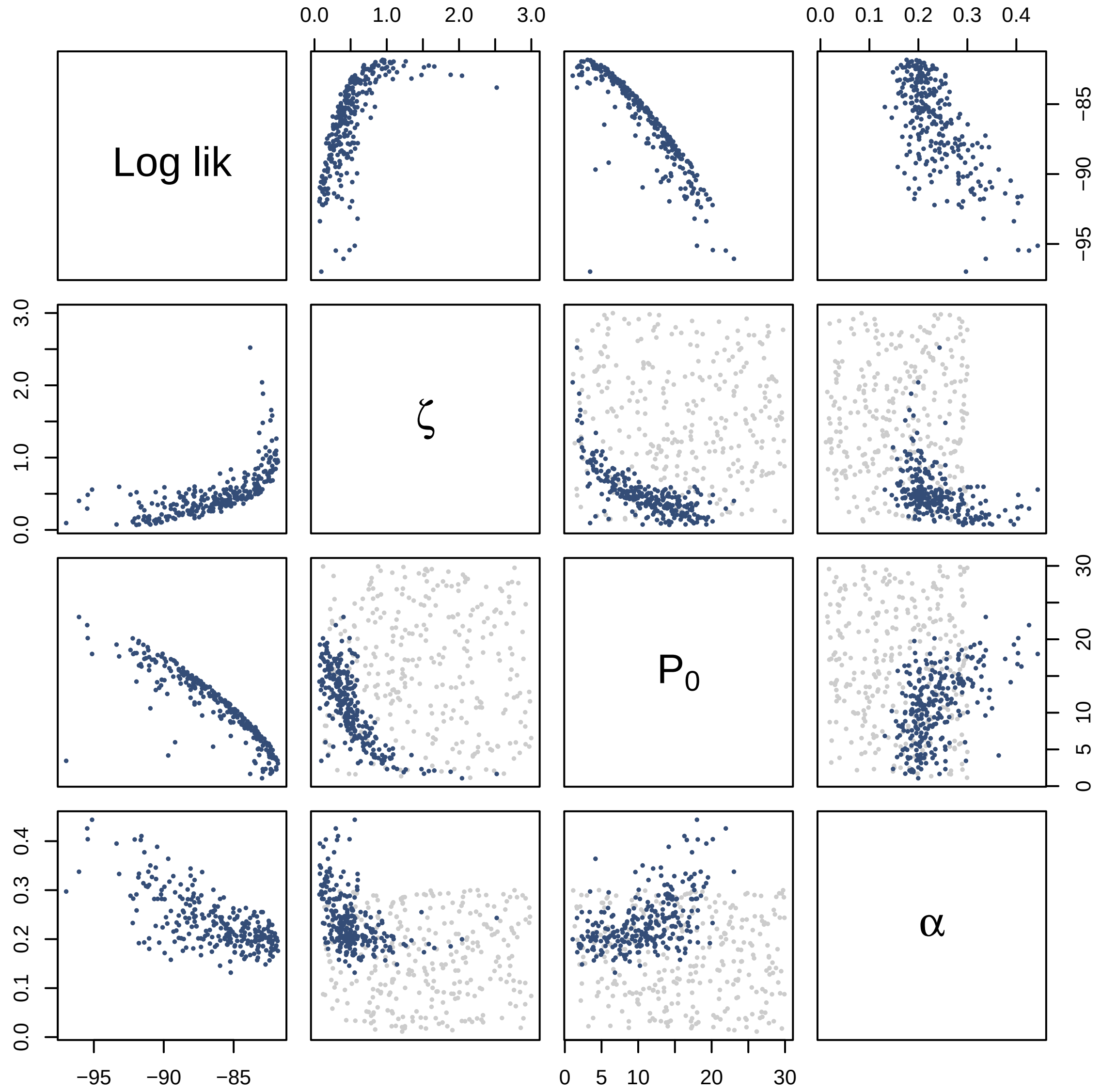

2.3 Profile likelihood

We employ the Profile Likelihood method to estimate confidence intervals. To illustrate the mechanics of this method, we describe each step followed to calculate the initial effective contact rate's (ζ) uncertainty bounds. We repeat this process for the remaining parameters.

2.3.1 ζ - Initial effective contact rate

To begin with, we plot the initial effective contact rate's likelihood surface using the information from the global search to gain insight into the curvature of ζ.

2.3.1.1 Initial likelihood surface

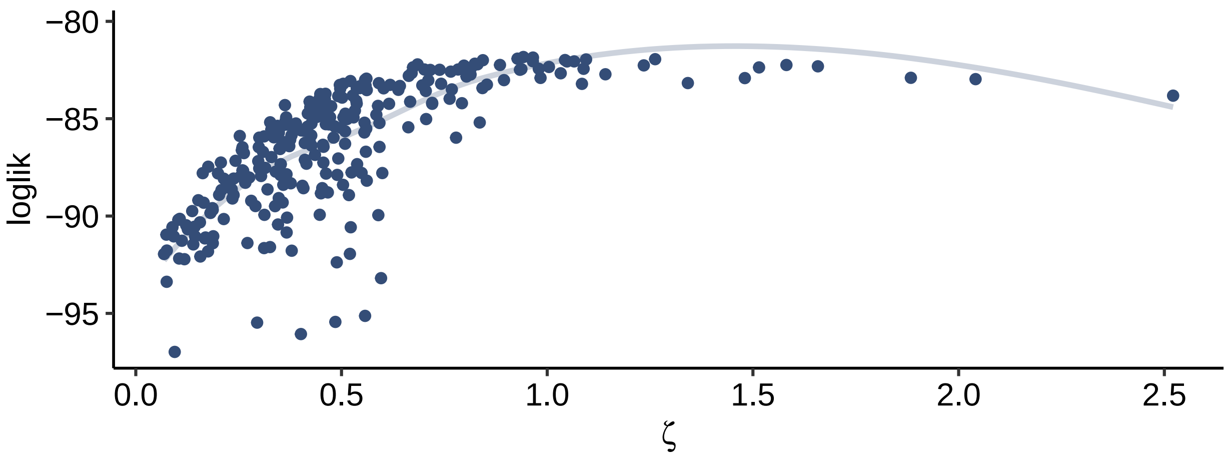

2.3.1.2 Exploration hypercube

We subsequently define a region near the MLE (hypercube) from which we draw several hundreds of samples. The specific number of samples varies according to the complexity of exploring each parameter's space. For ζ, we draw 1,000 samples.

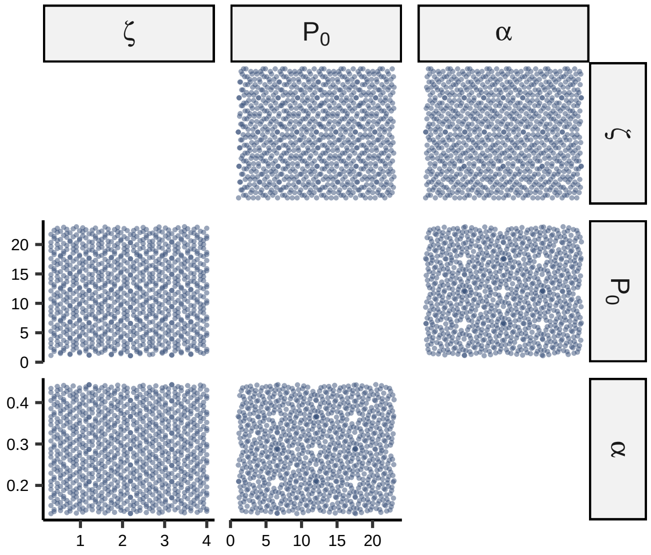

2.3.1.3 Likelihood maximisation

For each sample, we run the Iterated Filtering algorithm. In this case, though, ζ is held constant, whereas the other parameters are subject to stochastic perturbations.

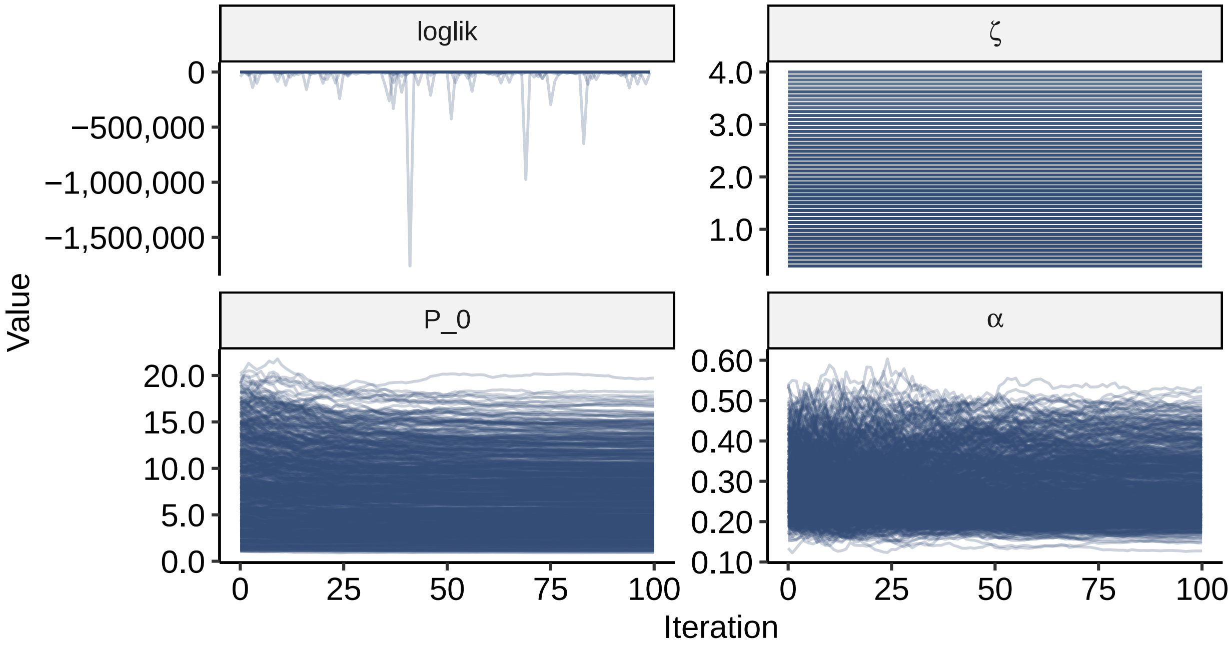

2.3.1.4 Confidence intervals

As with the local and global searches, we estimate via the Particle Filter the likelihood for each of the point estimates obtained from the Iterated Filtering algorithm. From this calculation, we construct the confidence intervals using the Profile Likelihood method and its refined version, the Monte Carlo-adjusted profile. The red dashed line indicates the cut-off at the 95% confidence level.

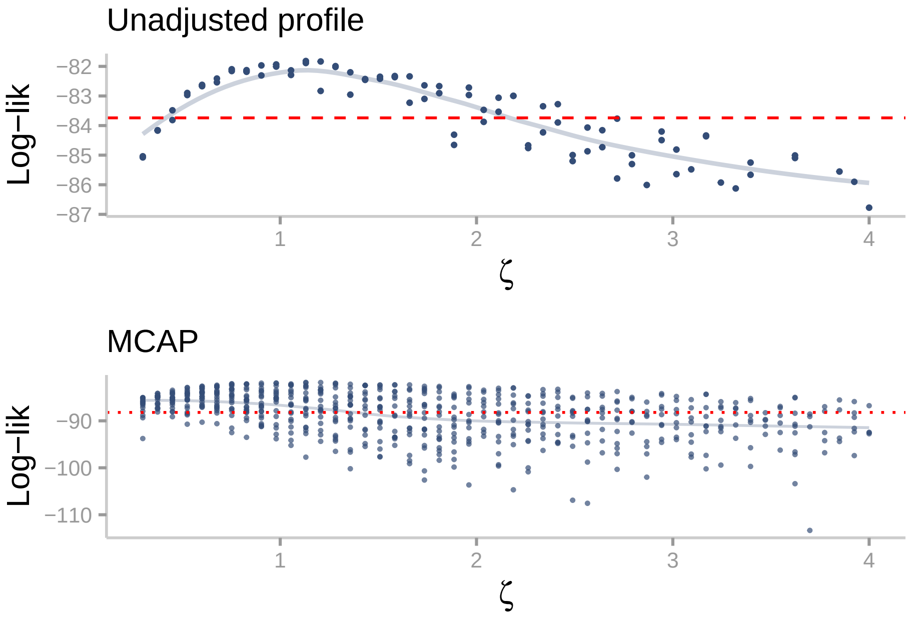

2.3.2 P_0 - Initial number of preclinical individuals

2.3.2.1 Initial likelihood surface

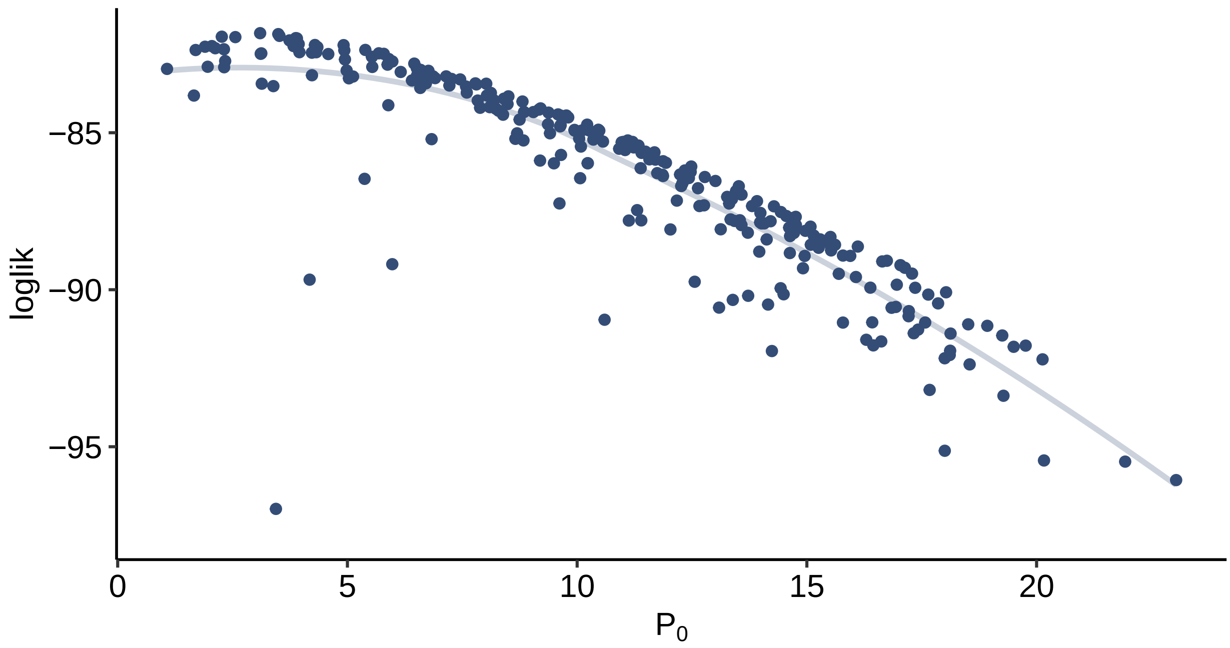

2.3.2.2 Exploration hypercube

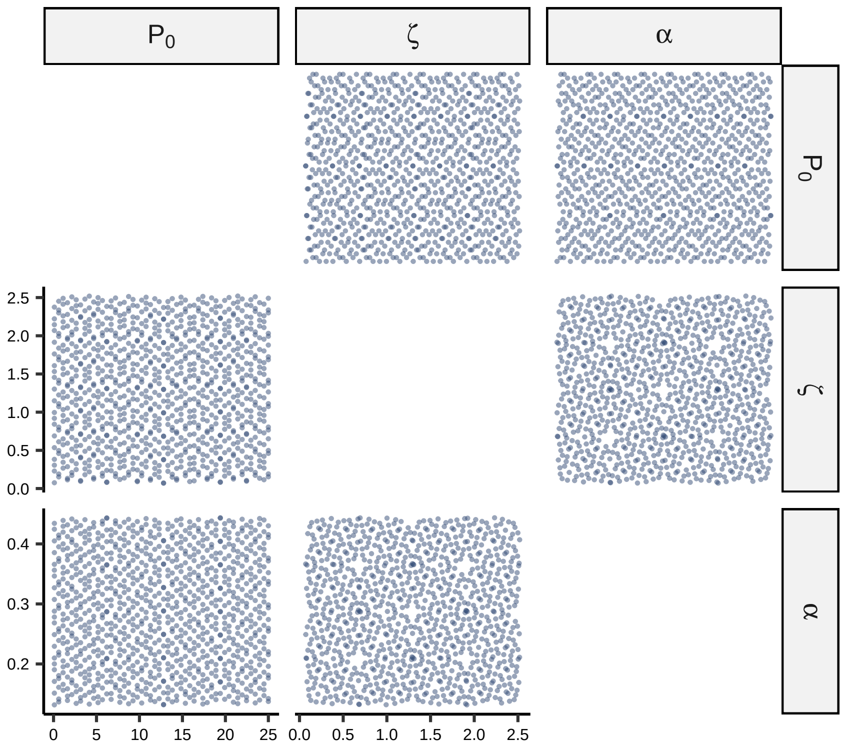

2.3.2.3 Likelihood maximisation

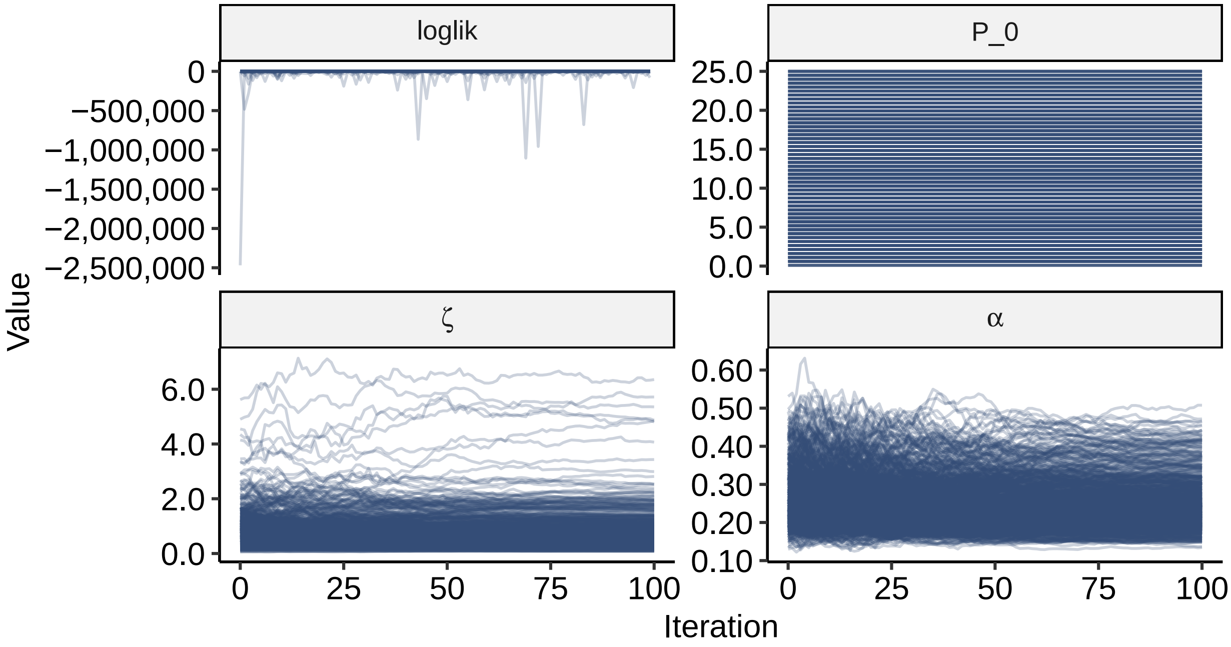

2.3.2.4 Confidence intervals

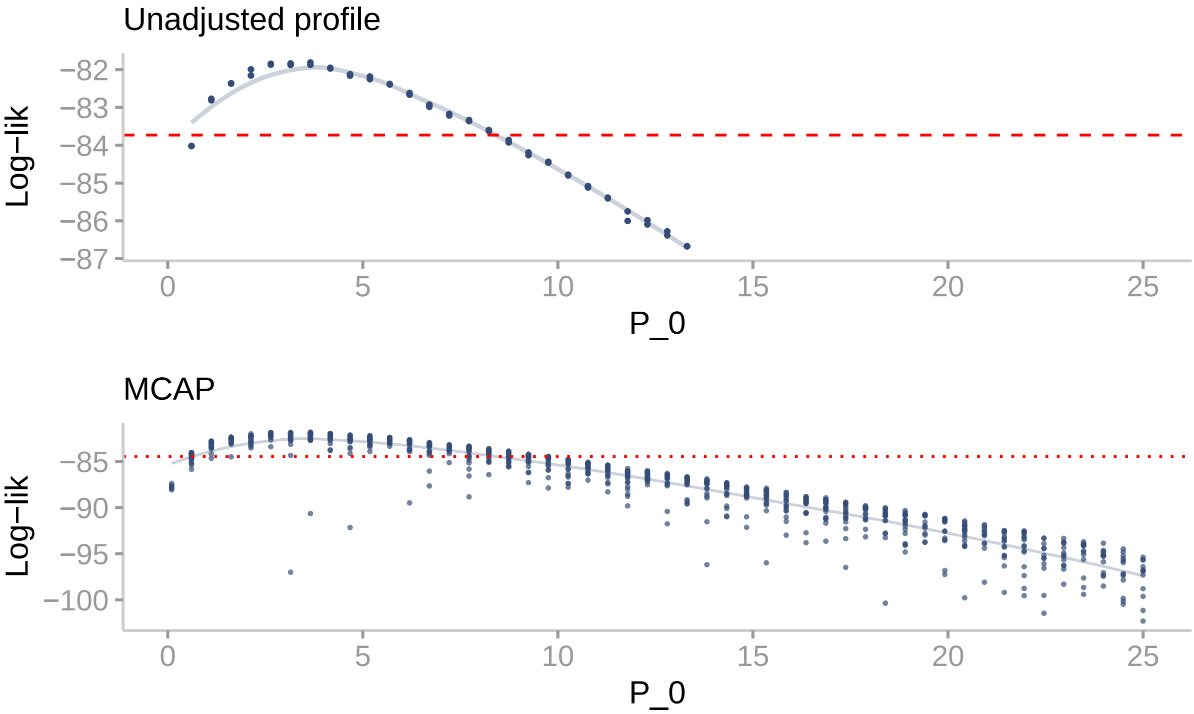

2.3.3 α - Volatility of effective contact rate

2.3.3.1 Initial likelihood surface

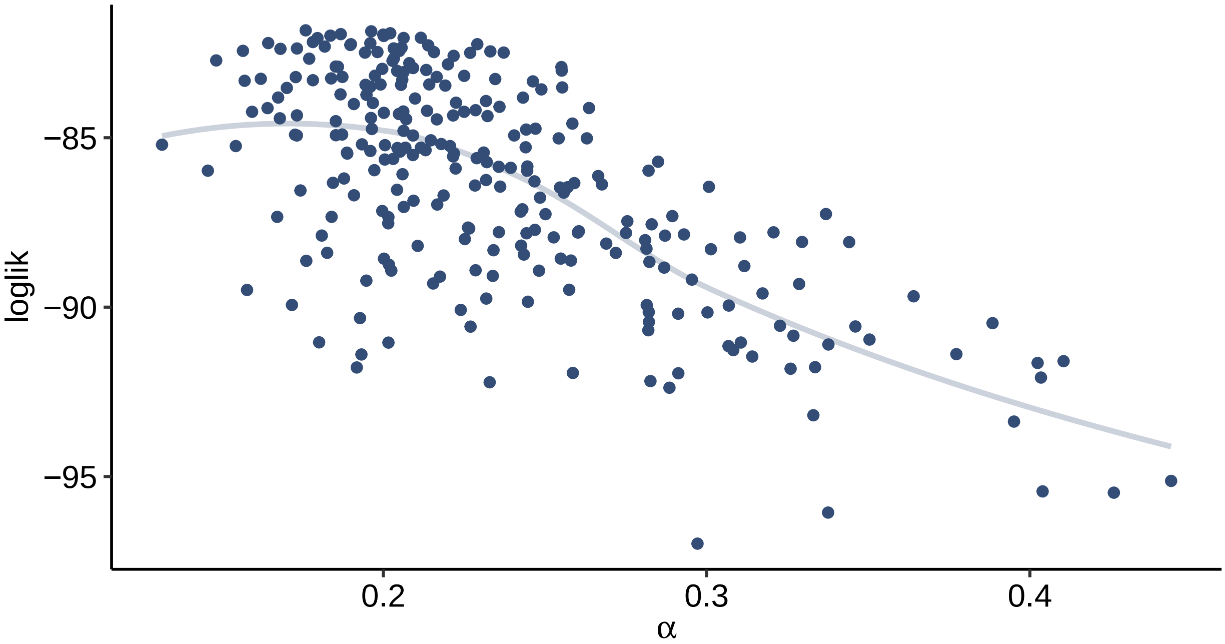

2.3.3.2 Exploration hypercube

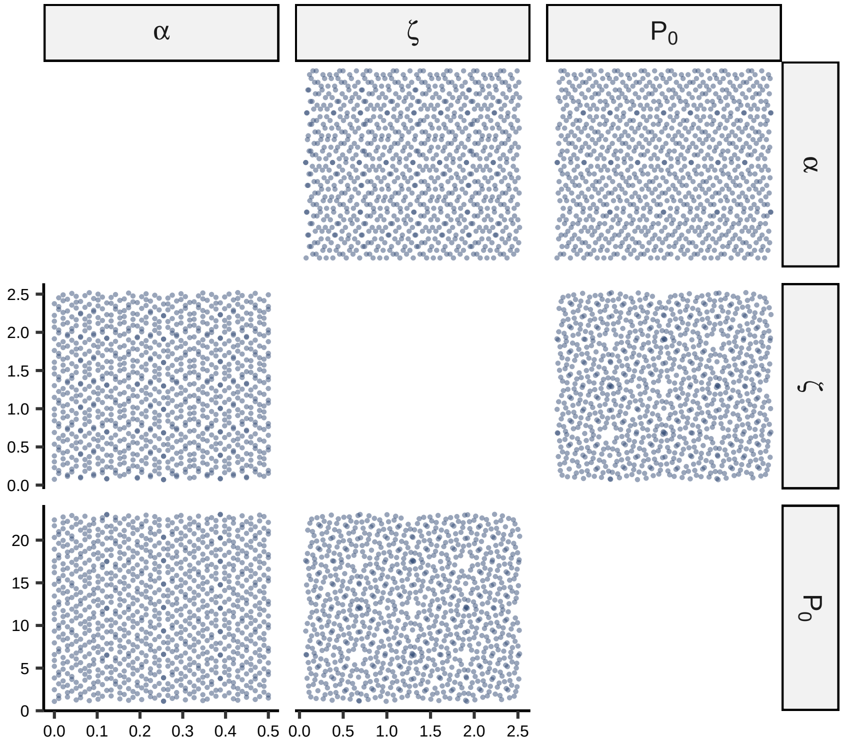

2.3.3.3 Likelihood maximisation

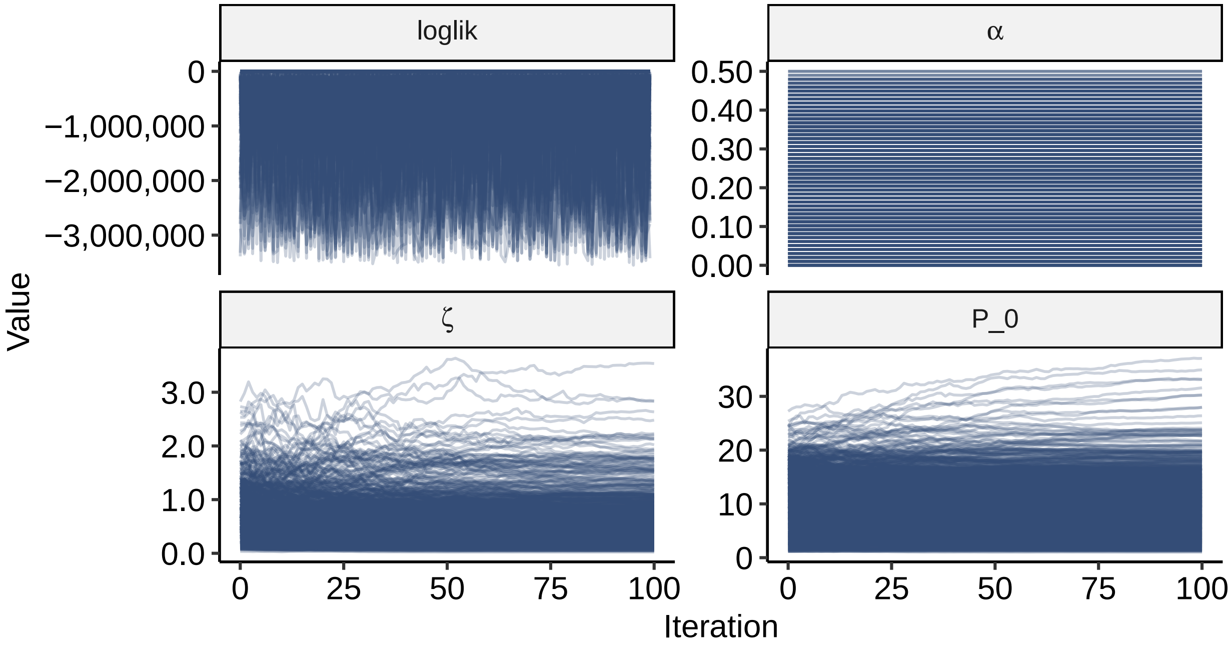

2.3.3.4 Confidence intervals

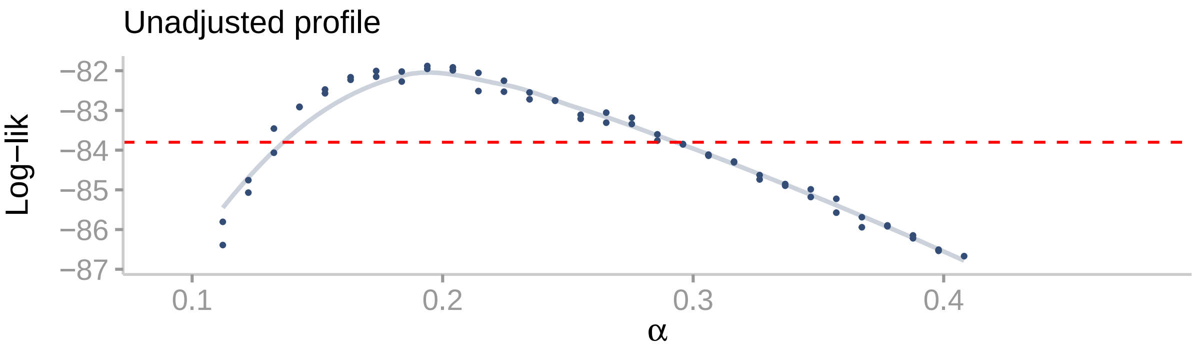

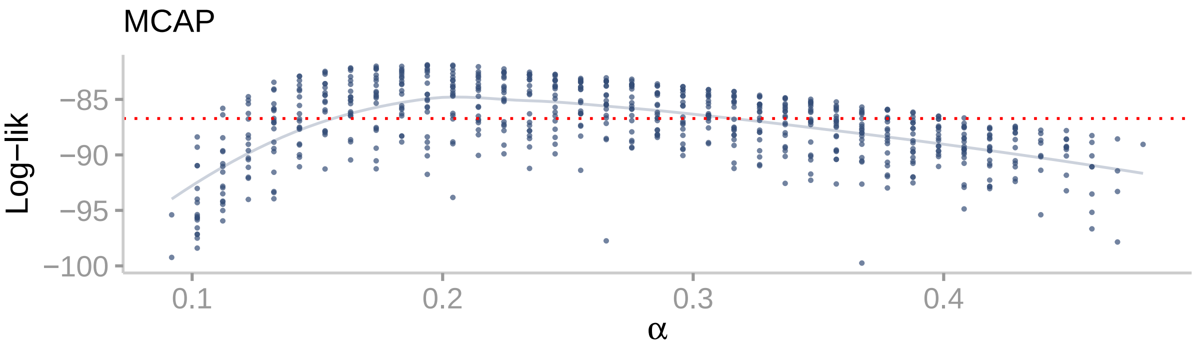

It took approximately 9 hours to compute the inference process (Iterated Filtering + Particle Filter) on this model's fixed parameters.

2.3.4 Estimates

In this section, we present a summary of the estimates obtained from the profile likelihood.

2.3.4.1 From the likelihood surface

We collate all the likelihood estimates from the previous steps into a single database. The resulting likelihood surfaces exhibit quadratic shapes (shown below). We, therefore, assume that these surfaces are approximations of the likelihood profiles. Following this assumption, we estimate each parameter's 95% confidence intervals.

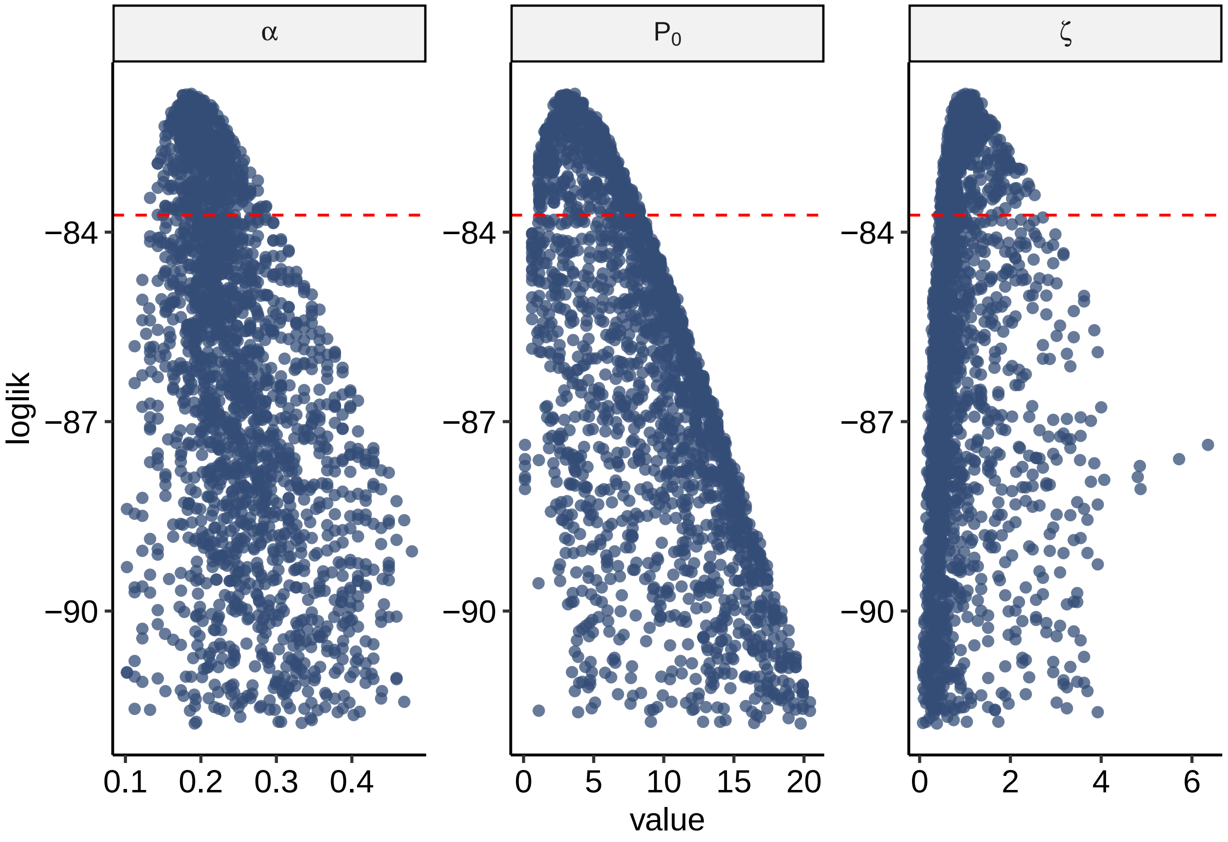

Parameter	MLE	Lower limit	Upper limit
α	0.19	0.13	0.29
$\Re_0$	4.95	2.17	12.40
ζ	1.01	0.44	2.53
P_0	3.66	1.03	8.30

2.3.4.2 From likelihood profiles

Parameter	MLE	Lower limit	Upper limit
α	0.19	0.13	0.29
$\Re_0$	5.53	2.21	11.81
ζ	1.13	0.45	2.41
P_0	3.66	1.12	8.23

2.3.4.3 From MCAP

Parameter	MLE	Lower limit	Upper limit
α	0.21	0.16	0.32
$\Re_0$	1.63	1.47	6.76
ζ	0.33	0.30	1.38
P_0	3.49	0.65	8.40

2.3.4.4 Comparison

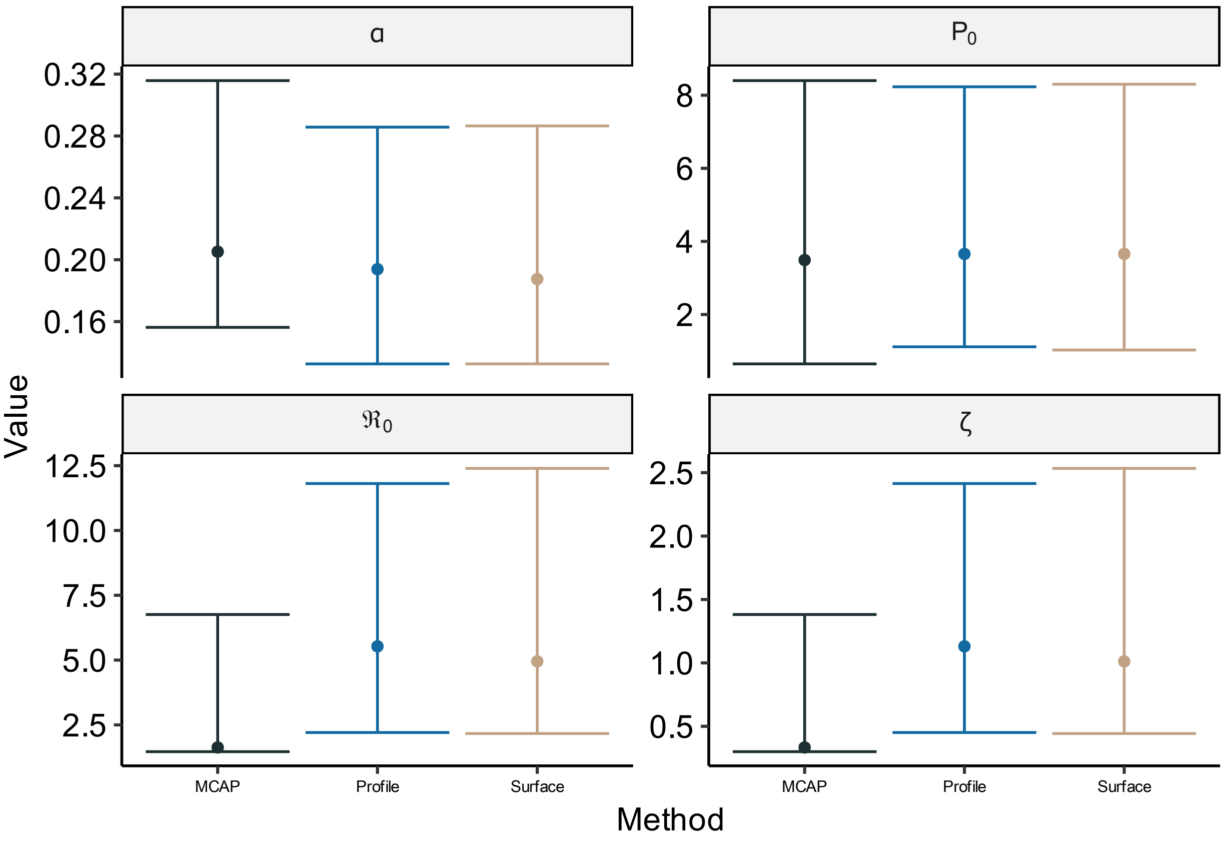

3 Prediction

The reader should recall that we obtain predictions for the latent states from the filtering distribution, which is intractable. To circumvent this difficulty, we use samples to approximate it. We briefly describe such a process.

3.1 Sampling space

First, we define a hypercube near the MLE (neighbourhood).

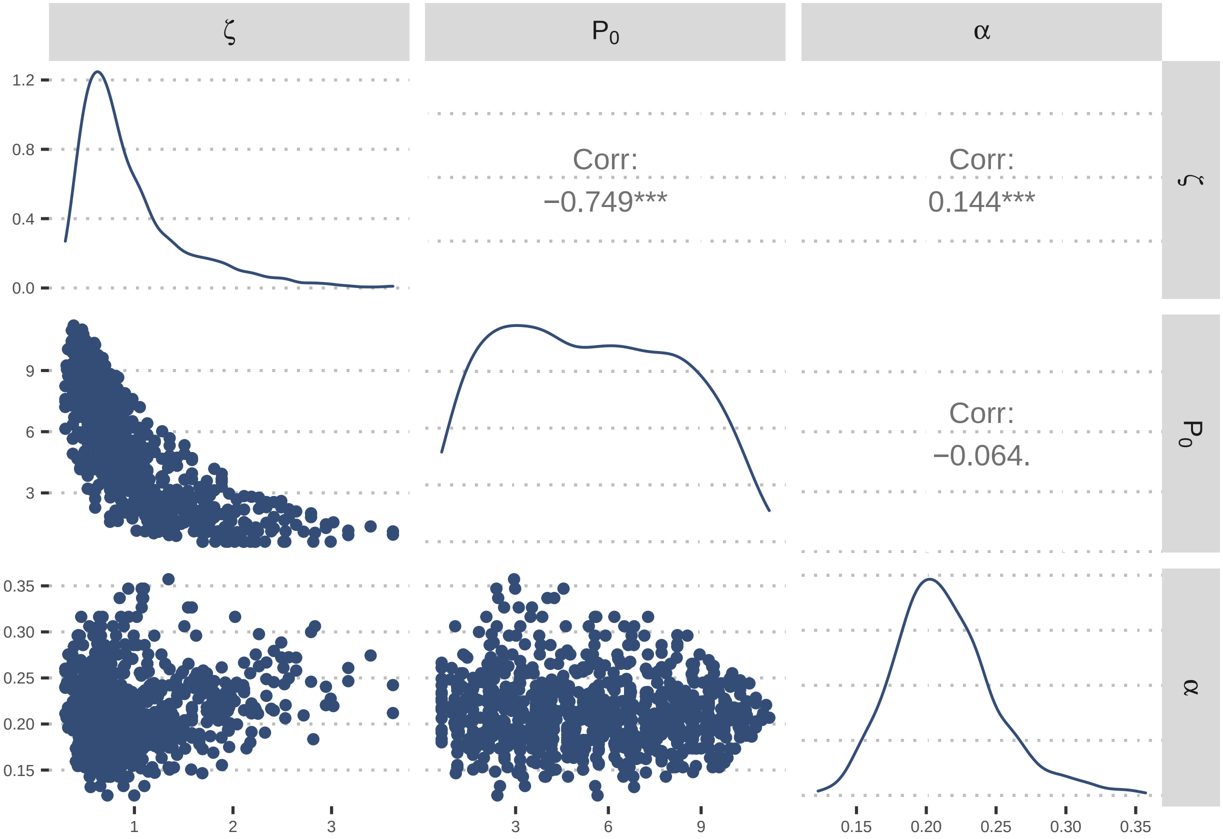

3.2 Draws

Then, we draw 200 samples from such hypercube.

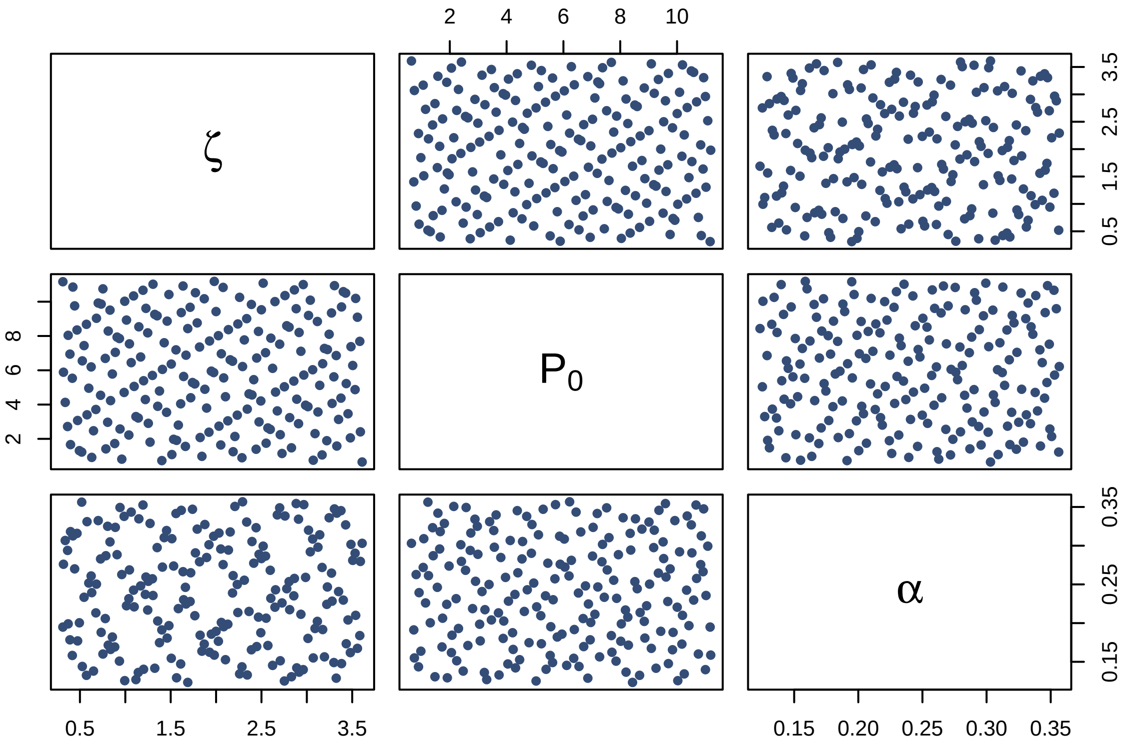

3.3 Hidden states

Finally, we feed the Particle Filter with the samples from the previous step. This method returns a set of draws weighted by its corresponding likelihood to approximate the filtering distribution at each time step.

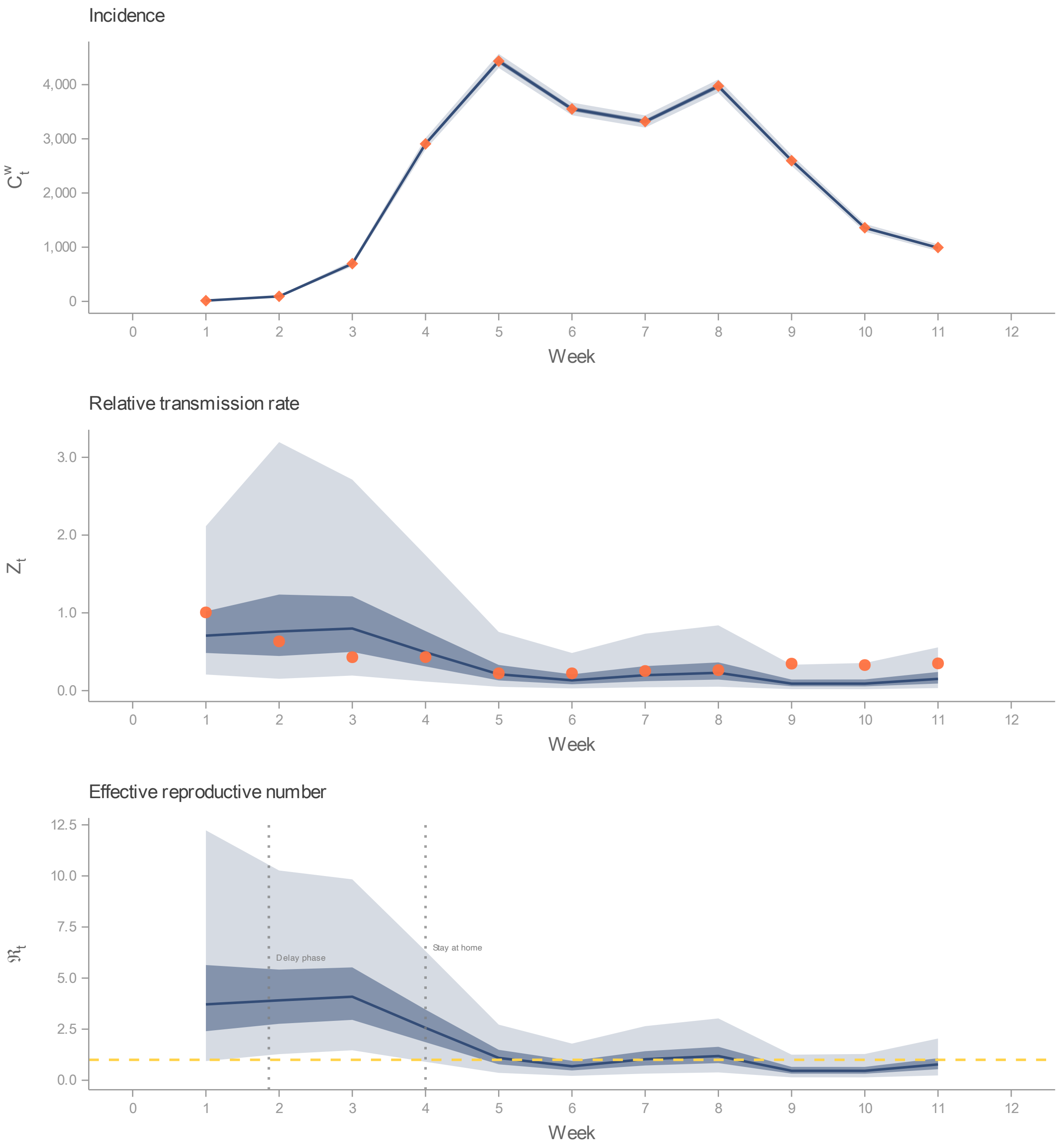

4 Original Computing Environment

```
## R version 4.1.2 (2021-11-01)
## Platform: aarch64-apple-darwin20 (64-bit)
## Running under: macOS Monterey 12.3.1
##
## Matrix products: default
## BLAS:   /Library/Frameworks/R.framework/Versions/4.1-arm64/Resources/lib/libRblas.0.dylib
## LAPACK: /Library/Frameworks/R.framework/Versions/4.1-arm64/Resources/lib/libRlapack.dylib
##
## locale:
## [1] en_US.UTF-8/en_US.UTF-8/en_US.UTF-8/C/en_US.UTF-8/en_US.UTF-8
##
## attached base packages:
## [1] parallel  stats     graphics  grDevices utils     datasets  methods
## [8] base
##
## other attached packages:
##  [1] scales_1.1.1      patchwork_1.1.1   ggrepel_0.9.1     ggpubr_0.4.0
##  [5] ggalt_0.4.0       GGally_2.1.2      ggplot2_3.3.5     extraDistr_1.9.1
##  [9] tidyr_1.1.4       tictoc_1.0.1      tibble_3.1.6      stringr_1.4.0
## [13] readxl_1.3.1      readr_2.1.1       purrr_0.3.4       pomp_3.6
## [17] lubridate_1.8.0   imputeTS_3.2      dplyr_1.0.8       doRNG_1.8.2
## [21] rngtools_1.5.2    doParallel_1.0.16 iterators_1.0.13  foreach_1.5.1
##
## loaded via a namespace (and not attached):
##  [1] nlme_3.1-153       ggtext_0.1.1       xts_0.12.1         bit64_4.0.5
##  [5] ash_1.0-15         RColorBrewer_1.1-2 backports_1.4.1    tools_4.1.2
##  [9] utf8_1.2.2         R6_2.5.1           KernSmooth_2.23-20 mgcv_1.8-38
## [13] colorspace_2.0-2   nnet_7.3-16        withr_2.4.3        tidyselect_1.1.1
## [17] bit_4.0.4          curl_4.3.2         compiler_4.1.2     extrafontdb_1.0
## [21] cli_3.3.0          xml2_1.3.3         labeling_0.4.2     tseries_0.10-49
## [25] lmtest_0.9-39      fracdiff_1.5-1     proj4_1.0-10.1     mvtnorm_1.1-3
## [29] quadprog_1.5-8     digest_0.6.29      rmarkdown_2.11     pkgconfig_2.0.3
## [33] htmltools_0.5.2    extrafont_0.17     highr_0.9          fastmap_1.1.0
## [37] maps_3.4.0         rlang_1.0.2        TTR_0.24.3         rstudioapi_0.13
## [41] quantmod_0.4.18    farver_2.1.0       generics_0.1.1     zoo_1.8-9
## [45] vroom_1.5.7        car_3.0-12         magrittr_2.0.3     Matrix_1.3-4
## [49] Rcpp_1.0.7         munsell_0.5.0      fansi_0.5.0        abind_1.4-5
## [53] lifecycle_1.0.1    stringi_1.7.6      forecast_8.15      yaml_2.2.1
## [57] carData_3.0-4      MASS_7.3-54        plyr_1.8.6         grid_4.1.2
## [61] crayon_1.4.2       lattice_0.20-45    splines_4.1.2      gridtext_0.1.4
## [65] hms_1.1.1          knitr_1.37         pillar_1.6.4       ggsignif_0.6.3
## [69] reshape2_1.4.4     codetools_0.2-18   urca_1.3-0         glue_1.6.2
## [73] evaluate_0.14      stinepack_1.4      deSolve_1.30       vctrs_0.4.1
## [77] tzdb_0.2.0         Rttf2pt1_1.3.9     cellranger_1.1.0   gtable_0.3.0
## [81] reshape_0.8.8      xfun_0.29          broom_0.7.10       rstatix_0.7.0
## [85] coda_0.19-4        timeDate_3043.102  ellipsis_0.3.2
```
