# Supplementary material for: Inferring the effective reproductive number from deterministic and semi-deterministic compartmental models using incidence and mobility data
Source: PLoS Comput Biol. 2022 Jun 27;18(6):e1010206. doi: 10.1371/journal.pcbi.1010206 (PMC9269962; doi:10.1371/journal.pcbi.1010206)
Supplement: S6 Text — (PDF) [file pcbi.1010206.s006.pdf]

S6 Appendix

This appendix illustrates the inference process carried out on DGP2. Its process model consists of an SEIR-type formulation whose relative effective contact rate is described by a Cox-Ingersoll-Ross structure. Moreover, DGP2's measurement model assumes that weekly incidence counts are distributed according to the **Poisson distribution**, and that **mobility data is a proxy** measurement for the relative effective contact rate. In particular, we apply *Iterated Filtering* and the *Particle Filter* to obtain estimates (via samples) for the effective reproductive number and other parameters that explain Ireland's first wave of COVID-19 in 2020.

Contents

1 Structure **2**
- 1.1 Process model . . . 2
- 1.2 Measurement model . . . 2
- 1.3 Unmodelled predictors . . . 3
- 1.4 Unknown parameters . . . 3

2 Parameter inference **4**
- 2.1 Local search . . . 4
  - 2.1.1 Likelihood maximisation . . . 4
  - 2.1.2 Likelihood estimates . . . 5
- 2.2 Global search . . . 6
  - 2.2.1 Likelihood maximisation . . . 6
  - 2.2.2 Likelihood estimates . . . 7
- 2.3 Profile likelihood . . . 8
  - 2.3.1 ζ - Initial effective contact rate . . . 8
  - 2.3.2 P_0 - Initial number of preclinical individuals . . . 11
  - 2.3.3 α - Volatility of effective contact rate . . . 12
  - 2.3.4 τ - Variance of the measured transmission rate . . . 13
  - 2.3.5 ν - Adjustment speed . . . 14
  - 2.3.6 υ - Long-term goal . . . 15
  - 2.3.7 Estimates . . . 16

3 Prediction **18**
- 3.1 Sampling space . . . 18
- 3.2 Draws . . . 19
- 3.3 Cutoff . . . 20
- 3.4 Hidden states . . . 23

4 Original Computing Environment **24**

1 Structure

1.1 Process model

$$\frac{dS}{dt} = -S_t \lambda_t \tag{1}$$

$$\frac{dE}{dt} = S_t \lambda_t - \sigma E_t \tag{2}$$

$$\frac{dP}{dt} = \omega \sigma E_t - \eta P_t \tag{3}$$

$$\frac{dI}{dt} = \eta P_t - \gamma I_t \tag{4}$$

$$\frac{dA}{dt} = (1 - \omega) \sigma E_t - \kappa A_t \tag{5}$$

$$\frac{dR}{dt} = \kappa A_t + \gamma I_t \tag{6}$$

$$\lambda_t = \frac{\beta_t (I_t + P_t + \mu A_t)}{N_t} \tag{7}$$

$$\beta_t = \zeta Z_t \tag{8}$$

$$\frac{dZ}{dt} = \nu(\upsilon - Z_t) + \sqrt{\alpha} Z_t dW \tag{9}$$

$$dW \sim Normal(0, \sqrt{dt}) \tag{10}$$

1.2 Measurement model

$$\frac{dC}{dt} = \eta P_t - C_t \delta(t \bmod 7) \tag{11}$$

$$y_w^1 \sim Pois(C_t) \tag{12}$$

$$y_w^2 \sim Normal(Z_t, \tau) \tag{13}$$

1.3 Unmodelled predictors

Name	Symbol	Value	Units	Source
Incubation period	σ^{-1}	3	Days	Davies (2020)
Duration of preclinical infectiousness	η^{-1}	2.1	Days	Davies (2020)
Duration of clinical infectiousness	γ^{-1}	2.9	Days	Davies (2020)
Clinical fraction	ω	0.7	Unitless	HPSC (2020)
Asymptomatic infectious period	κ^{-1}	5	Days	Davies (2020)
Population	N_0	4937796	People	United Nations (2019)
Relative infectiousness	μ	0.5	Unitless	Davies (2020)
Initial susceptible	S_0	$N_0 - P_0$	People	Assumption
Initial exposed	E_0	0	People	Assumption
Initial clinical infectious	I_0	0	People	Assumption
Initial recovered	R_0	0	People	Assumption
Initial subclinical infectious	A_0	0	People	Assumption
Initial reported cases	C_0	0	People	By definition
Initial mobility effect	Z_0	1	Unitless	By definition

1.4 Unknown parameters

Name	Symbol	Units
Initial effective contact rate	ζ	People / day
Volatility of effective contact rate	α	Unitless
Variance of the measured transmission rate	τ	Unitless
Initial preclinical infectious	P_0	People
Long-term goal	υ	Unitless
Adjustment speed	ν	day^{-1}

2 Parameter inference

2.1 Local search

We start the inference process with a preliminary test. Specifically, we verify that Iterated Filtering algorithm, applied to this DGP (DGP2) and data, converges to regions of high likelihood.

2.1.1 Likelihood maximisation

Accordingly, from a single point in the parameter space, we search for the Maximum Likelihood Estimate (MLE) via Iterated Filtering. We repeat this process **twenty** times.

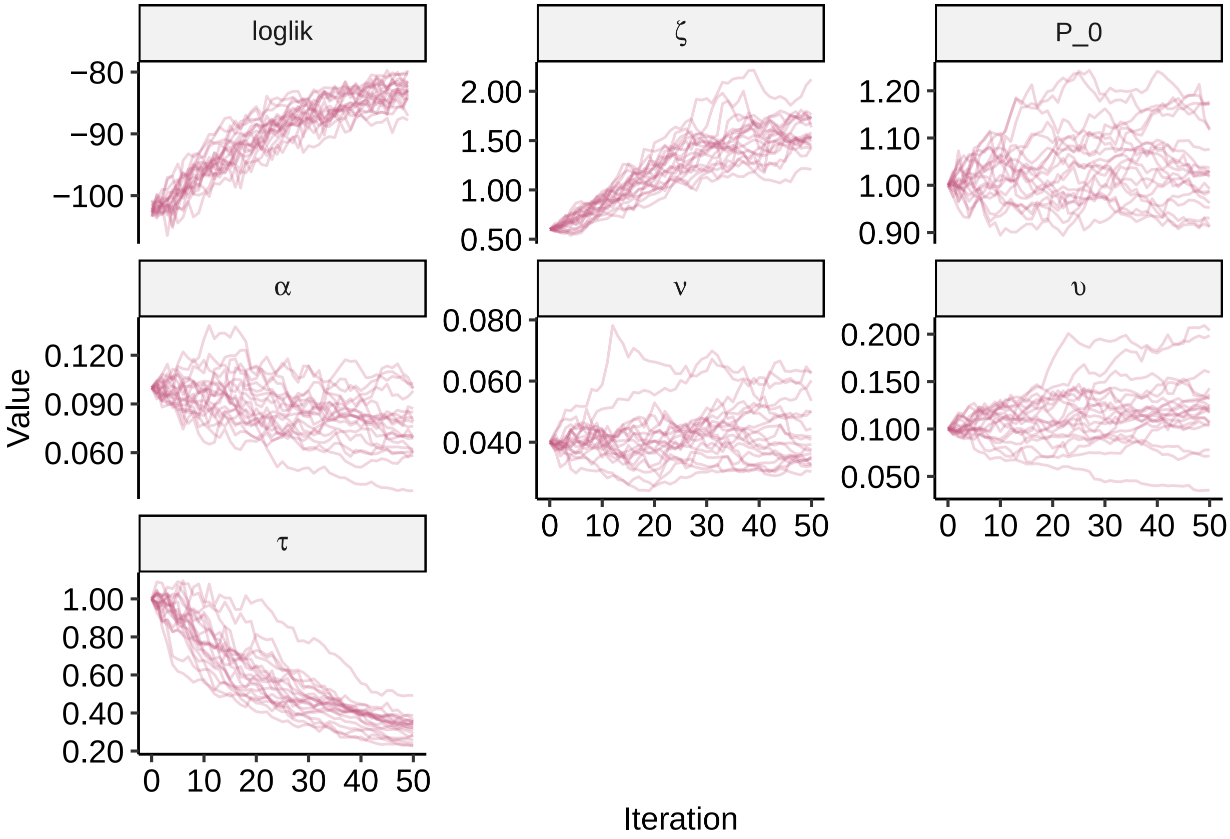

2.1.2 Likelihood estimates

The likelihood estimates obtained from the Iterated Filtering algorithm are merely an approximation to the actual values at those points. This difference occurs for two reasons: the Iterated Filtering algorithm is run with fewer particles than are needed for a good likelihood evaluation; 2) the stochastic perturbations applied to the inferred parameters at each iteration. Consequently, it is necessary to run the Particle Filter to obtain reliable likelihood estimates. Specifically, we use the values from each run's final filtering iteration as inputs to the Particle Filter.

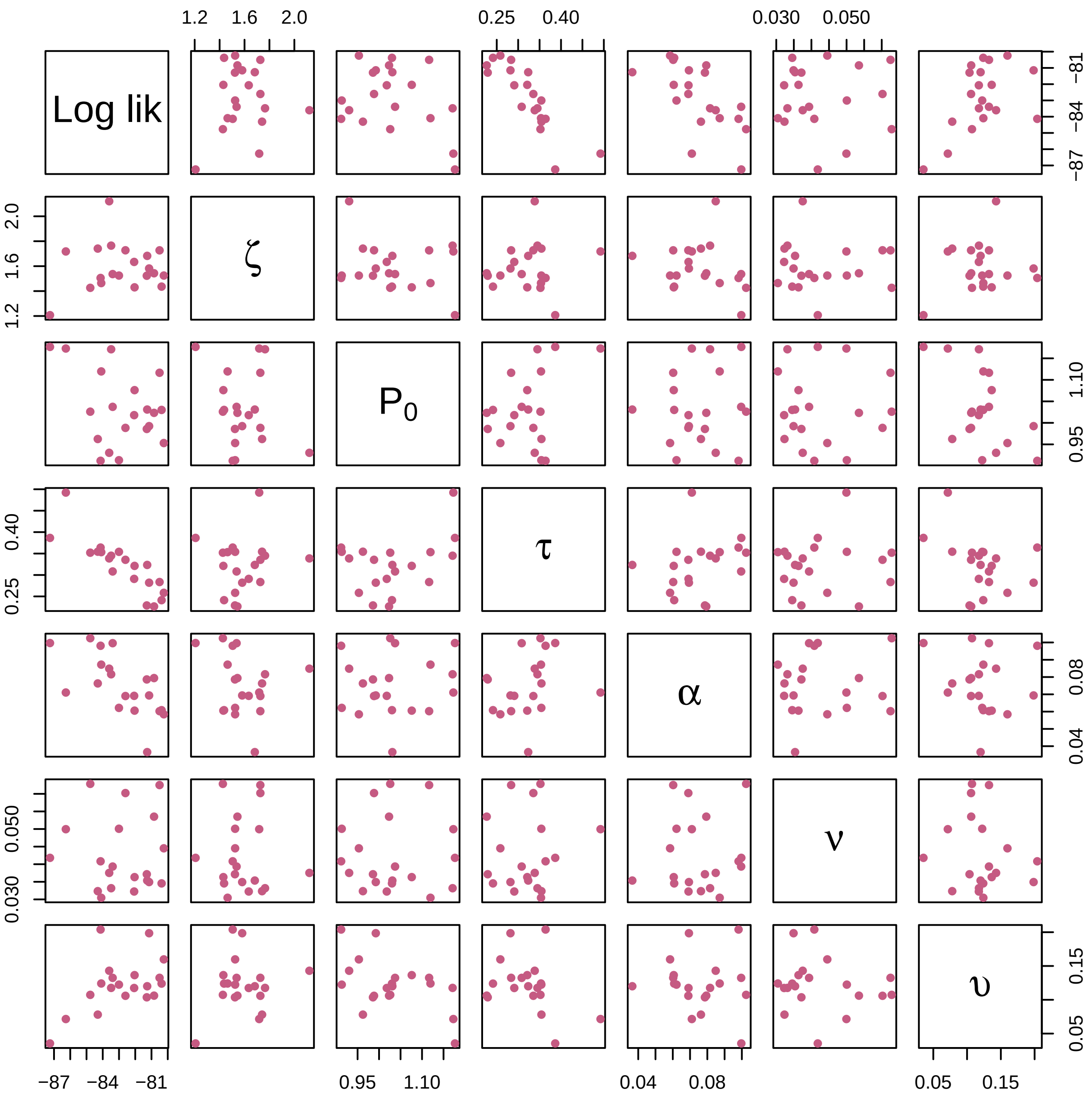

2.2 Global search

In this step, we follow a similar process described in Section 2.1, but this time increasing the number of starting points (300) and filtering iterations. Also, there is only one run for each starting point (in contrast with the 20 runs in Section 2.1). We refer to this step as *global search*, whose purpose is to construct a likelihood surface that allows us to identify regions of high plausibility.

2.2.1 Likelihood maximisation

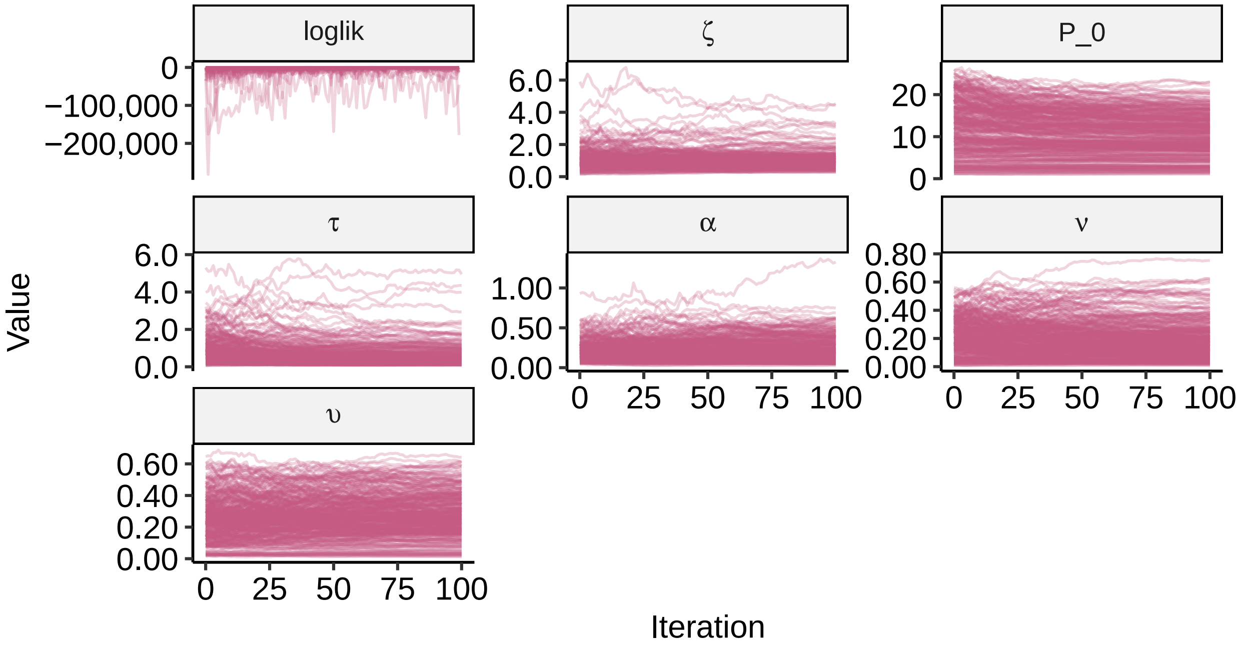

Given the random-walk behaviour embedded in the iterated filtering algorithm, some iterations wildly diverge from regions of high likelihood, distorting the log-lik trace plot (shown above). For this reason, we zoom in to the traces that reached convergence.

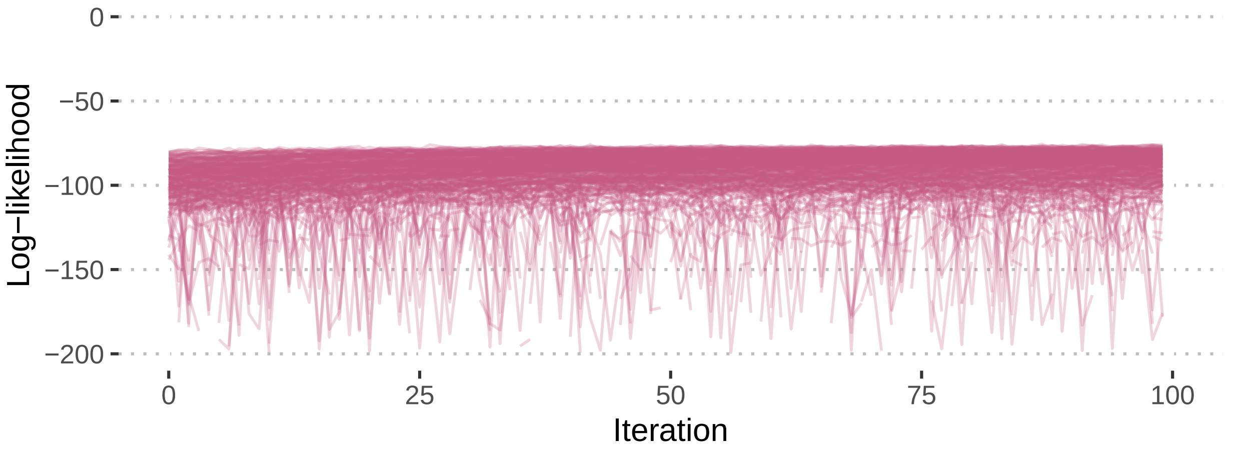

2.2.2 Likelihood estimates

In the graph below, grey dots denote starting points, whereas the other dots are the point estimates obtained from the Iterated Filtering algorithm. We can notice that the estimates tend to converge to certain regions of the parameter space.

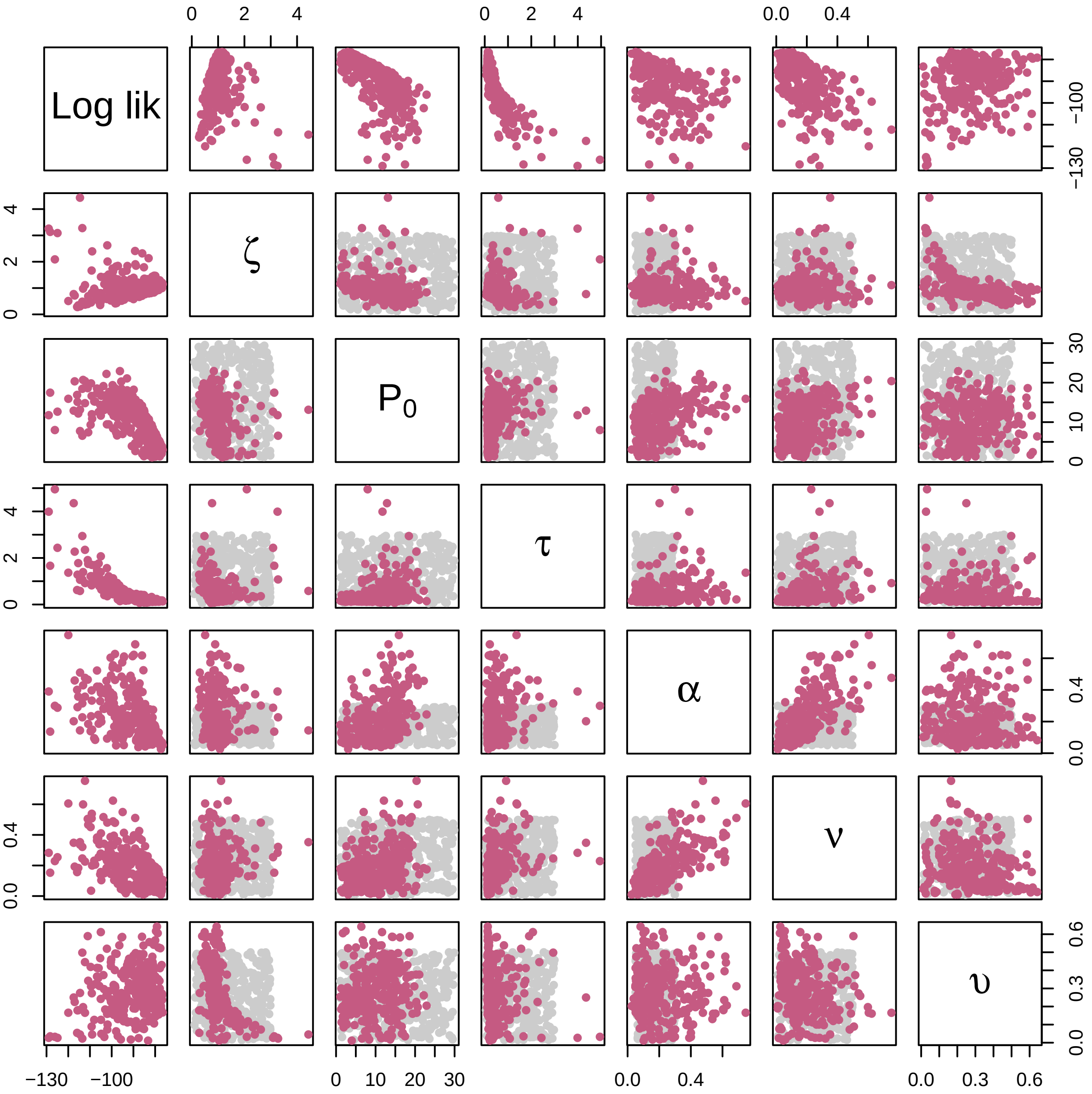

2.3 Profile likelihood

As with DGP1, we employ the profile likelihood method to estimate DGP2's confidence intervals. See Appendix 2d for the description of each step to obtain the uncertainty bounds. Here, we only present the results. Notice that we only show the likelihood maximisation (via iterated filtering) of the initial effective contact rate (ζ). However, this process is applied to all the parameters (not shown).

2.3.1 ζ - Initial effective contact rate

2.3.1.1 Initial likelihood surface

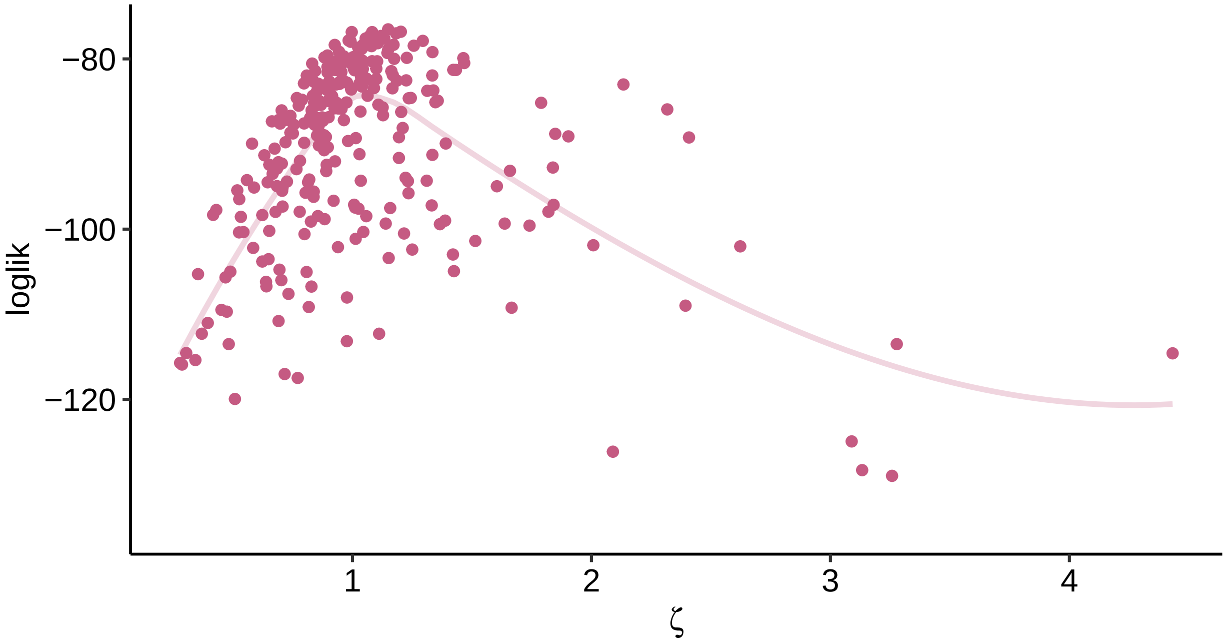

2.3.1.2 Exploration hypercube

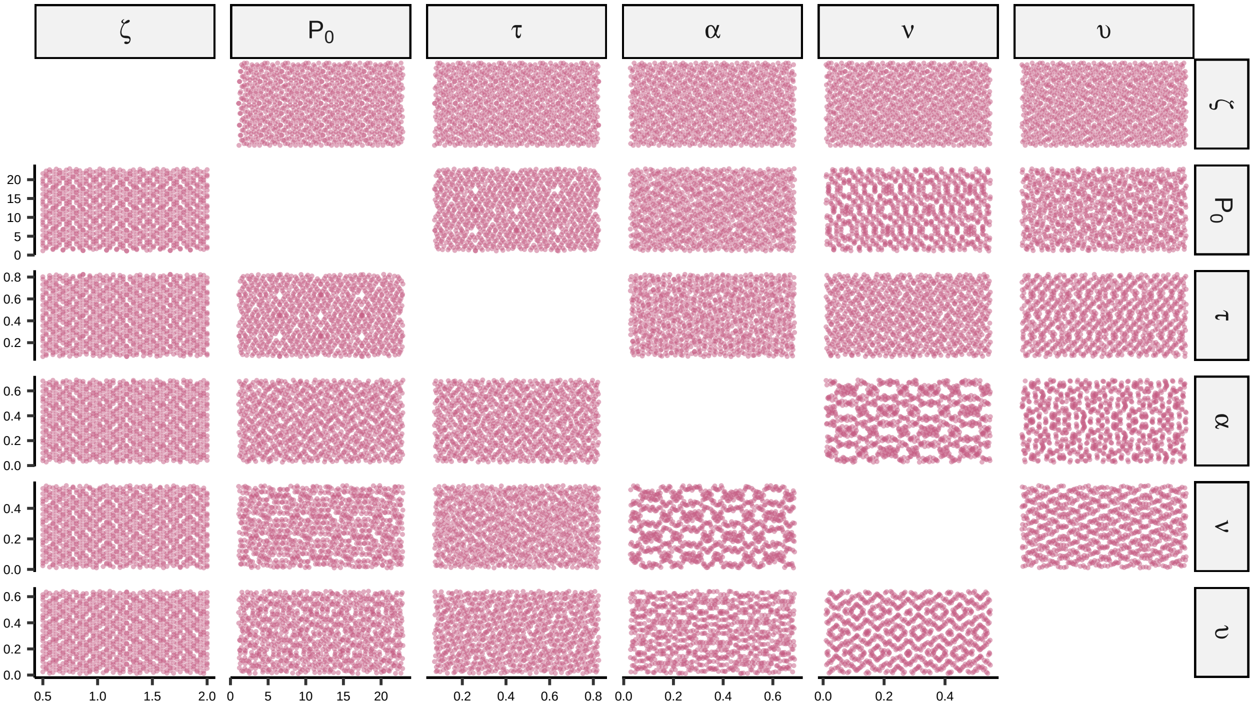

2.3.1.3 Likelihood maximisation

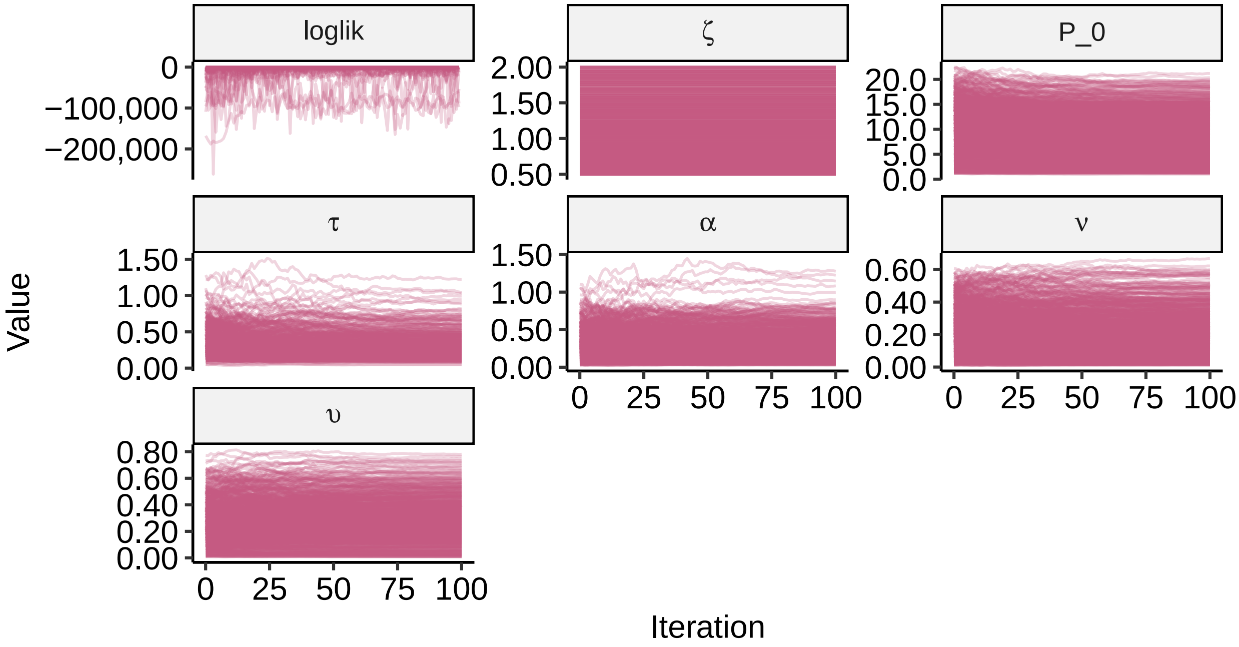

2.3.1.4 Confidence intervals

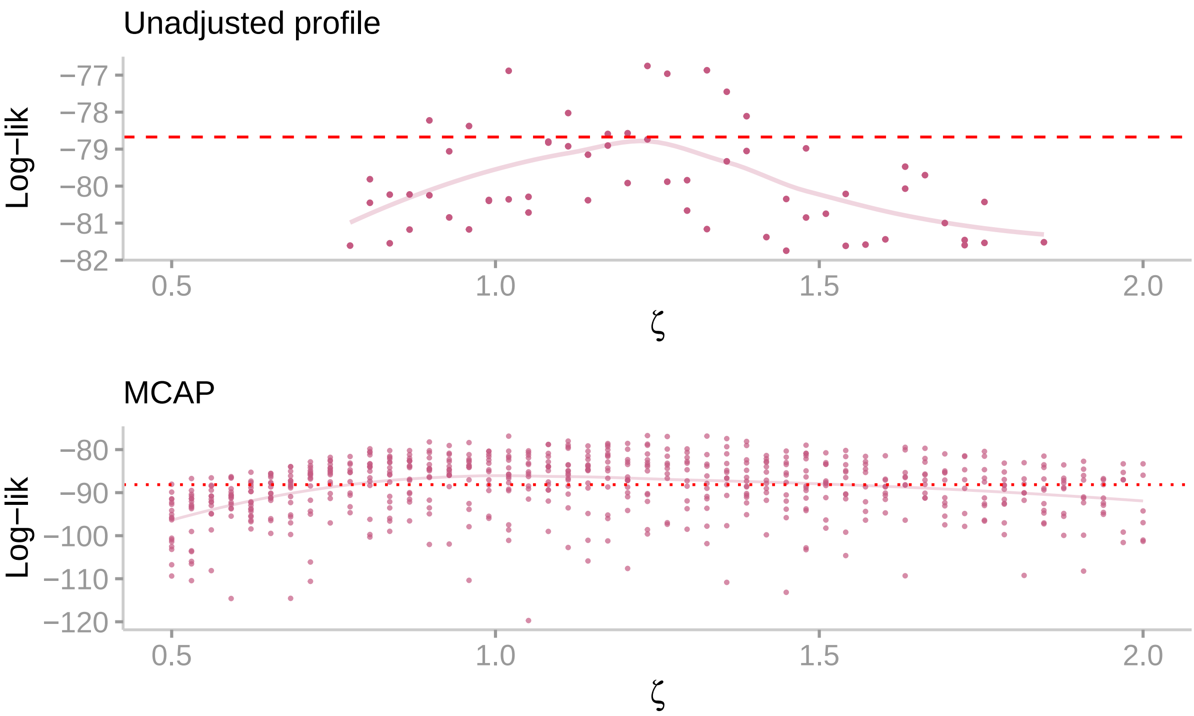

2.3.2 P_0 - Initial number of preclinical individuals

2.3.2.1 Initial likelihood surface

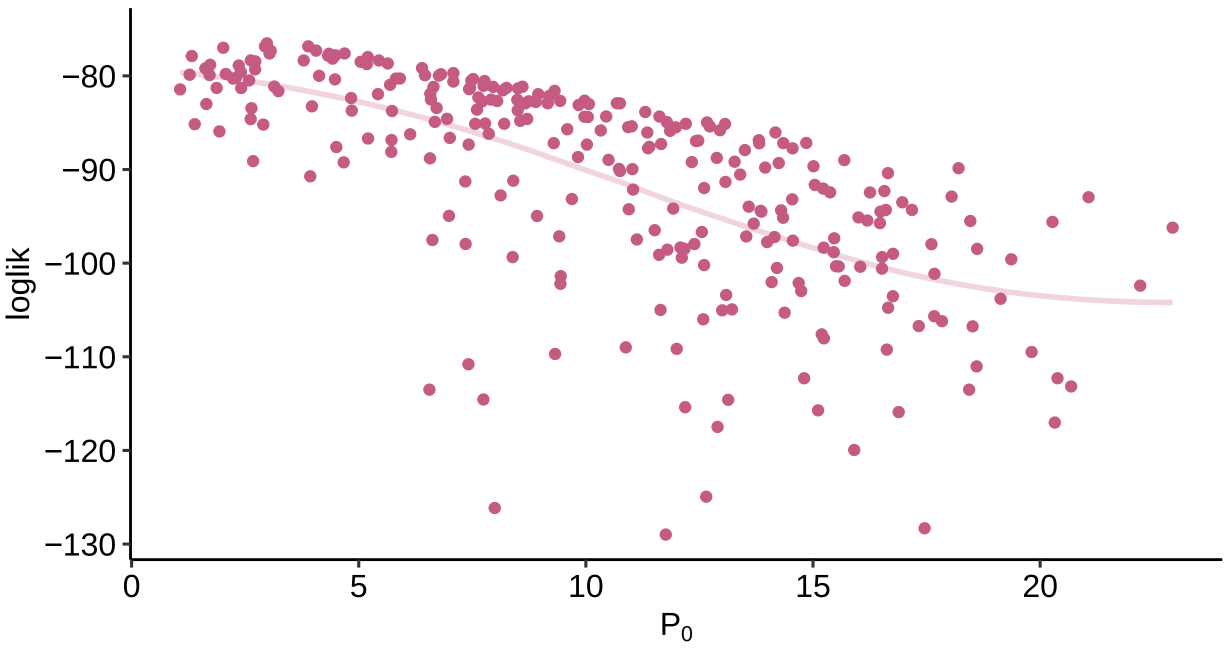

2.3.2.2 Confidence intervals

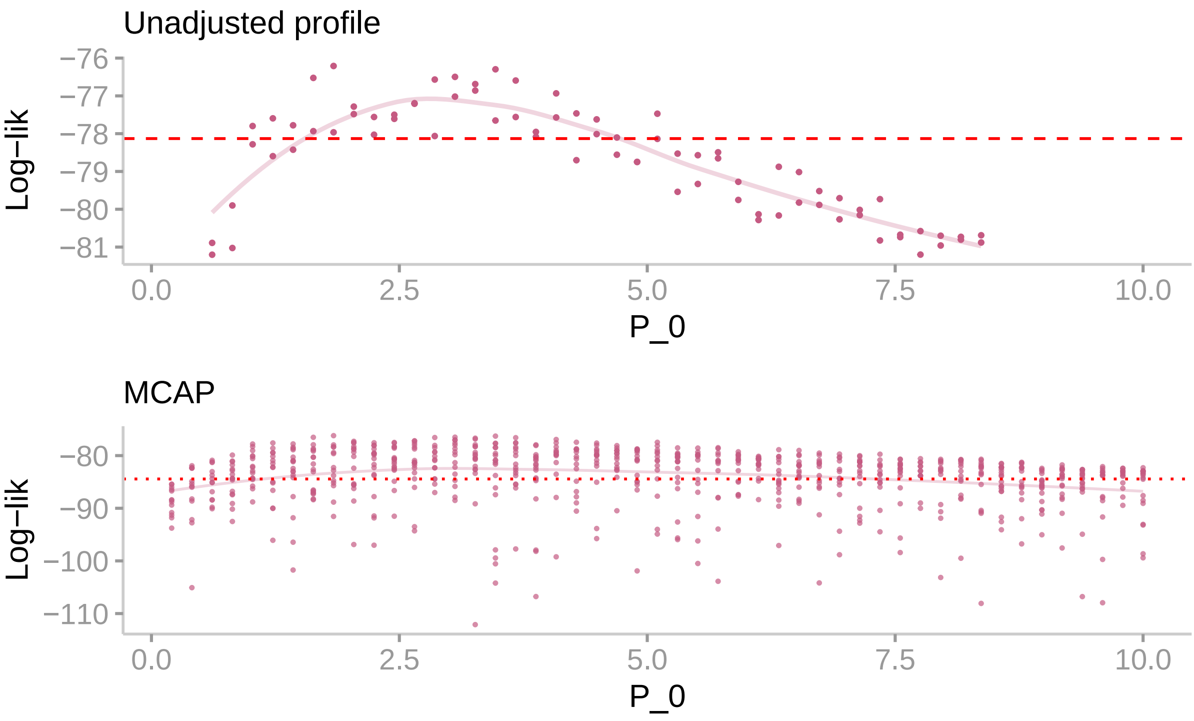

2.3.3 α - Volatility of effective contact rate

2.3.3.1 Initial likelihood surface

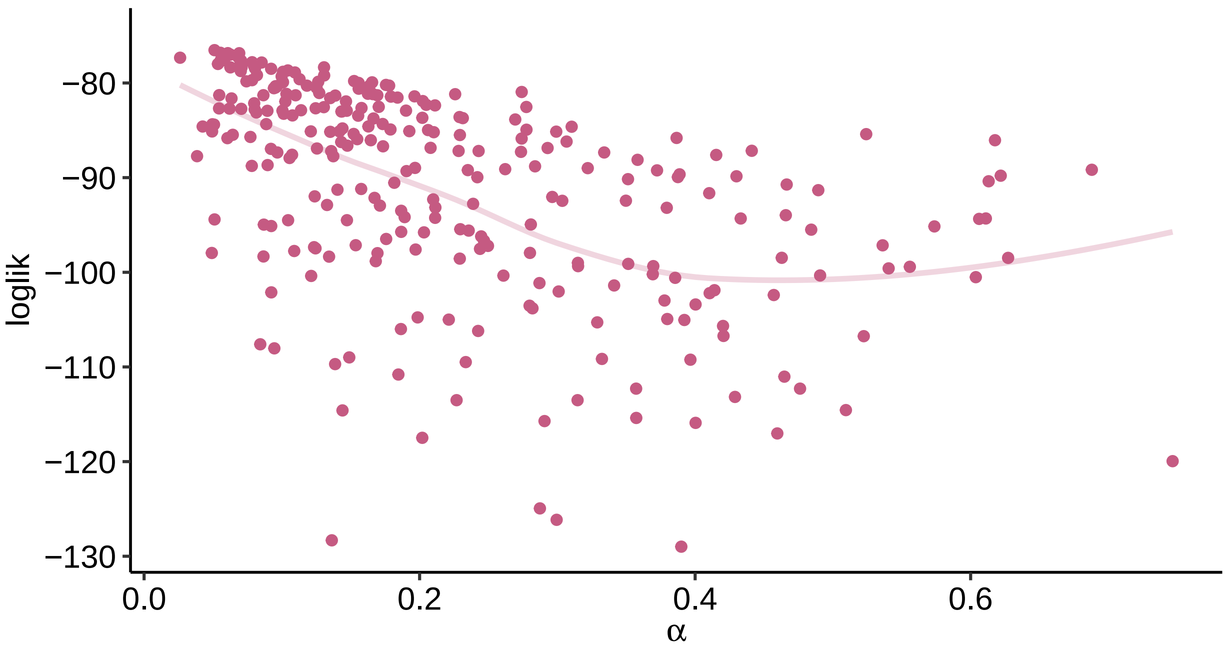

2.3.3.2 Confidence intervals

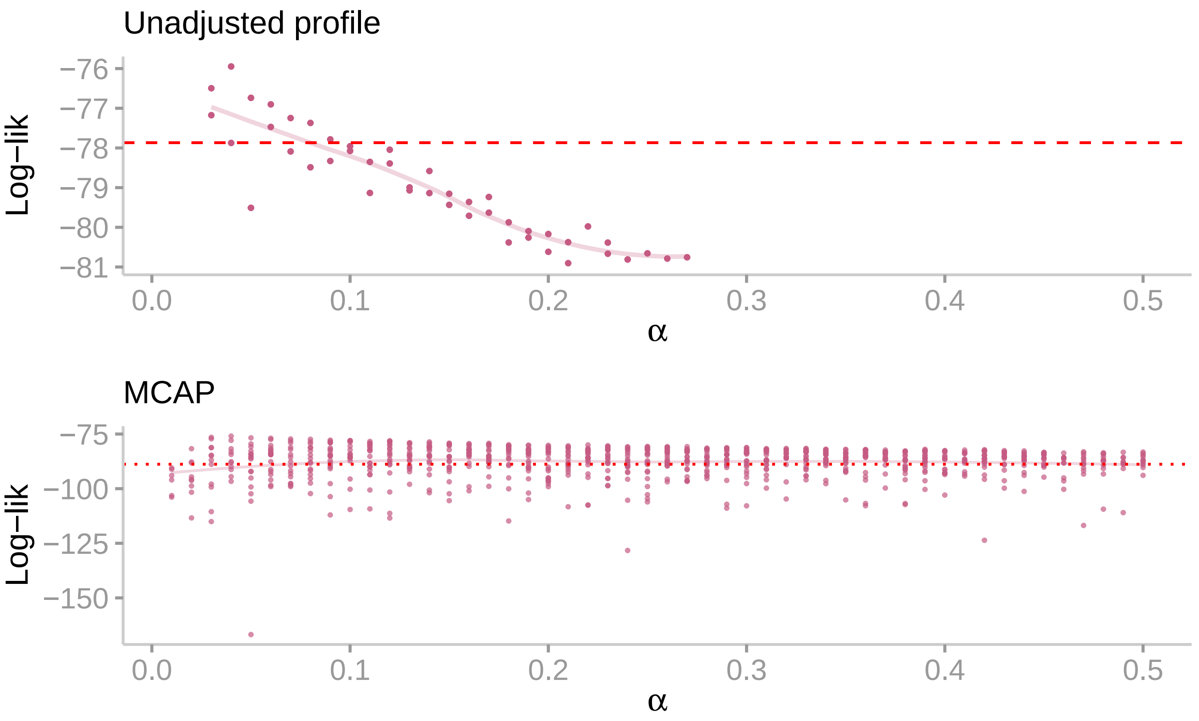

2.3.4 τ - Variance of the measured transmission rate

2.3.4.1 Initial likelihood surface

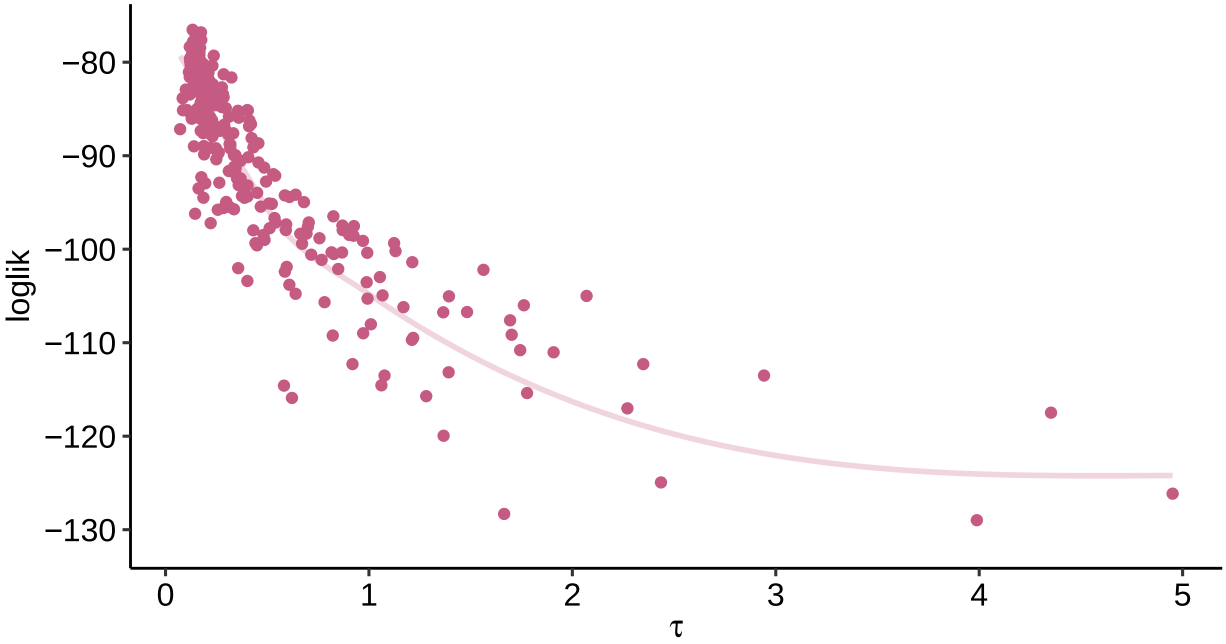

2.3.4.2 Confidence intervals

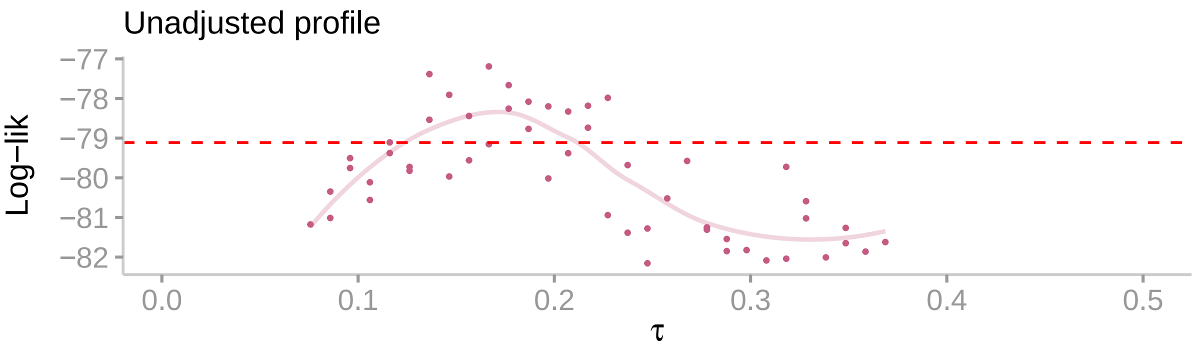

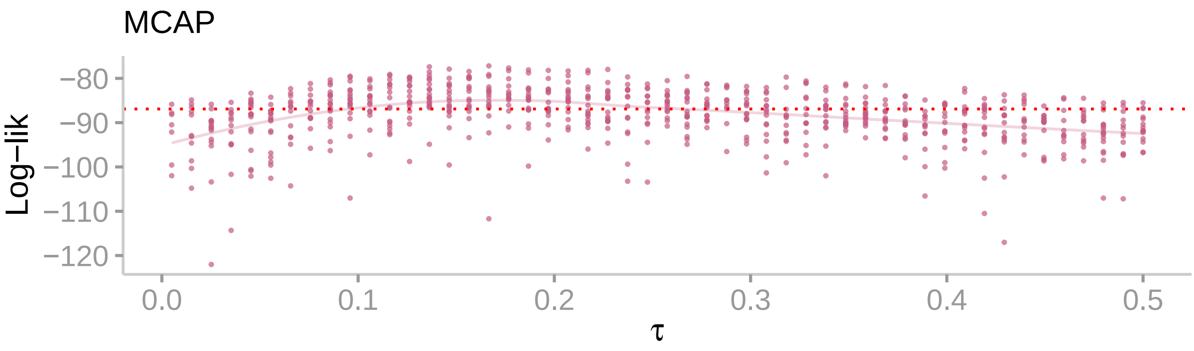

2.3.5 ν - Adjustment speed

2.3.5.1 Initial likelihood surface

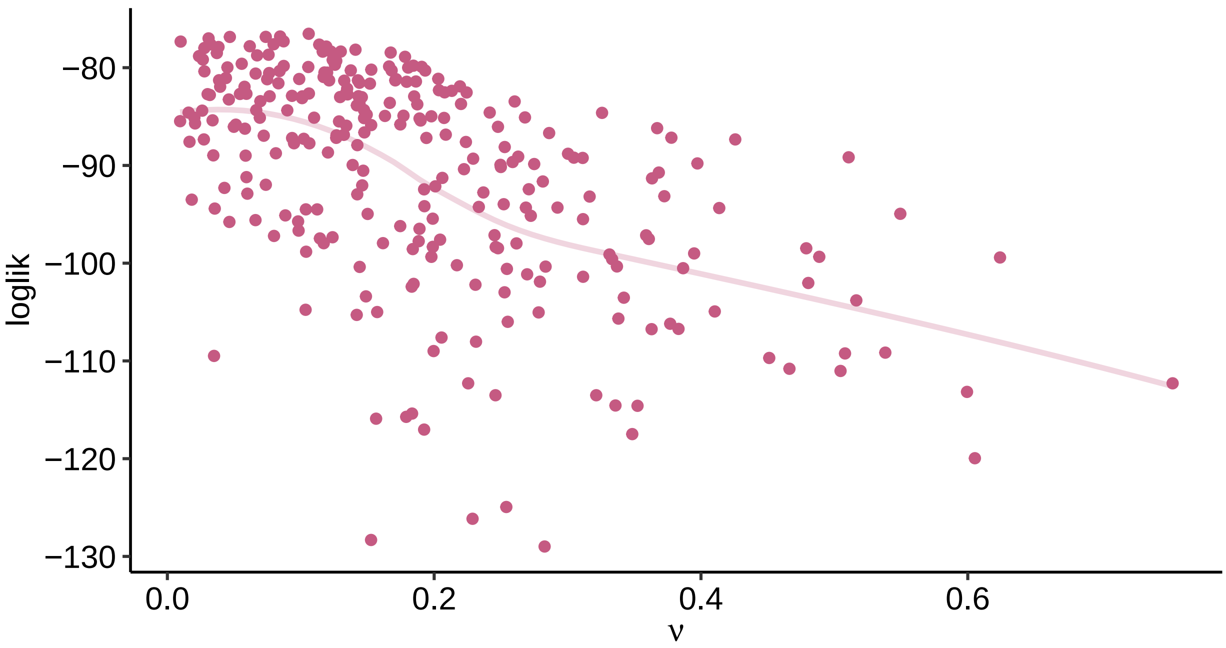

2.3.5.2 Confidence intervals

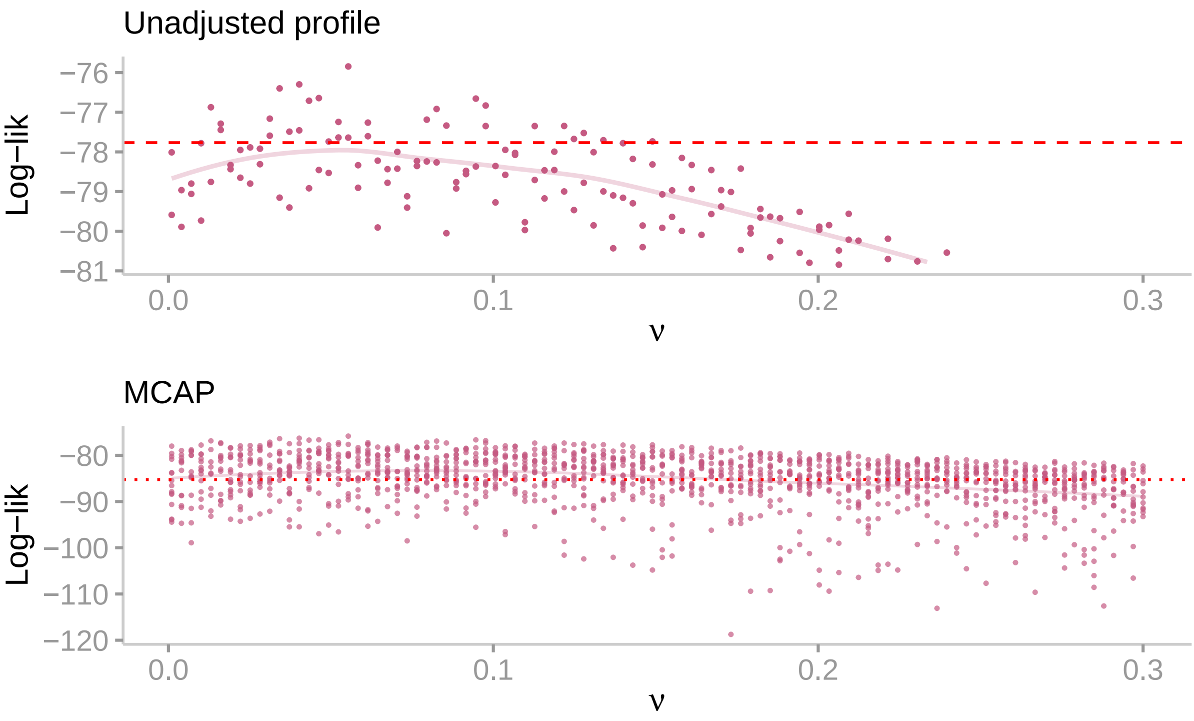

2.3.6 υ - Long-term goal

2.3.6.1 Initial likelihood surface

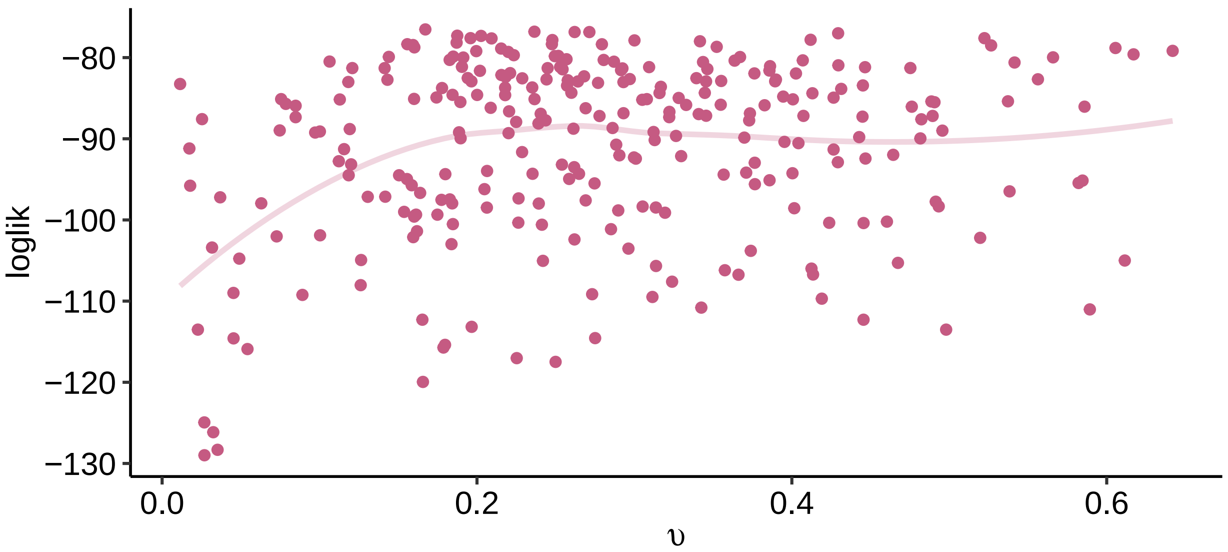

2.3.6.2 Confidence intervals

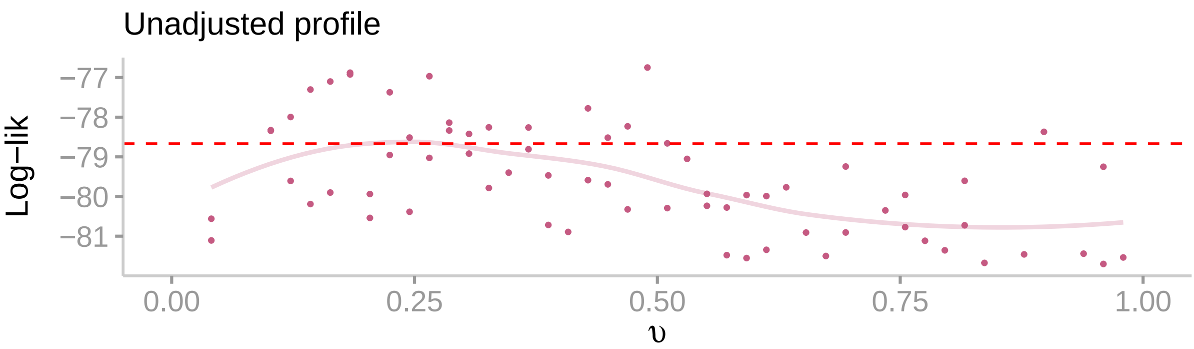

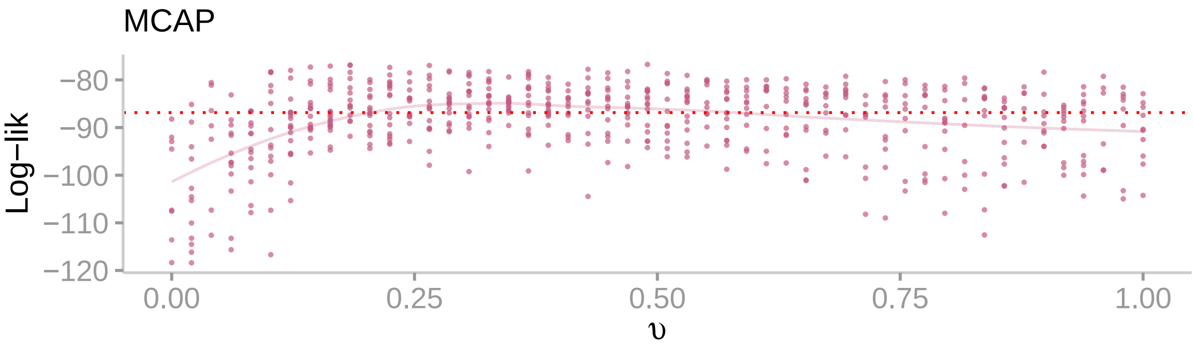

It took approximately 20 hours to compute the inference process (iterated filtering + particle filter) on DGP2's fixed parameters.

2.3.7 Estimates

In this section, we present a summary of the estimates obtained from the profile likelihood.

2.3.7.1 From the likelihood surface

We collate all the likelihood estimates from the previous steps into a single database. The resulting likelihood surfaces exhibit quadratic shapes (shown below). We, therefore, assume that these surfaces are approximations of the likelihood profiles. Following this assumption, we estimate each parameter's 95% confidence intervals.

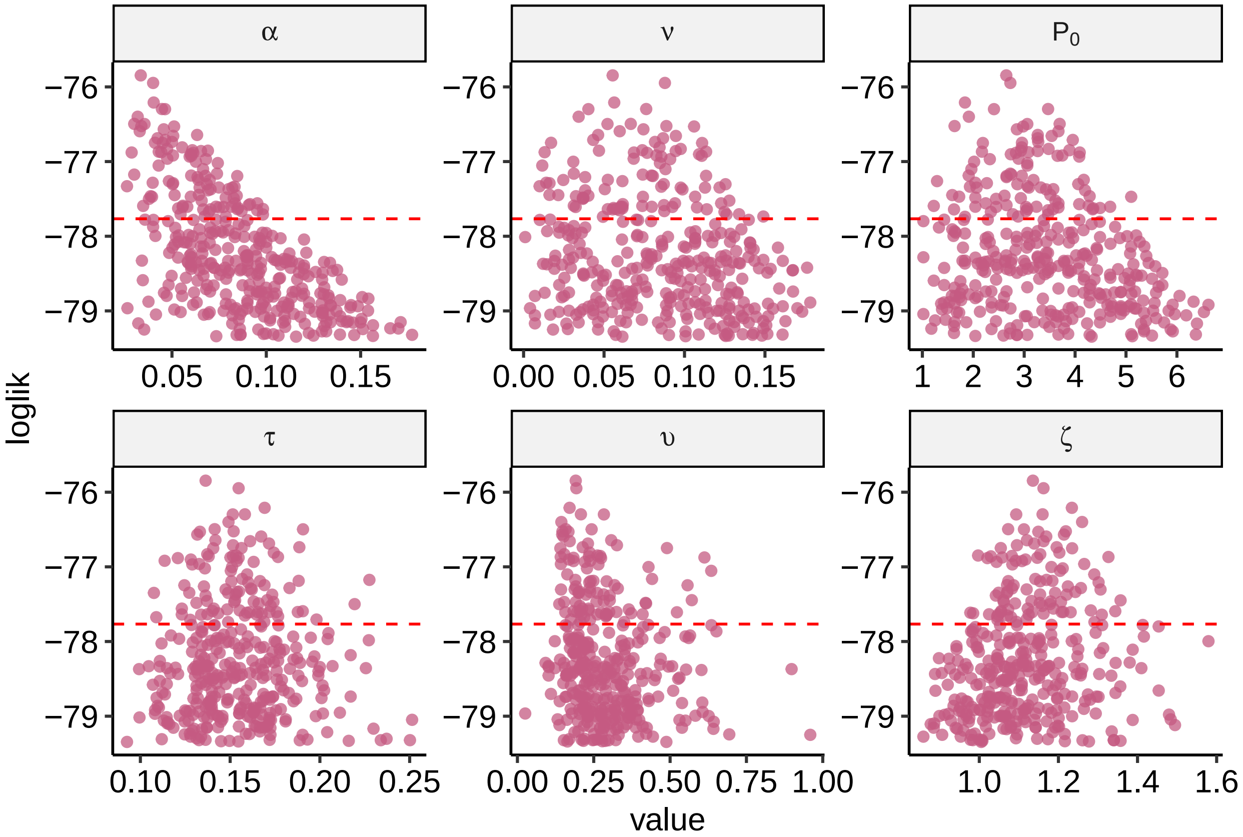

Parameter	MLE	Lower limit	Upper limit
α	0.033	0.026	0.098
ν	0.055	0.010	0.149
$\Re_0$	5.555	4.781	6.639
τ	0.136	0.108	0.228
υ	0.191	0.137	0.635
ζ	1.136	0.977	1.357
P_0	2.649	1.224	5.102

2.3.7.2 From likelihood profiles

Parameter	MLE	Lower limit	Upper limit
α	0.040	0.030	0.090
ν	0.055	0.013	0.149
$\Re_0$	6.040	4.393	6.789
τ	0.167	0.116	0.227
υ	0.490	0.102	0.898
ζ	1.235	0.898	1.388
P_0	1.837	1.020	5.102

2.3.7.3 From MCAP

Parameter	MLE	Lower limit	Upper limit
α	0.149	0.069	0.486
ν	0.080	0.001	0.172
$\Re_0$	4.907	3.798	7.507
τ	0.170	0.096	0.265
υ	0.337	0.205	0.580
ζ	1.003	0.776	1.535
P_0	3.028	1.136	7.294

2.3.7.4 Comparison

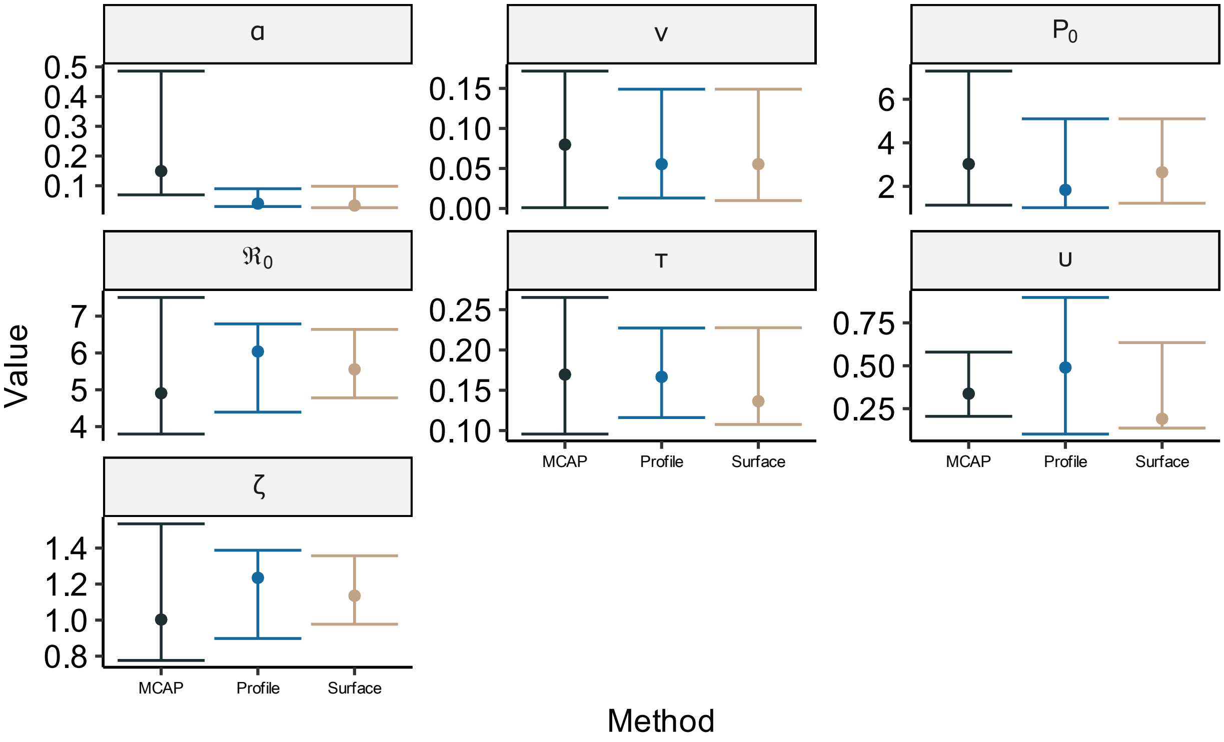

3 Prediction

The reader should recall that we obtain predictions for the latent states from the filtering distribution, which is intractable. To circumvent this difficulty, we use samples to approximate it. We briefly describe such a process.

3.1 Sampling space

First, we define a hypercube near the MLE (neighbourhood).

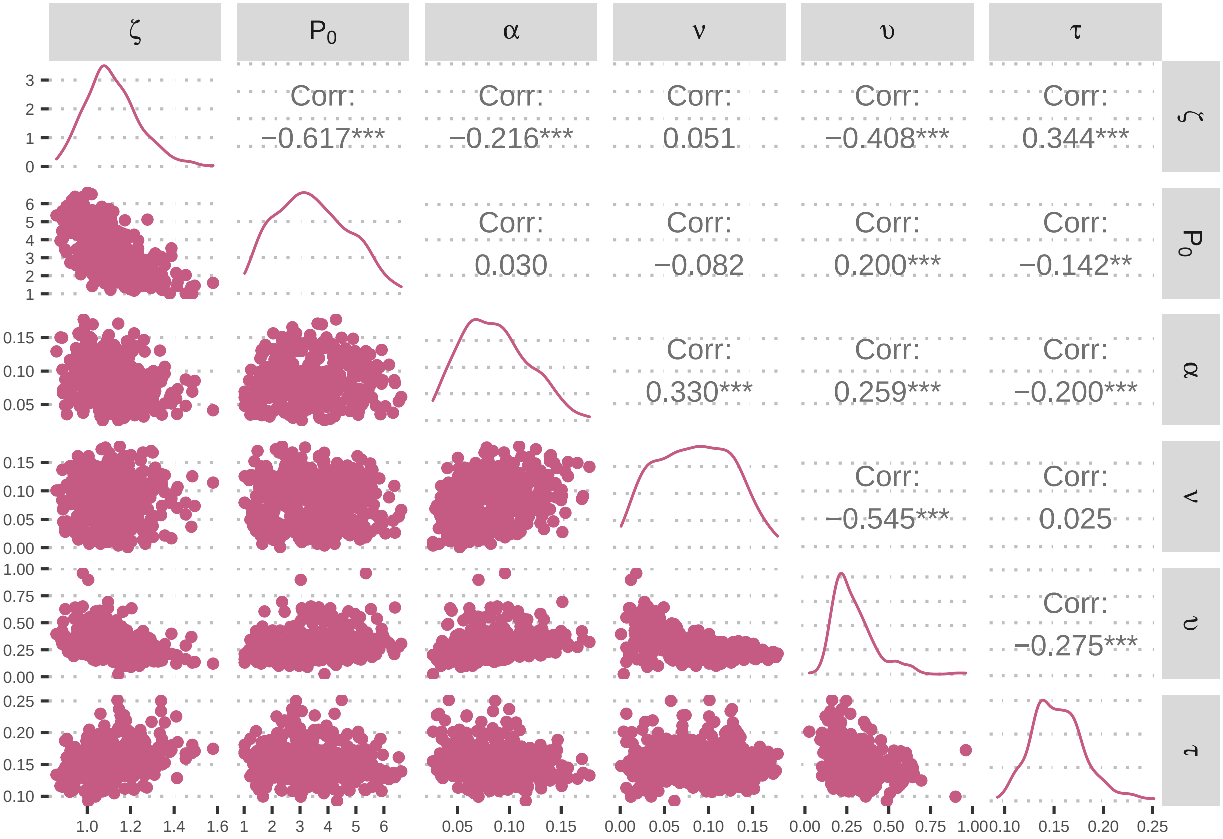

3.2 Draws

We then model the MLE's neighbourhood as a copula, from which sets of samples are drawn and whose marginal probability distributions are uniform but correlated at higher dimensions. We follow this procedure (instead of a hypercube) to address the complexity in this parameter space, where slight deviations from the regions of high likelihood yield unreasonably low values.

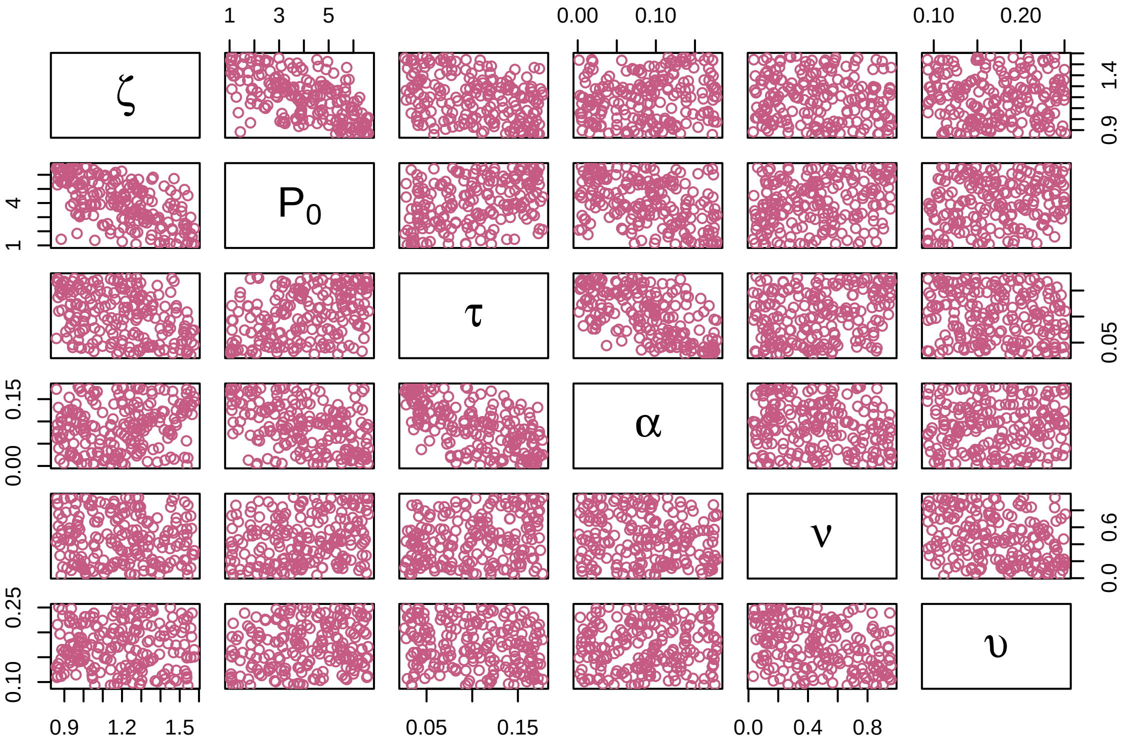

3.3 Cutoff

Using the sets of draws from the previous section (200), we run the particle filter and estimate their likelihood. Even though the copula prevents, to some extent, the exploration of undesired regions of the parameter space that may bias the results, some runs yield abnormal likelihood values. The violin plot below shows the likelihood distribution at different cut-off log-lik values. We notice that, in all plots, the likelihood concentrates on a common region, but several outliers skew the distributions.

In this graph, we explore the distribution of the log likelihood values for each starting coordinate at different cut-off points.

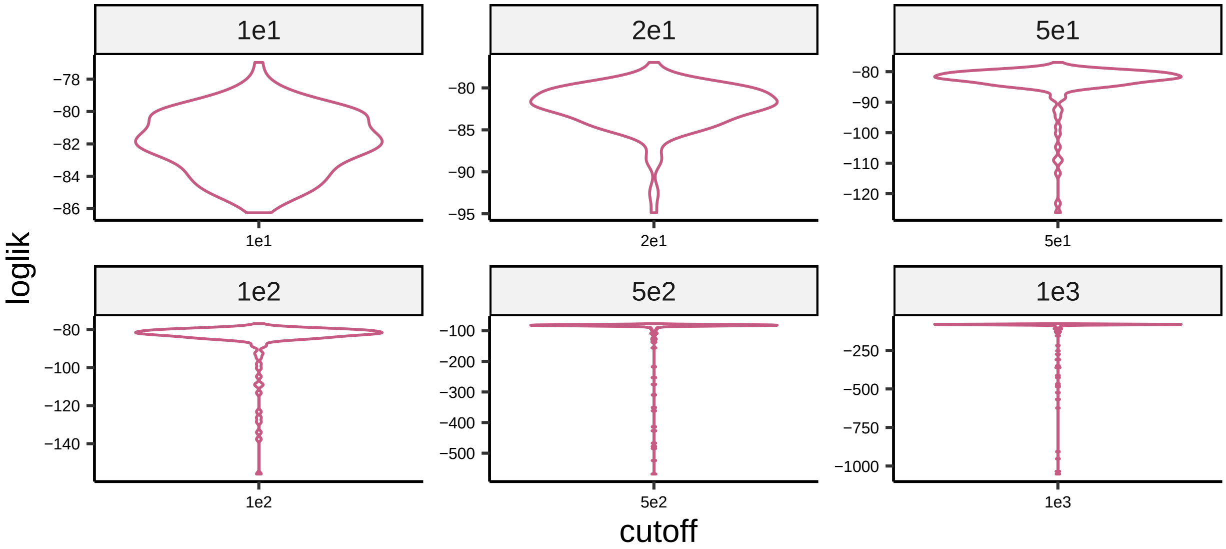

This outcome suggests that using all the samples may bias the estimates towards low probability regions, or even worse, produce computation overflows. Thus, we opt for a cut-off of 20 log-likelihood units (or 485×10^6 likelihood units) given that it contains 67 % of all the starting points and appears to include only a few outliers. It can be seen that these *outliers* are concentrated at the edges of the likelihood surface (high values of υ and ν). These results highlight the complex surface created by this particular high-dimensional DGP. The figure below compares included (light colour) and excluded (dark colour) parameter values.

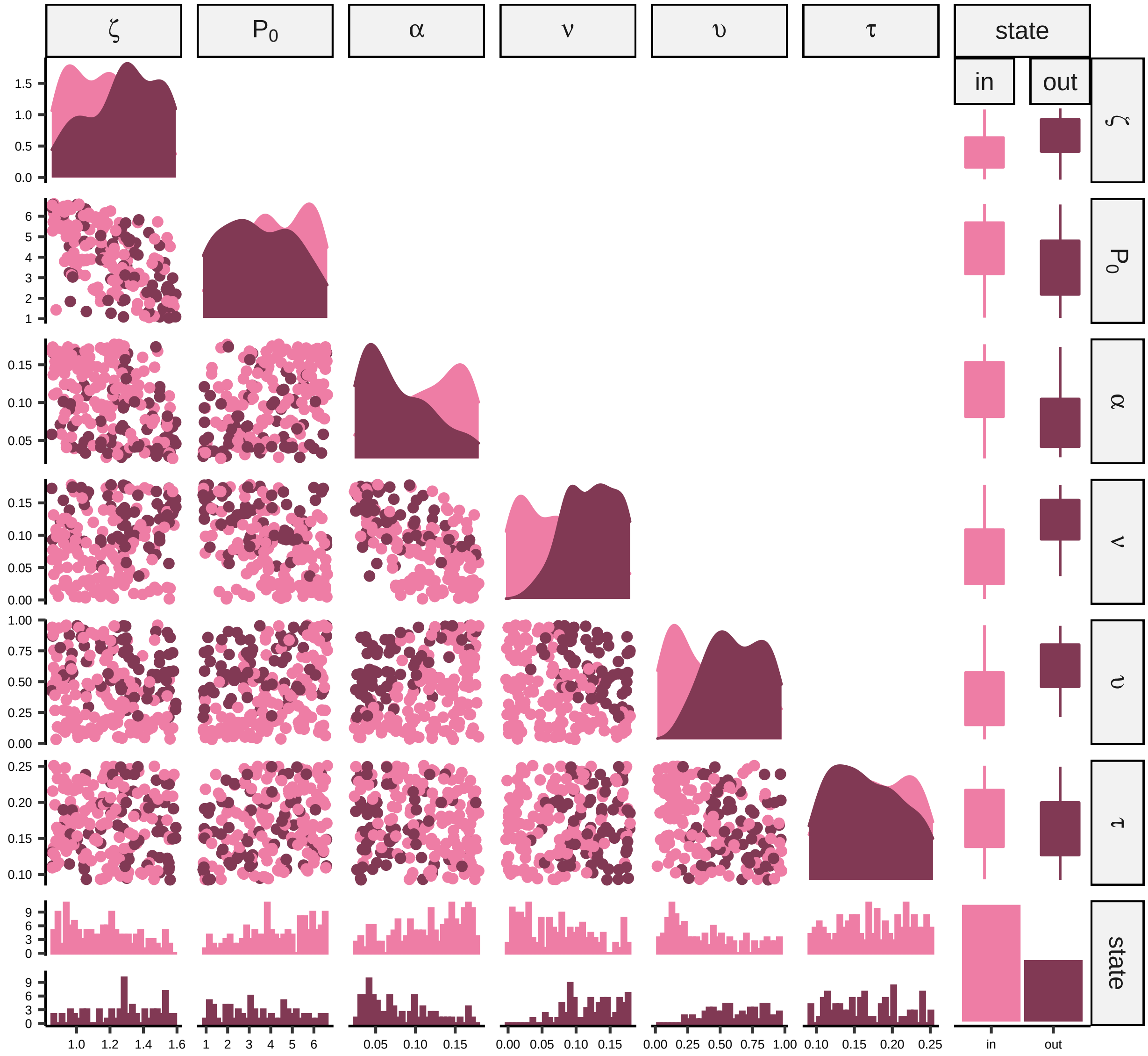

ζ
P_0
α
ν
υ
τ
state
in
out

We further compared the excluded sets against the likelihood surface (grey). Essentially, we notice that values near the upper limit of υ, ν, and to a lesser extent, ζ lead to numerical instability.

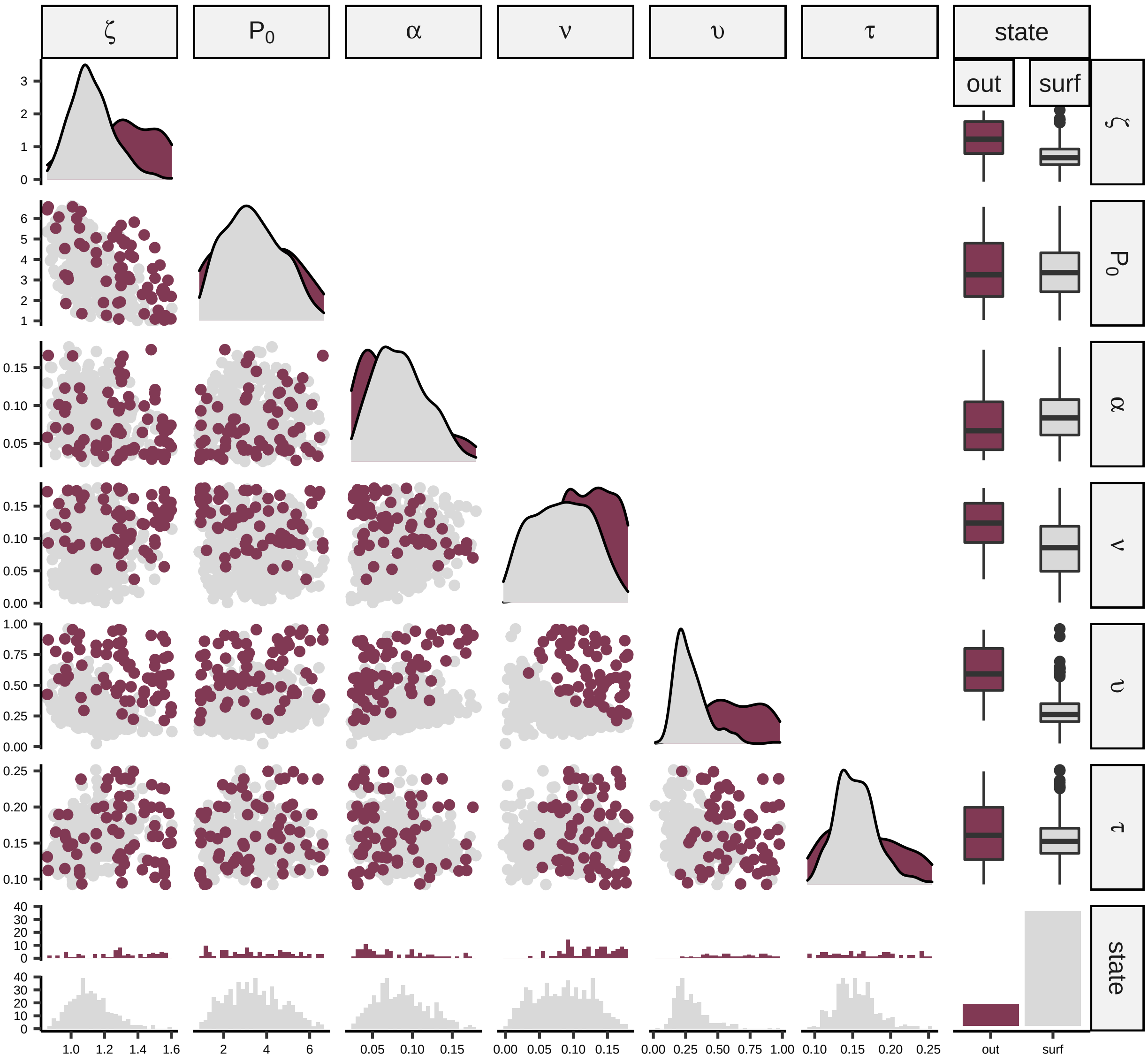

3.4 Hidden states

Finally, from the selected samples, we approximate the filtering distribution at each time step.

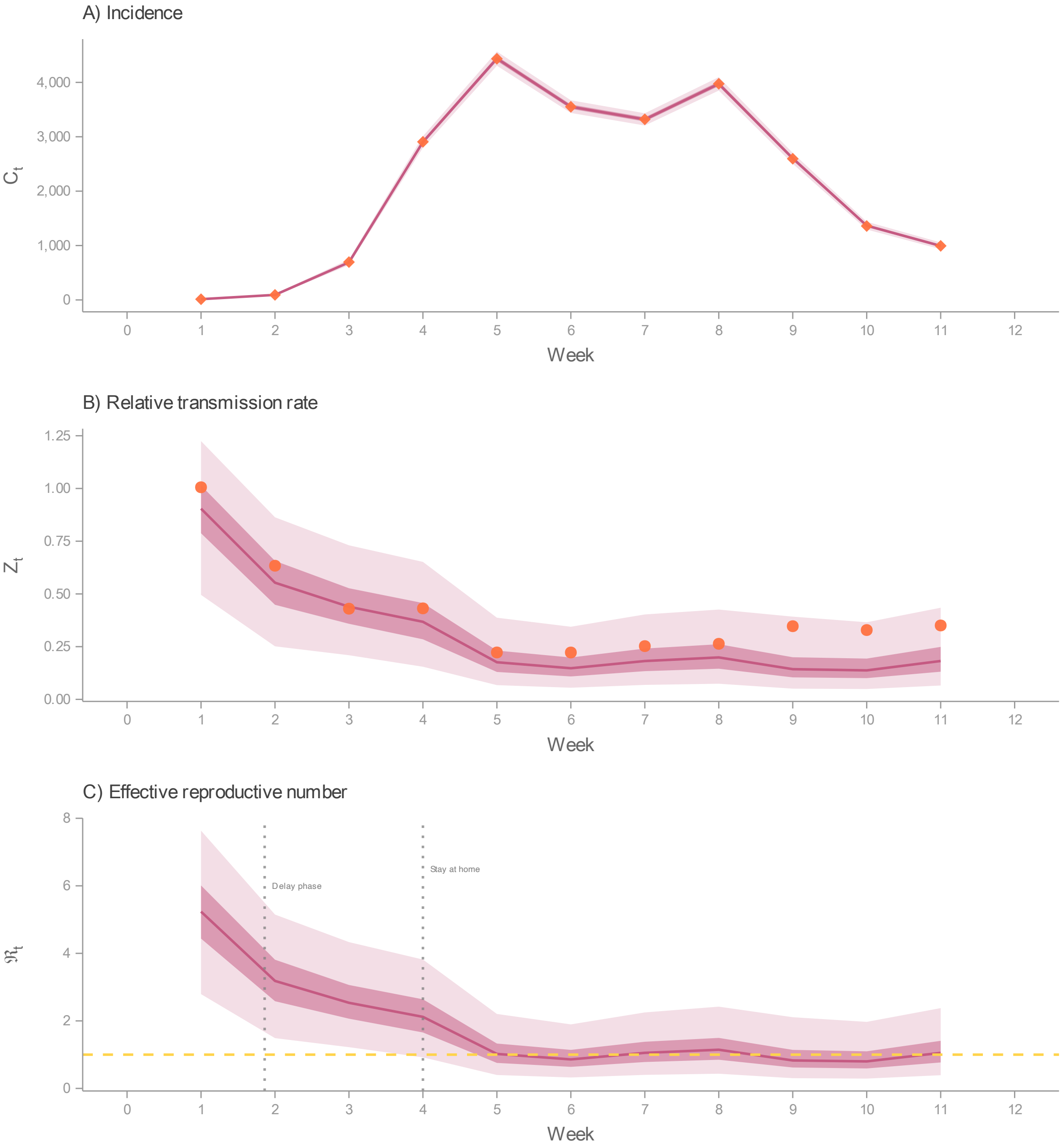

4 Original Computing Environment

```
## R version 4.1.2 (2021-11-01)
## Platform: aarch64-apple-darwin20 (64-bit)
## Running under: macOS Monterey 12.3.1
##
## Matrix products: default
## BLAS:   /Library/Frameworks/R.framework/Versions/4.1-arm64/Resources/lib/libRblas.0.dylib
## LAPACK: /Library/Frameworks/R.framework/Versions/4.1-arm64/Resources/lib/libRlapack.dylib
##
## locale:
## [1] en_US.UTF-8/en_US.UTF-8/en_US.UTF-8/C/en_US.UTF-8/en_US.UTF-8
##
## attached base packages:
## [1] parallel  stats     graphics  grDevices utils     datasets  methods
## [8] base
##
## other attached packages:
##  [1] scales_1.1.1      patchwork_1.1.1   ggrepel_0.9.1     ggpubr_0.4.0
##  [5] ggalt_0.4.0       GGally_2.1.2      ggplot2_3.3.5     extraDistr_1.9.1
##  [9] tidyr_1.1.4       tictoc_1.0.1      tibble_3.1.6      stringr_1.4.0
## [13] readxl_1.3.1      readr_2.1.1       purrr_0.3.4       pomp_3.6
## [17] lubridate_1.8.0   imputeTS_3.2      dplyr_1.0.8       doRNG_1.8.2
## [21] rngtools_1.5.2    doParallel_1.0.16 iterators_1.0.13  foreach_1.5.1
## [25] copula_1.0-1
##
## loaded via a namespace (and not attached):
##  [1] colorspace_2.0-2    ggsignif_0.6.3      ellipsis_0.3.2
##  [4] gridtext_0.1.4      ggtext_0.1.1        rstudioapi_0.13
##  [7] farver_2.1.0        gsl_2.1-7.1         bit64_4.0.5
## [10] fansi_0.5.0         mvtnorm_1.1-3       xml2_1.3.3
## [13] splines_4.1.2       codetools_0.2-18    extrafont_0.17
## [16] knitr_1.37          broom_0.7.10        Rttf2pt1_1.3.9
## [19] stabledist_0.7-1    compiler_4.1.2      backports_1.4.1
## [22] Matrix_1.3-4        fastmap_1.1.0       cli_3.3.0
## [25] htmltools_0.5.2     tools_4.1.2         coda_0.19-4
## [28] gtable_0.3.0        glue_1.6.2          reshape2_1.4.4
## [31] maps_3.4.0          Rcpp_1.0.7          carData_3.0-4
## [34] cellranger_1.1.0    fracdiff_1.5-1      vctrs_0.4.1
## [37] urca_1.3-0          nlme_3.1-153        extrafontdb_1.0
## [40] lmtest_0.9-39       timeDate_3043.102   xfun_0.29
## [43] lifecycle_1.0.1     rstatix_0.7.0       MASS_7.3-54
## [46] zoo_1.8-9           vroom_1.5.7         hms_1.1.1
## [49] proj4_1.0-10.1      RColorBrewer_1.1-2  yaml_2.2.1
## [52] quantmod_0.4.18     curl_4.3.2          reshape_0.8.8
## [55] stringi_1.7.6       highr_0.9           tseries_0.10-49
## [58] pcaPP_1.9-74        TTR_0.24.3          rlang_1.0.2
## [61] pkgconfig_2.0.3     evaluate_0.14       lattice_0.20-45
## [64] labeling_0.4.2      stinepack_1.4       bit_4.0.4
## [67] tidyselect_1.1.1    deSolve_1.30        plyr_1.8.6
## [70] magrittr_2.0.3      R6_2.5.1            generics_0.1.1
## [73] ADGofTest_0.3       mgcv_1.8-38         pillar_1.6.4
## [76] withr_2.4.3         xts_0.12.1          abind_1.4-5
## [79] nnet_7.3-16         ash_1.0-15          pspline_1.0-18
```

```
## [82] crayon_1.4.2        car_3.0-12          KernSmooth_2.23-20
## [85] utf8_1.2.2          tzdb_0.2.0          rmarkdown_2.11
## [88] grid_4.1.2          forecast_8.15       digest_0.6.29
## [91] numDeriv_2016.8-1.1 stats4_4.1.2        munsell_0.5.0
## [94] quadprog_1.5-8
```
