# Supplementary material for: Inferring the effective reproductive number from deterministic and semi-deterministic compartmental models using incidence and mobility data
Source: PLoS Comput Biol. 2022 Jun 27;18(6):e1010206. doi: 10.1371/journal.pcbi.1010206 (PMC9269962; doi:10.1371/journal.pcbi.1010206)
Supplement: S7 Text — (PDF) [file pcbi.1010206.s007.pdf]

S7 Appendix

This appendix aims to illustrate the inference process applied to DGP3. This structure consists of nine candidate deterministic process models (PM3) and an observational or measurement model (OM2) that accounts for the daily COVID-19 cases detected in Ireland's first wave. We envision this inference process in a Bayesian context, where the predicted values stem from DGP3's expected value, which is approximated using Hamiltonian Monte Carlo (HMC).

Contents

1 DGP3 - Adaptive expectations **3**
1.1 Process model (PM3) . . . 3
1.2 Measurement model candidates (OM2) . . . 3
1.2.1 Poisson . . . 3
1.2.2 Negative binomial . . . 3

2 Inference (Poisson) **4**
2.1 Priors . . . 4
2.2 Sampling . . . 4
2.2.1 1st order delay . . . 5
2.2.2 2nd order delay . . . 5
2.2.3 3rd order delay . . . 6
2.2.4 4th order delay . . . 6
2.2.5 5th order delay . . . 7
2.2.6 6th order delay . . . 7
2.2.7 7th order delay . . . 8
2.2.8 8th order delay . . . 8
2.2.9 9th order delay . . . 9
2.3 Expected values . . . 10
2.3.1 Predicted incidence compared to daily case counts . . . 10
2.3.2 Predicted relative contact rate compared to mobility indexes . . . 11
2.3.3 Likelihood by delay order . . . 12
2.3.4 Accuracy . . . 13
2.4 Posterior distribution . . . 15
2.5 Candidate selection . . . 16

3 Computational time **16**

4 Inference (Negative binomial) **17**
4.1 Five unknowns . . . 17
4.1.1 1st order delay . . . 17
4.1.2 All delay orders . . . 18
4.1.3 Exploratory estimates . . . 25
4.1.4 Exploratory predicted relative contact rate . . . 26
4.1.5 Exploratory predicted effective contact rate . . . 27
4.2 Only one unknown . . . 28
4.2.1 Trace plots . . . 28
4.3 Two specific unknowns . . . 29

4.3.1 Expected values . . . 29
4.3.2 Likelihood . . . 31
4.3.3 Accuracy . . . 32
4.3.4 Posterior distribution . . . 34

5 Prediction of hidden states **35**

6 Original Computing Environment **36**

References **37**

1 DGP3 - Adaptive expectations

1.1 Process model (PM3)

$$\frac{dS}{dt} = -S_t \lambda_t \tag{1}$$

$$\frac{dE}{dt} = S_t \lambda_t - \sigma E_t \tag{2}$$

$$\frac{dP}{dt} = \omega \sigma E_t - \eta P_t \tag{3}$$

$$\frac{dI}{dt} = \eta P_t - \gamma I_t \tag{4}$$

$$\frac{dA}{dt} = (1 - \omega)\sigma E_t - \kappa A_t \tag{5}$$

$$\frac{dR}{dt} = \kappa A_t + \gamma I_t \tag{6}$$

$$\lambda_t = \frac{\beta_t (I(_t + P_t + \mu A_t)}{N_t} \tag{7}$$

$$\beta_t = \zeta Z_t^1 \tag{8}$$

$$\frac{dZ^i}{dt} = \begin{cases} \frac{(\upsilon - Z_t^i)}{(\nu^{-1}/n)} & \text{for} \quad i = n \\ \\ \frac{(Z_t^{i+1} - Z_t^i)}{(\nu^{-1}/n)} & \text{for} \quad i < n \end{cases} \tag{9}$$

Where $i \in (\mathbb{Z}_+)^n$ denotes each of the stages in an n-order information delay structure.

1.2 Measurement model candidates (OM2)

$$\frac{dC}{dt} = \eta P_t - C_t \delta(t \, mod \, 1) \tag{10}$$

1.2.1 Poisson

$$y_d^1 \sim Pois(C_t) \tag{11}$$

1.2.2 Negative binomial

$$y_d^1 \sim NBin(C_t, \phi^{-1}) \tag{12}$$

2 Inference (Poisson)

2.1 Priors

For all of the nine candidate models, we adopt the following priors:

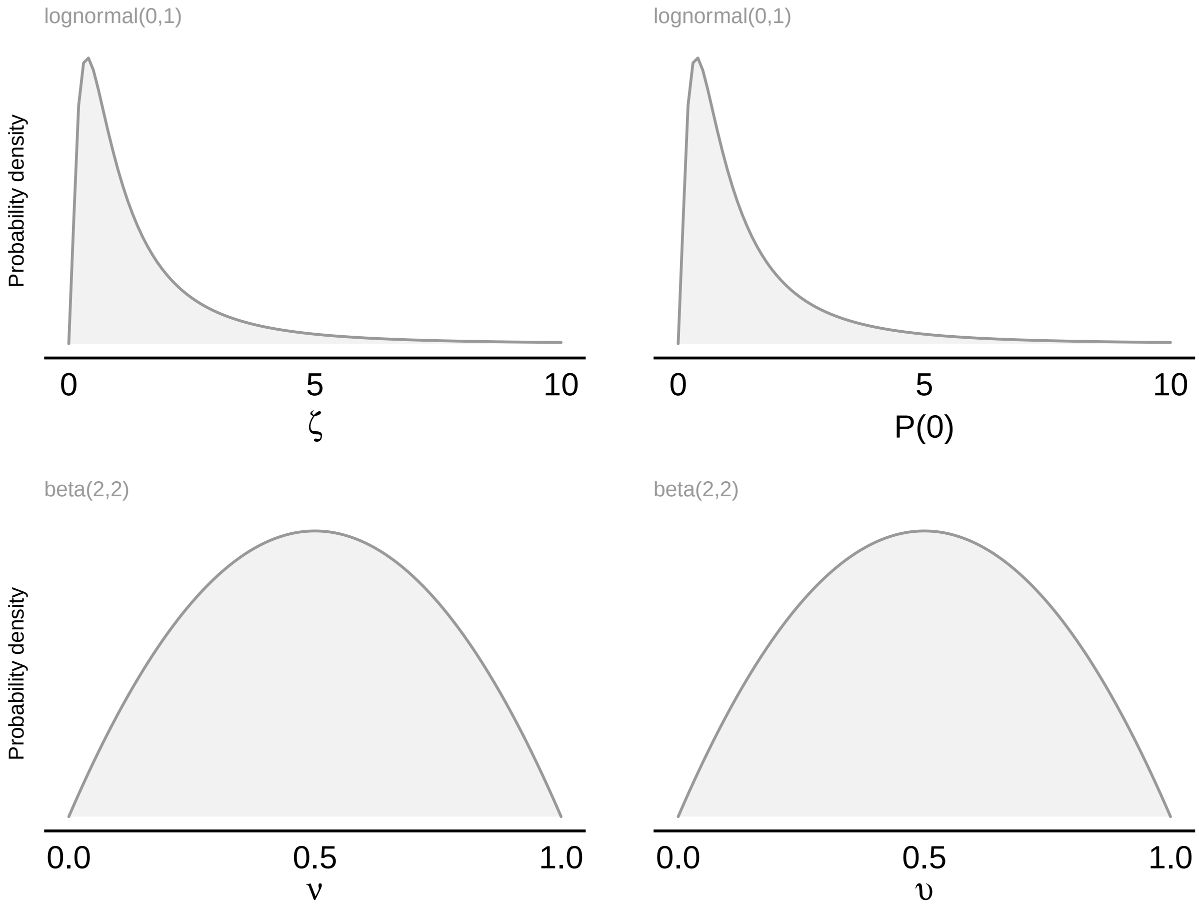

2.2 Sampling

For validation purposes, we show the results from the sampling algorithm (HMC) by means of trace plots. These visualisation tools are time-series of the draws for a particular parameter. Here, *time* refers to the order in which the draws were sampled. These plots suggest that there are no issues in the sampling procedure. Further diagnostics (see the Github repository) indicate that no *pathological behaviour* was observed during the process, and adequate Effective Sample Sizes and potential scale reduction factors ($\widehat{R} < 1.01$) were obtained. These outcomes suggest that the Markov chains converged to the posterior distribution.

2.2.1 1st order delay

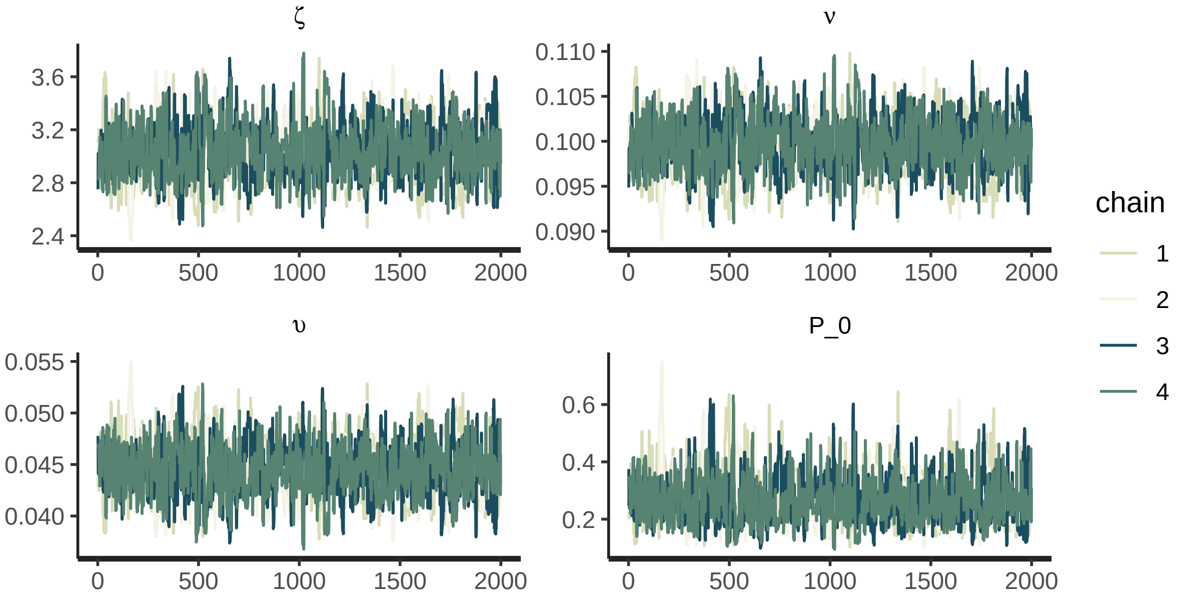

2.2.2 2nd order delay

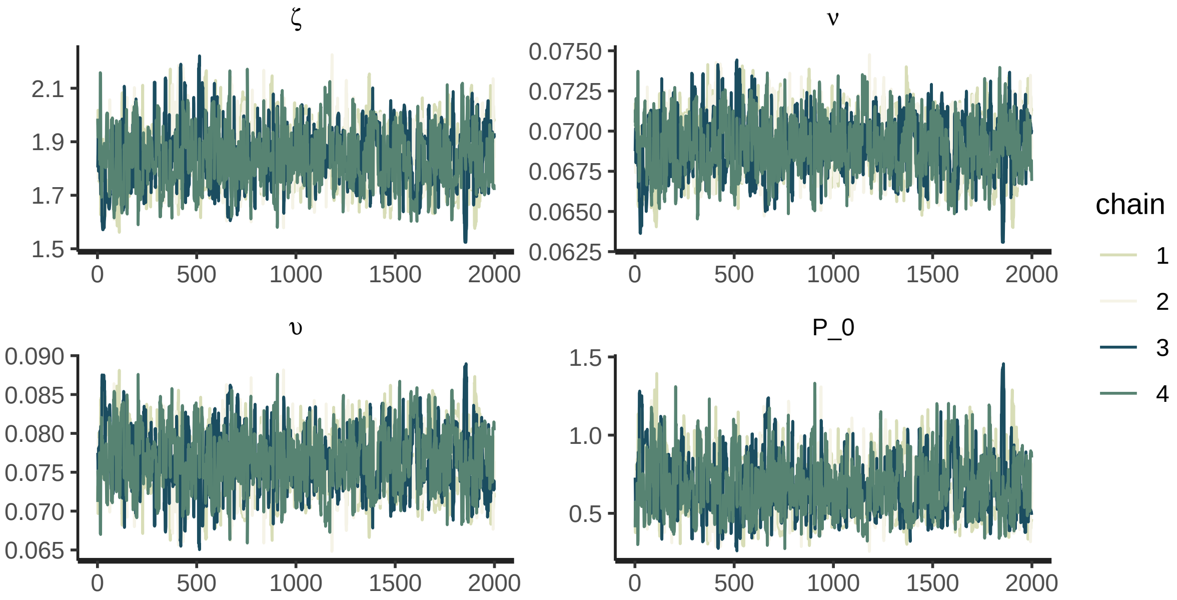

2.2.3 3rd order delay

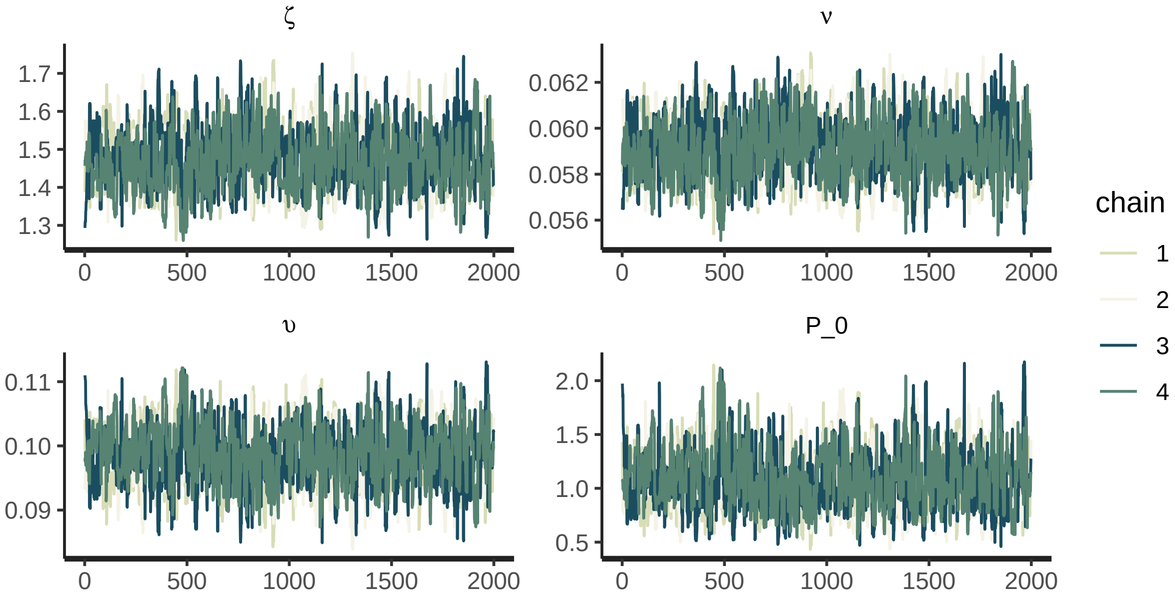

2.2.4 4th order delay

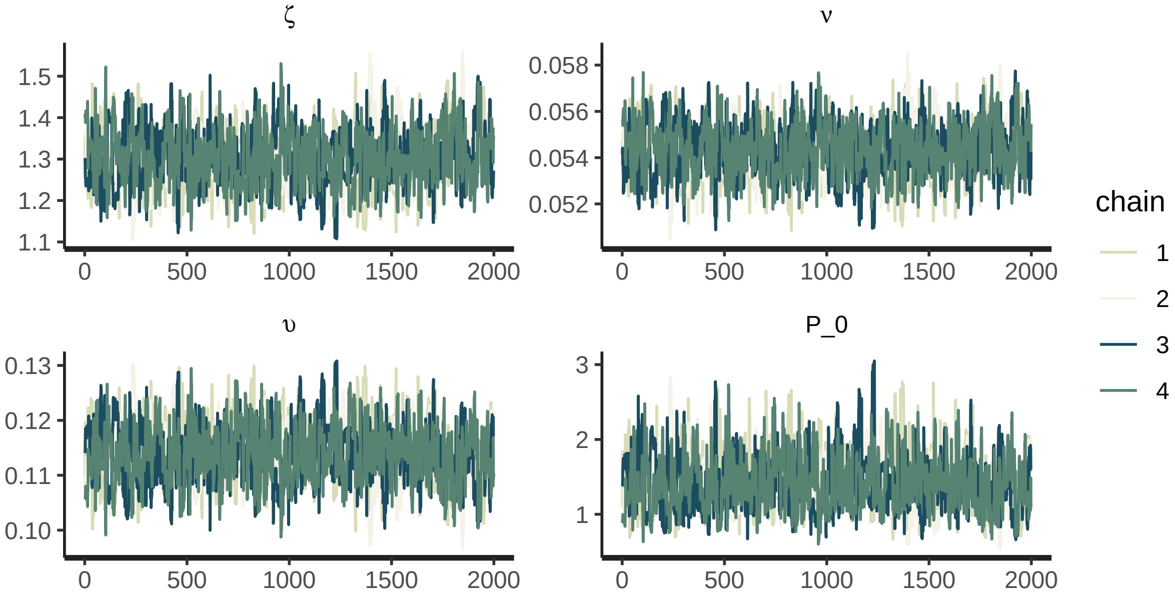

2.2.5 5th order delay

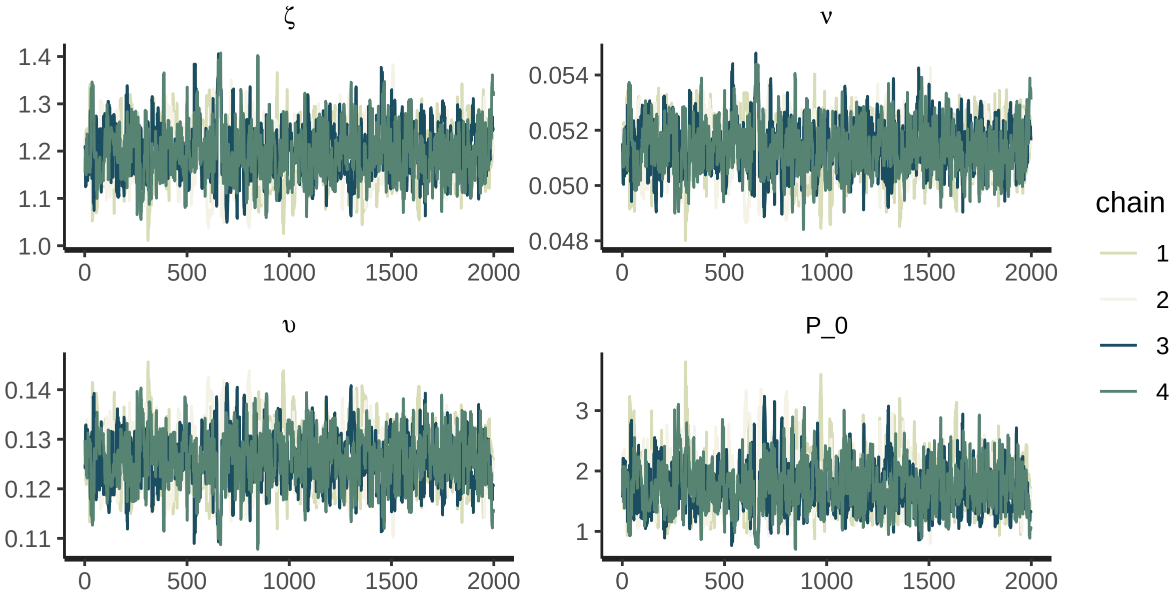

2.2.6 6th order delay

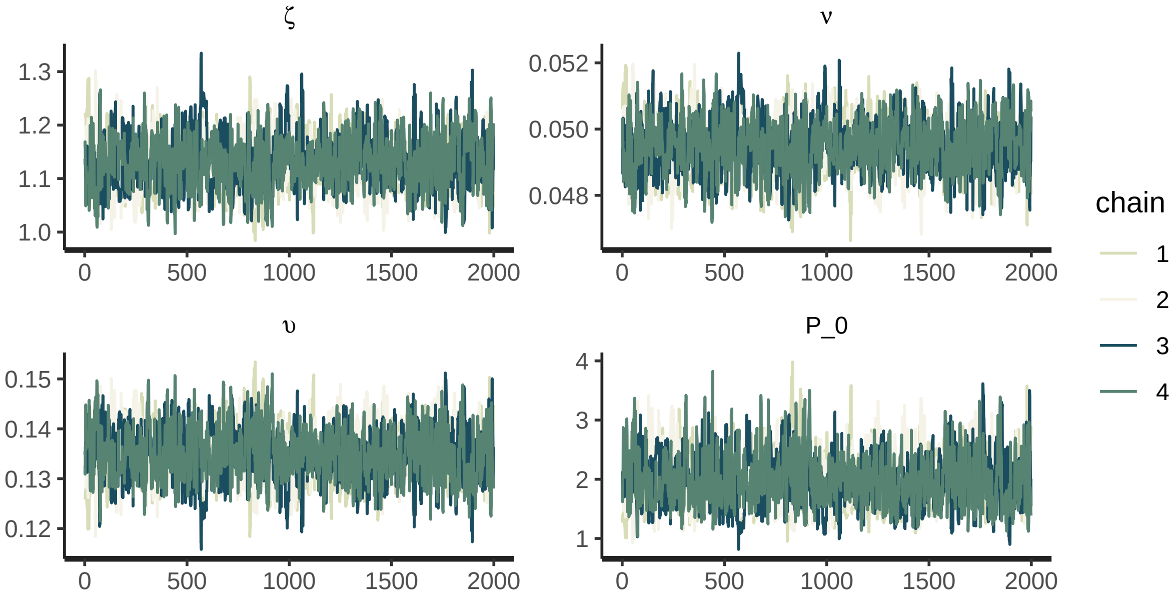

2.2.7 7th order delay

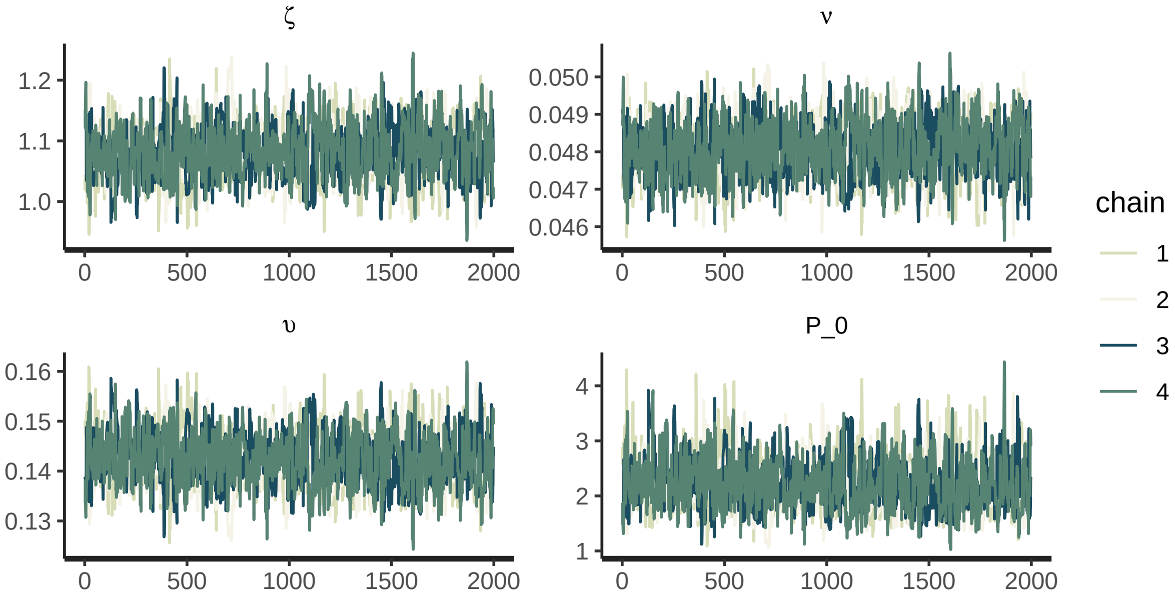

2.2.8 8th order delay

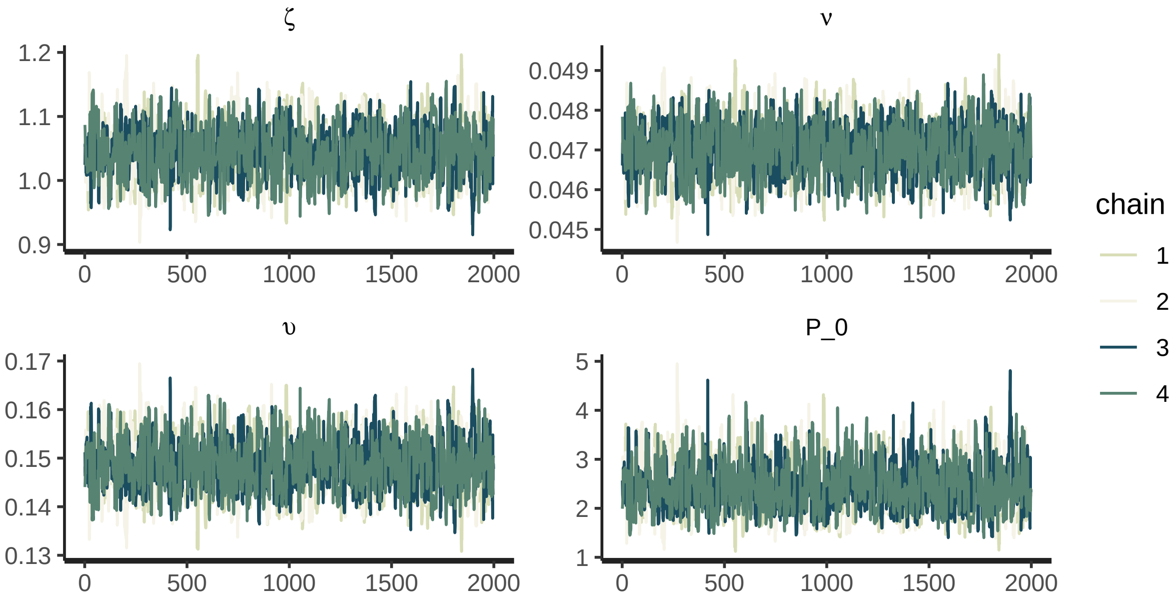

2.2.9 9th order delay

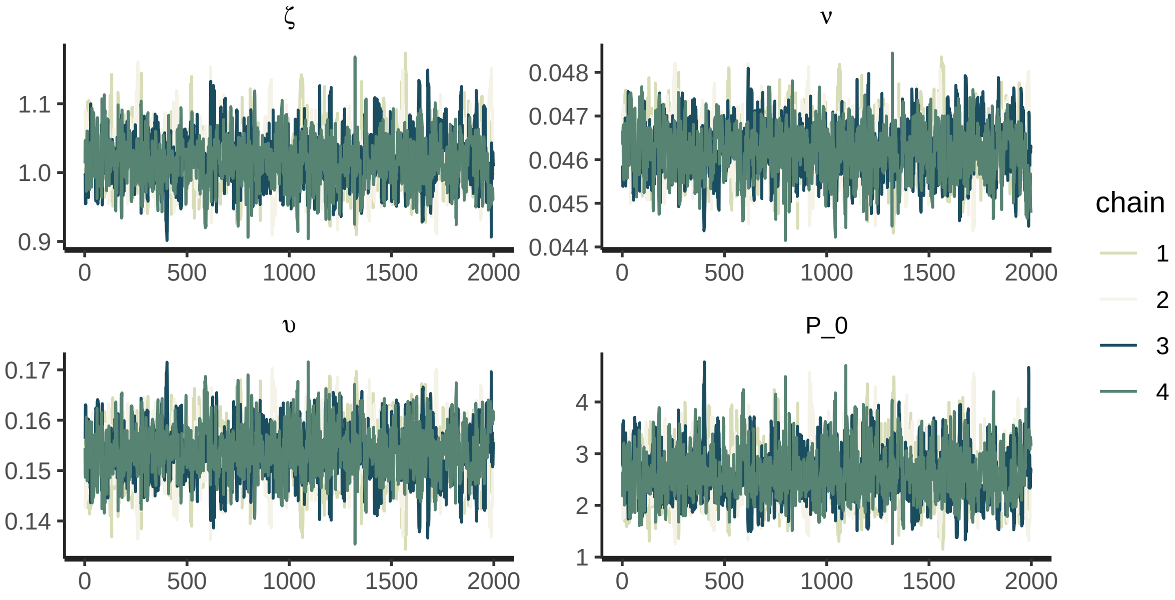

2.3 Expected values

2.3.1 Predicted incidence compared to daily case counts

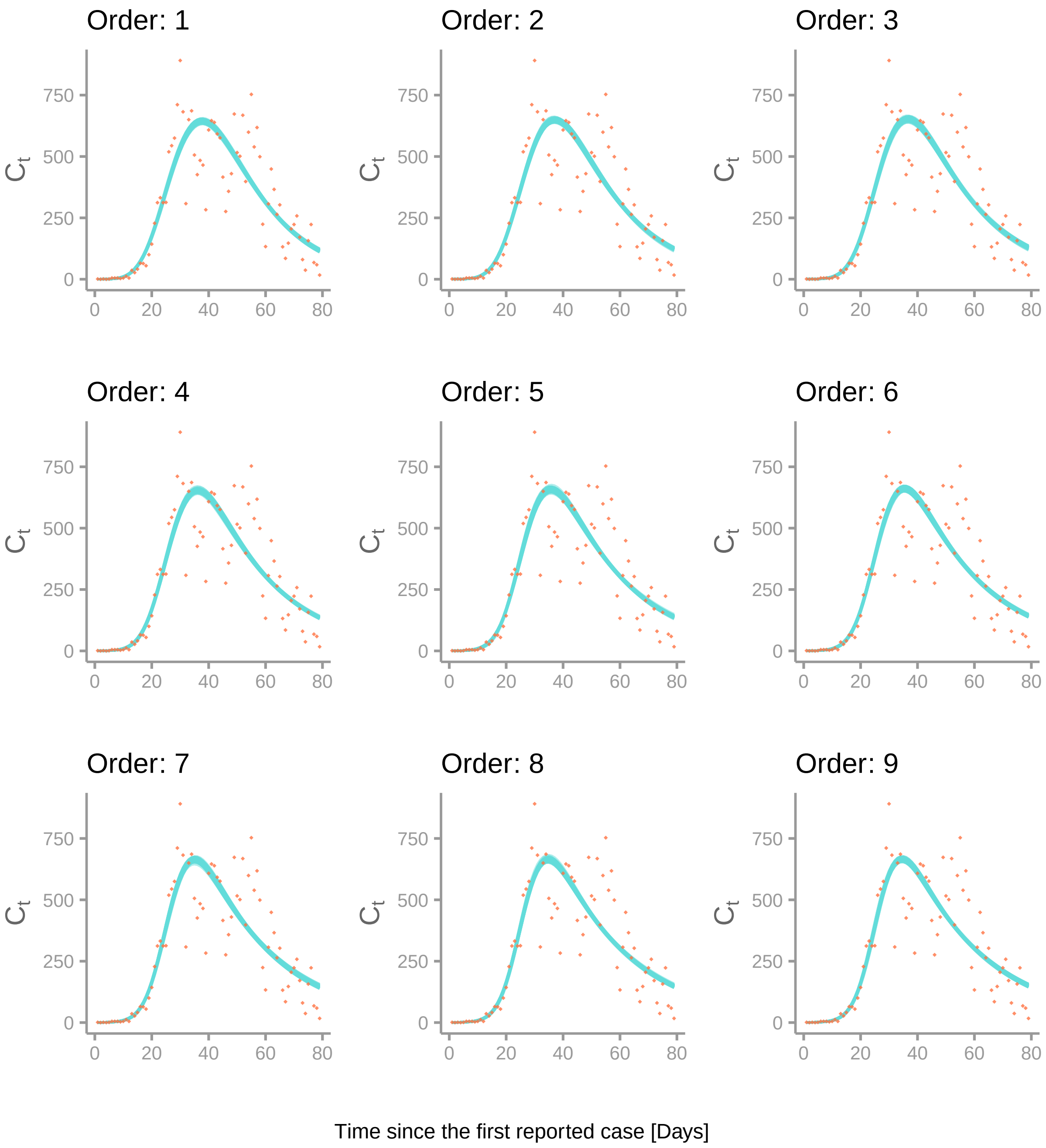

2.3.2 Predicted relative contact rate compared to mobility indexes

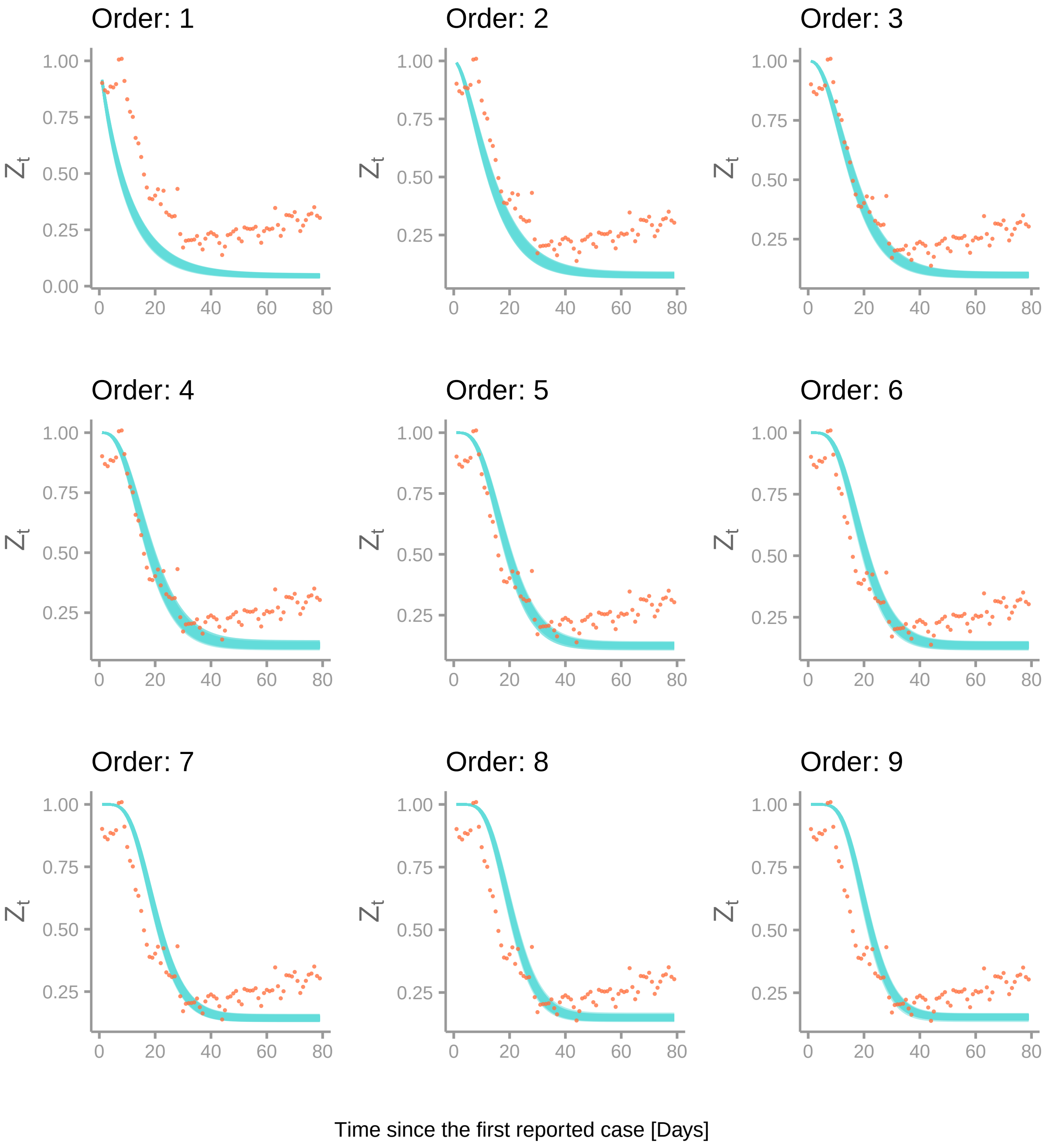

2.3.3 Likelihood by delay order

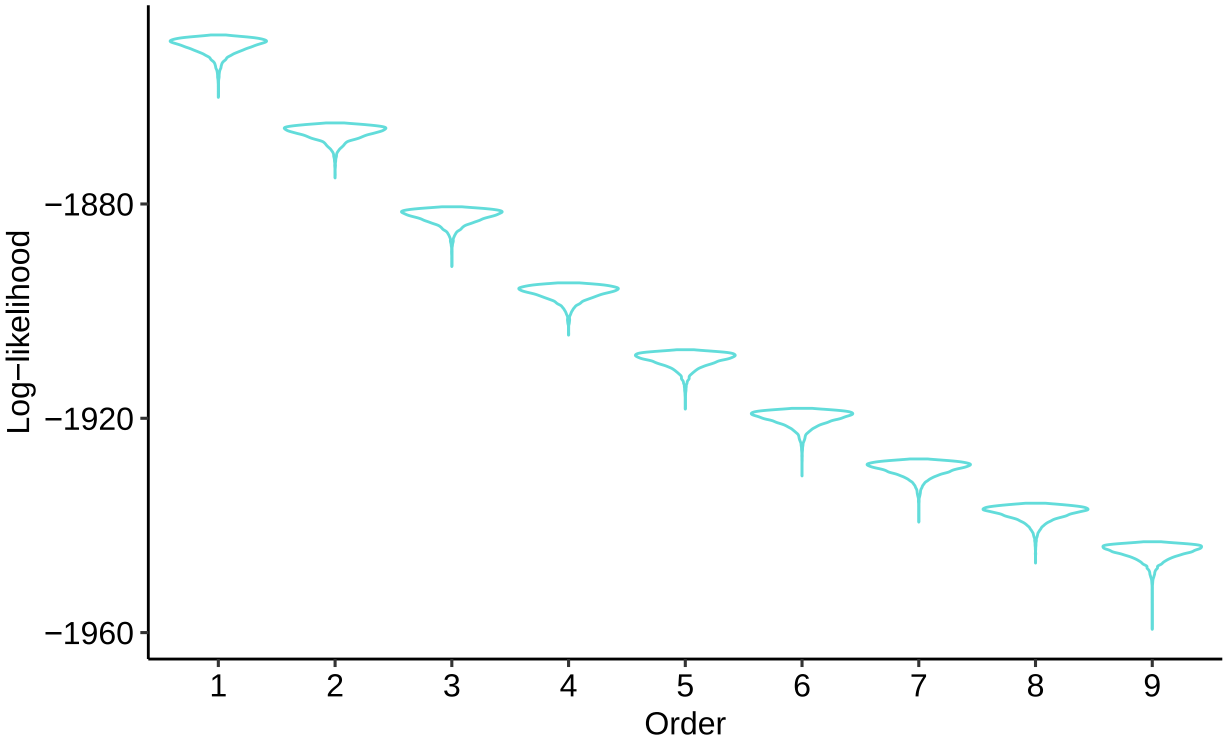

order	mean	q2.5	q25	q50	q75	q97.5
1	-1850.5	-1854.3	-1851.3	-1850.2	-1849.5	-1848.7
2	-1866.8	-1870.0	-1867.5	-1866.5	-1865.8	-1865.1
3	-1882.4	-1885.9	-1883.1	-1882.1	-1881.4	-1880.7
4	-1896.7	-1900.1	-1897.3	-1896.3	-1895.7	-1894.9
5	-1909.2	-1912.8	-1909.8	-1908.8	-1908.1	-1907.4
6	-1920.1	-1923.7	-1920.7	-1919.7	-1919.0	-1918.3
7	-1929.5	-1933.0	-1930.2	-1929.2	-1928.5	-1927.8
8	-1937.8	-1941.2	-1938.4	-1937.4	-1936.8	-1936.0
9	-1945.0	-1948.7	-1945.7	-1944.7	-1944.0	-1943.3

2.3.4 Accuracy

To measure the accuracy of the predicted values, we calculate the Mean absolute scale error (MASE) for each trajectory (incidence and relative transmission rate) generated from the sampling procedure. Incidence trajectories are compared to daily case counts, whereas relative transmission rates are contrasted to mobility indexes. We present the results graphically (violin plots) and numerically (tables). Dotted lines in the plots indicate the performance threshold (1). Values below the unity indicate good performance.

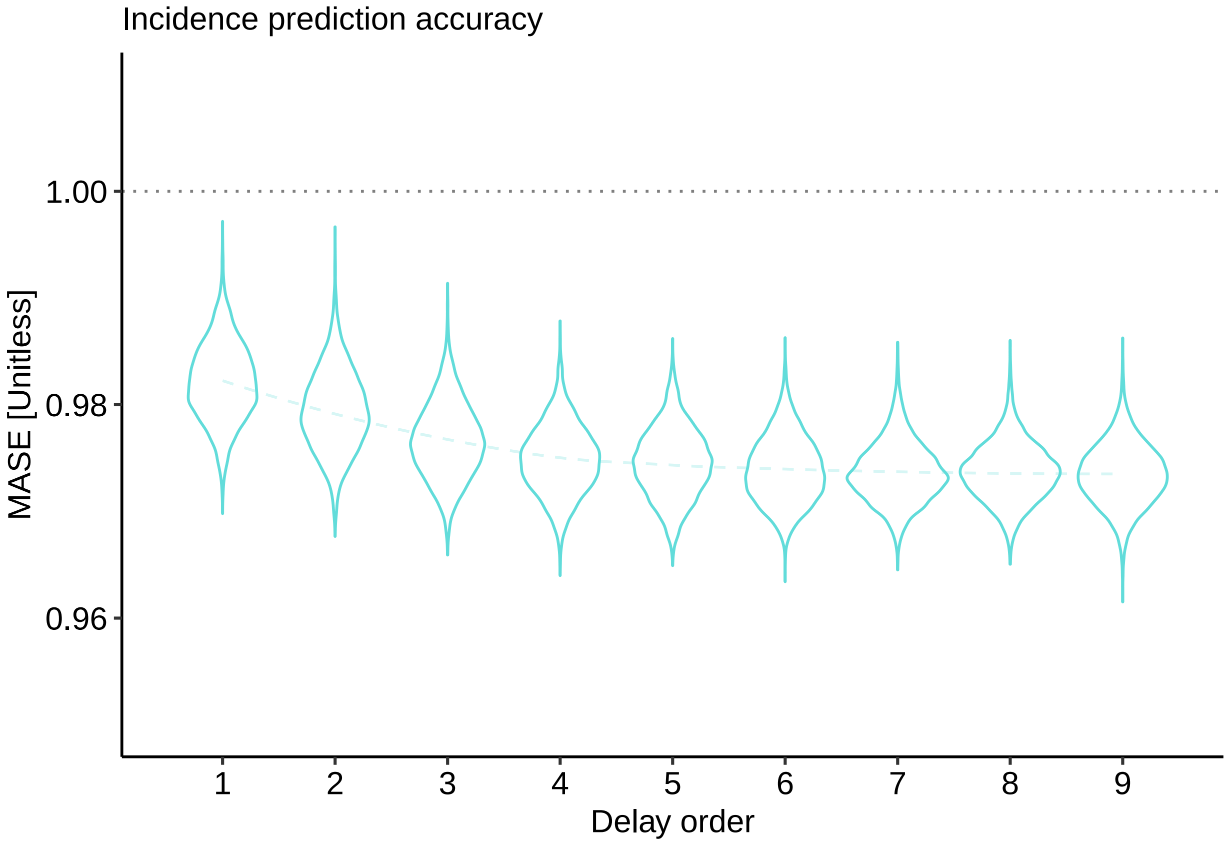

order	mean	q2.5	q25	q50	q75	q97.5
1	0.982	0.975	0.980	0.982	0.985	0.989
2	0.979	0.972	0.977	0.979	0.982	0.987
3	0.977	0.970	0.974	0.976	0.979	0.984
4	0.975	0.969	0.973	0.975	0.977	0.981
5	0.975	0.968	0.972	0.975	0.977	0.981
6	0.974	0.968	0.972	0.974	0.976	0.980
7	0.974	0.968	0.972	0.973	0.975	0.980
8	0.974	0.968	0.972	0.974	0.975	0.979
9	0.973	0.968	0.971	0.973	0.975	0.979

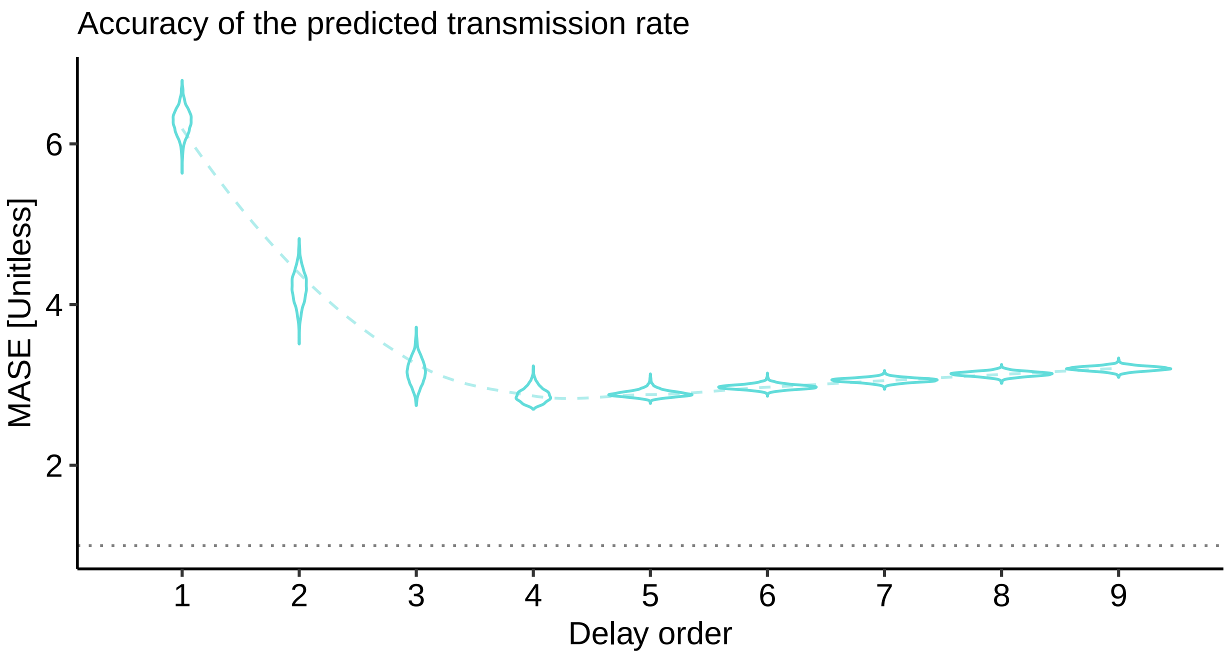

order	mean	q2.5	q25	q50	q75	q97.5
1	6.280	5.944	6.169	6.284	6.389	6.607
2	4.213	3.819	4.077	4.217	4.350	4.588
3	3.172	2.877	3.060	3.168	3.279	3.502
4	2.877	2.738	2.813	2.869	2.930	3.070
5	2.894	2.826	2.864	2.887	2.917	3.002
6	2.976	2.919	2.954	2.974	2.995	3.044
7	3.059	3.003	3.040	3.059	3.078	3.115
8	3.136	3.079	3.116	3.137	3.156	3.195
9	3.204	3.146	3.184	3.203	3.223	3.260

2.4 Posterior distribution

This section summarises the parameter samples obtained from the HMC algorithm. The first summary corresponds to violin plots by parameter and the order of the delay. The second summary corresponds to a table that shows parameter means and standard deviations (in parenthesis) by delay order. Here, we notice that standard deviations are significantly small compared to the average value (mean). In other words, the probability mass is located in a low-volume and high-density region of the parameter space.

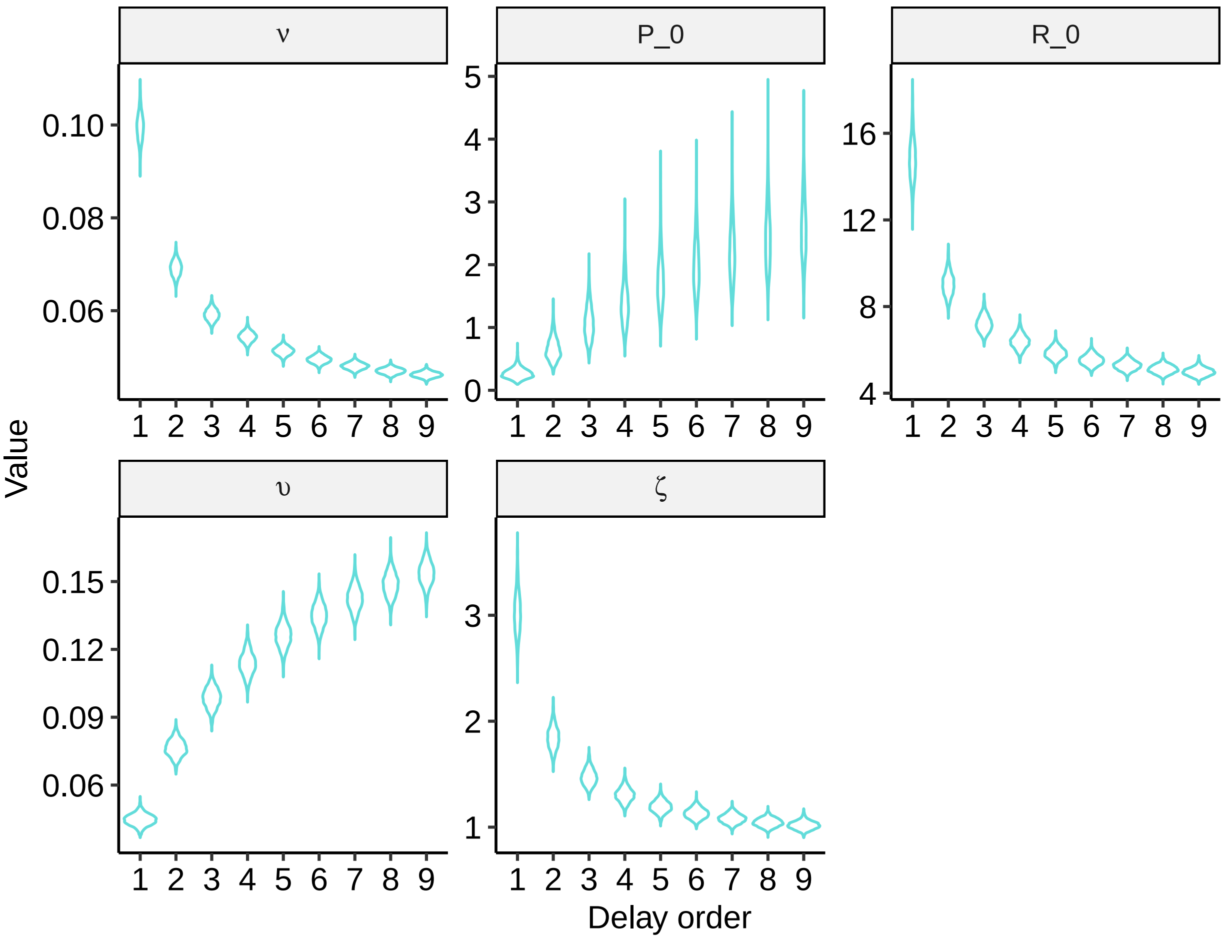

Order	R(0)	ζ	ν	υ	P(0)
1	14.81 (1.020)	3.03 (0.208)	0.10 (0.003)	0.04 (0.003)	0.27 (0.084)
2	9.03 (0.526)	1.85 (0.108)	0.07 (0.002)	0.08 (0.004)	0.66 (0.180)
3	7.21 (0.384)	1.47 (0.079)	0.06 (0.001)	0.10 (0.005)	1.08 (0.273)
4	6.38 (0.335)	1.31 (0.068)	0.05 (0.001)	0.11 (0.005)	1.41 (0.367)
5	5.85 (0.280)	1.20 (0.057)	0.05 (0.001)	0.13 (0.005)	1.75 (0.421)
6	5.53 (0.244)	1.13 (0.050)	0.05 (0.001)	0.14 (0.005)	1.99 (0.445)
7	5.29 (0.220)	1.08 (0.045)	0.05 (0.001)	0.14 (0.005)	2.23 (0.478)
8	5.11 (0.199)	1.04 (0.041)	0.05 (0.001)	0.15 (0.005)	2.44 (0.496)
9	4.98 (0.194)	1.02 (0.040)	0.05 (0.001)	0.15 (0.005)	2.60 (0.524)

2.5 Candidate selection

In the preceding sections, we estimated performance metrics to ascertain which model candidate (delay order) explains the observed dynamics more accurately. On the one hand, metrics of incidence accuracy (MASE) show that increasing the delay order leads to marginal better fits, but it also decreases the log-likelihood. On the other hand, the mobility data's best fit (MASE) occurs when the delay order is equal to **four**.

3 Computational time

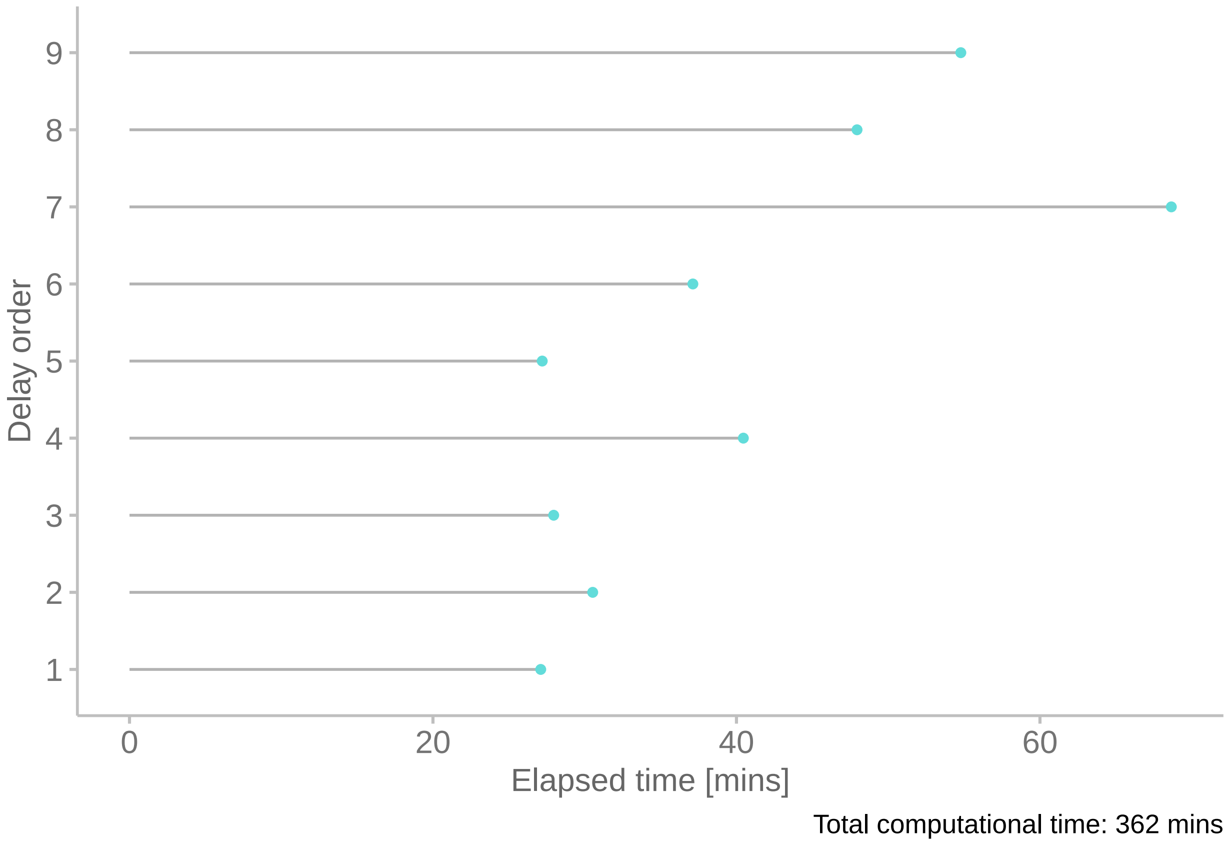

4 Inference (Negative binomial)

4.1 Five unknowns

Misspecification in the measurement model, such as unaccounted overdispersion and unmodelled variability, can lead to overly confident conclusions (Bretó 2018) or biased estimates. In the preceding section, we employed a stringent measurement model (Poisson), which ties the observation mean and variance. In this section, we replace the Poisson model with the *Negative Binomial* (NBin) one, a structure that allows the DGP to handle overdispersion (if present) in the observations. Although the Nbin framework is more flexible, it also increases the DGP's complexity by adding a new parameter: ϕ. The reader should recall that as $\phi \to 0$, NBin converges to the Poisson distribution.

In order to understand these new parameter spaces, we fit the daily incidence data to the nine process model candidates, which are coupled with the NBin observational model. Here, we assume ζ, ν, υ, ϕ and P_0 as unknown parameters. For each model, we run (via Stan) **eight** Markov chains from different starting points.

4.1.1 1st order delay

4.1.1.1 Trace plot

The results indicate that the 1st-order information delay structure coupled with the Nbin measurement model yields a complex bimodal posterior distribution. That is, chains reach either of two equilibrium regions. We support this assessment by the distinct pattern observed in trace plots. Light-coloured chains settle on a high-density (log-likelihood) but low-volume region (narrow-band chains). Conversely, dark-coloured chains settle on a low-density but high-volume region. In addition to this, Stan diagnostics (See Github repository) confirm such pathological behaviour in this parameter space by signalling the occurrence of divergent transitions and abnormal *energies*. Interestingly, Stan only detects divergences and abnormal energy values in the low-density/high-volume region. We thus refer to chains in the high-density region as *well-behaved* chains.

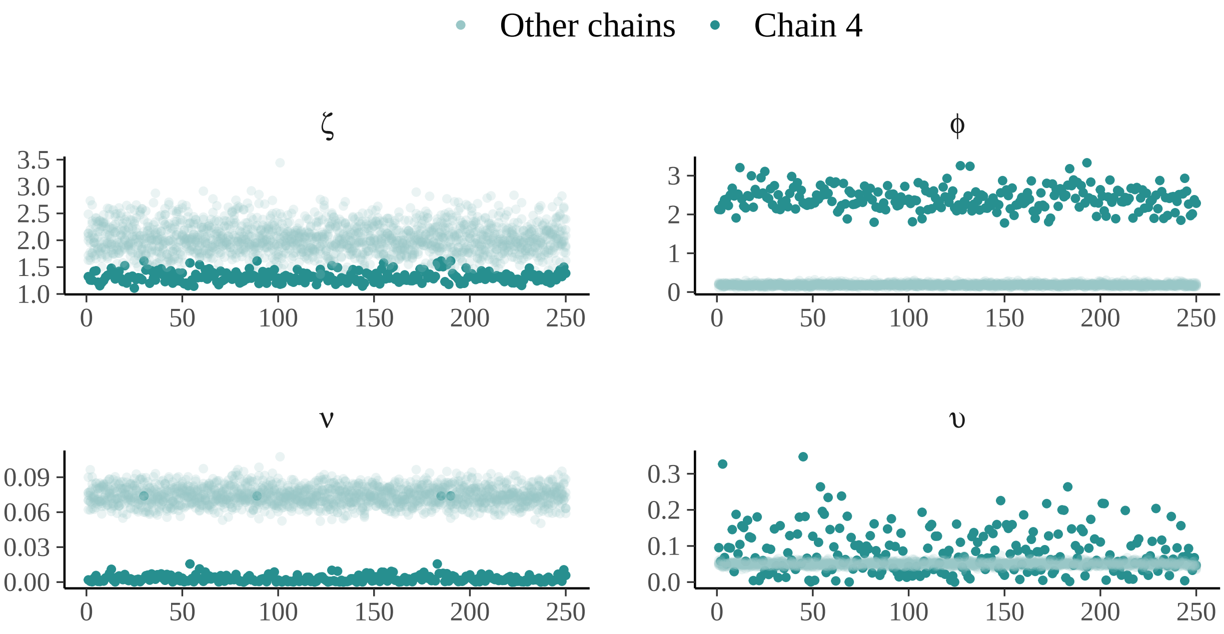

4.1.1.2 Posterior predictive checks

Further, we consider posterior predictive checks as a more immediate appraisal. That is, we compare the predicted incidence against the actual data, discriminating by chain type. Here, it can be seen that, unlike the other chains, samples from Chain 4 do not fit the data.

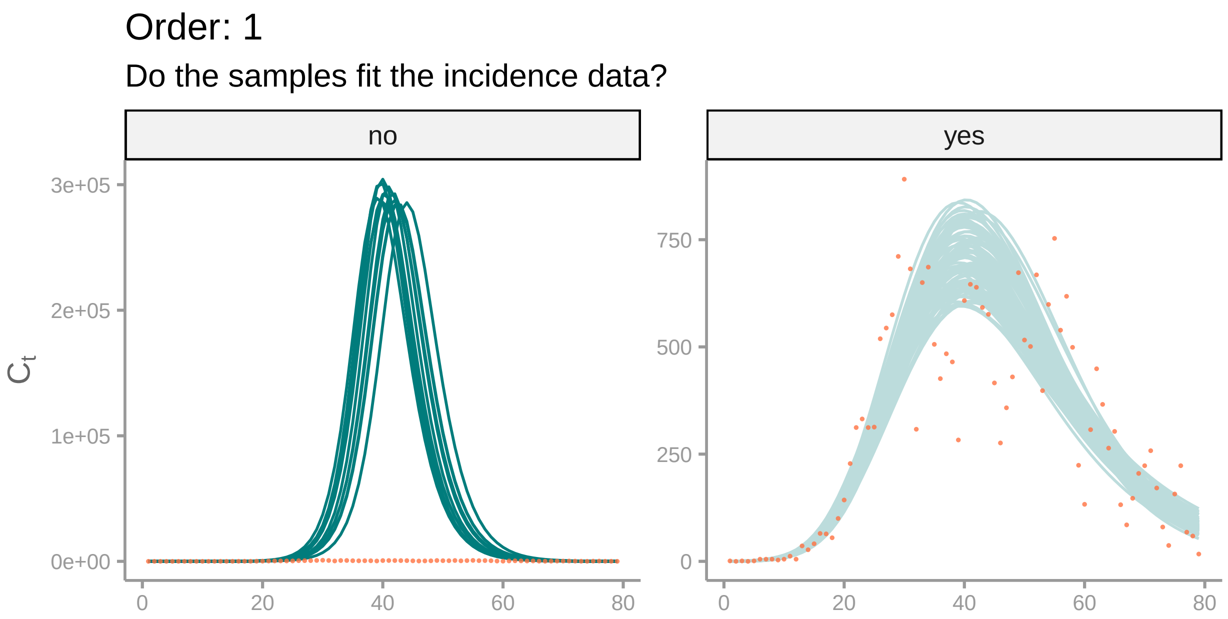

4.1.2 All delay orders

The parameter space of the other models (2nd-order to 9th-order) also exhibit pathological behaviour. Below, we show the posterior distribution by parameter, delay order, and chain via boxplots. In these graphs, we see the clear-cut difference between the two probability mass regions. If we look at parameter ϕ, we notice that chains settle either on low overdispersion (near zero) or high overdispersion values (between 2 and 3). This division also corresponds to high and low-density regions (see log-lik boxplot), respectively.

Variable: ζ

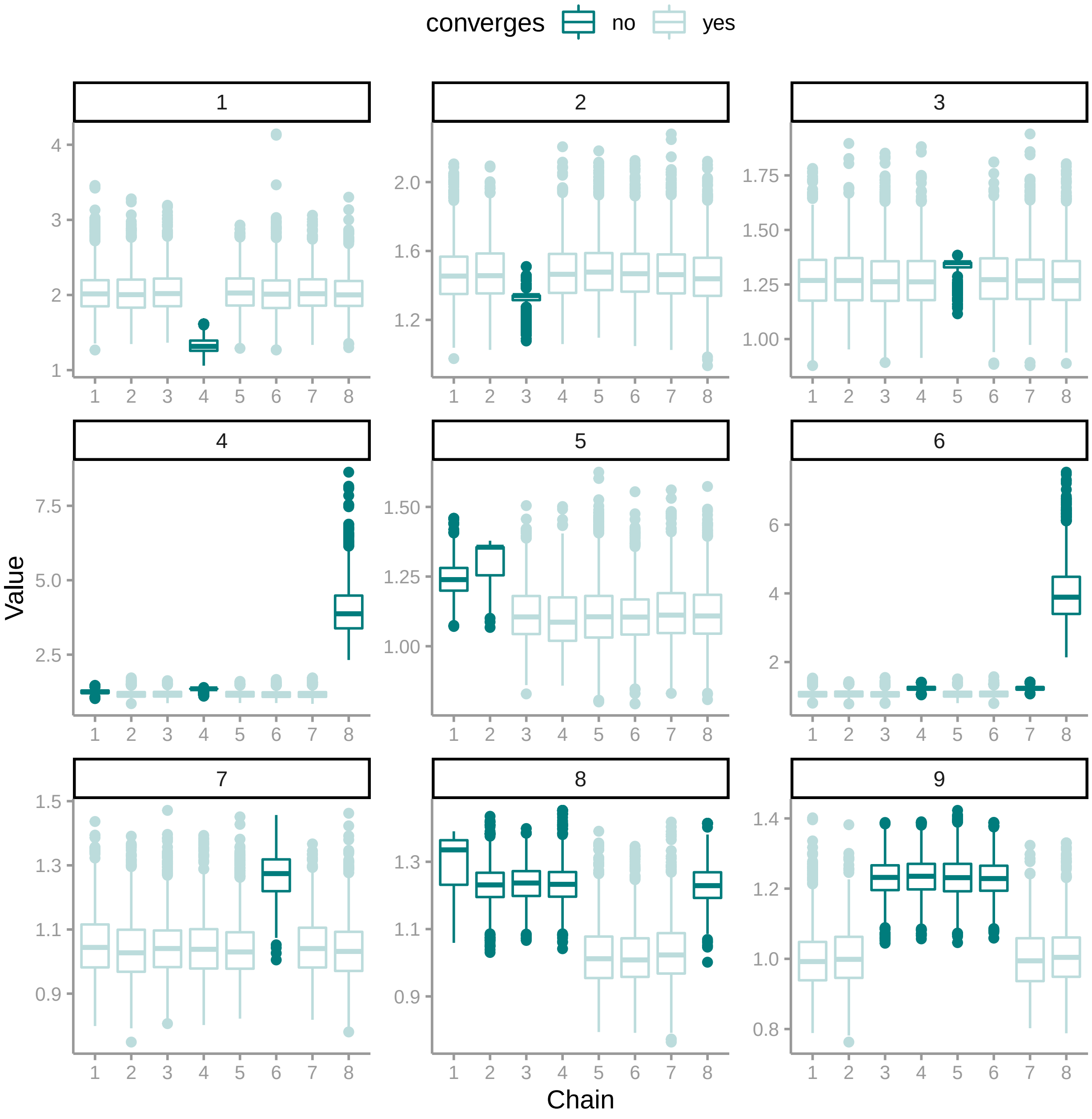

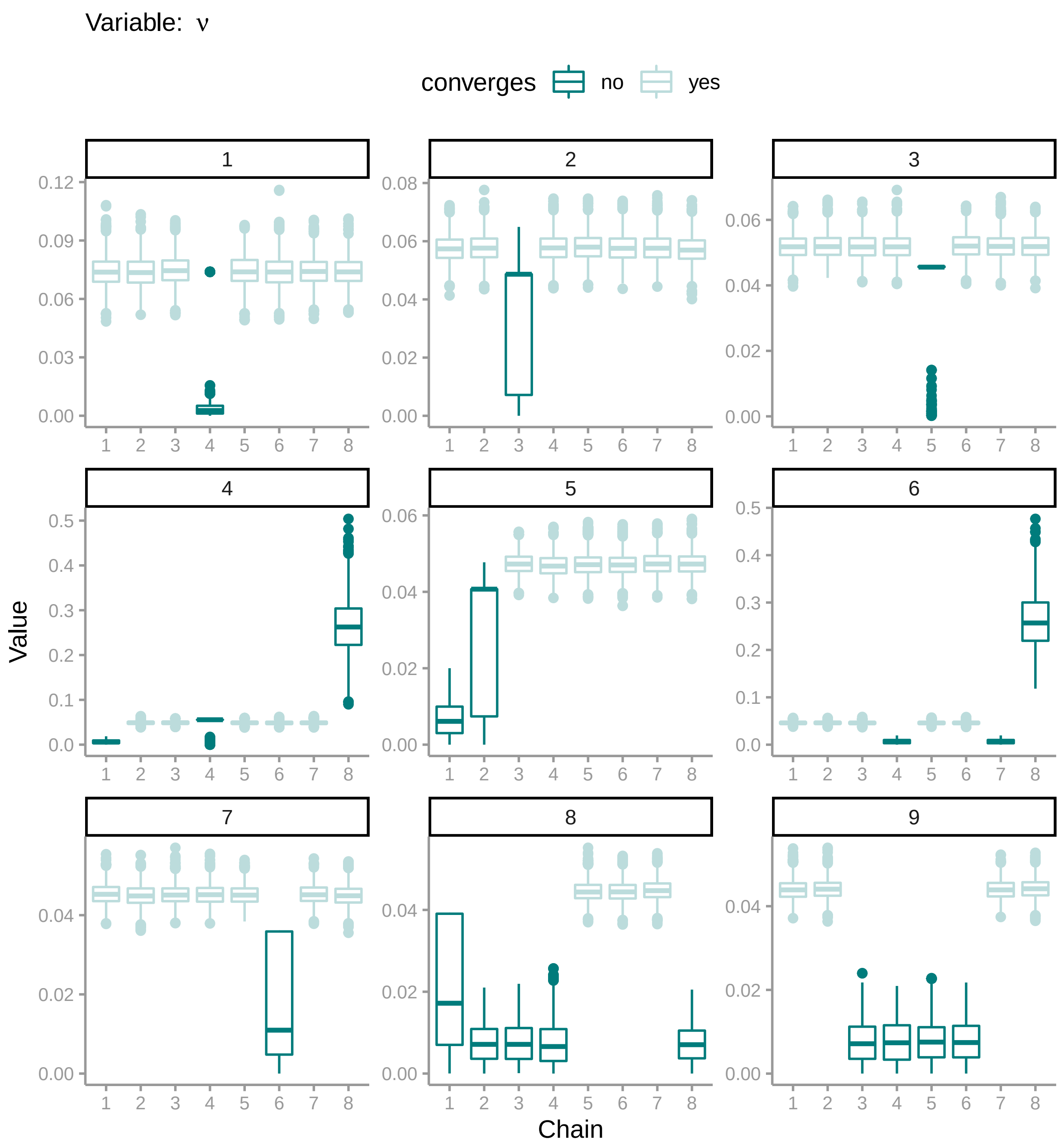
Variable: ν
converges
no
yes
1
2
3
4
5
6
7
8
9
Value
Chain

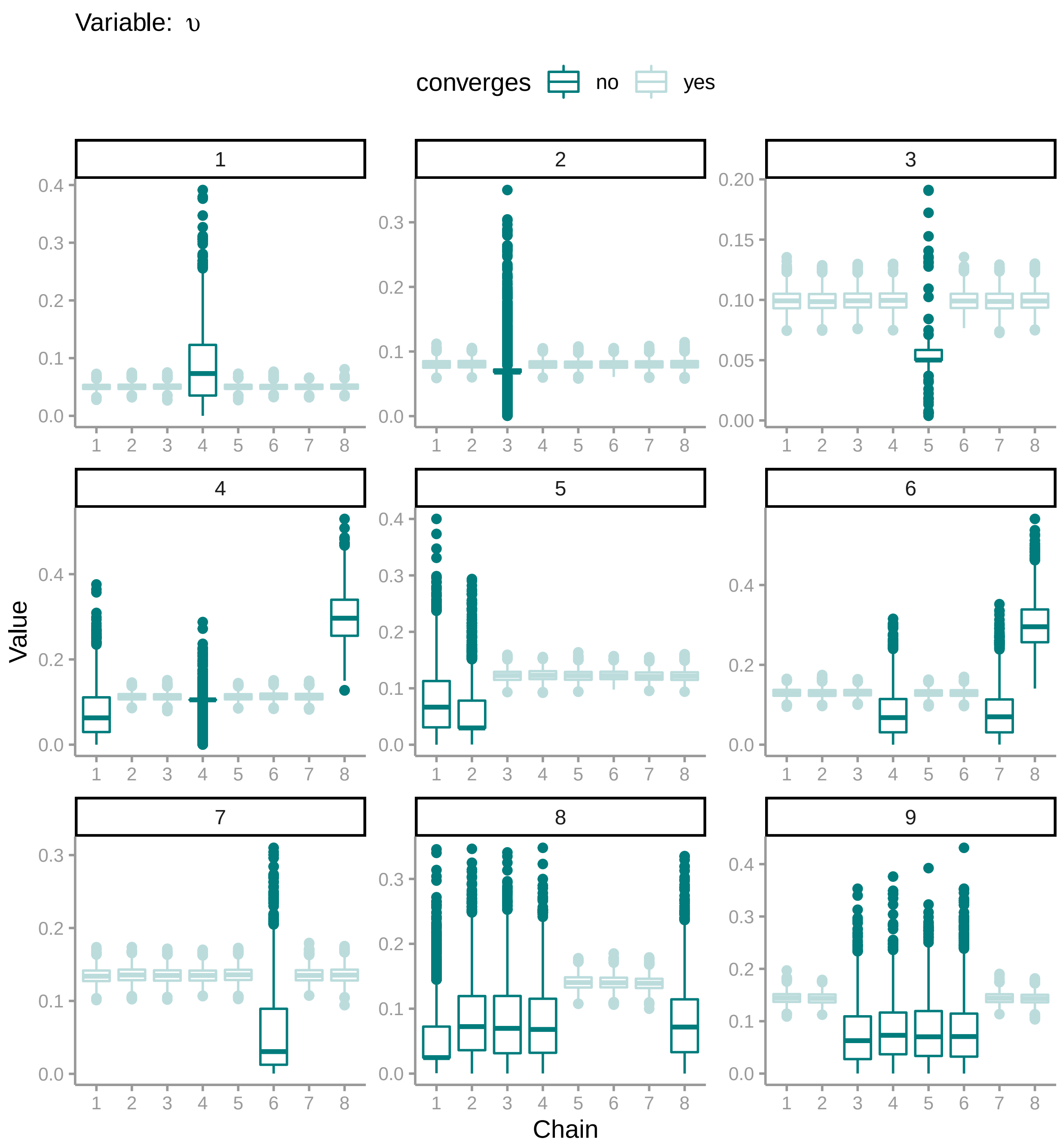
Variable: υ
converges
no
yes
1
2
3
4
5
6
7
8
9
Value
Chain

Variable: ϕ

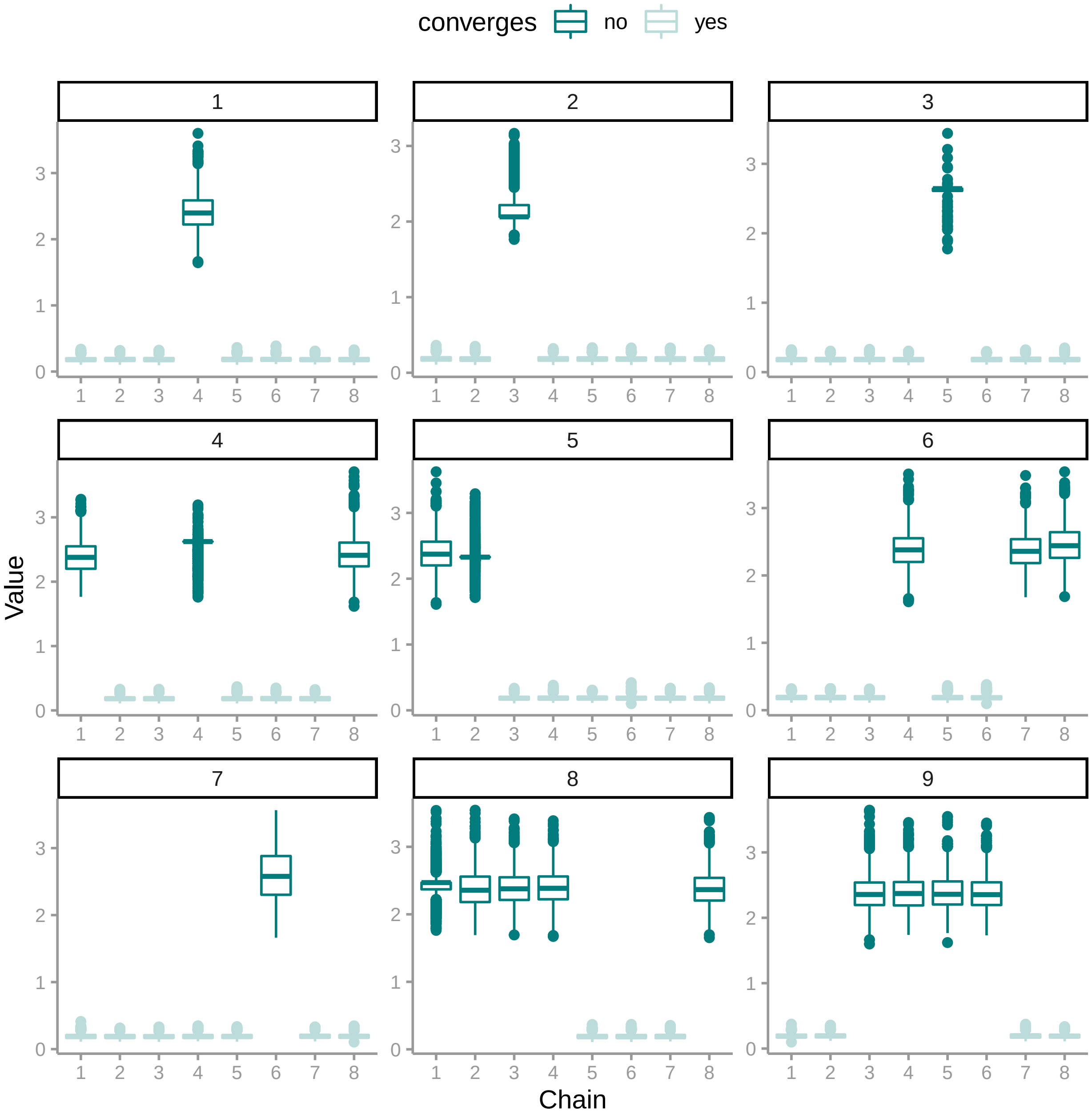

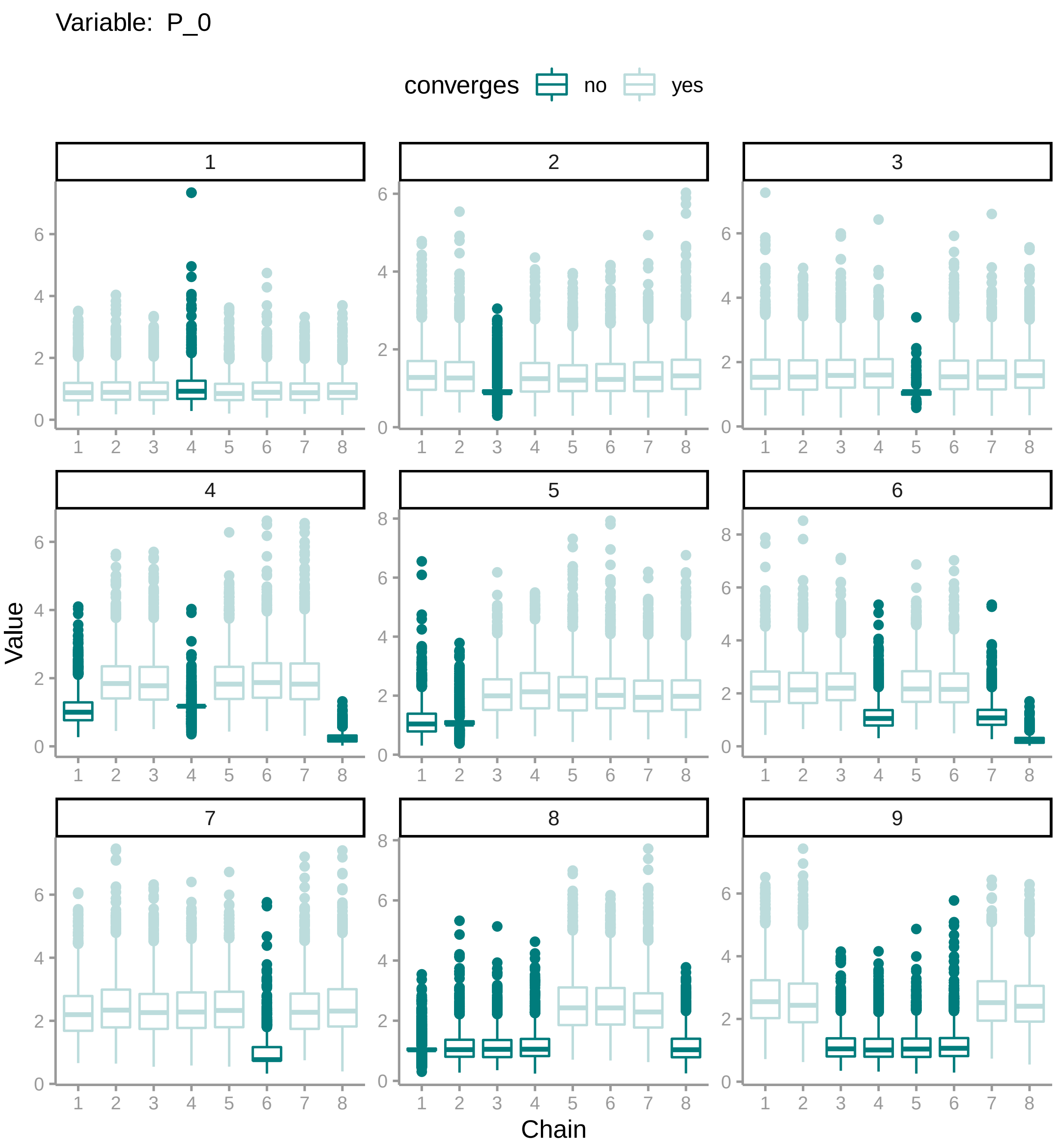
Variable: P_0
converges
no
yes
1
2
3
4
5
6
7
8
9
Value
Chain

Variable: log_lik

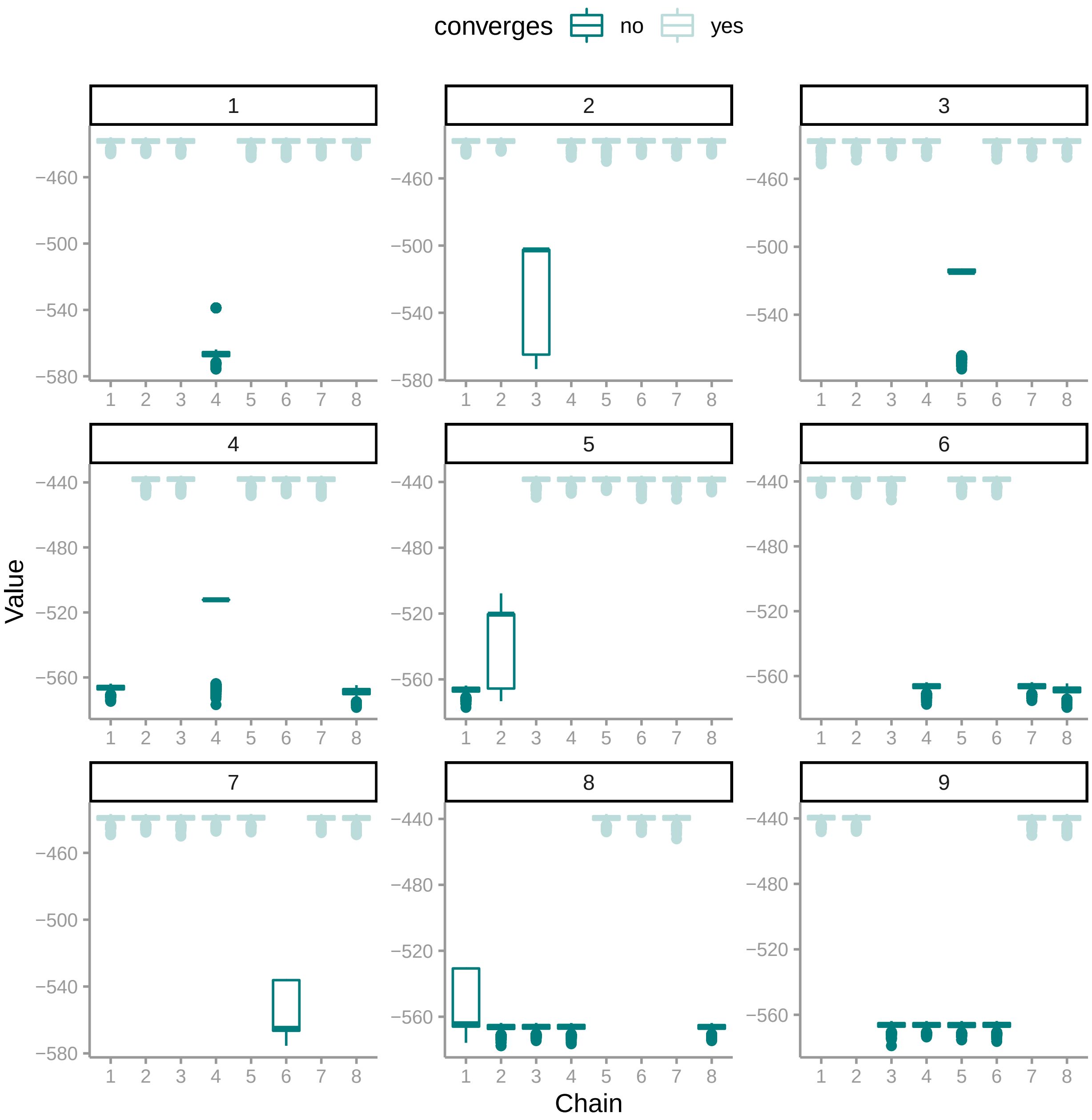

4.1.3 Exploratory estimates

With the purpose of exploring the information provided by high-density regions, we calculate, from the chains that fit the incidence data, summary statistics for the unknown parameters. The table below presents mean values and standard deviations (in parenthesis). When compared to the values calculated from the Poisson distribution (see Section 2.4), we notice similar insights. For instance, both distributions yield notably *thin* estimates for ν & υ, which determine the dynamics of the relative effective contact rate. Similarly, ζ, whose uncertainty contributes to the uncertainty in the effective contact rate, is also narrow, albeit there is a slight bias in estimates from the Poisson distribution, which tend to overestimate $\Re_0$.

It should be remarked that ignoring pathological chains is not a sound approach. Namely, we cannot assume that the parameter space is well-behaved when the evidence tells otherwise. Thus, we employ such estimated values for comparison and exploration purposes rather than for a inference one.

order	R(0)	ζ	ν	υ	P(0)	ϕ
1	9.96 (1.342)	2.04 (0.274)	0.07 (0.008)	0.05 (0.005)	0.97 (0.463)	0.18 (0.033)
2	7.22 (0.832)	1.48 (0.170)	0.06 (0.005)	0.08 (0.007)	1.36 (0.587)	0.18 (0.033)
3	6.24 (0.667)	1.28 (0.136)	0.05 (0.004)	0.10 (0.009)	1.68 (0.708)	0.18 (0.032)
4	5.72 (0.573)	1.17 (0.117)	0.05 (0.003)	0.11 (0.009)	1.95 (0.777)	0.19 (0.032)
5	5.44 (0.520)	1.11 (0.106)	0.05 (0.003)	0.12 (0.010)	2.13 (0.838)	0.19 (0.033)
6	5.23 (0.483)	1.07 (0.099)	0.05 (0.003)	0.13 (0.010)	2.31 (0.874)	0.19 (0.033)
7	5.10 (0.460)	1.04 (0.094)	0.05 (0.003)	0.14 (0.011)	2.41 (0.888)	0.19 (0.034)
8	5.01 (0.451)	1.02 (0.092)	0.04 (0.003)	0.14 (0.011)	2.51 (0.940)	0.19 (0.035)
9	4.91 (0.417)	1.00 (0.085)	0.04 (0.002)	0.14 (0.011)	2.61 (0.925)	0.19 (0.034)

4.1.4 Exploratory predicted relative contact rate

From the well-behaved chains, we also estimate the predicted relative effective contact rate.

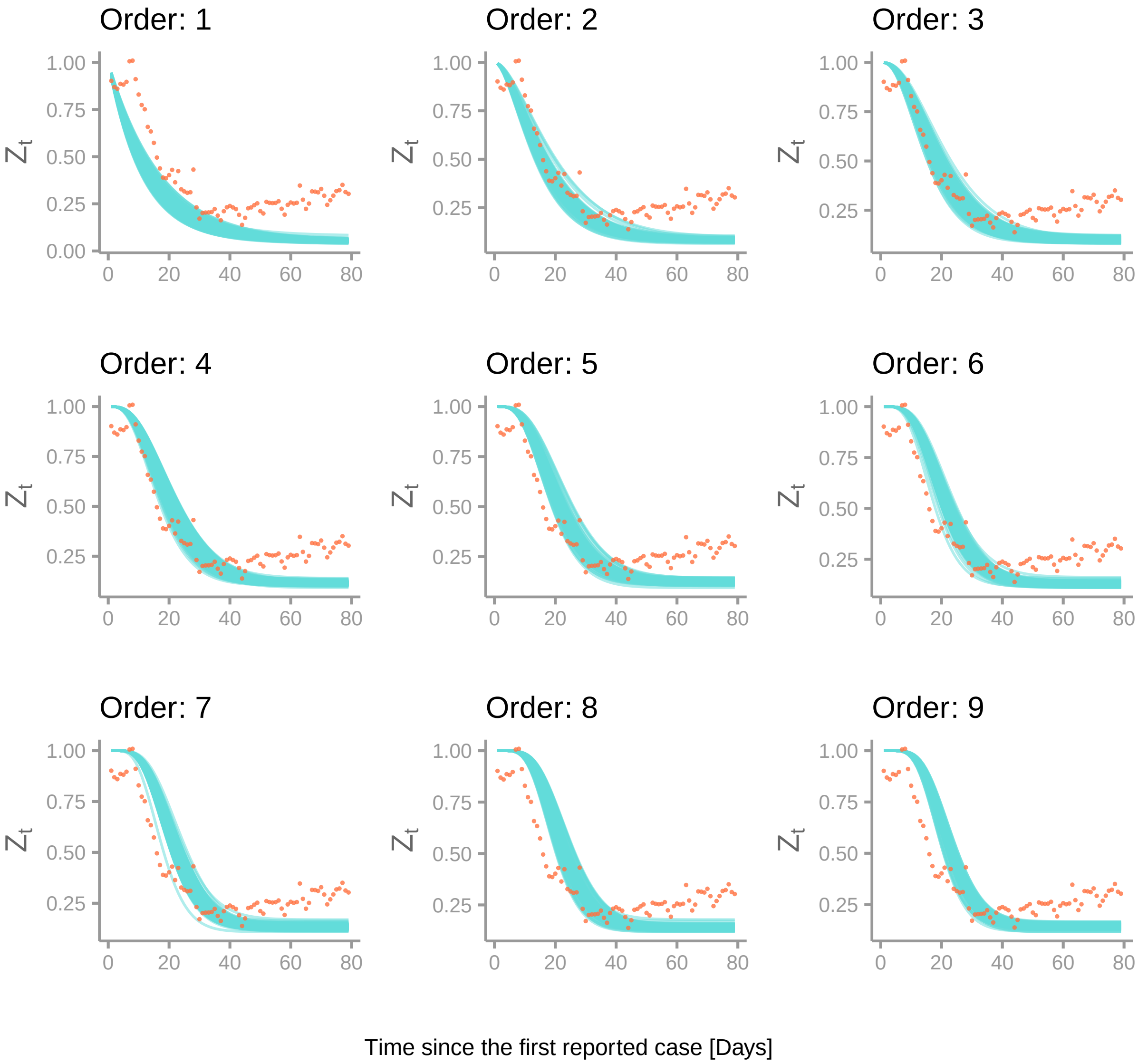

We see that the negative binomial (in this particular region of the parameter space) yields slightly thicker uncertainty intervals in the 4th-order model in comparison to those generated by the Poisson distribution.

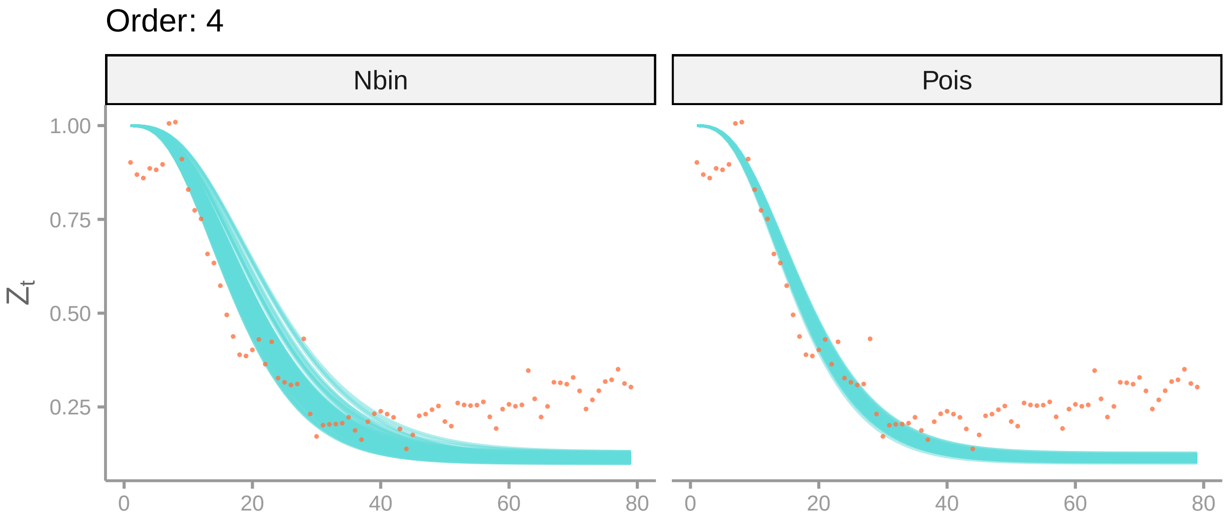

4.1.5 Exploratory predicted effective contact rate

Here, we compare the predicted $\Re_t$ from Section 1 (Poisson), and that from the well-behaved chains in the Nbin distribution. As expected, the Nbin offers more flexibility than the Poisson distribution, mainly for the two first weeks, but overall both predictions convey similar information. In other words, the use of the Poisson distribution do not significantly compromise the results. Based on these results, we conjecture that the constraint on narrow uncertainty intervals stems from the effective contact rate's particular deterministic formulation rather than the choice of the measurement model.

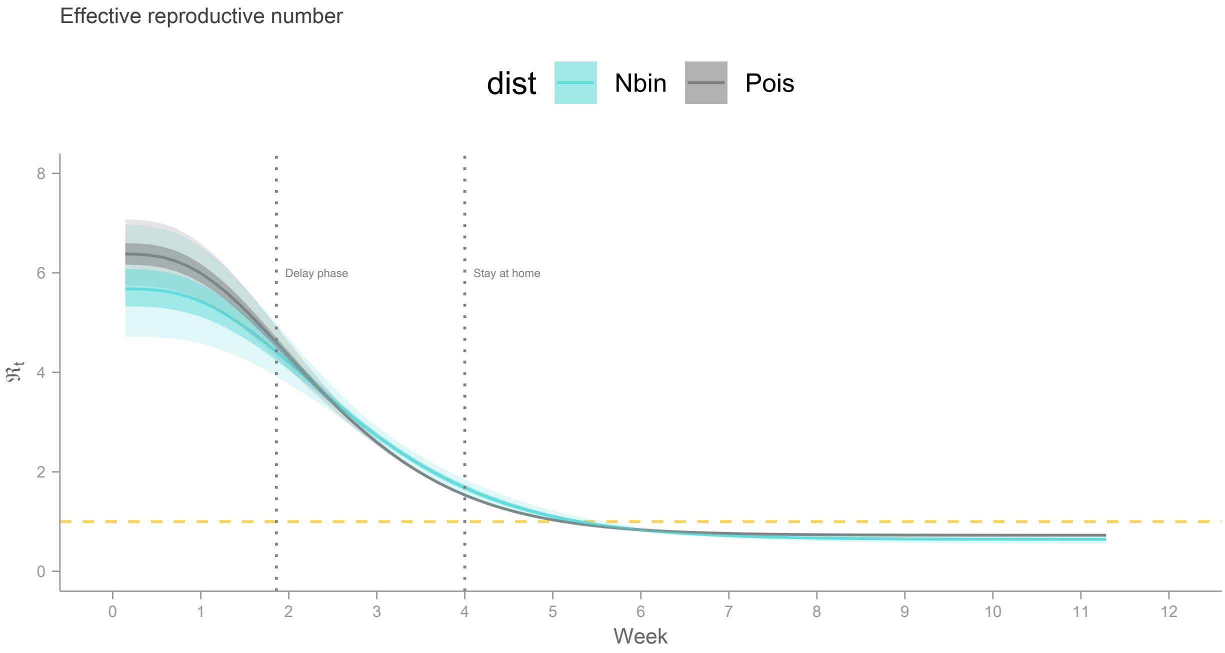

4.2 Only one unknown

To provide further evidence of the complexity generated by the Nbin distribution, we identify that even a single parameter can create bimodality in the 1st-order delay model. To illustrate this finding, we assume as unmodelled predictors or known values, the mean values of the well-behaved chains (Section 4.1.3). We do so for the parameters $P(0)$, υ, ν, and ϕ (see table below), leaving ζ as the only unknown. We also show ζ estimates from the well-behaved chains.

4.2.1 Trace plots

This experiment shows that even leaving *zeta* as the only unknown, the bimodality persists.

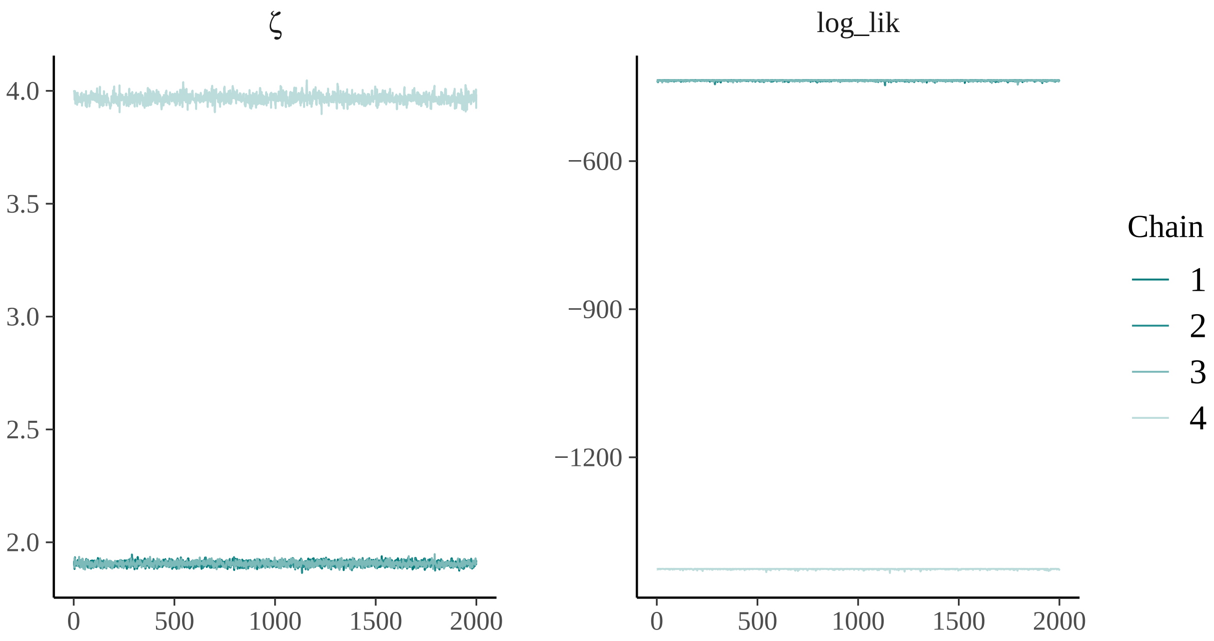

4.3 Two specific unknowns

After several attempts and strategies to find a robust model parameterisation that achieves convergence, we identify that leaving P_0 and ϕ as unknowns yield well-behaved chains, irrespectively of particular starting points and algorithm tuning. Nevertheless, this strategy assumes that ζ, ν and υ are known quantities, which we fix at the values found above (Section 4.1.3). In consequence, this can be seen as another exploratory exercise (instead of inference), considering that we employ the data twice.

4.3.1 Expected values

4.3.1.1 Predicted incidence compared to daily case counts

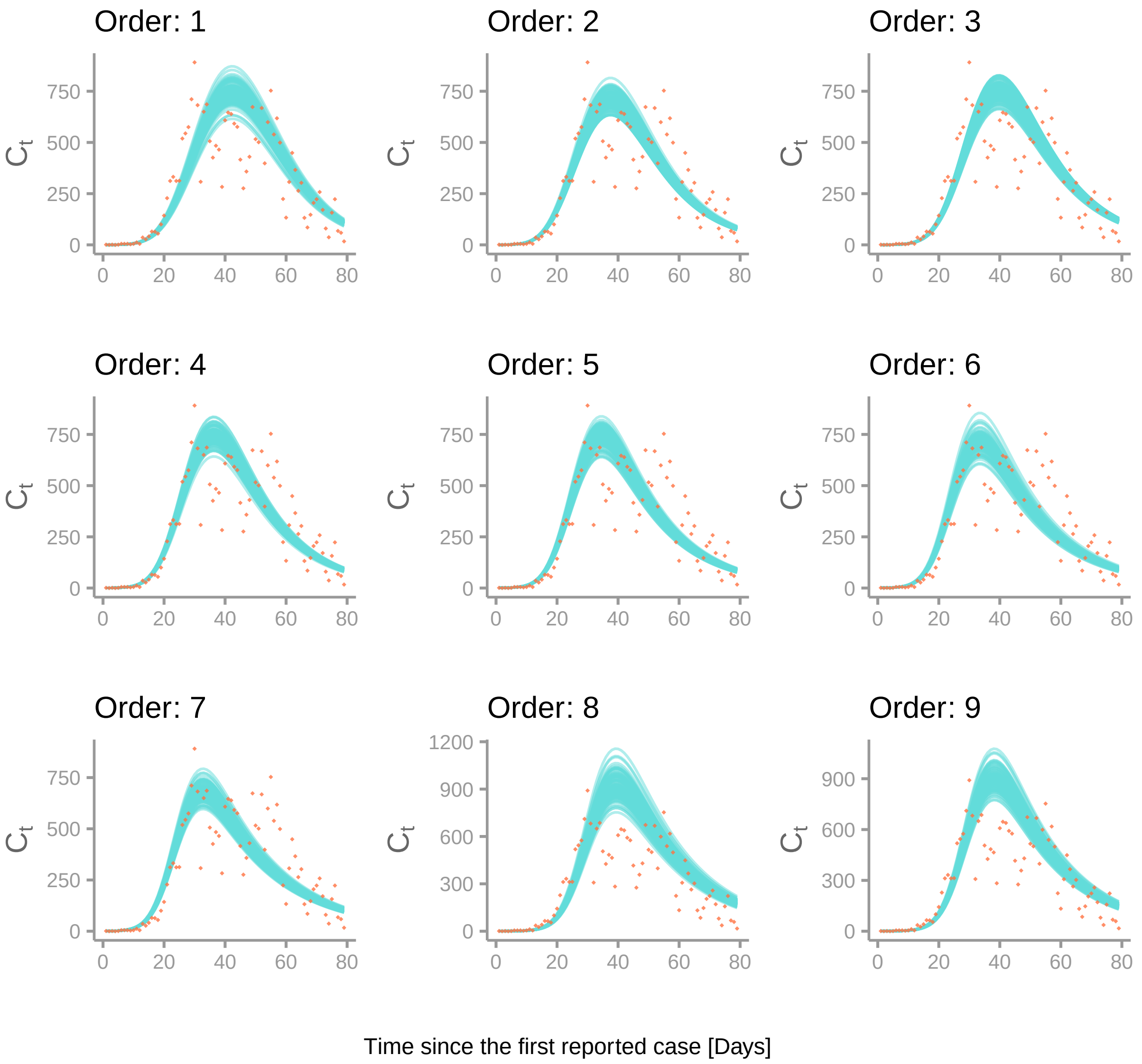

4.3.1.2 Predicted relative contact rate compared to mobility indexes

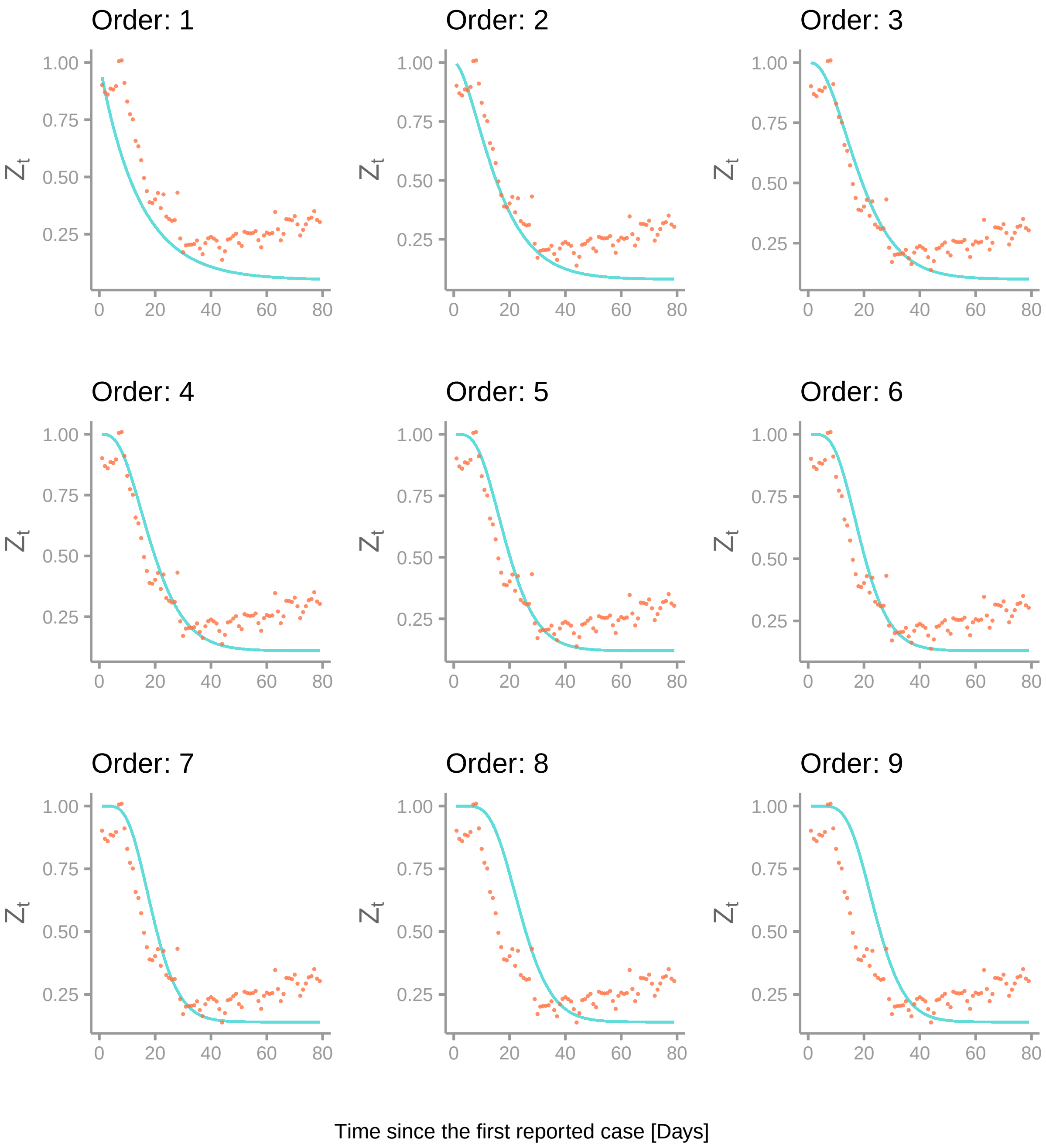

4.3.2 Likelihood

Likelihood values suggest preference for 2nd, 3rd and **4th** (highest) order delay models.

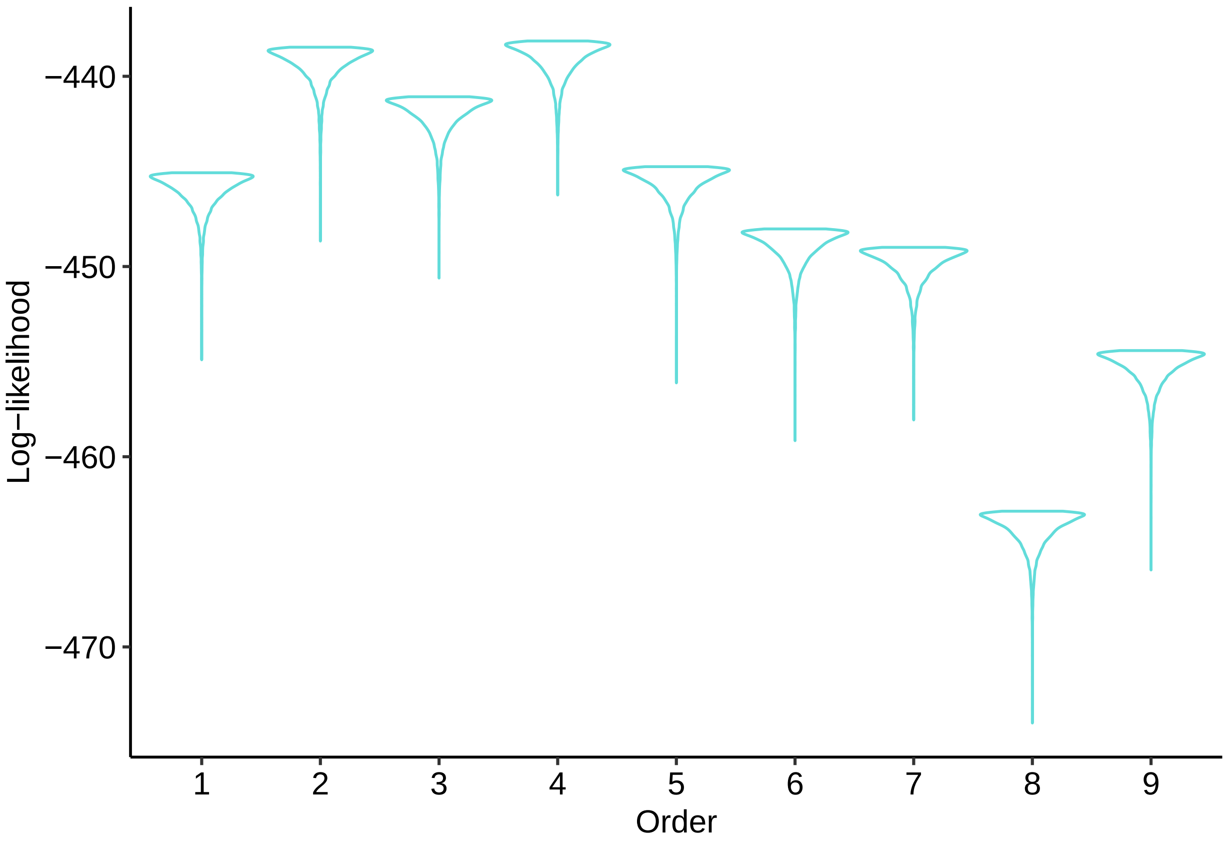

order	mean	q2.5	q25	q50	q75	q97.5
1	-446.0	-448.6	-446.4	-445.8	-445.4	-445.1
2	-439.5	-442.2	-439.8	-439.1	-438.8	-438.5
3	-442.1	-444.8	-442.4	-441.8	-441.4	-441.1
4	-439.1	-441.8	-439.5	-438.8	-438.4	-438.2
5	-445.7	-448.3	-446.1	-445.4	-445.0	-444.8
6	-449.0	-451.6	-449.4	-448.7	-448.3	-448.0
7	-450.0	-452.6	-450.3	-449.7	-449.3	-449.0
8	-463.9	-466.5	-464.2	-463.5	-463.1	-462.9
9	-455.4	-458.0	-455.8	-455.1	-454.7	-454.4

4.3.3 Accuracy

4.3.3.1 Incidence

The incidence MASE suggests that the 1st, 8th and 9th order delay structures do not yield accurate incidence predictions.

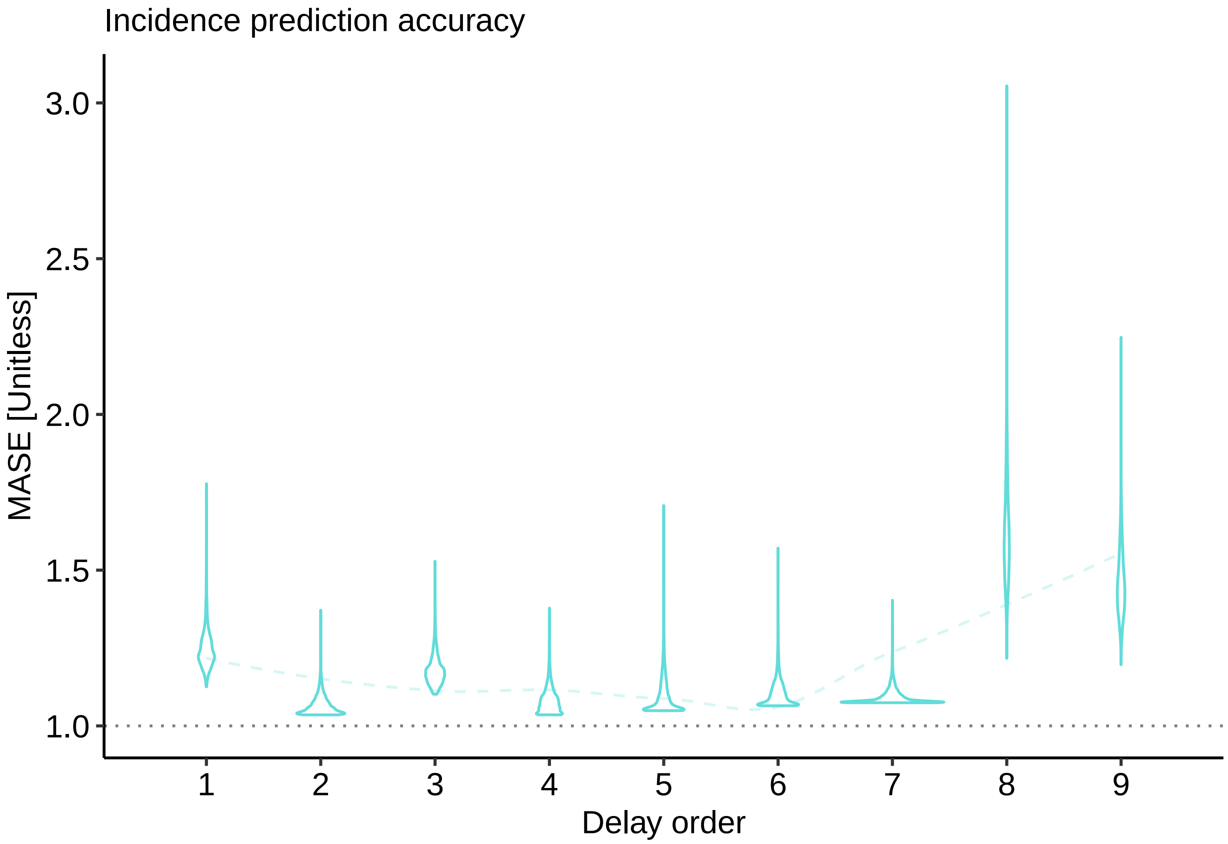

order	mean	q2.5	q25	q50	q75	q97.5
1	1.2	1.2	1.2	1.2	1.3	1.4
2	1.1	1.0	1.0	1.1	1.1	1.2
3	1.2	1.1	1.1	1.2	1.2	1.3
4	1.1	1.0	1.1	1.1	1.1	1.2
5	1.1	1.0	1.1	1.1	1.1	1.3
6	1.1	1.1	1.1	1.1	1.1	1.2
7	1.1	1.1	1.1	1.1	1.1	1.2
8	1.6	1.4	1.5	1.6	1.7	2.0
9	1.5	1.3	1.4	1.4	1.5	1.7

4.3.3.2 Mobility

We reach convergence at the expense of fixing some parameters. In particular, by letting υ and ν fixed to a point estimate, the predicted relative contact rate (Z_t) is also a point estimate at each time t. Based on these point estimates, the 3rd, **4th**, 5th, 6th, and 7th order delays yield the most accurate trajectories (compared to mobility data).

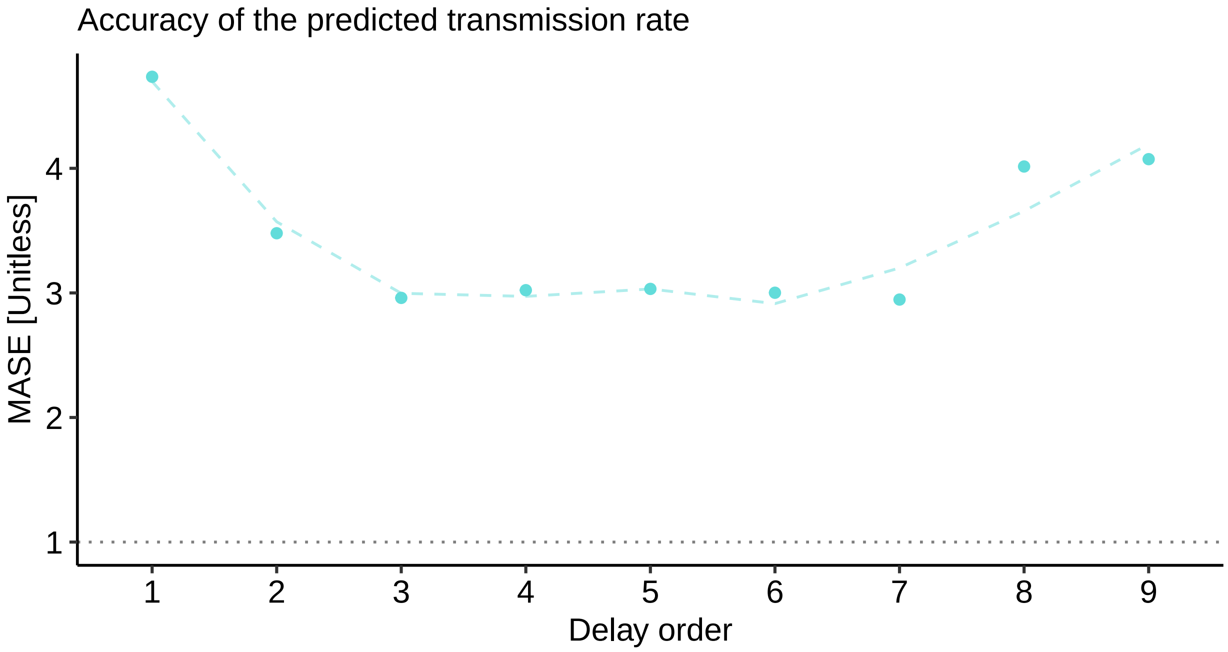

Order	MASE
1	4.7
2	3.5
3	3.0
4	3.0
5	3.0
6	3.0
7	2.9
8	4.0
9	4.1

4.3.4 Posterior distribution

The following table summarises parameter estimates from the samples obtained through the HMC algorithm. Specifically, we show mean values and standard deviations (in parenthesis).

Order	R(0)	ζ	ν	υ	P(0)	ϕ
1	9.98 (0)	2.04 (0)	0.07 (0)	0.05 (0)	0.52 (0.03)	0.22 (0.04)
2	7.24 (0)	1.48 (0)	0.06 (0)	0.08 (0)	1.57 (0.08)	0.19 (0.03)
3	6.26 (0)	1.28 (0)	0.05 (0)	0.1 (0)	1.08 (0.06)	0.2 (0.04)
4	5.72 (0)	1.17 (0)	0.05 (0)	0.11 (0)	2.18 (0.12)	0.19 (0.03)
5	5.43 (0)	1.11 (0)	0.05 (0)	0.12 (0)	3.11 (0.18)	0.22 (0.04)
6	5.23 (0)	1.07 (0)	0.05 (0)	0.13 (0)	3.76 (0.22)	0.24 (0.04)
7	5.09 (0)	1.04 (0)	0.05 (0)	0.14 (0)	4.19 (0.25)	0.25 (0.04)
8	4.99 (0)	1.02 (0)	0.04 (0)	0.14 (0)	1.18 (0.09)	0.34 (0.06)
9	4.89 (0)	1 (0)	0.04 (0)	0.14 (0)	1.4 (0.09)	0.28 (0.05)

Further, the table below demonstrates that the estimated means (from this exploratory exercise) are similar to those obtained from the inference process carried out through the Poisson distribution (in parenthesis).

Order	R(0)	ζ	ν	υ	P(0)	ϕ
1	9.98 (14.81)	2.04 (3.03)	0.07 (0.10)	0.05 (0.04)	0.52 (0.27)	0.22 (0)
2	7.24 (9.03)	1.48 (1.85)	0.06 (0.07)	0.08 (0.08)	1.57 (0.66)	0.19 (0)
3	6.26 (7.21)	1.28 (1.47)	0.05 (0.06)	0.1 (0.10)	1.08 (1.08)	0.2 (0)
4	5.72 (6.38)	1.17 (1.31)	0.05 (0.05)	0.11 (0.11)	2.18 (1.41)	0.19 (0)
5	5.43 (5.85)	1.11 (1.20)	0.05 (0.05)	0.12 (0.13)	3.11 (1.75)	0.22 (0)
6	5.23 (5.53)	1.07 (1.13)	0.05 (0.05)	0.13 (0.14)	3.76 (1.99)	0.24 (0)
7	5.09 (5.29)	1.04 (1.08)	0.05 (0.05)	0.14 (0.14)	4.19 (2.23)	0.25 (0)
8	4.99 (5.11)	1.02 (1.04)	0.04 (0.05)	0.14 (0.15)	1.18 (2.44)	0.34 (0)
9	4.89 (4.98)	1 (1.02)	0.04 (0.05)	0.14 (0.15)	1.4 (2.60)	0.28 (0)

5 Prediction of hidden states

Based on the results above, we select the 4th-order information delay structure with a Poisson measurement model as DGP3.

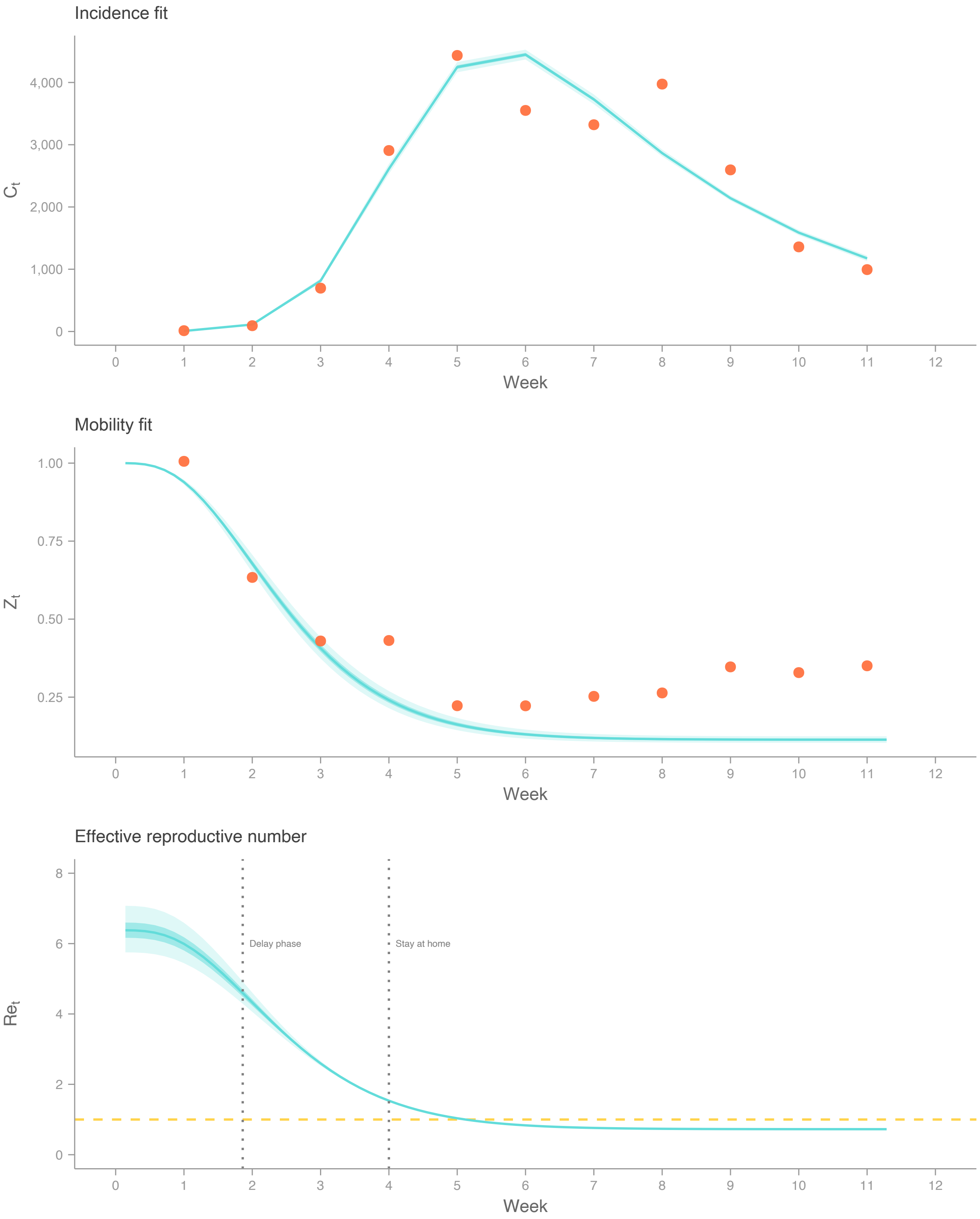

6 Original Computing Environment

```
## R version 4.1.0 (2021-05-18)
## Platform: x86_64-apple-darwin17.0 (64-bit)
## Running under: macOS Big Sur 10.16
##
## Matrix products: default
## BLAS:   /Library/Frameworks/R.framework/Versions/4.1/Resources/lib/libRblas.dylib
## LAPACK: /Library/Frameworks/R.framework/Versions/4.1/Resources/lib/libRlapack.dylib
##
## locale:
## [1] en_IE.UTF-8/en_IE.UTF-8/en_IE.UTF-8/C/en_IE.UTF-8/en_IE.UTF-8
##
## attached base packages:
## [1] stats     graphics  grDevices utils     datasets  methods   base
##
## other attached packages:
##  [1] scales_1.1.1        patchwork_1.1.1      ggrepel_0.9.1
##  [4] ggpubr_0.4.0        ggalt_0.4.0          GGally_2.1.1
##  [7] tidyr_1.1.3         tictoc_1.0.1         stringr_1.4.0
## [10] rstan_2.21.2        ggplot2_3.3.5        StanHeaders_2.21.0-7
## [13] reshape2_1.4.4      readxl_1.3.1         readsdr_0.2.0.9000
## [16] readr_2.0.1         purrr_0.3.4          posterior_0.1.5
## [19] Metrics_0.1.4       lubridate_1.7.10     kableExtra_1.3.4
## [22] imputeTS_3.2        extraDistr_1.9.1     dplyr_1.0.9
## [25] cmdstanr_0.4.0      bayesplot_1.8.0
##
## loaded via a namespace (and not attached):
##   [1] backports_1.2.1     systemfonts_1.0.2   plyr_1.8.6
##   [4] splines_4.1.0       inline_0.3.19       digest_0.6.27
##   [7] htmltools_0.5.2     fansi_0.5.0         magrittr_2.0.3
##  [10] checkmate_2.0.0     tzdb_0.1.2          openxlsx_4.2.3
##  [13] extrafont_0.17      RcppParallel_5.1.4  matrixStats_0.59.0
##  [16] vroom_1.5.4         xts_0.12.1          extrafontdb_1.0
##  [19] svglite_2.0.0       forecast_8.15       tseries_0.10-48
##  [22] prettyunits_1.1.1   colorspace_2.0-1    rvest_1.0.0
##  [25] haven_2.4.1         xfun_0.30           callr_3.7.0
##  [28] crayon_1.4.1        jsonlite_1.7.2      zoo_1.8-9
##  [31] glue_1.6.2          gtable_0.3.0        webshot_0.5.2
##  [34] proj4_1.0-10.1      V8_3.4.2            distributional_0.2.2
##  [37] car_3.0-10          pkgbuild_1.2.0      Rttf2pt1_1.3.8
##  [40] quantmod_0.4.18     maps_3.3.0          abind_1.4-5
##  [43] stinepack_1.4       DBI_1.1.1           rstatix_0.7.0
##  [46] Rcpp_1.0.6          viridisLite_0.4.0   gridtext_0.1.4
##  [49] foreign_0.8-81      bit_4.0.4           stats4_4.1.0
##  [52] httr_1.4.2          RColorBrewer_1.1-2  ellipsis_0.3.2
##  [55] pkgconfig_2.0.3     reshape_0.8.8       loo_2.4.1
##  [58] farver_2.1.0        nnet_7.3-16         utf8_1.2.1
##  [61] tidyselect_1.1.1    labeling_0.4.2      rlang_1.0.2
##  [64] munsell_0.5.0       cellranger_1.1.0    tools_4.1.0
##  [67] cli_3.3.0           generics_0.1.0      broom_0.7.6
##  [70] ggridges_0.5.3      evaluate_0.15       fastmap_1.1.0
##  [73] yaml_2.2.1          processx_3.5.2      knitr_1.39
##  [76] bit64_4.0.5         zip_2.2.0           nlme_3.1-152
```

```
## [79] ash_1.0-15          xml2_1.3.2          compiler_4.1.0
## [82] rstudioapi_0.13     curl_4.3.1          ggsignif_0.6.1
## [85] tibble_3.1.6        stringi_1.6.2       highr_0.9
## [88] ps_1.6.0            forcats_0.5.1       lattice_0.20-44
## [91] Matrix_1.3-3        tensorA_0.36.2      urca_1.3-0
## [94] vctrs_0.4.1         pillar_1.7.0        lifecycle_1.0.1
## [97] lmtest_0.9-38       data.table_1.14.0   R6_2.5.0
## [100] KernSmooth_2.23-20 gridExtra_2.3       rio_0.5.26
## [103] codetools_0.2-18   MASS_7.3-54         withr_2.4.2
## [106] fracdiff_1.5-1     mgcv_1.8-35         parallel_4.1.0
## [109] hms_1.1.0          ggtext_0.1.1        quadprog_1.5-8
## [112] grid_4.1.0         timeDate_3043.102   rmarkdown_2.13
## [115] carData_3.0-4      TTR_0.24.2
```

References

Bretó, Carles. 2018. "Modeling and Inference for Infectious Disease Dynamics: A Likelihood-Based Approach." *Statistical Science* 33 (1): 57–69. https://doi.org/10.1214/17-STS636.
