# Supplementary material for: Inferring the effective reproductive number from deterministic and semi-deterministic compartmental models using incidence and mobility data
Source: PLoS Comput Biol. 2022 Jun 27;18(6):e1010206. doi: 10.1371/journal.pcbi.1010206 (PMC9269962; doi:10.1371/journal.pcbi.1010206)

S8 Appendix

This appendix illustrates the comparison analysis performed on the output generated by the Data Generating Processes studied in this work.

Contents

1 Comparison of DGP1's candidate measurement models **2**
1.1 Incidence 2
1.2 Relative effective contact rate 3
1.3 Effective reproduction number 4
1.4 Parameter estimates 5

2 Simulations from MLE **6**

3 Hidden states by DGP **7**

1 Comparison of DGP1's candidate measurement models

This section shows the comparison among the inferences produced by DGP1's candidate models 2, 5, and 6. The reader can find the specification of each model in S1.

1.1 Incidence

Recall that models 5 and 6 account for weekly incidence data. The difference between these two models is that model 6 links the relative contact rate to Apple's driving indexes. The graph below shows that both models yield similar fits to the weekly number of new cases. That is, incorporating mobility data into model 6 does not compromise its accuracy in predicting the incidence rate.

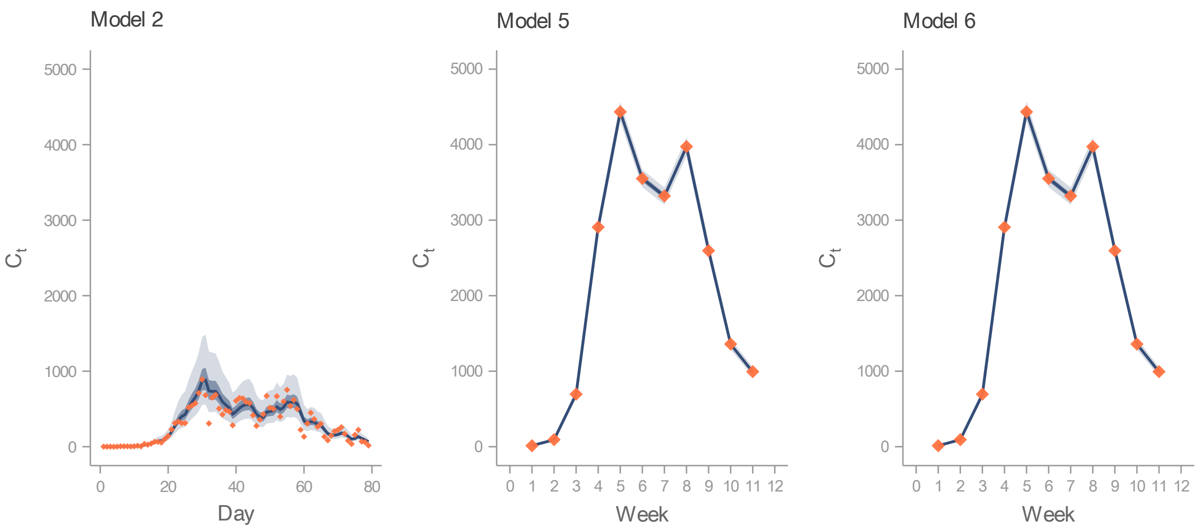

1.2 Relative effective contact rate

Regarding the relative effective contact rate (Z_t), Models 2 and 5 convey similar uncertainties. This result implies that changing the periodicity in the measurement model (from daily to weekly) does not translate into a loss of information. However, accounting for mobility data in the measurement model has an effect on the predictions generated by Model 6. Specifically, the uncertainty in (Z_t) is smaller than that estimated by models 2 and 5.

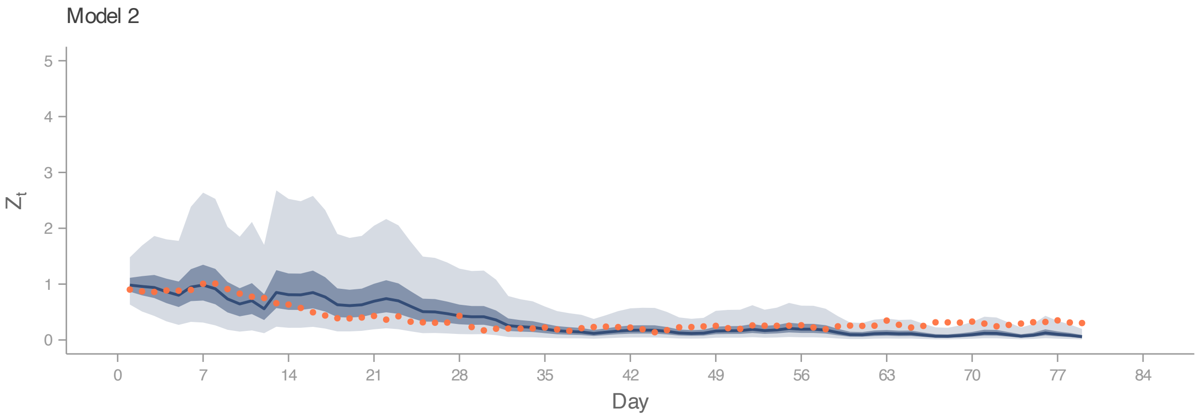

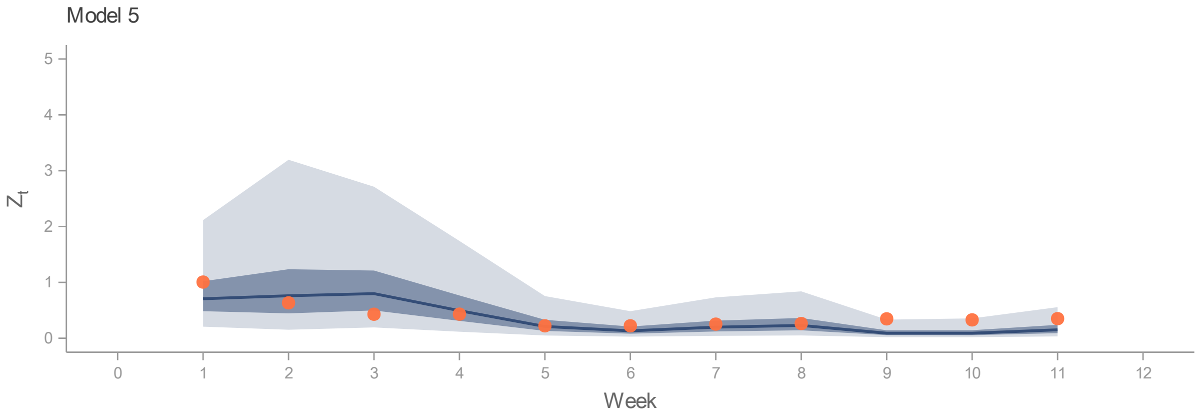

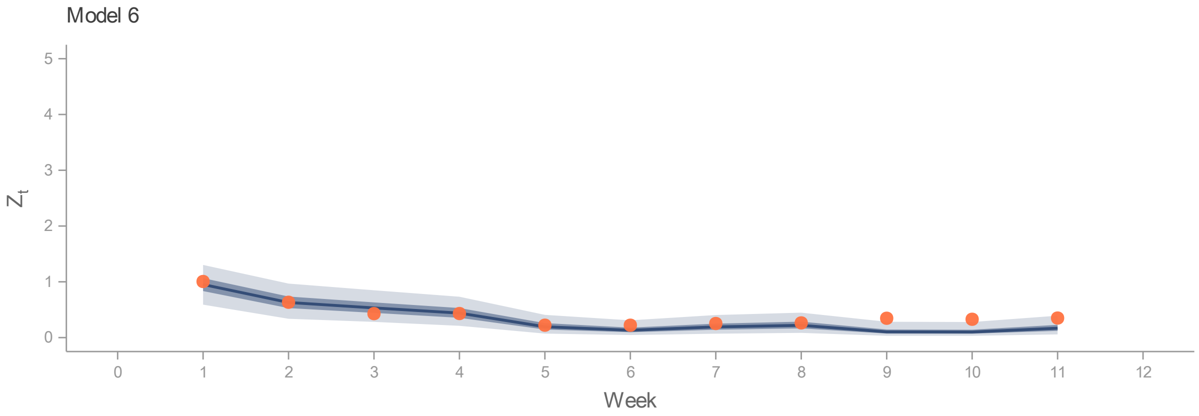

1.3 Effective reproduction number

Likewise, the difference in uncertainties is transferred to the predicted $\Re_t$.

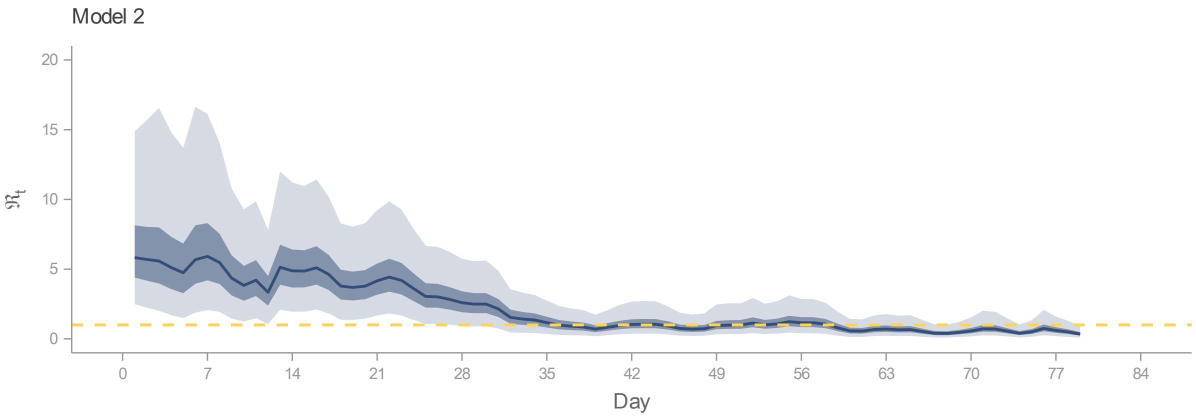

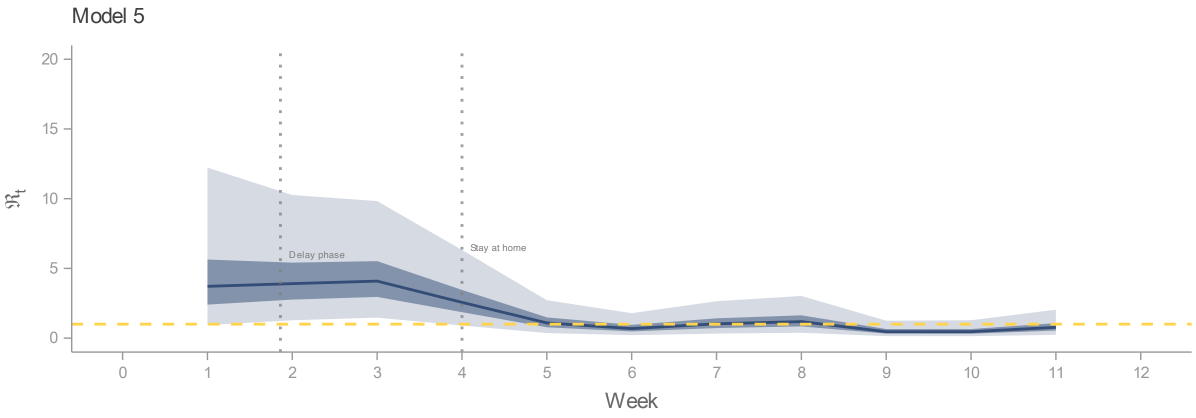

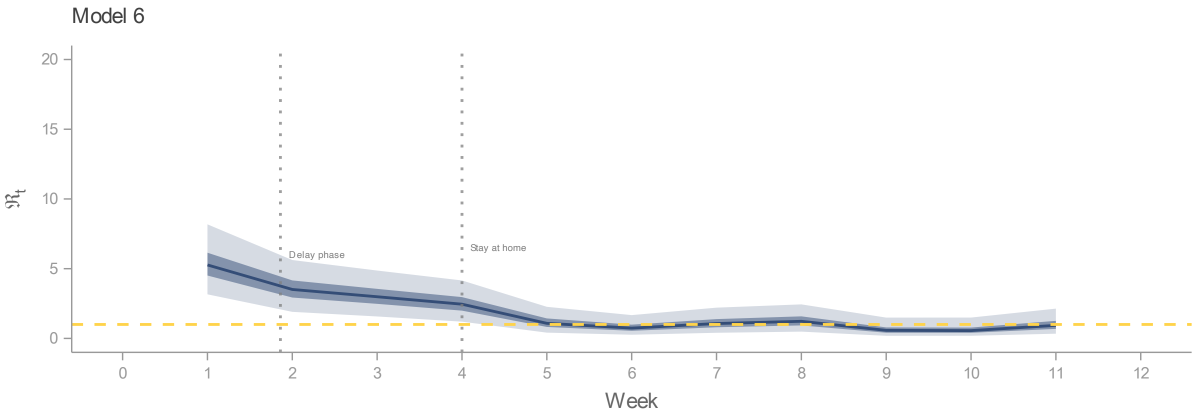

1.4 Parameter estimates

The inference of time-independent parameters across Models 2, 5, and 6 reinforces the message outlined in the preceding sections: Models 2 and 5 estimate similar quantities, whereas Model 6 yields more precise estimates (due to the incorporation of mobility data).

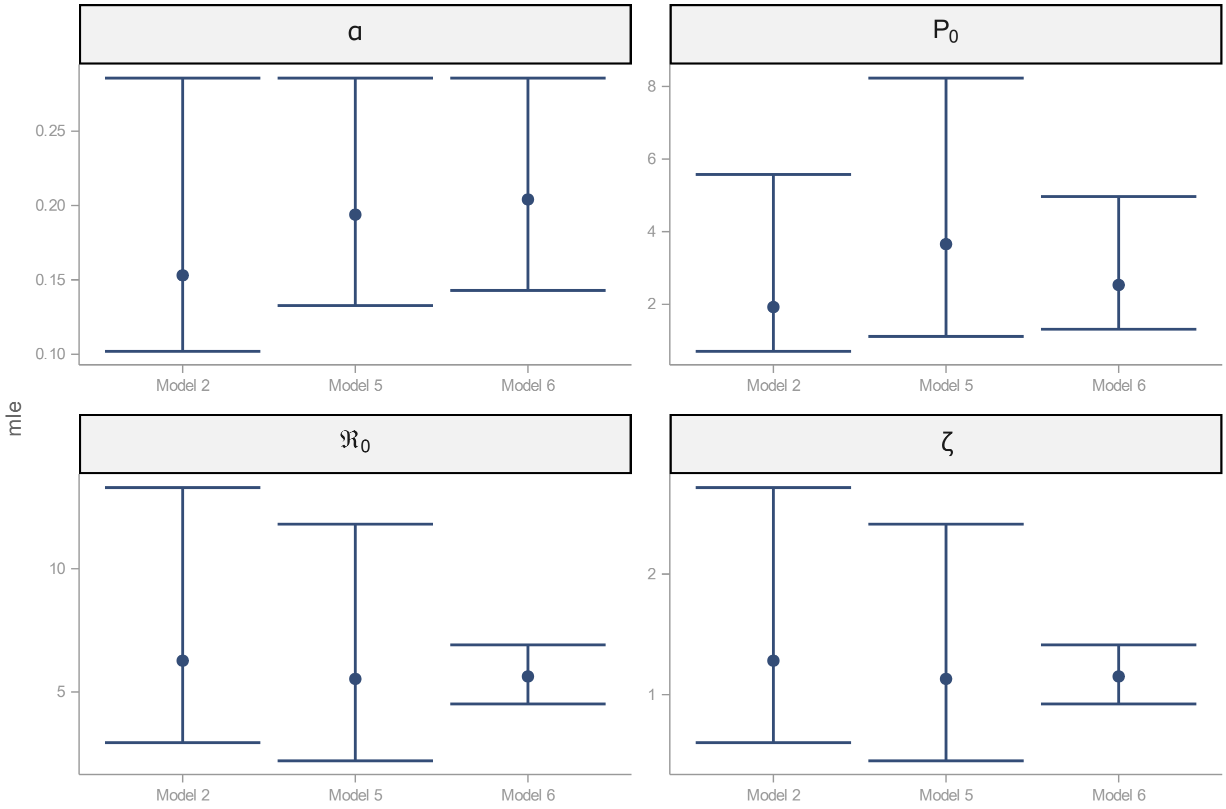

2 Simulations from MLE

This section is added for reproducibility purposes.

We simulate DGP1 and DGP2 using the Maximum Likelihood Estimate (obtained in S5 and S6, respectively). These simulations produce sample trajectories of β_t, which are showcased in the main document.

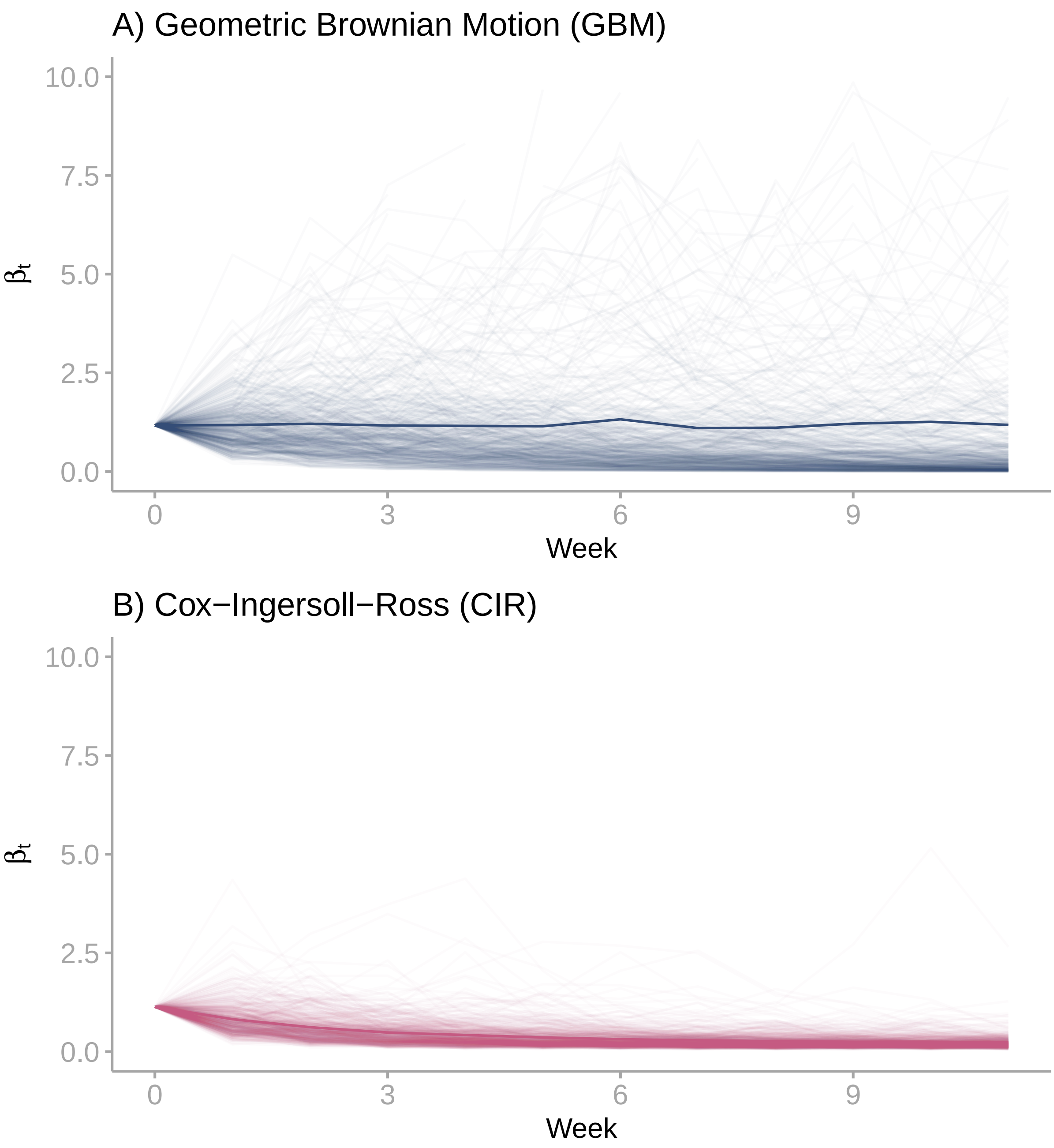

3 Hidden states by DGP

This section is added for reproducibility purposes.

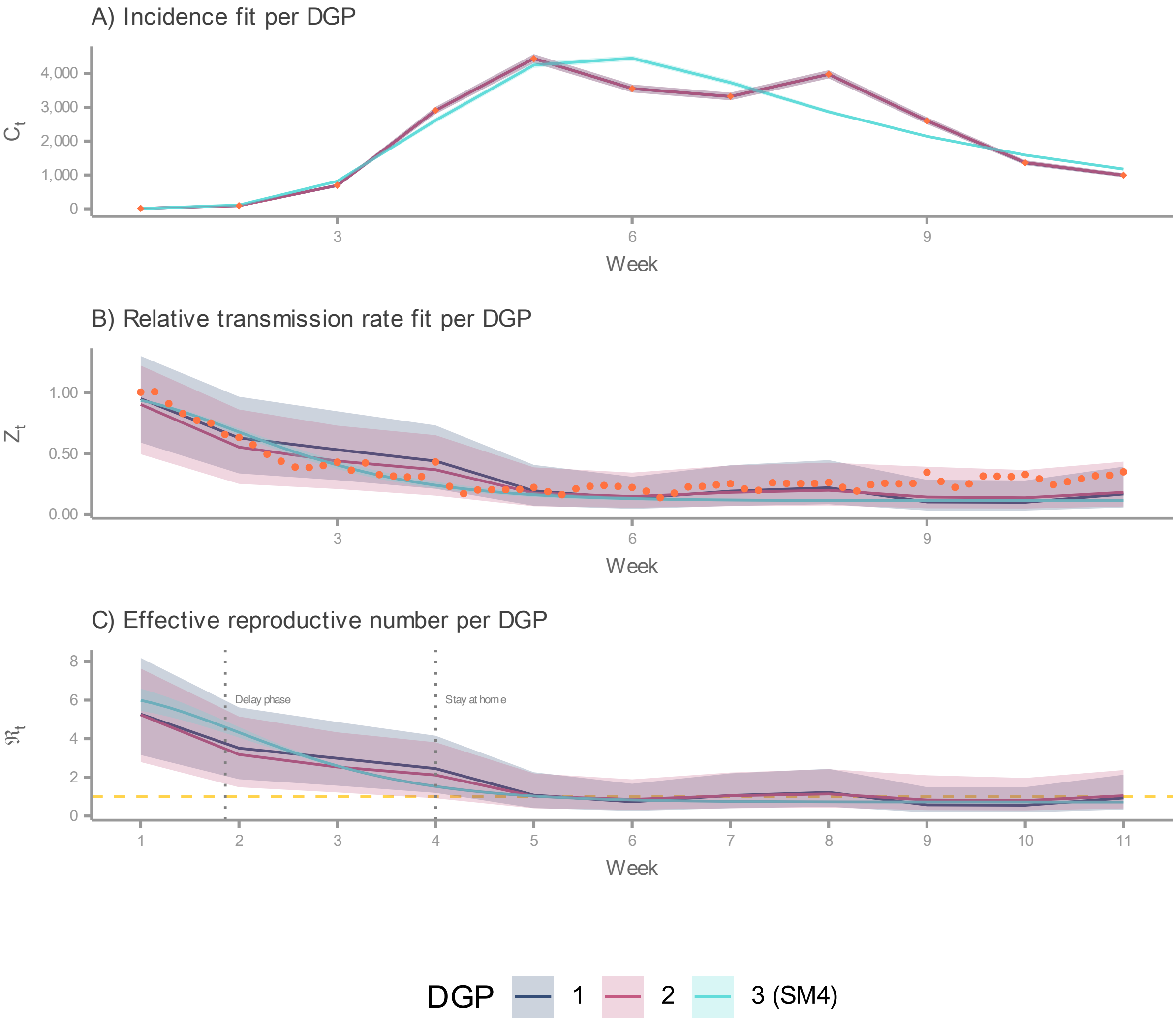

Supplement: S8 Text — (PDF) [file pcbi.1010206.s008.pdf]
